# FKBP10 promotes clear cell renal cell carcinoma progression and regulates sensitivity to the HIF2α blockade by facilitating LDHA phosphorylation

**DOI:** 10.1038/s41419-024-06450-x

**Published:** 2024-01-17

**Authors:** Ren Liu, Zhihao Zou, Lingwu Chen, Yuanfa Feng, Jianheng Ye, Yulin Deng, Xuejin Zhu, Yixun Zhang, Jundong Lin, Shanghua Cai, Zhenfeng Tang, Yingke Liang, Jianming Lu, Yangjia Zhuo, Zhaodong Han, Xiaohui Ling, Yuxiang Liang, Zongren Wang, Weide Zhong

**Affiliations:** 1 Department of Urology, The First Affiliated Hospital, Sun Yat-sen University, Guangzhou, China; 2 Department of Urology, Guangdong Key Laboratory of Clinical Molecular Medicine and Diagnostics, Guangzhou First People’s Hospital, School of Medicine, South China University of Technology, Guangzhou, China; 3 Graduate School of Guangzhou Medical University, Guangzhou Lab, Guangzhou Medical University, Guangzhou, China; 4 Department of Urology, Minimally Invasive Surgery Center, Guangdong Key Laboratory of Urology, Guangzhou Urology Research Institute, The First Affiliated Hospital of Guangzhou Medical University, Guangzhou Medical University, Guangzhou, China; 5 Department of Urology, Affiliated Cancer Hospital and Institute of Guangzhou Medical University, Guangzhou, China; 6 Reproductive Medicine Centre, Huizhou Central People’s Hospital, Huizhou, 516001 Guangdong China

**Keywords:** Renal cell carcinoma, Renal cell carcinoma

## Abstract

Renal cell carcinoma (RCC) is one of the three major malignant tumors of the urinary system and originates from proximal tubular epithelial cells. Clear cell renal cell carcinoma (ccRCC) accounts for approximately 80% of RCC cases and is recognized as a metabolic disease driven by genetic mutations and epigenetic alterations. Through bioinformatic analysis, we found that FK506 binding protein 10 (FKBP10) may play an essential role in hypoxia and glycolysis pathways in ccRCC progression. Functionally, FKBP10 promotes the proliferation and metastasis of ccRCC in vivo and in vitro depending on its peptidyl-prolyl cis-trans isomerase (PPIase) domains. Mechanistically, FKBP10 binds directly to lactate dehydrogenase A (LDHA) through its C-terminal region, the key regulator of glycolysis, and enhances the LDHA-Y10 phosphorylation, which results in a hyperactive Warburg effect and the accumulation of histone lactylation. Moreover, HIFα negatively regulates the expression of FKBP10, and inhibition of FKBP10 enhances the antitumor effect of the HIF2α inhibitor PT2385. Therefore, our study demonstrates that FKBP10 promotes clear cell renal cell carcinoma progression and regulates sensitivity to HIF2α blockade by facilitating LDHA phosphorylation, which may be exploited for anticancer therapy.

## Introduction

Renal cell carcinoma (RCC) originates from the epithelial cells of the renal tubules and is one of the three major urological malignancies and the 14th most commonly occurring malignancy globally [1]. Clear cell renal cell carcinoma (ccRCC) accounts approximately 80% of RCC cases and has the highest mortality. ccRCC gets its name from the large intracellular lipid droplets and glycogen deposits, which form vacuolar clear cell structures [2]. This suggests profoundly altered lipid and glucose metabolism during the development of ccRCC. Therefore, renal cancer is widely considered a metabolic disease [3]. Renal carcinoma cells are known to exhibit the classic Warburg effect of dramatically accelerated glycolysis and upregulated expression of glycolytic pathway enzymes [4]. Tumor cells utilize lactate dehydrogenase (LDHA), a key regulatory enzyme of the glycolytic pathway, for lactate conversion from pyruvate. In contrast to oxidative phosphorylation, aerobic glycolysis produces less adenosine triphosphate (ATP), which requires high glucose consumption and lactate production to sustain the proliferation of tumors [5].

The above significant metabolic alterations in ccRCC can be attributed to its distinct epigenetic characteristics. In ccRCC, germline loss-of-function mutations in the tumor suppressor gene von Hippel‒Lindau (VHL) lead to dysregulated expression of the hypoxia inducible factor (HIF) family and their associated cancer-promoting mediators [6]. The VHL-HIF axis is one of the most commonly activated pathways in ccRCC, and VHL inactivation leads to stable expression of HIF1α and HIF2α (encoded by HIF1A and EPAS1). In recent years, two first-class HIF2α inhibitors, PT2385 and PT2977, had entered clinical trials and achieved good therapeutic effects [7, 8]. In 2022, PT2977 (renamed belzutifan) was approved by the US FDA for the clinical treatment of ccRCC patients [9]. However, many patients showed intrinsic resistance or develop acquired resistance [10, 11]. Therefore, an in-depth exploration of more efficient therapeutic targets related to the sensitivity of HIF2α inhibitors is of great importance in rational clinical therapeutic application of ccRCC.

In this study, bioinformatics analyses were performed by screening ccRCC single-cell datasets based on the hallmarks of the hypoxia pathway and glycolysis, and it was found that FKBP10 (FK506 binding protein 10) may play an important role in the progression of ccRCC. FKBP10 belongs to the FKBP family which contain the characteristic active PPIase domain that can catalyze the interaction of specific imideophilic bonds between cis/trans prolyl conformers, resulting in a rate-limiting change in protein conformation [12]. Members of this family play important roles in many signaling pathways involved in inflammation, adaptive immune response, cancer, and developmental biology and are associated with several cellular processes, including protein folding, stability and trafficking, kinase activity, and receptor signaling [13]. Among others, FKBP10 contains four PPIase domains, which is the one with the most PPIase domains in the FKBP family. Studies have found that FKBP10 is involved in the progression of idiopathic pulmonary fibrosis [14], osteogenesis imperfecta [15] and cancers including lung cancer [16, 17], ovarian cancer [18], prostate cancer [19] and leukemia [20]. Multiple studies have suggested that FKBP10 is highly expressed in tumor tissues and is considered as a therapeutic target for ccRCC through experimental and bioinformatics approaches, but there is a lack of further in-depth studies [21–24].

## Materials and methods

### Patients and tissues

The study was authorized by the Ethics Committees of the First Affiliated Hospital of Sun Yat-sen University (IIT-2023-009) and the Guangzhou First People’s Hospital (Approval No. K-2021-072K-2021-072). 79 paraffin-embedded tissues from patients with ccRCC were provided by the pathology department of the First Affiliated Hospital of Sun Yat-sen University and 9 paired samples of ccRCC and paracancerous tissues were derived from Guangzhou First People’s Hospital. Prior to the collection of patient samples, informed consent was obtained from all subjects, and the clinical features of the ccRCC patients are summarized in Supplementary Table. None of the patients had received radiotherapy, chemotherapy, or other treatments. Pathological evaluation of each case was performed by two pathologists separately.

Commercial tissue microarrays (TMAs) were purchased from Shanghai Outdo Biotech Company (HKidE030PG02) containing 30 cases of ccRCC tissues with clinical information and from Xian Bioaitech Company (u090Ki01) containing 80 cases of ccRCC tissues and 10 cases of normal kidney tissues.

### Cell culture and transfection

Human renal tubular epithelial HK2 cells and the ccRCC cell lines ACHN, 786 O and OSRC2 were purchased from the American Type Culture Collection (Manassas, MD, USA). The ccRCC cell lines Caki1 and RCC4 were purchased from Beina Chuanglian Biotechnology Institute (Beijing, China). All cell lines were tested for mycoplasma and short tandem repeat (STR) profiles. Cells were incubated at 37 °C and 5% CO_2_. Hypoxic cell culture and cell transfection with plasmids were performed as described previously [25].

### Plasmids and reagents

The DNA sequences of human FKBP10 (wild-type and mutations), LDHA (wild-type and mutations), HIF1α and VHL were synthesized by Tsingke Biotechnology Co., Ltd. (Beijing, China) and loaded into the CSII-EF-MCS-IRES-Venus vector. The short hairpin RNA (shRNA) sequence and the primers for qRT‒PCR were purchased from Tsingke Biotechnology Co., Ltd. (Beijing, China), and then ligated into the pLVX-Puro-GFP or pLKO.1-NEO-mCherry empty vector to construct the shRNA plasmid. All the shRNA sequences are provided in the Table S5. The antibodies used in this study are listed in the Supplementary Table 1. AZD4547 (S2801) was purchased from Selleck (Houston, TX, USA). PT2385 (HY-12867) was purchased from MCE (Shanghai, China). DMOG (MB4331) and MG132 (MB5137) were purchased from Meilunbio (Dalian, China).

### In vivo mouse experiments

The animal use protocol in this study was reviewed and approved by the Animal Ethical and Welfare Committee (AEWC). BALB/c nude mice and NCG mice were purchased from GemPharmatech Co., Ltd (Shanghai, China) and bred in an SPF-grade experimental animal room (Forevergen, Guangzhou, China).

For the renal orthotopic tumor model, 40 BALB/c nude mice (4 weeks old, male) were anesthetized with an intraperitoneal injection of 40 mg/kg sodium pentobarbital (1%) and then randomized into 4 groups. A total of 5 × 10^5^ luciferase-transduced tumor cells in 10 µL PBS were injected into the subcapsular space of the right kidney. Six weeks after injections, all mice were sacrificed, and orthotopic xenograft tumors were extracted. Tumor weights were estimated by subtracting the weight of the contralateral kidney from the weight of the tumor-implanted kidney.

To establish the lung metastasis model, 1 × 10^6^ luciferase-transduced tumor cells in 50 µL PBS were administered intravenously via tail vein infusion. After six weeks, the mice were euthanized, lungs were harvested, and metastases were counted.

Tumor growth and metastasis were monitored by an in vivo imaging system (IVIS Lumina Series III, PerkinElmer, MA, USA). D-Luciferin (150 mg/kg, Yeasen Biotechnology, Shanghai, China) was intraperitoneally injected for 10 min before imaging.

To create a patient derived xenograft (PDX) model, tumor fragments mixed with Matrigel were subcutaneously implanted into immunodeficient NCG mice. Tumors were removed from the mice when the diameter of the tumor reached 1 cm and were passaged for two generations in the NCG mice. When the PDX model had been engrafted into third-generation of mice, it was considered a successful PDX model. For drug treatment assays, the mice were randomized into 4 groups when the volume of each tumor was approximately 100 mm^3^. Ten mice per group were intratumorally injected with FKBP10 shRNA AAV (GeneChem, Shanghai, China) or the control shRNA AAV, which was performed daily for 5 days. Starting at the second AAV administration, mice received concomitant oral administration with the HIF2α inhibitor PT2385 (10 mg/kg) or PBS twice daily respectively until the endpoint.

### qRT‒PCR

RNA extraction was conducted using the TRIzol Reagent (15596018, Gibco, MA, USA). Total RNA was reverse transcribed into cDNA using a HiScript II Q-RT SuperMix kit ( + qDNA wiper) (Q71102, Vazyme, Nanjing, China). qRT‒PCR was performed for quantification of mRNA according to our previous study [25]. Sequences of the DNA primers for qRT‒PCR are provided in Table S6.

### Immunoprecipitation and immunoblotting

Cell lysates were collected for coimmunoprecipitation (Co-IP) analysis using an immunoprecipitation kit (#88805, Thermo Fisher Scientific, MA, USA). Briefly, protein A/G magnetic beads were incubated with specific antibodies for 4 h, and the beads were washed with modified coupling buffer. Then, the antibody was crosslinked to beads with DSS and incubated with cell lysate overnight at 4 °C. The beads were washed three times with IP lysis/wash buffer and eluted with elution buffer.

The eluate was mixed with loading buffer, heated for 5 min in a 100 °C water bath, cooled to RT, and subjected to SDS‒PAGE. Western blot analysis was performed as previously described [25]. The antibodies used for Western blot analysis are listed in the Table S3.

### Seahorse assay

Seahorse assays were performed to detect the extracellular acidification (ECAR) and the oxygen consumption rate (OCR) by using an XFe24 Extracellular Flux Analyzer (Agilent, California, USA). Mitochondrial function was determined by measuring the level of OCR via the XFe24 Cell Mito Stress Test (100850, Agilent) and the level of glycolysis was detected by measuring ECAR using the XFe24 Glycolytic Rate Assay (103344, Agilent).

### LDH activity assay and lactate production assay

The evaluations of LDH activity and lactate production were determined using commercially available kits (BC0685 and BC2230, Solarbio, Beijing, China) according to the manufacturer’s instructions.

### In vitro pull-down assay

Recombinant Flag-tagged FKBP10 was expressed and purified from HEK293 cells. Recombinant His-tagged LDHA was purchased from Abcam (ab93699, Cambridge, UK). His pull-down assays were performed using HisPur Cobalt resin (Thermo Fisher Scientific, 21277, MA, USA) by incubating with FKBP10 proteins at 4 °C overnight. Flag pull-down assays were performed by incubating Flag-tagged FKBP10 proteins immobilized on anti-DYKDDDDK affinity resin (Thermo Fisher Scientific, A36803, MA, USA). Interacting proteins were eluted by imidazole elution buffer and subjected to SDS‒PAGE analysis.

### In vitro kinase assay

Recombinant His-tagged FGFR1 (ab291115, Abcam, Cambridge, UK) was incubated with His-tagged LDHA and Flag-tagged FKBP10 proteins in kinase buffer containing 150 mM NaCl, 5 mM DTT, 10 mM MnCl_2_, 0.01% Trition-100, 10 mM HEPES (pH 7.5) and 200 mM ATP at 30 °C for 60 min. The reaction was terminated by dipping in a boiling water bath for 5 min. The proteins were snap-frozen in liquid nitrogen and subjected to Western blot analysis of LDHA-Y10 phosphorylation.

### CUT&RUN

CUT&RUN assays were performed according to a previous study [25]. In brief, 3×10^5^ RCC4 cells were bound to concanavalin A-coated magnetic beads and incubated with 5 μg of HIF1α or HIF2α antibody for 8 h at 4 °C after membrane permeabilization with digitonin. Then, protein A-MNase was added to the cell-bead slurry and activated by incubation with 2 mM CaCl_2_ on ice for 30 min. Spike-in control DNA was added to each sample for normalization. Samples were then incubated for 30 min at 37 °C to release the CUT&RUN fragments, followed by DNA extraction with a DNA purification kit (14209, CST, MA, USA). DNA products were quantified by qRT‒PCR relative to input, and the primers are described in the Supplementary Table 3.

### Bioinformatic analysis

Bioinformatic analysis was performed using a public single-cell RNA sequencing data from 13 ccRCC patients [26]. To evaluate the activation of hypoxia- and glycolysis-pathways in ccRCC patients, the gene sets of “HALLMARK-HYPOXIA” and “HALLMARK-GLYCOLYSIS” were obtained from the Molecular Signatures Database (MSigDB version 6.0). The AUCell algorithm was used to describe the activity of specific sets of genes in ccRCC cells and divide the cells into high activity and low activity groups according to the degree of variation in AUC scores. The tSNE algorithm was used for clustering data and screening and ranking differentially expressed genes according to *P* values. All bioinformatic analyses were performed in R software (version 4.0.2) and respective R packages.

### Statistical analysis

SPSS software (version 23.0) and GraphPad Prism 7 were used for statistical analysis in this study. The results were analyzed using an independent sample t test. Survival analysis was carried out using Kaplan‒Meier curves and log-rank tests. Correlations were analyzed using Pearson correlation. A *P* value less than 0.05 signified that the groups differed statistically.

## Results

### FKBP10 may play an important role in hypoxia and glycolysis pathways

**Fig. 1:**
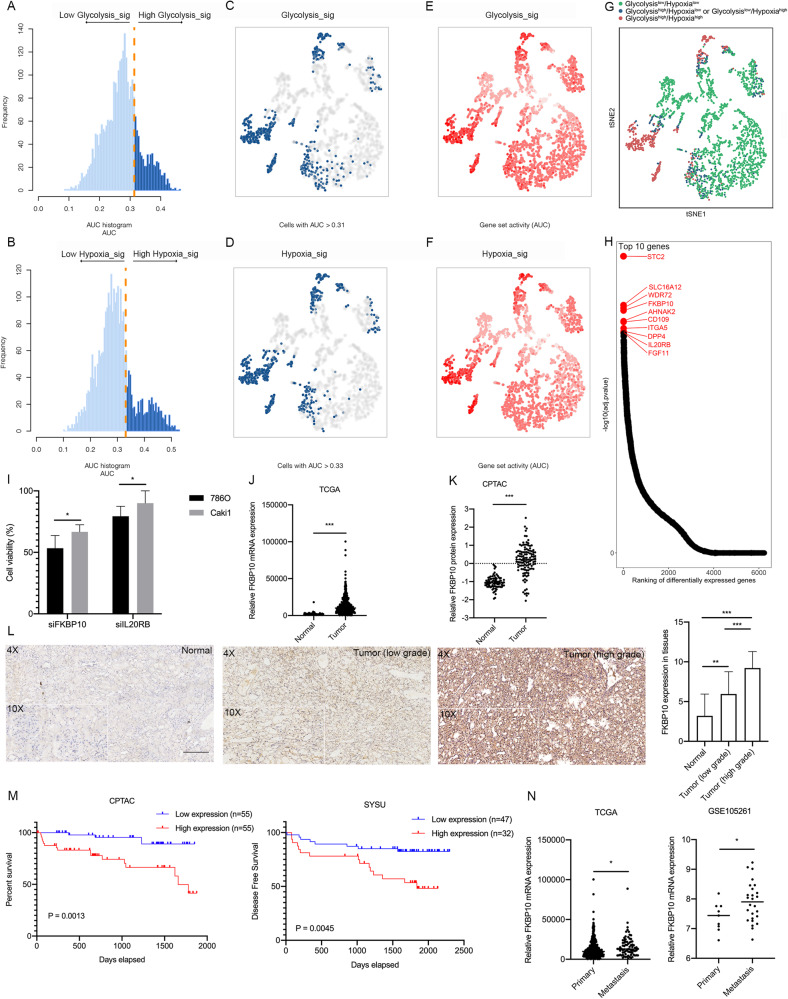
FKBP10 may play an important role in ccRCC progression and indicate poor prognosis in ccRCC patients. **A**, **B** The distribution of AUC and cell frequency. **C**, **D** tSNE diagrams for cells with AUCs higher than the selected threshold. Blue dots indicate cells with higher AUC scores, and gray dots indicate cells with lower AUC scores. **E**, **F** tSNE diagrams for glycolysis-sig and hypoxia-sig activities that colored by AUC. **G** The tSNE diagram of three groups divided by glycolysis-sig and hypoxia-sig. **H** Ranking plot according to *P* values. **I** Cell viability assay of 786 O cells in different treatment groups for 72 h. **J**, **K** FKBP10 expression in ccRCC and normal kidney tissues from TCGA-KIRC or CPTAC-KIRC datasets. **L** Representative images of FKBP10 immunohistochemistry staining in ccRCC and adjacent normal tissues. Scale bar: 100 µm. The histogram indicates the IHC scores of FKBP10 expression. **M** Kaplan‒Meier curves for OS or DFS between the FKBP10 low and high expression groups. **N** FKBP10 expression in primary ccRCC tissues and metastatic ccRCC tissues from TCGA or GSE105261. The data are shown as the means ± SD. Statistical analyses were performed with a two-tailed unpaired Student’s *t* test (*P < 0.05, **P < 0.01, ***P < 0.001).

Data derived from a ccRCC single-cell sequencing database containing 13 patients were analyzed using the AUCell algorithm based on the hallmarks of hypoxia or glycolysis pathways respectively. Per-cell AUC scores were calculated using the ‘AUCell’ package according to the activity of a specific gene set. Cells were divided into a high-activity group and a low-activity group according to the degree of variation in the AUC scores (Fig. 1A, B). In Fig. 1C, D, dark blue indicates high activity cells, and in Fig. 1E, F, the depth of the color represents the level of the AUC score. Using the tSNE algorithm, all cells were divided into three groups (Glycolysis^low^/Hypoxia^low^ group, Glycolysis^high^/Hypoxia^low^ or Glycolysis^low^/Hypoxia^high^ group and Glycolysis^high^/Hypoxia^high^ group). A total of 464 differentially expressed genes were screened between the Glycolysis^low^/Hypoxia^low^ group and the Glycolysis^high^/Hypoxia^high^ group ( | log2FC | > 1, FDR-adjusted *P* < 0.01) (Fig. 1G). The top ten differentially expressed genes according to the *P* values were showed in Fig. 1H. We next analyzed TCGA ccRCC cohort data via a Kaplan-Meier survival and found that only FKBP10 and IL20RB negatively correlated with patients’ overall (Fig. S1A). Further cell viability studies were performed, showing that FKBP10 knocking down had more pronounced inhibitory effect than IL20RB on 786 O and Caki1 cells (Fig. 1I). Therefore, FKBP10 was selected for further study.

### Highly expressed FKBP10 indicates poor prognosis of ccRCC patients

To preliminarily explore the expression of FKBP10 in human ccRCC tissues, we analyzed the mRNA sequencing data of 530 ccRCC tissues and 72 benign renal tissues in the TCGA database and the protein sequencing data of 118 ccRCC tissues and 84 renal benign tissues in the CPTAC database. The results showed that the mRNA and protein levels of FKBP10 were significantly increased in tumor tissues compared to benign tissues (Fig. 1J, K). The higher the mRNA or protein expression of FKBP10 in tumor tissues, the higher the pathological grade or TNM stage of the tumor (Fig. S1B–E). To confirm this finding, Western blot assays were performed to detect 9 paired samples of ccRCC and paracancerous tissues. The results showed that FKBP10 protein expression in tumor tissues was significantly higher than that in adjacent benign tissues (Fig. S1F). Similarly, various ccRCC cell lines (786 O, Caki1, 769 P, RCC4, OSRC2 and ACHN) showed higher expression of FKBP10 than renal tubular epithelial cells (HK2) (Fig. S1F).

IHC results revealed that the expression of FKBP10 in ccRCC tissues was significantly higher than that in benign tissues. Moreover, FKBP10 was more highly expressed in pathological high-grade tumors (Grade 3/4) than in low-grade tumors (Grade 1/2) (Fig. 1L, Table S1). By analyzing TCGA-KIRC, GSE105261 datasets and an our RCC cohort, we found that metastatic ccRCC patients showed higher FKBP10 expression, which indicated that FKBP10 might promote the metastasis of ccRCC (Fig. 1N, Table S1). Kaplan‒Meier survival analyses showed that high FKBP10 levels had worse prognosis (Fig. 1M). Univariate and multivariate Cox analysis confirmed that the high expression of FKBP10 was correlated with poor patient DFS in our RCC cohort (Table S2).

### FKBP10 promotes ccRCC proliferation, migration and invasion

**Fig. 2:**
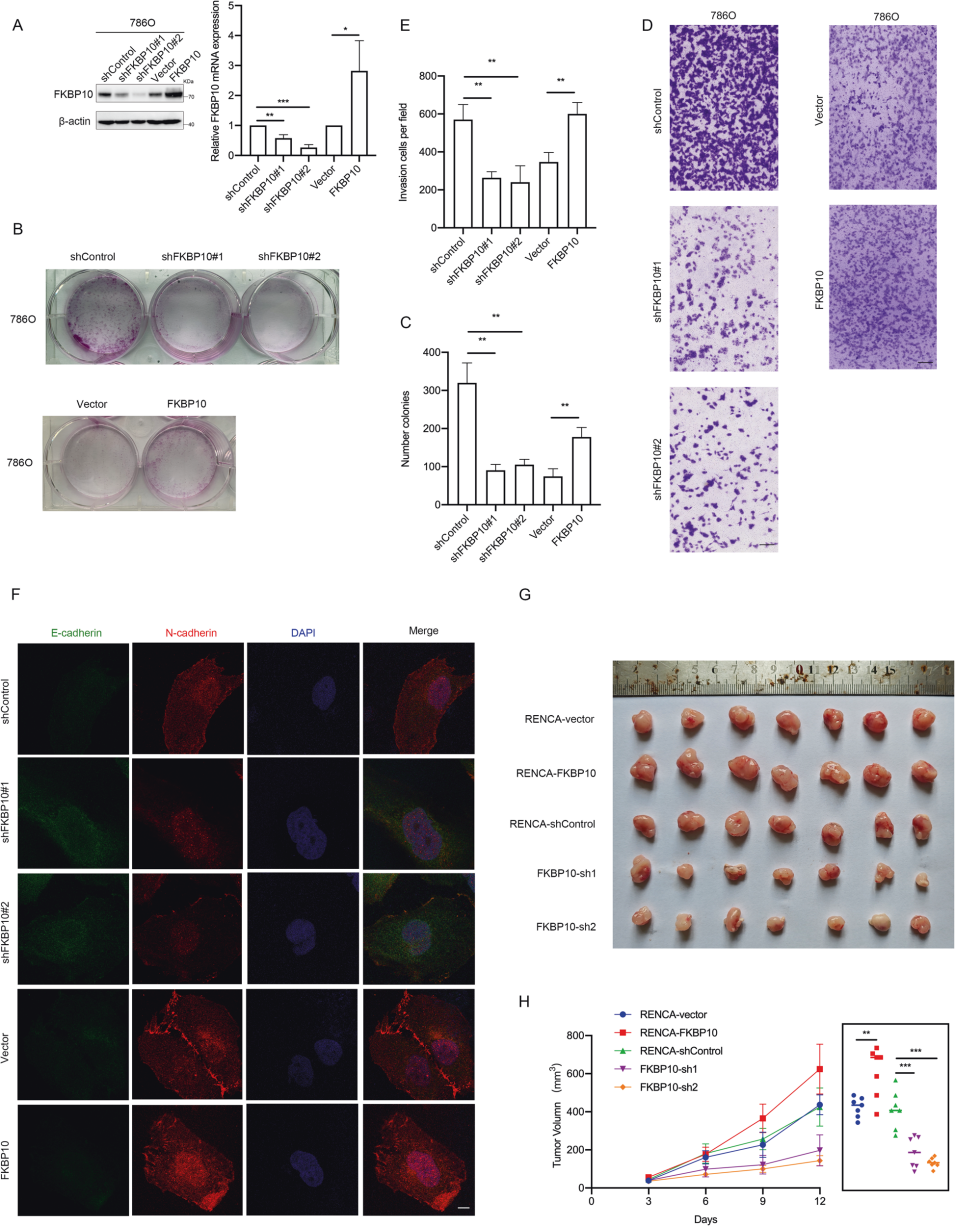
FKBP10 promotes ccRCC proliferation, migration and invasion. **A** The overexpression or knockdown of FKBP10 in 786 O cells was measured by Western blot and qRT‒PCR assays. **B**, **C** Cell proliferation abilities were measured by colony formation assays. **D**, **E** Cell invasion abilities were measured by Transwell assays. Scale bar: 100 µm. Data are presented as the means ± SD, and were independently replicated three times. **F** Representative images of immunofluorescence staining for E-cadherin (green) and N-cadherin (red) (magnification ×100). DAPI (blue) was applied for nuclear staining. Scale bar: 10 µm. **G**, **H** Gross appearance of the tumors and tumor growth curves of each group are shown. Statistical analyses were performed with a two-tailed unpaired Student’s *t* test (NS, *P* > 0.05, * *P* < 0.05, **P < 0.01, ***P < 0.001).

To explore the biological function of FKBP10 in ccRCC, 786 O and Caki1 cell lines with stable FKBP10 overexpression or knockdown were established, and cells transfected with empty vectors were used as negative controls (Fig. 2A and S2E). The results of the CCK8 assay and plate colony formation assay suggested that overexpression or knockdown of FKBP10 could significantly enhance or inhibit the proliferation of ccRCC cells, respectively (Fig. 2B, C and S2A–D). The results of wound healing assays and Transwell assays showed that FKBP10 could significantly promote the migration and invasion ability of ccRCC cells (Fig. 2D, E, S1F–K).

Previous studies have shown that the epithelial-mesenchymal transition (EMT) pathway, which has been proposed as a critical mechanism during cancer progression and metastasis, also plays a key role in metastatic ccRCC [27]. Therefore, Western blot analysis and confocal microscopy were performed, and we detected that FKBP10 promoted the expression of N-cadherin and inhibited the expression of E-cadherin but did not affect the expression of Vimentin (Fig. 2F and S2L). To further explore the tumorigenic capacity of FKBP10 in ccRCC, we used cell lines derived xenograft (CDX) model subcutaneously injected with RENCA cells (Fig. 2G, H and S2M), which showed that FKBP10 knockdown or overexpressed could inhibit or promote tumor growth in vivo. Collectively, FKBP10 promotes ccRCC progression in vitro and in vivo.

### FKBP10 can directly bind with LDHA

**Fig. 3:**
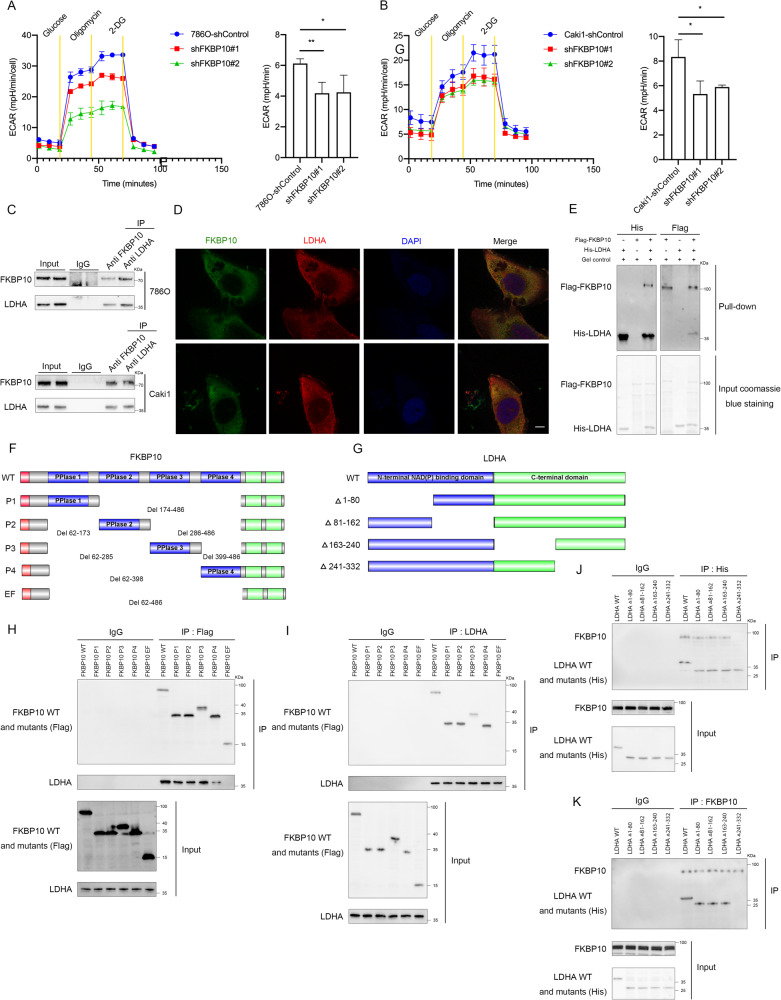
FKBP10 can directly bind with LDHA. **A**, **B** Extracellular acidification (ECAR) of 786 O and Caki1 cells with FKBP10 knockdown. Data are presented as the means ± SD and were independently replicated three times. **C**, **D** Immunoprecipitation analyses of the binding of endogenous FKBP10 and LDHA in 786 O and Caki1 cells. The cellular localizations of FKBP10 and LDHA were analyzed by immunofluorescence (magnification ×100). Cell nuclei were stained with DAPI (blue). Scale bar: 10 µm. **E** In vitro pull-down assays indicated that FKBP10 directly bound with LDHA. **F** Schema of deletion mutants of FKBP10. Domains illustrated include signal peptide (red), PPlase (blue), and EF hand (green). **G** Schema of LDHA deletion mutants. The N-termini are labeled in blue, and the C- termini are green. **H**, **J** Co-IP assays of exogenous FKBP10 with different LDHA fragments or exogenous LDHA with different FKBP10 mutations in HEK293 cells. IgG was used as a negative control for immunoprecipitation. **I**, **K** Co-IP analyses of the interaction between various regions of FKBP10 and endogenous LDHA or various regions of LDHA and endogenous FKBP10 in 786 O cells. IgG served as the negative control. Statistical analyses were performed with a two-tailed unpaired Student’s *t* test (*P < 0.05, **P < 0.01).

Previous bioinformatics screening showed that FKBP10 may play a significant role in the regulation of glycolysis in ccRCC. Aerobic glycolysis, as an important energy source for tumor cells, exerts crucial effects on tumor progression. Using a Seahorse XF24 analyzer, we found that the level of extracellular acidification (ECAR), which indirectly reflects glycolytic capacity and glycolysis in ccRCC cells, was significantly inhibited after knocking down FKBP10 expression (Fig. 3A, B). In contrast, the oxygen consumption rate (OCR), which reflects the mitochondrial oxidative phosphorylation level of tumor cells, was significantly increased as a compensatory mechanism (Fig. S3A, B). The above results revealed that FKBP10 can regulate glycolysis in ccRCC cells.

In the next step, we aimed to explore the mechanism by which FKBP10 regulates glycolysis in ccRCC. FKBP10 is a ubiquitously expressed chaperone protein with four PPIase domains that regulate protein folding and activity. Coimmunoprecipitation of an anti-FKBP10 antibody and mass spectrometry analysis were performed (Figure S3D). According to the results of IP-MS (Table S4) combined with computer modeling (https://www.rcsb.org/3d-view) (Fig. S3C), we speculated that FKBP10 may exert a regulatory function by binding with LDHA. Coimmunoprecipitation assays with endogenously expressed FKBP10 and LDHA in 786 O cells confirmed that FKBP10 interacted with LDHA (Fig. 3C). Furthermore, immunofluorescence colocalization showed that FKBP10 and LDHA were coexpressed in 786 O and Caki1 cells (Fig. 3D). In addition, in vitro His- and Flag-pull-down assays showed that FKBP10 was directly bound to LDHA (Fig. 3E).

We next mapped the critical region of FKBP10 and LDHA responsible for their binding sites to each other. FKBP10 was divided into five Flag-tagged fragments, including FKBP10-P1 (Del 174-486), FKBP10-P2 (Del 62-173 and Del 286-486), FKBP10-P3 (Del 62-285 and Del 399-486), FKBP10-P4 (Del 62-398) and FKBP10-EF (Del 62-486) (Fig. 3F); LDHA was divided into four His-tagged fragments, including LDHA-△1-80, LDHA-△81-162, LDHA-△163-240 and LDHA-△241-332, to narrow down the binding region (Fig. 3G). Coimmunoprecipitation results showed that exogenous FKBP10 in 293 T cells could bind to LDHA-WT and other fragments except LDHA-△241-332 (Fig. 3H); exogenous LDHA could bind to FKBP10-WT and other fragments except FKBP10-EF (Del 62-486) (Fig. 3J). The above results preliminarily confirmed that FKBP10 directly binds to the C-terminal region of LDHA (positions 241-332), and FKBP10 formes a complex with LDHA through its four PPIase domains, indicating that one PPIase domain is sufficient for FKBP10 to bind with LDHA. Further coimmunoprecipitation assays with endogenously expressed FKBP10 and LDHA in 786 O cells showed the consistent results (Fig. 3I and K).

### FKBP10 promotes the phosphorylation of the LDHA-Y10 site

**Fig. 4:**
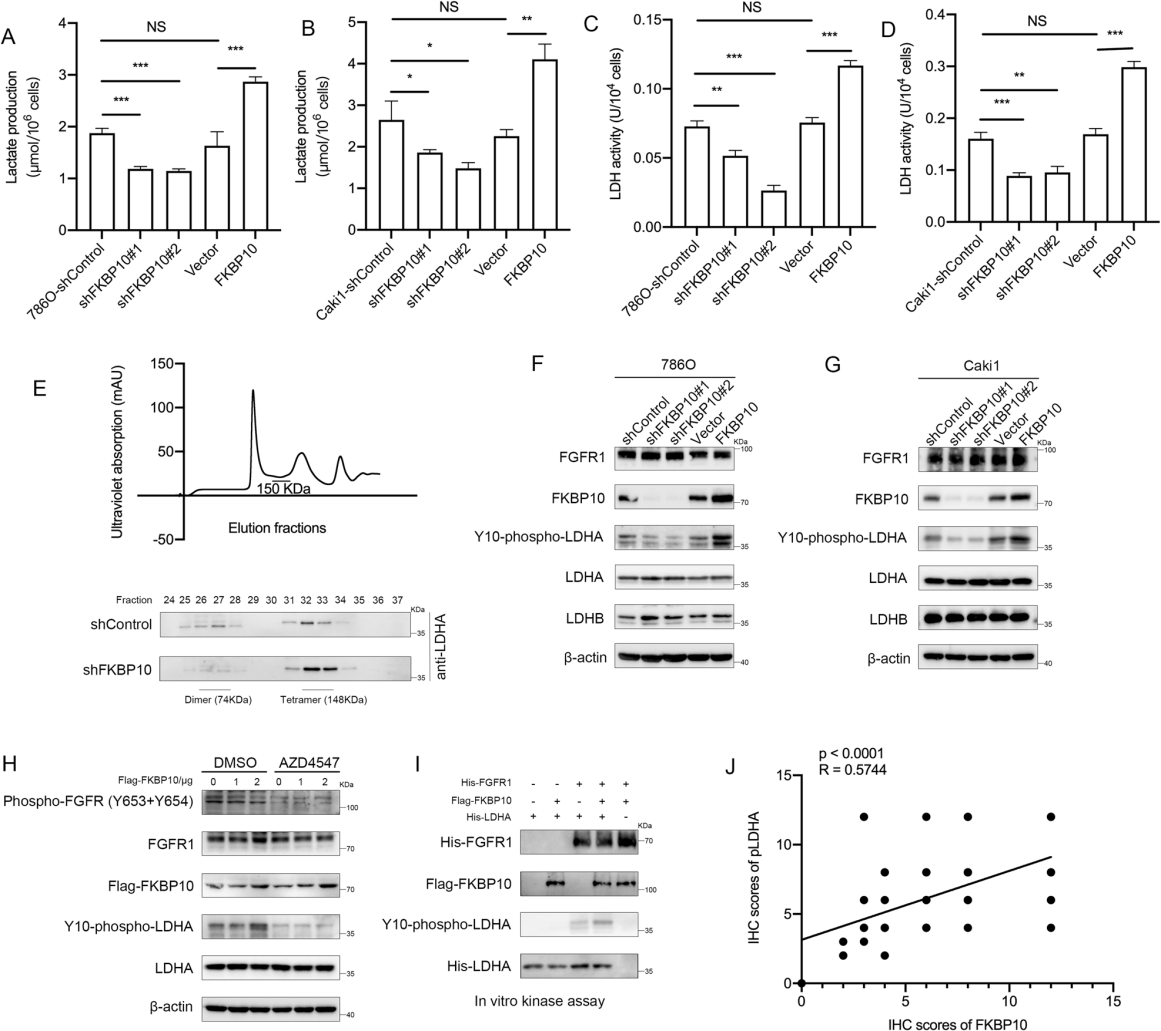
FKBP10 promotes the phosphorylation of the LDHA-Y10 site. **A**–**D** Lactate production levels and LDH enzyme activity in FKBP10-overexpressing or FKBP10-knockdown cells were examined as indicated. Data are presented as the means ± SD, and were independently replicated three times. **E** Gel filtration analysis of whole lysates from 3 × 10^6^ 786 O cells stably transfected with control shRNA or shFKBP10 shRNA. Western blot analysis of LDHA protein levels in various fractions collected via gel filtration. **F**, **G** FKBP10 regulated the phosphorylation of LDHA-Y10 in 786 O and Caki1 cells. **H** FKBP10 regulated the phosphorylation of LDHA in a FGFR1-dependent manner. **I** Purified recombinant LDHA and FKBP10 proteins were incubated with or without active recombinant FGFR1 in the in vitro kinase assay. **J** The IHC scores of LDHA-Y10 phosphorylation were positively correlated with FKBP10. Statistical analyses were performed with a two-tailed unpaired Student’s *t* test (NS, *P* > 0.05, * *P* < 0.05, **P < 0.01, ***P < 0.001).

Western blot results showed that no change was observed in the protein expression of LDHA or LDHB after knocking down FKBP10 in various ccRCC cell lines (Fig. S4A). We detected the lactate dehydrogenase activity and lactate production in ccRCC cells. The results showed that after overexpressing or knocking down FKBP10, the activity of lactate dehydrogenase and the production of lactate were significantly enhanced or inhibited (Fig. 4A–D). The results indicated that the regulation of LDHA by FKBP10 does not affect its own protein expression but regulates the enzyme activity of LDHA.

Previous studies have demonstrated that LDHA enzyme activity is determined by the phosphorylation levels of LDHA on the Tyr10, which can enhance LDHA tetramerization, or phosphorylation at Tyr38 to increase the binding capacity of LDHA to NADH [28]. Additionally, Y10 phosphorylation of LDHA catalyzed by fibroblast growth factor receptor 1 (FGFR1) is commonly observed in a variety of human tumors. Gel filtration chromatography showed that LDHA tetramer abundance decreased significantly upon FKBP10 knockdown (Fig. 4E). This showed that FKBP10 regulated LDHA by enhancing the formation of active tetramers, which is also consistent with the physiological function of the FKBP10 protein that participates in the regulation of protein folding and the formation of the spatial structure.

Western blot analysis showed that FKBP10 overexpression or knockdown upregulated or downregulated the phosphorylation of the LDHA-Y10 site, respectively, but did not affect the expression of LDHA or LDHB (Fig. 4F, G). In addition, 786 O cells were treated with the FGFR1 inhibitor AZD4547 (500 nM, 24 h) and assessed by Western blot assays. The results showed that overexpressed FKBP10 could not enhance the phosphorylation of LDHA-Y10 with AZD4547 treatment (Fig. 4H). To further prove this point, in vitro kinase and Western blot assays were performed, and in the absence of FGFR1, FKBP10 lost its regulatory effect on LDHA-Y10 phosphorylation (Fig. 4I). In summary, the above results indicated that FKBP10 promoted LDHA-Y10 phosphorylation in a FGFR1-dependent manner.

We further confirmed that FKBP10 expression correlated with LDHA-Y10 phosphorylation by using tissue microarrays (R = 0.5744, *P* < 0.001, Fig. 4J, S4B, C). The above results showed that FKBP10 can affect the protein function of LDHA by regulating its phosphorylation.

### FKBP10 promotes ccRCC proliferation and metastasis in vivo in a manner dependent on its PPIase activity

**Fig. 5:**
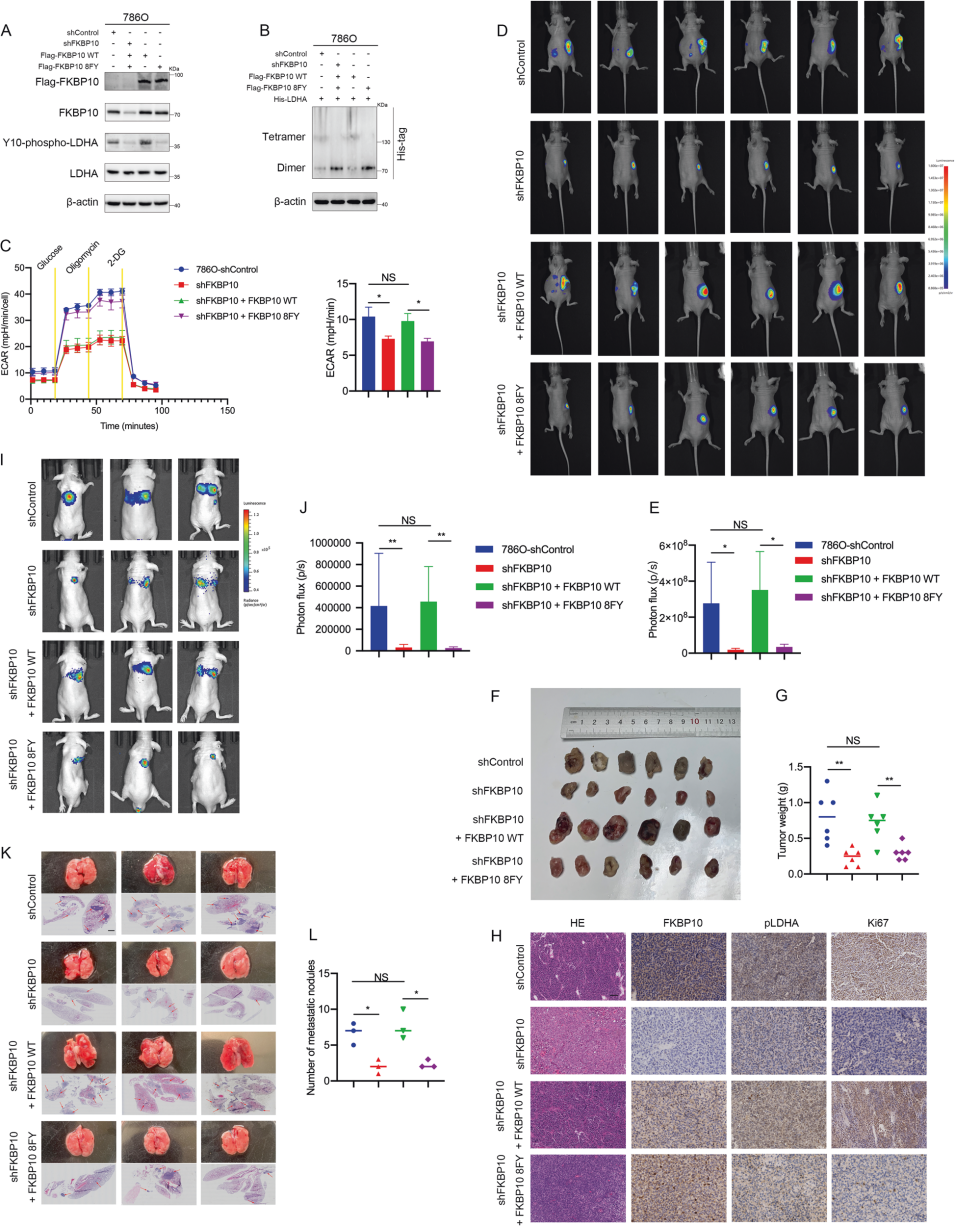
FKBP10 promotes ccRCC proliferation and metastasis in vivo depending on its PPlase activity. **A** FKBP10 regulated LDHA phosphorylation depending on its PPlase activity. **B** Nondenaturing protein electrophoresis analysis of His-tagged LDHA in 786 O cells transfected as indicated. **C** ECAR analysis of 786 O cells in (**A**). **D**, **E** Bioluminescence pictures of renal orthotopic tumors and quantitation of bioluminescence data are shown. **F**,**G** Gross appearance of the tumors and statistical analysis of tumor weights are shown. **H** Representative HE and IHC staining of tumors in the tested mice. Scale bar: 100 µm. **I**, **J** Bioluminescence pictures of lung metastases and quantitation of bioluminescence data are shown. **K**, **L** Gross appearance of the lung tissues analyzed by HE staining. Microscopic metastatic foci in the lung were counted. Arrows indicate pulmonary metastatic nodules. Scale bar: 100 µm. Statistical analyses were performed with a two-tailed unpaired Student’s *t* test (NS, *P* > 0.05, *P < 0.05, **P < 0.01, ***P < 0.001).

To further determine the mechanism underlying the pro-proliferation and pro-metastasis actions of FKBP10, we directly tested the relevance of its PPIase activity. By overexpressing a mutant FKBP10 (FKBP10-8FY, which contains mutations in all four PPIase domains including F79Y, F142Y, F191Y, F254Y, F303Y, F366Y, F417Y, and F478Y) [16] in shFKBP10 cells, Western blot assays showed that phosphorylation of LDHA could not be rescued in contrast to re-expression of FKBP10-WT (Fig. 5A). This shows that the regulation of FKBP10 on the phosphorylation level of LDHA depends on its PPIase function. Coimmunoprecipitation assays confirmed that the PPIase domains of FKBP10 were required for its binding with LDHA (Fig. S5A). Nondenaturing protein electrophoresis analysis showed that FKBP10 enhanced the formation of active tetramers of LDHA in a manner dependent on its PPIase function (Fig. 5B).

Then, we proved that the regulation of FKBP10 in LDHA activity and glycolysis in ccRCC was PPIase-dependent by performing Seahorse assays and detecting lactate production and LDH activity (Fig. 5C, S5B, I, J). Through cell function assays, including CCK8, plate clony formation, wound healing and Transwell assays, it was proven that FKBP10 promoted tumor proliferation, migration and invasion in vitro in a manner dependent on its PPIase function (Fig. S5C–H).

To strengthen this argument, we further established orthotopically implanted 786 O kidney tumors. Comparisons of in vivo imaging and tumor weights confirmed that renal orthotopic tumors after knocking down FKBP10 were significantly inhibited compared with the control group, and no metastases were detected at other sites. After re-expression of FKBP10-WT, the sizes and weights of renal tumors in situ were restored, but after re-expression of FKBP10-8FY, the sizes and weights of renal tumors in situ could not be restored and were still significantly inhibited compared with the control groups (Fig. 5D–G). The immunohistochemical results showed that FKBP10 could promote the phosphorylation of LDHA and enhance Ki67 expression, an endogenous cell proliferation marker, relying on the function of PPIase domains (Fig. 5H).

In our vein metastasis models, we observed that knocking down FKBP10 expression significantly reduced the sizes and numbers of lung metastases. Re-expression of FKBP10-WT restored the sizes and numbers of lung metastases, but not re-expression of FKBP10-8FY (Fig. 5I–L). The above results indicated that FKBP10 can promote the proliferation and metastasis of ccRCC in vivo depending on its PPIase function.

### FKBP10 promotes glycolysis and ccRCC progression via LDHA-Y10 phosphorylation

**Fig. 6:**
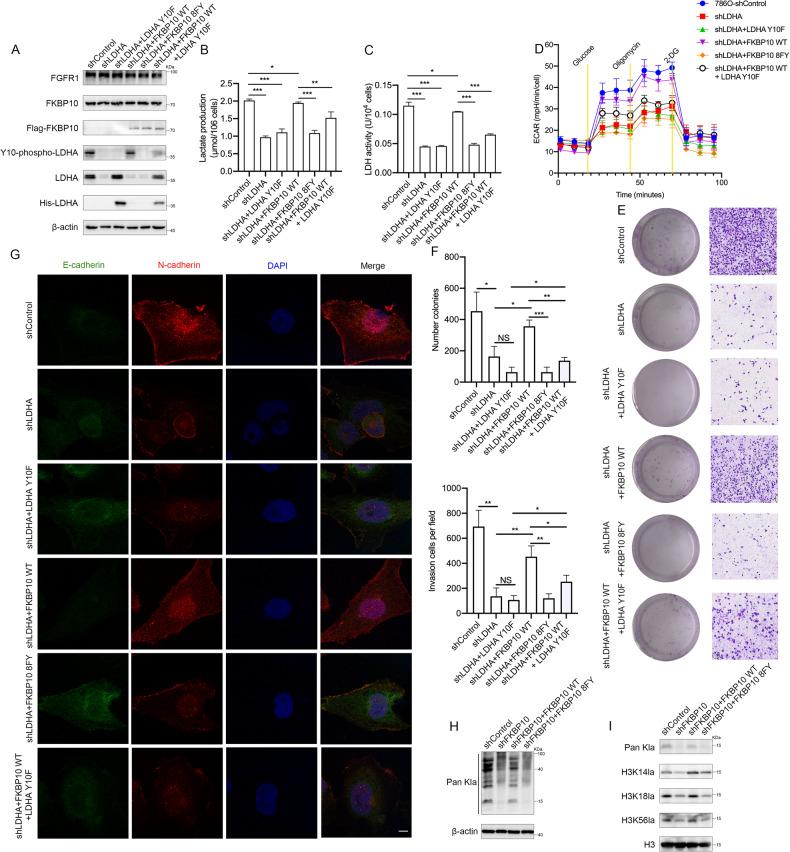
FKBP10 promotes glycolysis and ccRCC progression via LDHA-Y10 phosphorylation. **A** Western blot analysis of the LDHA-Y10 mutation. **B**, **C** Lactate production and LDH enzyme activity were examined in 786 O cells with the LDHA-Y10 mutation. **D** ECAR analysis of 786 O cells in (**A**). **E**, **F** Cell proliferation abilities were measured by colony formation assays. Cell invasion abilities were measured by Transwell assays. Scale bar: 100 µm. Data are presented as the means ± SD, and were independently replicated three times. **G** Representative images of immunofluorescence staining for E-cadherin (green) and N-cadherin (red) (magnification ×100). DAPI (blue) was applied for nuclear staining. Scale bar: 10 µm. **H** Western blot analysis of Pan Kla in 786 O cells as indicated. **I** Western blot analysis of histone Pan Kla levels, H3K14la, H3K18la and H3K56la in 786 O cells as indicated. Statistical analyses were performed with a two-tailed unpaired Student’s *t* test (* *P* < 0.05, **P < 0.01, ***P < 0.001).

Previous experiments have proven that the regulation of FKBP10 on LDHA is the regulation of its phosphorylation, rather than the regulation of its own expression. To explore whether FKBP10 depended on LDHA-Y10 phosphorylation to promote glycolysis and ccRCC progression, we established Y10F mutant LDHA cell lines and assessed them by Western blotting (Fig. 6A). Lactate production and LDH activity were detected, and the results showed that the regulation of FKBP10 in glycolysis in ccRCC was LDHA-Y10 phosphorylation dependent (Fig. 6B, C). A Seahorse assay was performed to confirm that FKBP10 cannot enhance the glycolysis in 786 O cells with the LDHA-Y10 mutation (Fig. 6D). The results of cell function experiments and immunofluorescence proved that FKBP10 lost the ability to promote ccRCC proliferation and metastasis with the LDHA Y10 mutation (Fig. 6E–G).

Since LDHA’s primary function is to generate lactate, and lactate as the final metabolite of the glycolytic pathway, it plays an important role in tumor progression and microenvironment regulation. The latest studies have showed that lactate directly modifies nuclear histones, a process that has been identified as lysine lactylation (Klac) [29]. Figure 6H, I shows that FKBP10 could regulate the lactylation level of ccRCC, which relied on its own PPIase domains. However, how lactate regulates the progression of ccRCC needs further exploration.

### HIFα negatively regulates FKBP10 expression at the transcriptional level

**Fig. 7:**
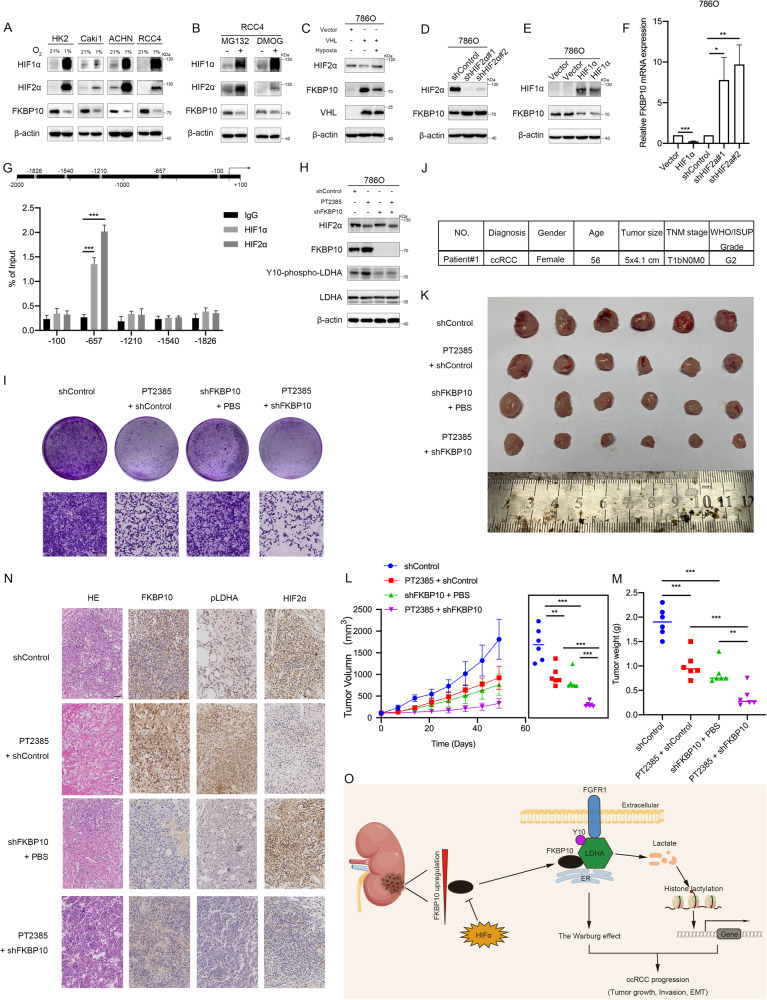
HIFα negatively regulates FKBP10, and inhibition of FKBP10 enhances the antitumor effects of PT2385. **A** HK2, Caki1, ACHN and RCC4 cells were exposed to 21% O_2_ or 1% O_2_ for 24 h. **B** RCC4 cells were treated with DMOG (1 mM) or MG132 (10 μM) for 24 h. **C** Western blot analysis of the indicated proteins in 786 O cells overexpressing VHL. **D**, **E** Western blot analysis of the indicated proteins in 786 O cells with HIF2α knockdown or HIF1α overexpression. **F** The mRNA levels of *FKBP10* in 786 O cells with HIF2α knockdown or HIF1α overexpression were examined by qRT‒PCR. **G** HIFα binding sites (HBSs) were obtained from the JADPAR promoter database within the human *FKBP10* promoter region. CUT&RUN assays and qRT‒PCR were performed with primers containing candidate HBS sites in the *FKBP10* promoter. **H** Western blot analysis of the indicated proteins in 786O-NC or 786O-shFKBP10 cells treated with PT2385 (20 µM) or PBS. **I** Cell proliferation abilities were measured by colony formation assays. Cell invasion abilities were measured by Transwell assays. Scale bar: 10 µm. Data are presented as the means ± SD and were independently replicated three times. **J** Clinical information for patient #1 from the patient’s cancer tissues. **K** Tumors from the shControl, PT2385+shControl, shFKBP10+PBS and PT2385+shFKBP10 groups are shown. **L**, **M** Tumor growth curves and tumor weight of each group. **N** Representative HE and IHC staining of the indicated proteins in tumors. **O** Schematic diagram to illustrating that FKBP10 mediated by HIFα promotes ccRCC progression via LDHA phosphorylation. Statistical analyses were performed with a two-tailed unpaired Student’s *t* test (* *P* < 0.05, **P < 0.01, ***P < 0.001).

Previous bioinformatic analysis revealed that FKBP10 may exert important functions in the hypoxic pathway of ccRCC. To explore the role of FKBP10 in the activation of the hypoxia pathway, HK2 cells and several ccRCC cell lines (Caki1, AHCN and RCC4) were treated with the hypoxia-mimicking agent CoCl_2_ (200 µM, 24 h) or exposed to hypoxia for 24 h (1% O_2_). Western blot analyses showed that the protein expression of FKBP10 was significantly reduced, while HIF1α and HIF2α were both upregulated (Fig. 7A, S6A). The ccRCC cell line RCC4 was treated with the proteasome inhibitor MG132 (10 µM) or the proline hydroxylase inhibitor DMOG (1 mM) for 24 h, both of which stabilized HIFα. Figure 7B illustrates that both DMOG and MG132 inhibited the expression of FKBP10, accompanied by elevated HIF1α and HIF2α. Furthermore, overexpressed VHL could promote the degradation of HIFα and significantly increase the expression of FKBP10 (Fig. 7C). Overexpressing VHL during treatment with CoCl_2_ restored the expression of HIF2α and inhibited the expression of FKBP10. The above results indicated that FKBP10 was inhibited by the hypoxia pathway.

Our next step was to knockdown the expression of HIF1α and HIF2α respectively, and found that FKBP10 expression was significantly upregulated (Fig. 7D, S6B–D). Given that in ccRCC cell lines, HIF2α was generally highly expressed while HIF1α had low or no expression, we overexpressed HIF1α in 786 O and Caki1 cells, and found that FKBP10 expression was suppressed at both the mRNA and protein levels (Fig. 7E, F, S6E). Therefore, we concluded that HIFα can inhibit FKBP10 expression at the transcriptional level. To confirm whether HIFα directly regulates FKBP10, several common transcriptional repressors regulated by HIFα including DEC1, REST, ZEB1 and ZEB2, were knocked down, and the mRNA expression of *FKBP10* was unchanged (Fig. S6F), which indicated that HIFα may directly inhibit the expression of FKBP10. To further demonstrate the binding of HIFα to the *FKBP10* promoter, the JASPAR database (http://jaspar.genereg.net/) was searched for HIF-binding sites (HBSs) within the FKBP10 promoter region. Several candidate sites were predicted, and a CUT&RUN assay was performed, which showed that HIF1α and HIF2α could directly bind with the *FKBP10* promoter sequence 657 bp upstream of the transcription start site (Fig. 7G).

### Inhibition of FKBP10 enhances the antitumor effects of the HIF2α inhibitor PT2385

Based on our previous experiments, we found that knocking down the expression of HIFα can upregulate FKBP10. Thus, we speculated that inhibition of FKBP10 can enhance the antitumor effect of HIF2α inhibitors. Western blot assays showed that after treatment with PT2385 (20 µM) for 24 h, HIF2α expression decreased along with FKBP10 expression, and the phosphorylation of LDHA was upregulated in 786 O cells. Knocking down FKBP10 in PT2385-treated cells significantly inbibited the phosphorylation level of LDHA (Fig. 7H). Cell function assays showed that inhibition of FKBP10 while using PT2385 had a more significant inhibitory effect on the proliferation and invasion abilities of the 786 O cells (Fig. 7I, S6G, H).

To further verify this, a ccRCC patient-derived xenograft (PDX) model was constructed (Fig. S6I). The patient was a 56-year-old female who was diagnosed with ccRCC in 2022. Detailed clinical information is provided in Fig. 7J. The results showed that simultaneous application of PT2385 and shFKBP10-AAV inhibited the tumor growth (Fig. 7K). The final tumor volumes and weights were significantly suppressed compared with those of PT2385 or shFKBP10-AAV using alone (Fig. 7L, M). Immunohistochemical assays demonstrated the inhibitory effects of PT2385 and shFKBP10-AAV on HIF2α and FKBP10, respectively (Fig. 7N). Taken together, the above results indicated that inhibition of FKBP10 could enhance the antitumor effect of the HIF2α inhibitor PT2385.

## Discussion

At present, the difficulty in the treatment of ccRCC mainly lies in the lack of ideal therapeutic targets for patients with advanced metastatic tumors or recurrence after surgery. Widely used immunosuppressants, tyrosine kinase inhibitors and the novel HIF2α inhibitors generally have initial insensitivity or long-term drug resistance. Therefore, it is urgent to further understand and explore new therapeutic targets and medication methods with high efficiency based on the prominent genetic features and metabolic signatures of ccRCC.

HIF2α behaves as a pro-oncogenic effector in the regulation of erythropoietin, tumor angiogenesis, enhancement of pentose phosphate pathways, resistance to oxidative damage, endoplasmic reticulum stress, and tumor metastasis [30]. Meanwhile, FKBP10 negatively regulated by HIF2α is identified as a novel target of ccRCC with a cancer-promoting role, which seems a rather paradoxical finding. But his may also explain why many ccRCC patients treated by HIF2α inhibitors achieved poor therapeutic efficacy and disease progression. So, treating FKBP10 low-expressing patients with HIF2α inhibitors or combined application of HIF2α and FKBP10 inhibitors may lead to new therapeutic strategies to prevent ccRCC progression.

Screening of small-molecule compounds targeting FKBP10 is a goal of further research in our next study to achieve clinical application value. Our research has proven that FKBP10 can promote the proliferation and metastasis of renal clear cell carcinoma, and it depends on its own PPIase functional domain. The PPIase functional domain was first discovered in the FKBP12 protein. Based on this domain, FKBP12 can be combined with the immunosuppressive drug tacrolimus (FK506), resulting in the inhibition of PPIase function [31]. Therefore, can FK506 combine with FKBP10 and exert an inhibitory function? Studies have shown that FK506 can only reduce the activity of FKBP10 by 25% and confirmed that only the first PPIase domain in FKBP10 can be inhibited by FK506 [32]. After we treated 786 O cells with FK506, we found that the phosphorylation level of LDHA did not change significantly, and the production of lactate and the activity of lactate dehydrogenase were not altered, which indicated that FK506 had no effect on the function of FKBP10 in regulating LDHA. Most likely, FKBP10 has four PPIase functional domains, and when one domain is inhibited, the other three domains play a compensatory role. Above all, when designing small molecule compounds or drugs targeting FKBP10, it is necessary to consider that all PPIase functional domains can be targeted at the same time to achieve the inhibition of FKBP10 function.

In conclusion, our study confirmed that FKBP10 can be a new target for the treatment of clear cell renal cell carcinoma, and inhibiting the expression of FKBP10 can synergistically enhance the antitumor effect of the HIF2α inhibitor PT2385, which opens up new ideas for the clinical treatment of patients with advanced clear cell renal cell carcinoma.

### Reporting summary

Further information on research design is available in the Nature Research Reporting Summary linked to this article.

### Supplementary information

Reporting Summary

Supplementary

Original Data File

## Data Availability

The data generated in this study are available within the article and its supplementary files.
