# Supplementary material for: FKBP10 promotes clear cell renal cell carcinoma progression and regulates sensitivity to the HIF2α blockade by facilitating LDHA phosphorylation
Source: Cell Death Dis. 2024 Jan 17;15(1):64. doi: 10.1038/s41419-024-06450-x (PMC10794466; doi:10.1038/s41419-024-06450-x)
Supplement: Supplementary file 2 — Supplementary [file 41419_2024_6450_MOESM2_ESM.pdf]

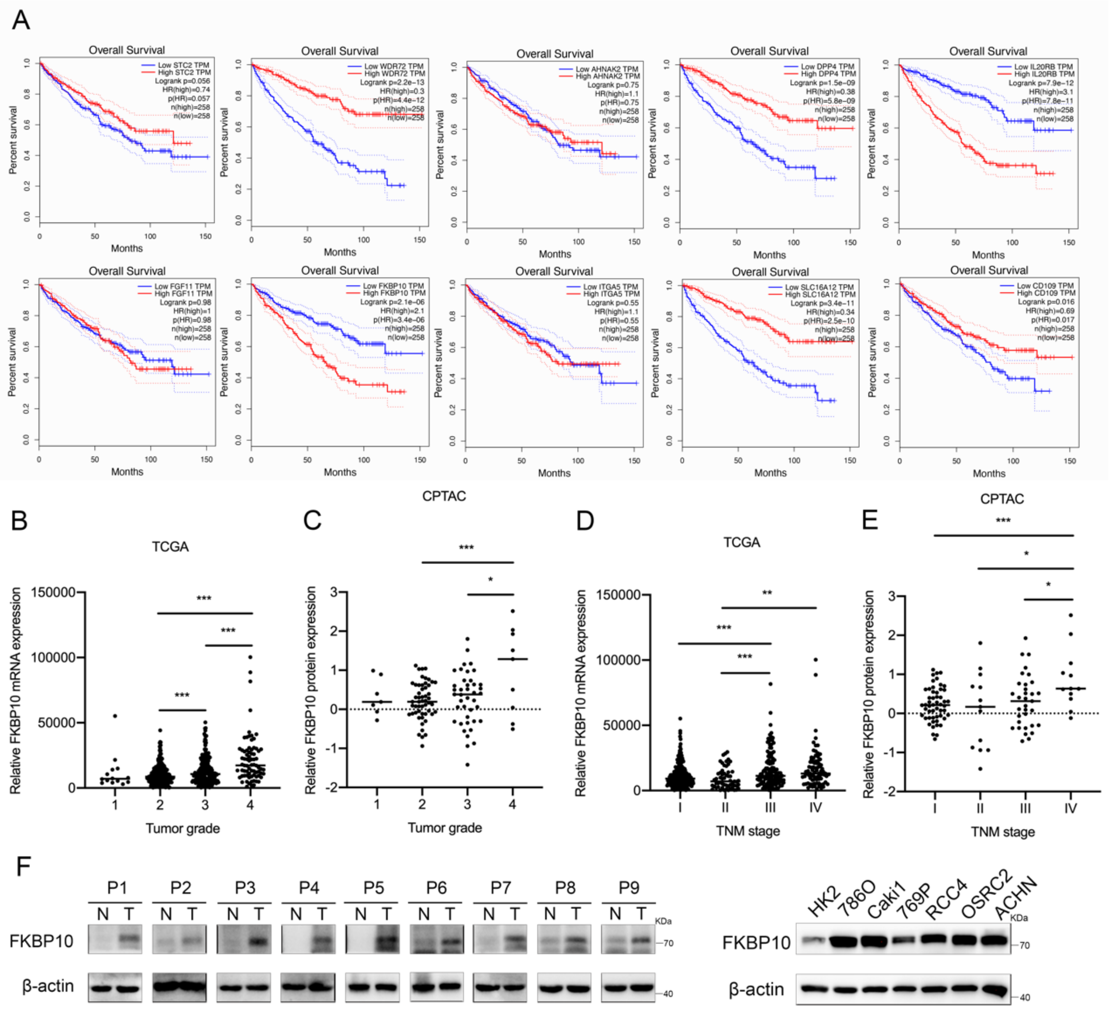

Supplementary Fig. 1 (A) Survival analyses (http://gepia.cancer-pku.cn).(B-C) (A)Analyses of FKBP10 expression in four different pathological grades of ccRCC based on the TCGA-KIRC or CPTAC-KIRC datasets. (D-E) Analyses of FKBP10 expression in four different clinical stages of ccRCC based on the TCGA-KIRC or CPTAC-KIRC datasets. (F) Expression of FKBP10 in 9 paired ccRCC samples and in human renal tubular epithelial cell line HK2 and various ccRCC cell lines. Statistical analyses were performed with a two-tailed unpaired Student's t test (NS, $P > 0.05$, * $P < 0.05$, **$P < 0.01$, ***$P < 0.001$).

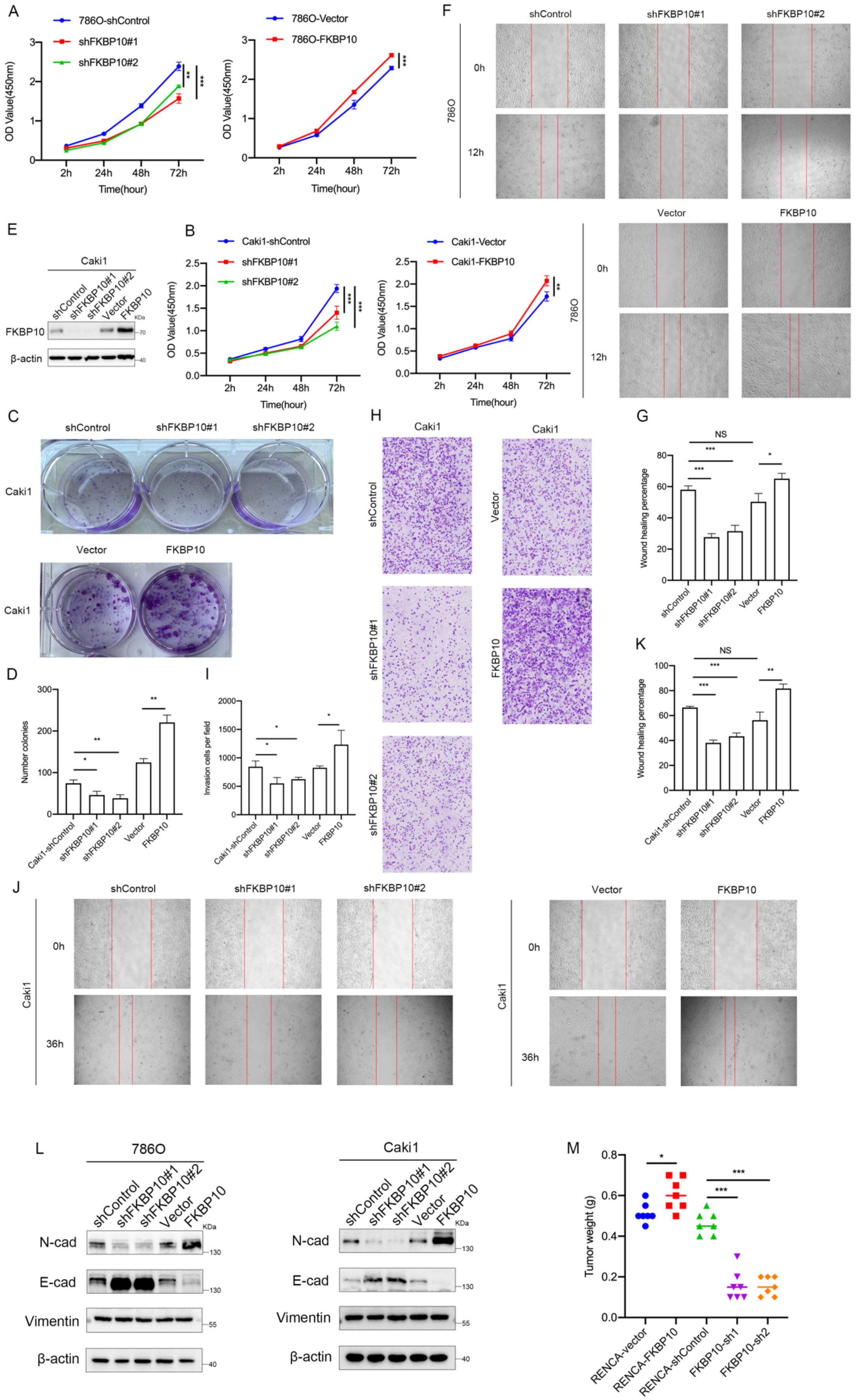

A
786O-shControl
shFKBP10#1
shFKBP10#2
OD Value(450nm)
Time(hour)
786O-Vector
786O-FKBP10
F
shControl
shFKBP10#1
shFKBP10#2
0h
12h
786O
E
Caki1
FKBP10
β-actin
B
Caki1-shControl
Caki1-Vector
Caki1-FKBP10
Vector
FKBP10
C
D
Number colonies
I
Invasion cells per field
H
G
Wound healing percentage
K
J
36h
L
N-cad
E-cad
Vimentin
M
Tumor weight (g)
RENCA-vector
RENCA-FKBP10
RENCA-shControl
FKBP10-sh1
FKBP10-sh2

Supplementary Fig. 2 (A-D) Cell proliferation as determined by CCK-8 and colony formation assays. (E) The overexpression or knockdown of FKBP10 in Caki1 cells were measured by Western blot. (F-K) Cell migration and invasion as determined by wound healing and Transwell assays. Red lines denote the margins of the wound. Scale bar: 100μm. The results are presented as mean ± SD of three independent experiments. (L) Western blot analyses of indicated proteins in 786O or Caki1 cells with FKBP10 overexpressed or knockdown. (M) Statistical analysis of tumor weights. Statistical analyses were performed with a two-tailed unpaired Student's t test (NS, $P > 0.05$, * $P < 0.05$, **$P < 0.01$, ***$P < 0.001$).

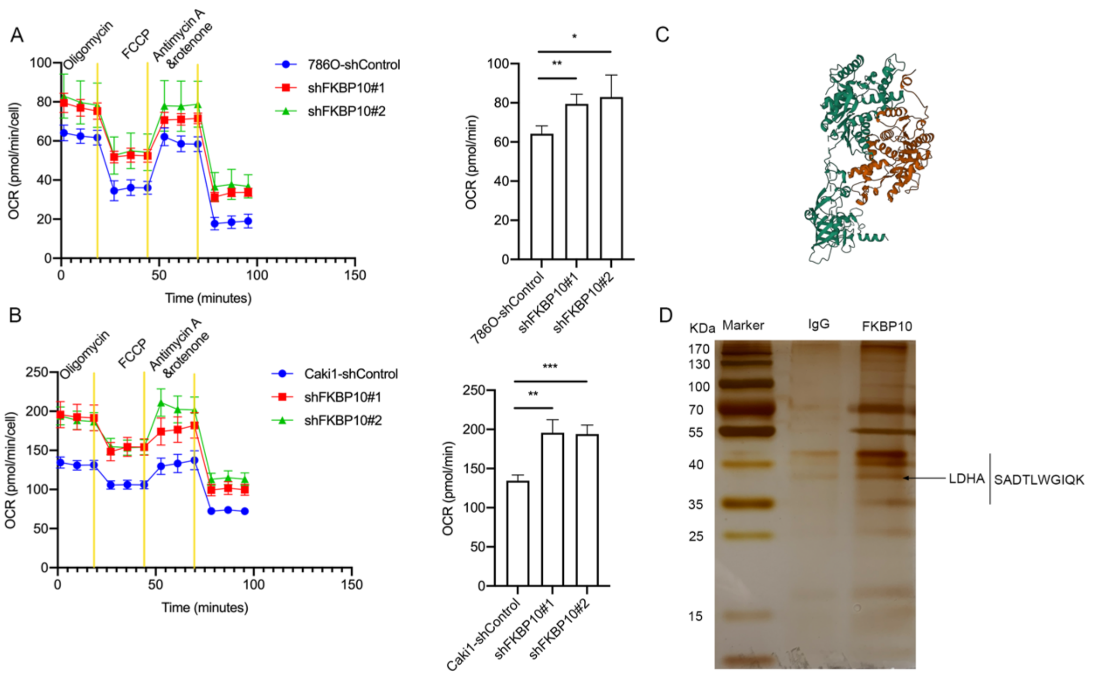

Supplementary Fig. 3 (A-B) Oxygen consumption rate (OCR) of 786O and Caki1 cells with FKBP10 knockdown. (C) Modeling of FKBP10 binding with LDHA. FKBP10 is colored green, and LDHA is colored red. (D) Proteins immunoprecipitated by FKBP10 or mouse IgG antibody were separated by SDS-PAGE and silver-stained. Arrowed band, which specificly bound with FKBP10, was cut out and identified by LS-MS/MS analysis. Statistical analyses were performed with a two-tailed unpaired Student's t test (* $P < 0.05$, **$P < 0.01$, ***$P < 0.001$).

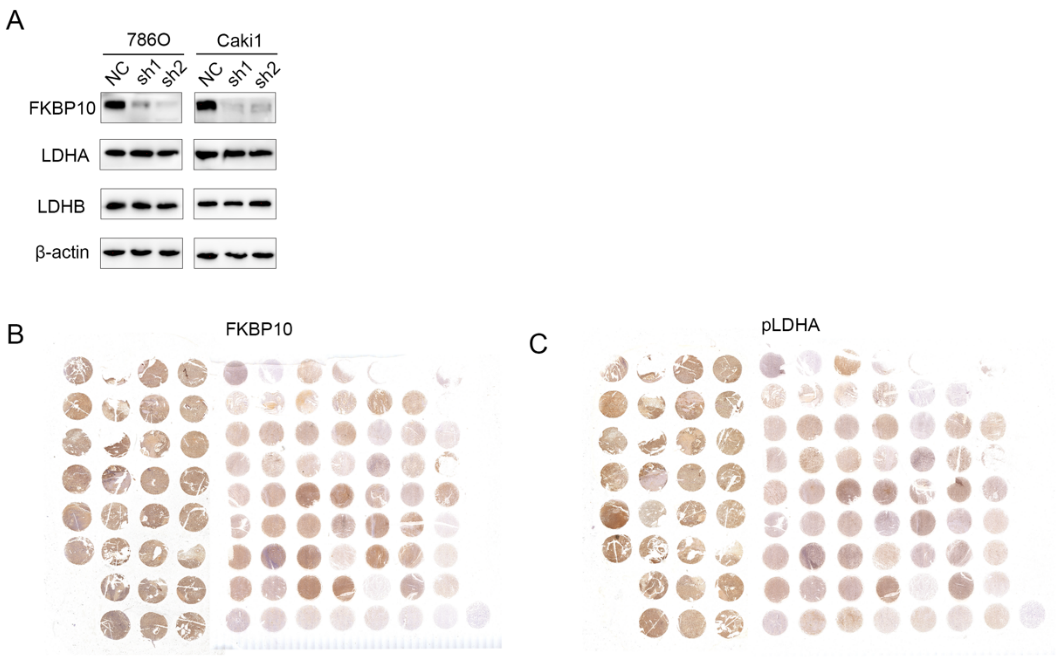

Supplement Fig. 4 (A) Western blot analysis of indicated proteins in 786O or Caki1 cells with FKBP10 knockdown. (B-C) IHC staining images of human ccRCC tissue microarray elucidated by FKBP10 or pLDHA antibody.

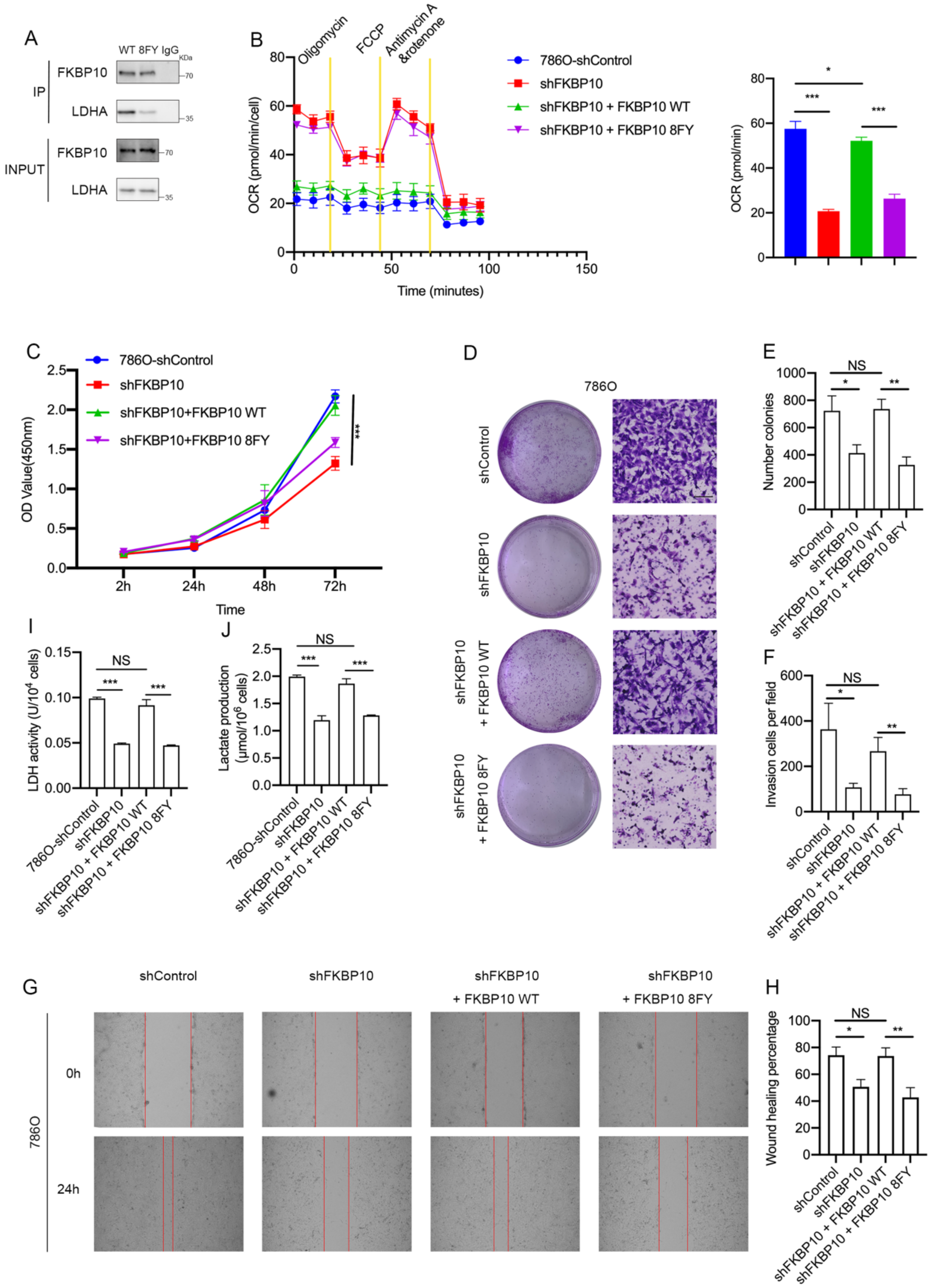

Supplement Fig. 5 (A) Co-IP analysis of the interaction between FKBP10 and LDHA in HEK293 cells with FKBP10-8FY overexpressed. (B) OCR analysis of 786O cells transfected with the indicated plasmids. (C-F) Cell proliferation as determined by CCK-8 assays and colony formation assays. Cell invasion as determined by Transwell assays. Scale bar: 100μm. (G-H) Cell migration as determined by wound healing assays. Red lines denote the margins of the wound. (I-J) Lactate production levels and LDH enzyme activity in 786O or Caki1 cells transfected with the indicated plasmids were examined.

The results are presented as mean ± SD of three independent experiments. Statistical analyses were performed with a two-tailed unpaired Student's t test (NS, $P > 0.05$, * $P < 0.05$, ** $P < 0.01$, *** $P < 0.001$).

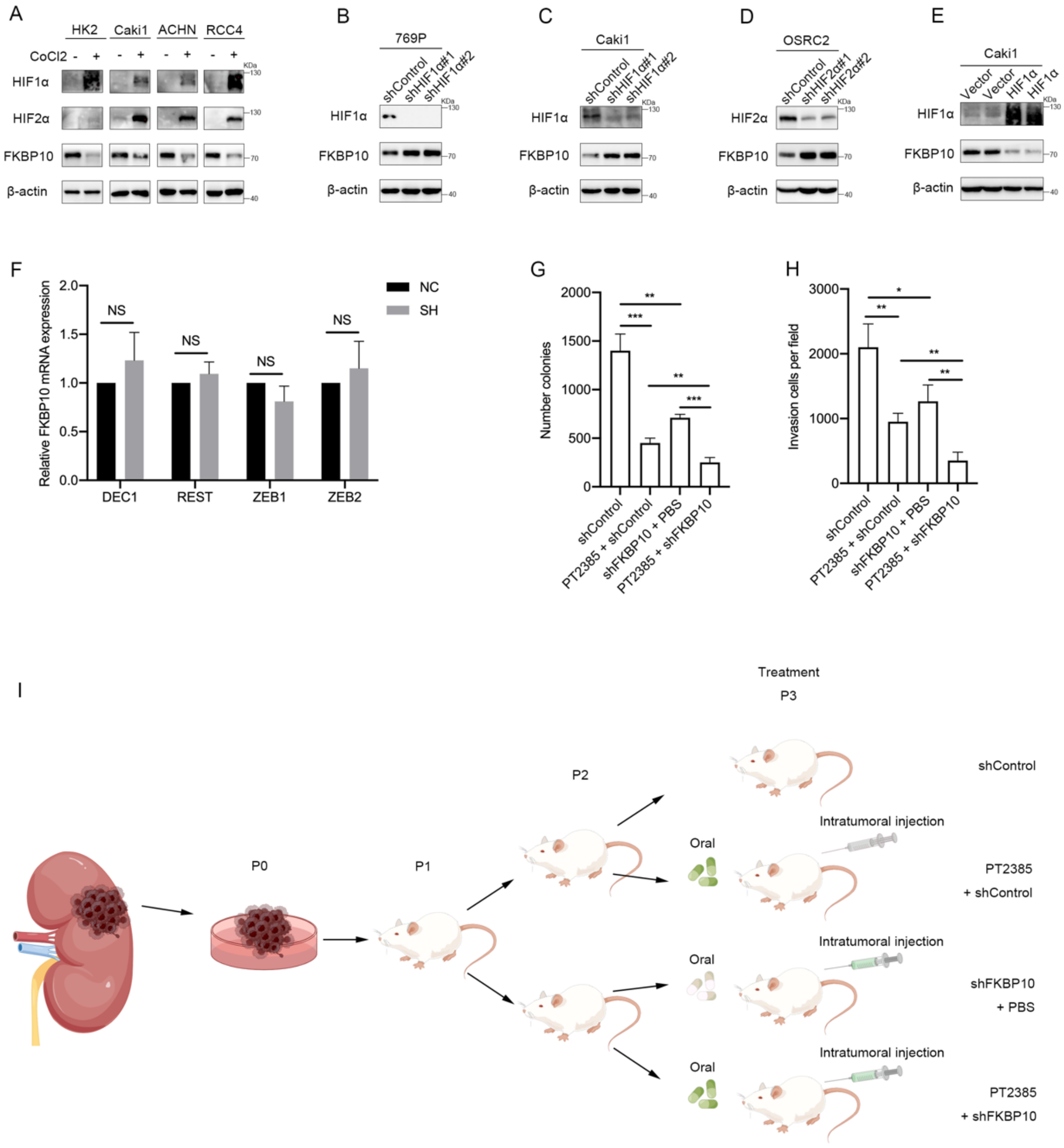

Supplementary Fig. 6 (A) HK2, Caki1, ACHN and RCC4 cells were treated with $CoCl_2$ (200μM) for 24h, followed by Western blot analysis. (B-C) Western blot analyses of indicated proteins in 769P or Caki1 cells with HIF1α knockdown. (D) Western blot analysis of indicated proteins in OSRC2 cells with HIF2α knockdown. (E) Western blot analysis of indicated proteins in Caki1 cells with HIF1α overexpressed. (F) The mRNA levels of *FKBP10* in 786O cells with DEC1, REST, ZEB1 or ZEB2 knockdown. (G-H) Lactate production levels and LDH enzyme activity in 786O-NC or 786O-shFKBP10 cells treated with PT2385 (20μM) or PBS. The results are presented as mean ± SD of three independent experiments. (I) Schematic diagram of PDX model construction of ccRCC. Statistical analyses were performed with a two-tailed unpaired Student's t test (NS, P > 0.05, * P < 0.05, **P < 0.01, ***P < 0.001).

Supplementary Table 1. Clinicopathological characteristics in 79 ccRCC patients with different FKBP10 expression level

Data were shown as n (%).

Parameter	Total	FKBP10 expression		P value
Age (year)		High	Low	0.25
<60	42 (53.2)	28	14	
⩾60	37 (46.8)	29	8	
Gender				0.44
Female	23 (29.1)	18	5	
Male	56 (70.9)	39	17	
Tumor stage				0.09
Ⅰ/Ⅱ	60 (75.9)	44	16	
Ⅲ/Ⅳ	19 (24.1)	10	9	
Grade				0.017*
1	23(29.1)	11	12	
2/3	56 (70.9)	43	13	
Size (Longest Diameter, cm)				0.029*
<7	62 (78.5)	42	20	
⩾7	11 (13.9)	11	0	
Metastasis				0.0008*
M0	56 (70.89)	35	21	
M1	20 (25.3)	20	0	

*P value < 0.05

Supplementary Table 2. Univariate and multivariate Cox regression analyses of different parameters on disease-free survival

Parameter	Univariate Analysis		Multivariate Analysis	
	HR (95%CI)	P value	HR (95%CI)	P value
Age (year)	1.001 (0.968,1.035)	0.94	-	-
Gender	1.13 (0.445, 2.868)	0.79	-	-
Stage	1.239 (0.845, 1.817)	0.27	-	-
Grade	2.227 (1.168, 4.147)	0.015*	1.651 (0.757, 3.603)	0.208
Metastasis	10.117 (4.318, 23.708)	<0.001*	4.093 (1.452, 11.542)	0.008*
Size	1.283 (1.13, 1.458)	<0.001*	1.055 (0.905, 1.231)	0.495
FKBP10 expression	1.443 (1.242, 1.675)	<0.001*	1.198 (0.999, 1.436)	0.051

HR: hazard ratio; CI: confidence interval

Supplementary Table 3. Antibodies used in this study.

Name (Species)	Catalog number	Company	Application
β-actin (Rabbit)	AF7018	Affinity	WB
FKBP10 (Rabbit)	12172-1-AP	Proteintech	WB; IHC
FKBP10 (Mouse)	D-4	SANTA CRUZ	IP; IF
N-cadherin (Rabbit)	22018-1-AP	Proteintech	WB; IF
E-cadherin (Mouse)	BF0219	Affinity	WB; IF
LDHA (Rabbit)	19987-1-AP	Proteintech	IP
LDHA (Rabbit)	DF6280	Affinity	WB; IF
pLDHA (Y10)	AF7172	Affinity	IHC; WB; IF
LDHB (Rabbit)	ab53292	Abcam	WB
His-Tag (Rabbit)	12698	CST	WB; IP
DYKDDDDK Tag (Mouse)	8146	CST	WB; IP
FGFR1 (Rabbit)	Ab76464	Abcam	WB
Ki67 (Rabbit)	AF0198	Affinity	IHC
pFGFR1(Y653+Y654) (Rabbit)	AF8210	Affinity	WB
L-Lactyl Lysine (Rabbit)	PTM-1401RM	PTM BIO	WB
H3K14la (Rabbit)	PTM-1414RM	PTM BIO	WB
H3K18la (Rabbit)	PTM-1427RM	PTM BIO	WB
H3K56la (Rabbit)	PTM1421RM	PTM BIO	WB
Histone H3 (Rabbit)	PTM-6613	PTM BIO	WB
HIF1α(Rabbit)	36169	CST	WB; ChIP
HIF2α(Rabbit)	87179	CST	WB; IHC; ChIP
VHL (Rabbit)	68547	CST	WB

Supplementary Table 4. FKBP10 partners were identified using IP-MS (Top 10)

Protein IDs	Gene symbol	Coverage	Unique peptides	Abundances (Grouped)
P67809	YBX1	60	7	77384138.41
F8W1R7	MYL6	58	6	67947471.44
P00338	LDHA	51	10	112933586.8
P23396	RPS3	50	10	55631766.69
P63173	RPL38	49	4	7708917.875
P22626	HNRNPA2B1	48	14	95398792.38
P61247	RPS3A	48	13	158444820
E5RI99	RPL30	48	4	10053958.19
O14950	MYL12B	45	1	12909184

Supplementary Table 5. Interfering sequences used in knockdown experiments.

Name	Sequence (5'-3')
shFKBP10-1	CCATCCCTAAACCACTTCCTT
shFKBP10-2	CCACTACAATGGCTCCTTGAT
shLDHA	GATCTGTGATTAAAGCAGTAA
shHIF1α-1	CCGCTGGAGACACAATCATAT
shHIF1α-2	CCAGTTATGATTGTGAAGTTA
shHIF2α-1	CAGTACCCAGACGGATTTCAA
shHIF2α-2	GCGCAAATGTACCCAATGATA
shDEC1	CCTACGAAGATTACTTAAGAA
shREST	CGAGTCTACAAGTGTATCATT
shZEB1	CCTCTCTGAAAGAACACATTA
shZEB2	CCCACCATGAATAGTAATTTA
shControl	CAACAAGATGAAGAGCACCAA

Supplementary Table 6. Sequence of the DNA primers for qRT-PCR

基因	Forward	Reverse
FKBP10	AGAACCACATCGAAGTAGAGGG	AGAACCACATCGAAGTAGAGGG
GAPDH	GGAGCGAGATCCCTCCAAAAT	GGCTGTTGTCATACTTCTCATGG
HIF1α	GAACGTCGAAAAGAAAAGTCTCG	CCTTATCAAGATGCGAACTCACA
HIF2α	CGGAGGTGTTCTATGAGCTGG	AGCTTGTGTGTTCGCAGGAA
DEC1	ATGACAATGAATGTTCTGGAGGC	CATCATGTGTAACACGGCAAGA
REST	GCCGCACCTCAGCTTATTATG	CCGGCATCAGTTCTGCCAT
ZEB1	GATGATGAATGCGAGTCAGATGC	ACAGCAGTGTCTTGTTGTTGT
ZEB2	CAAGAGGCGCAAACAAGCC	GGTTGGCAATACCGTCATCC
Promoter-100	AGGCCAGCTCAGTCTTCCCA	CTTCCTGTCCTCTTTCCCTA
Promoter-657	ATACGAGAAGGAAGCCCT	AAGGTGTGCAGGTGACAG
Promoter-1210	TCTCTCCCTCGTACTGCT	GGCGCAGTACGAGAAGTA
Promoter-1540	TCCTTCGACCGATCTGTG	CCTACCAGTACCTGCACT
Promoter-1826	CTCTAGGTCCGTGAGGGACT	ACCGCCAAGTATCTCAACTA
