# Supplementary material for: FKBP10 promotes clear cell renal cell carcinoma progression and regulates sensitivity to the HIF2α blockade by facilitating LDHA phosphorylation
Source: Cell Death Dis. 2024 Jan 17;15(1):64. doi: 10.1038/s41419-024-06450-x (PMC10794466; doi:10.1038/s41419-024-06450-x)

Fig. 2A

Actin

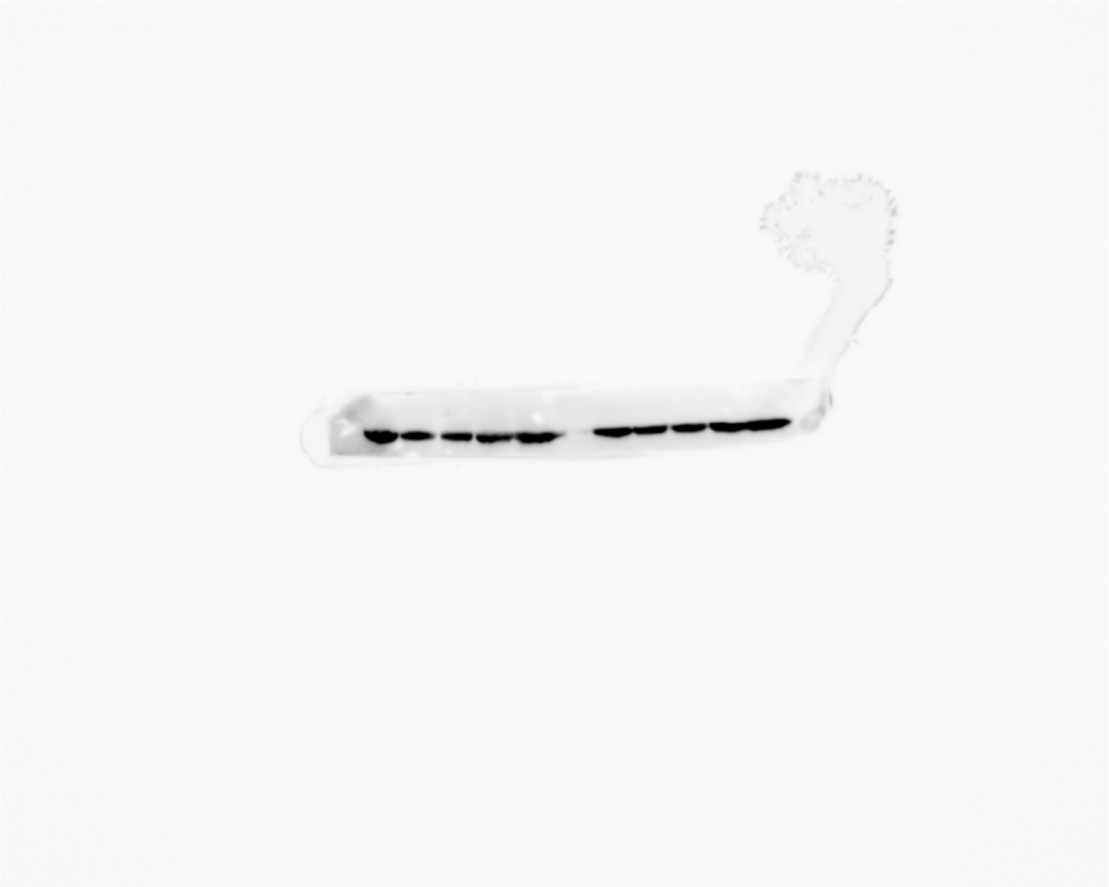

FKBP10

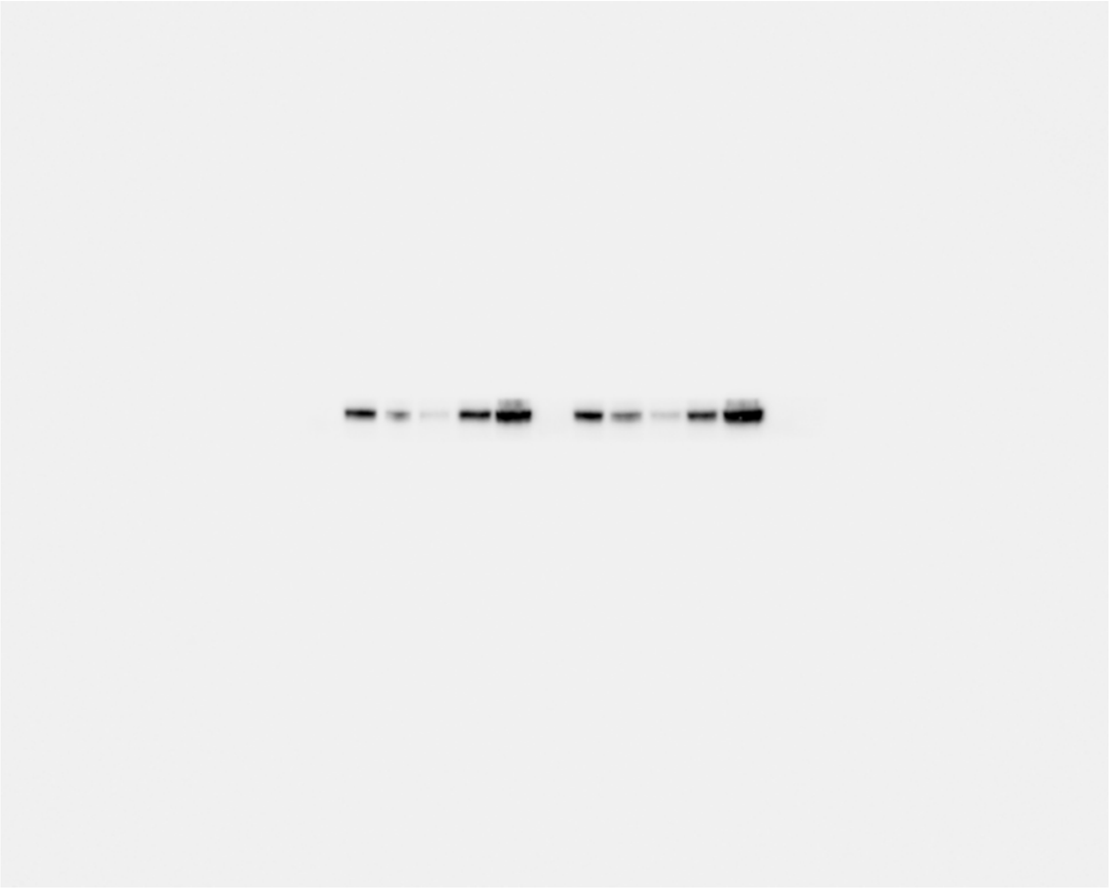

Fig. 3C
FKBP10-786O

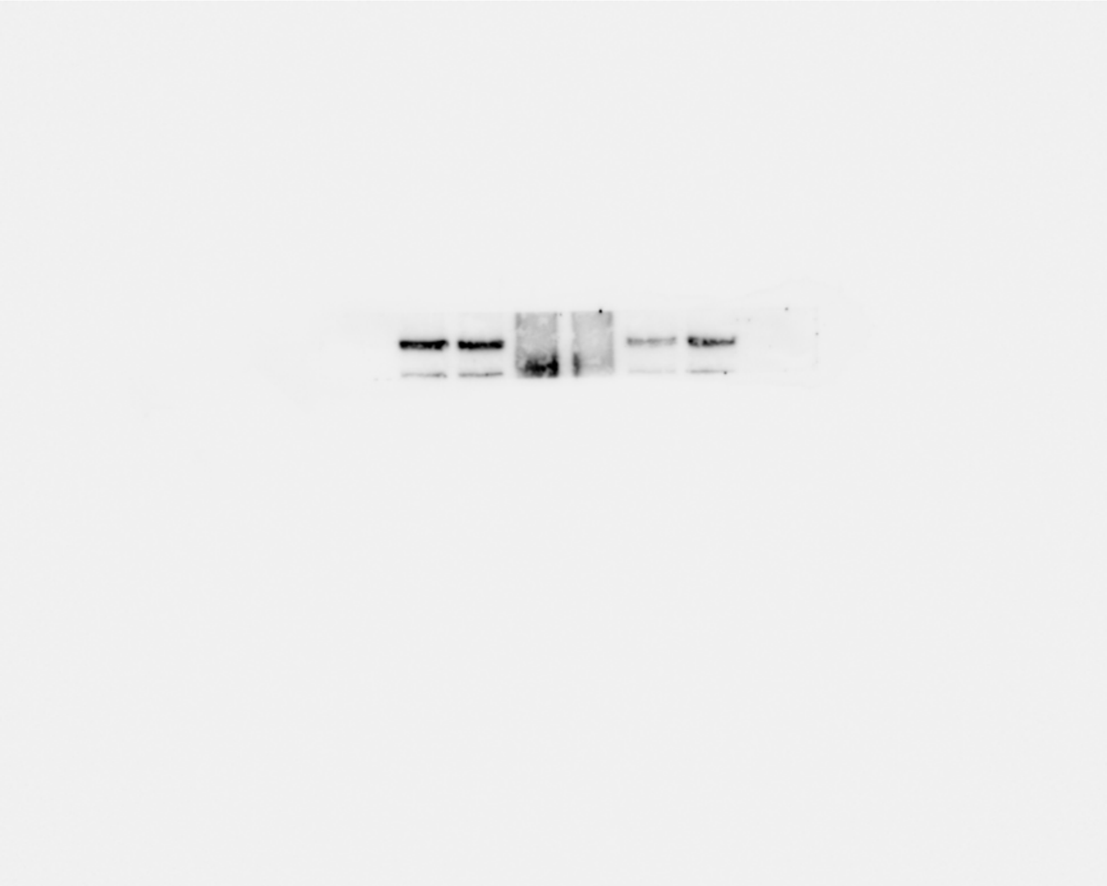

LDHA-786O

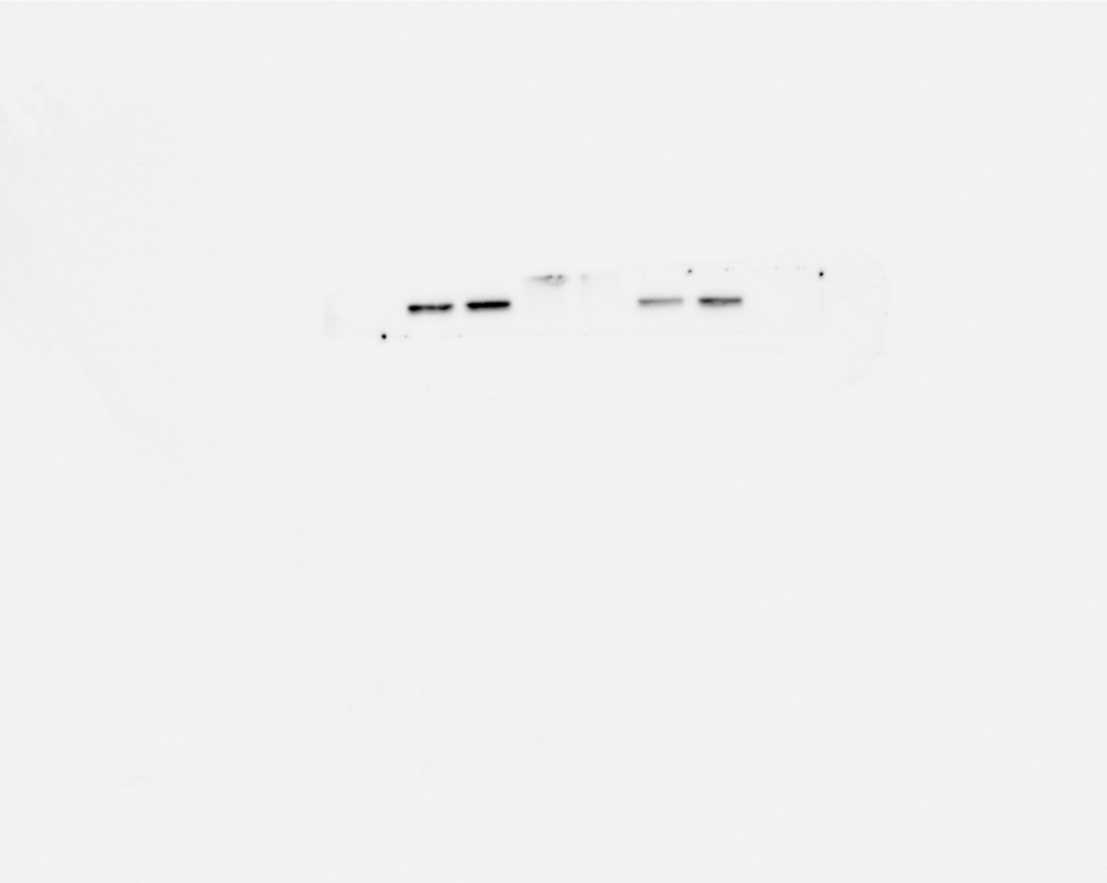

FKBP10-Caki1

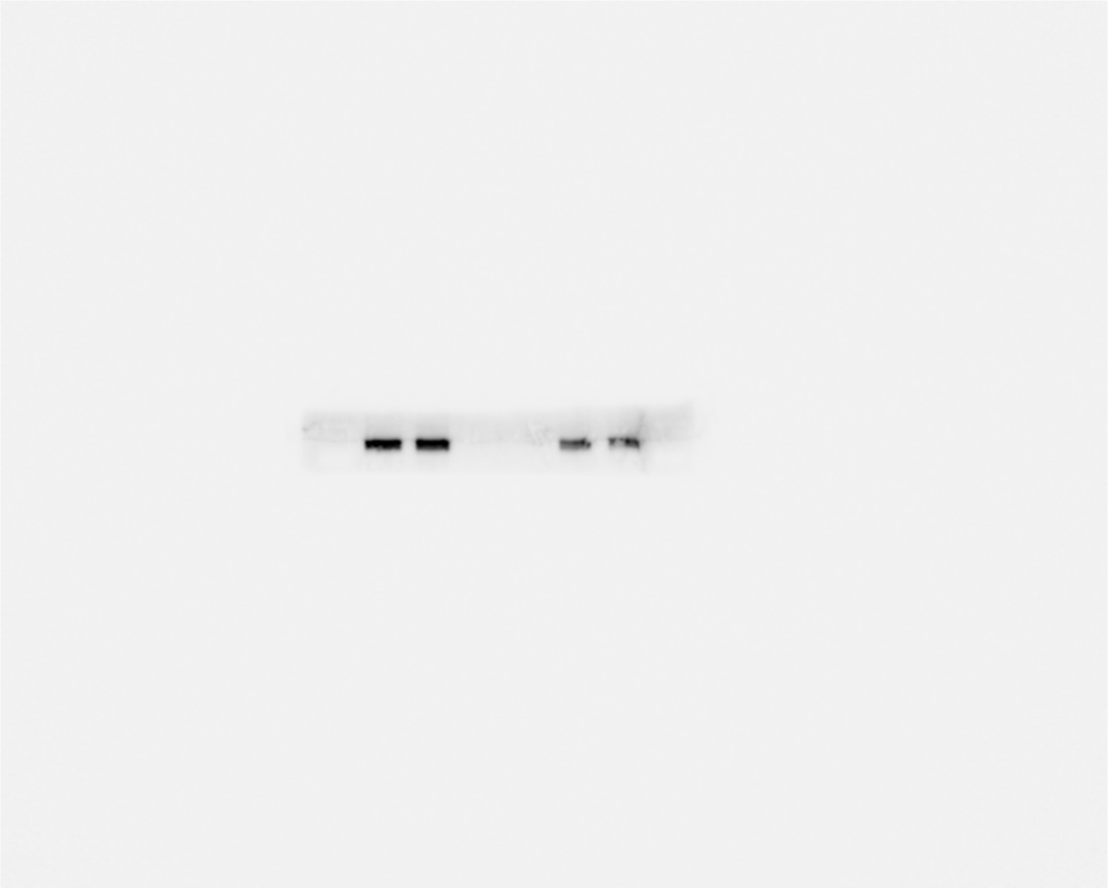

LDHA-Caki1

Fig. 3E

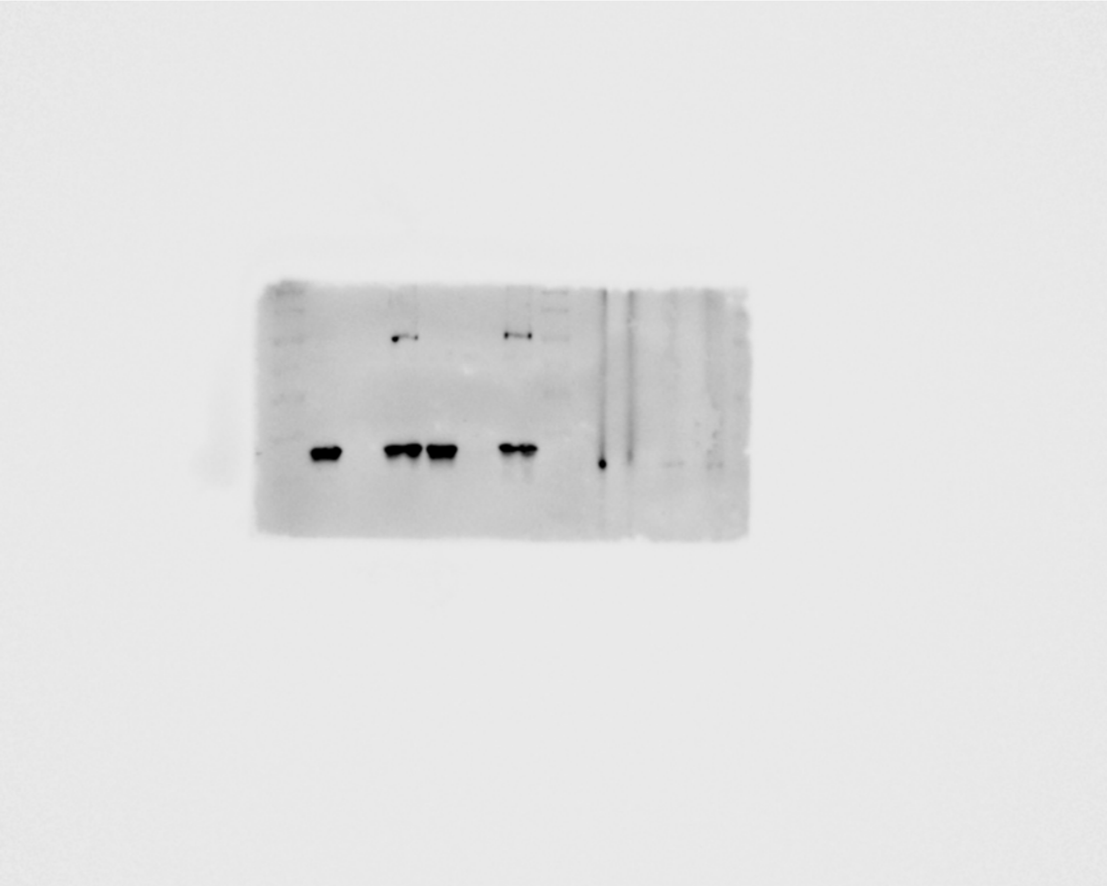

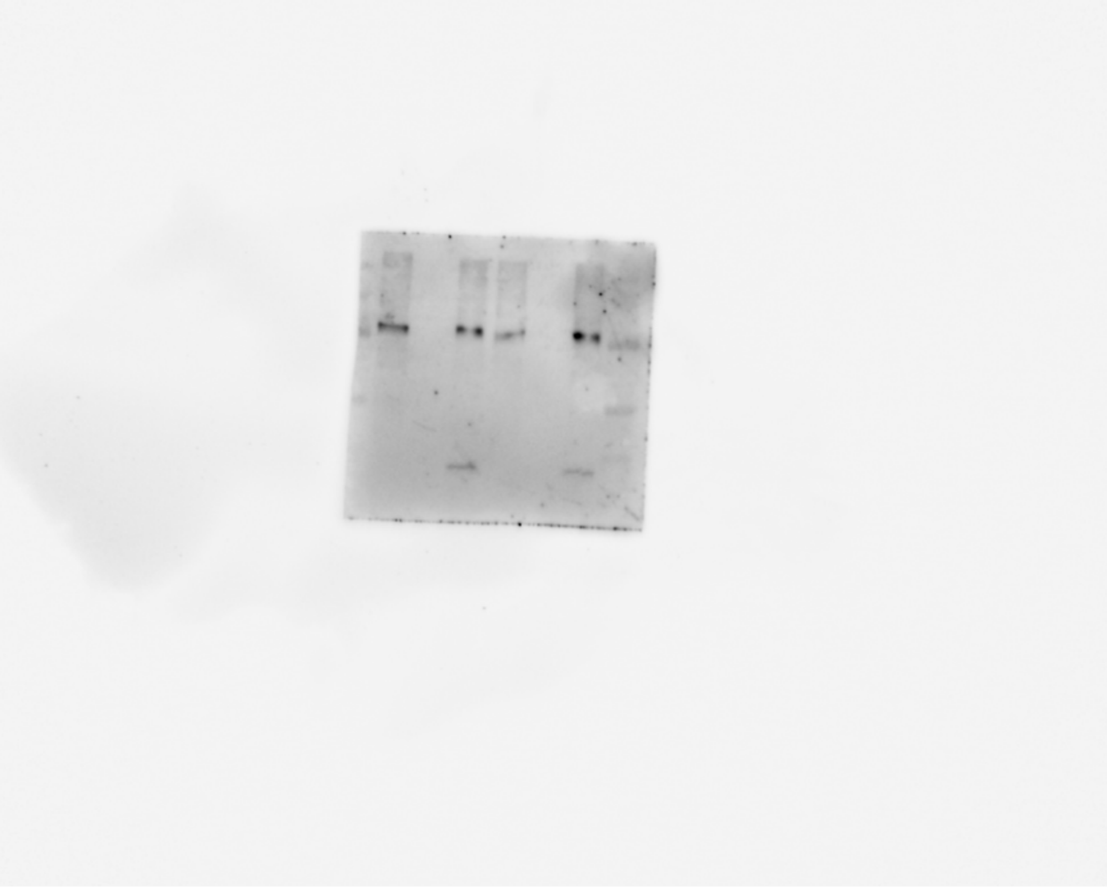

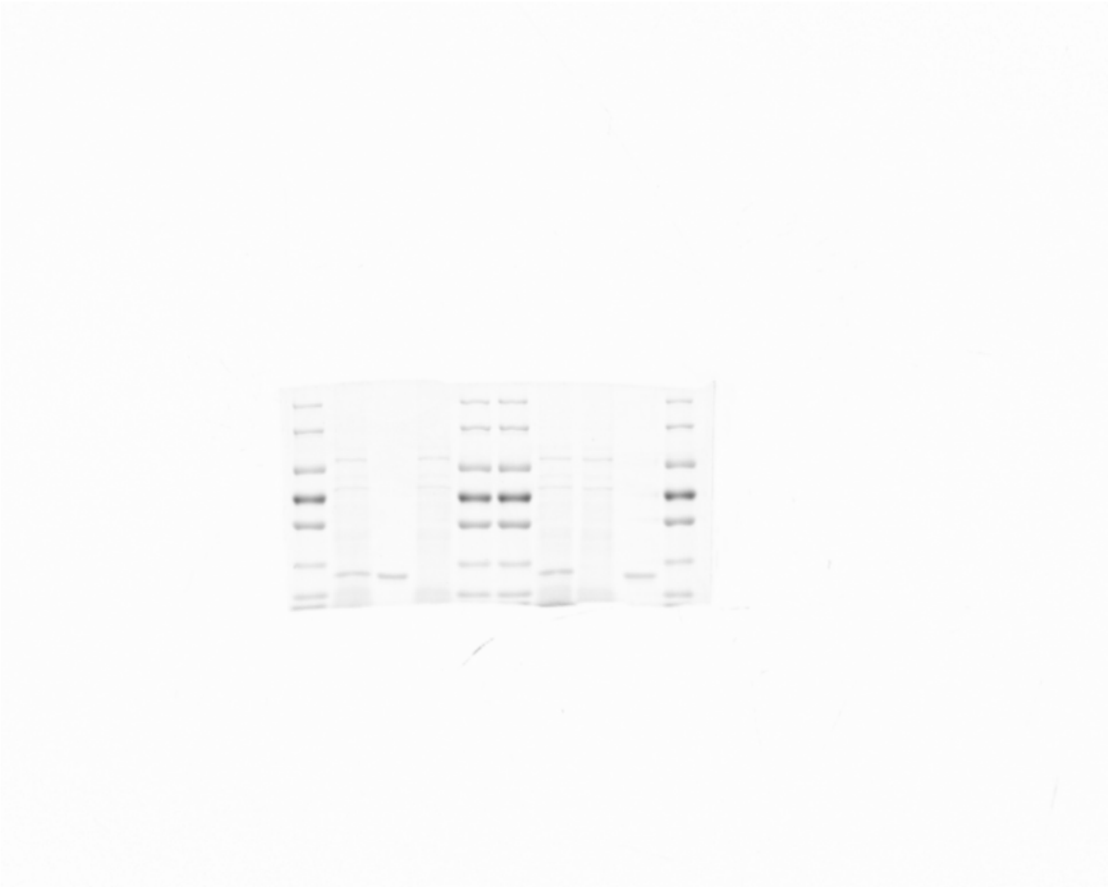

Fig. 3H

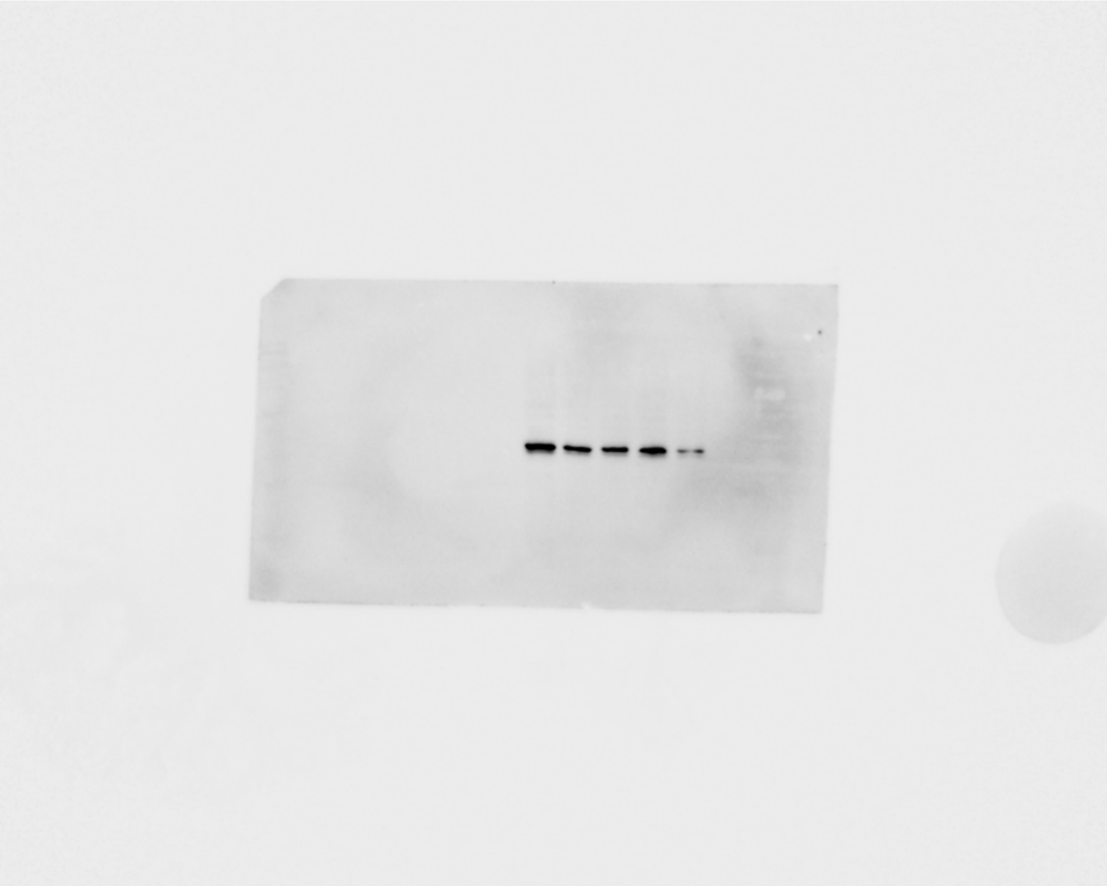

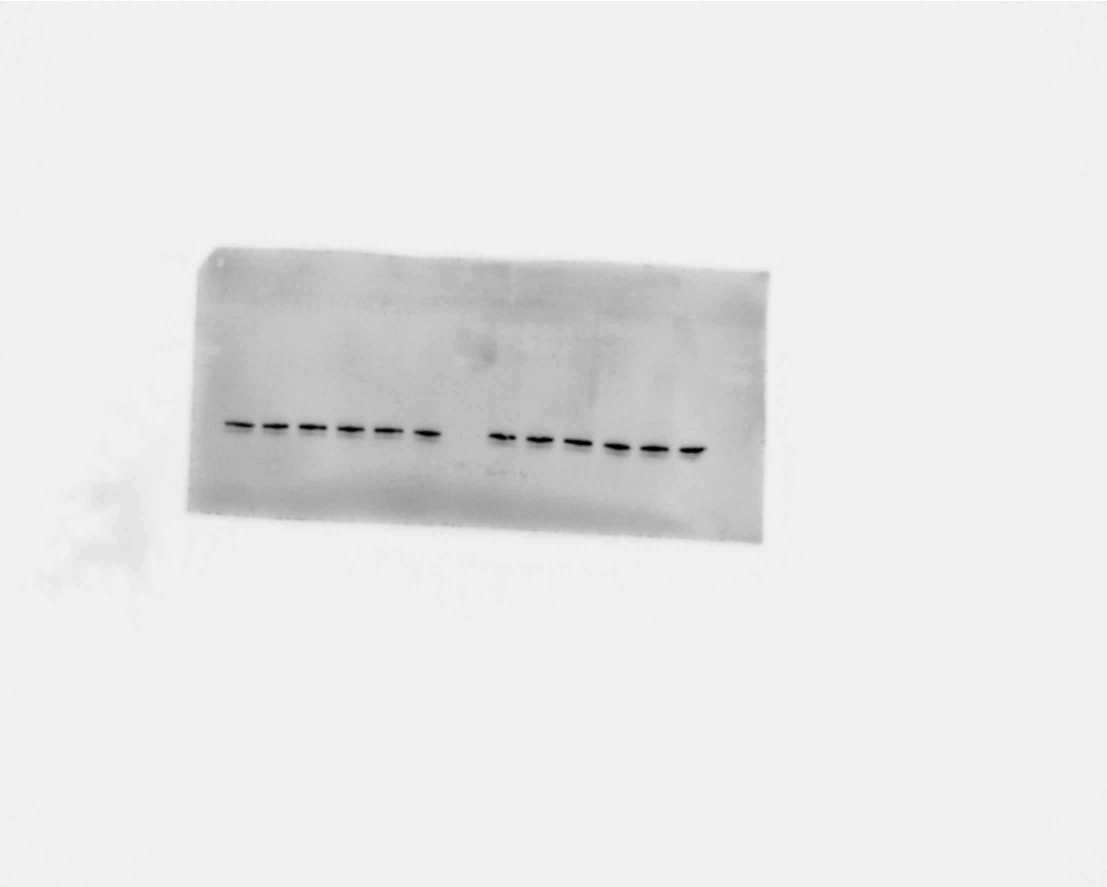

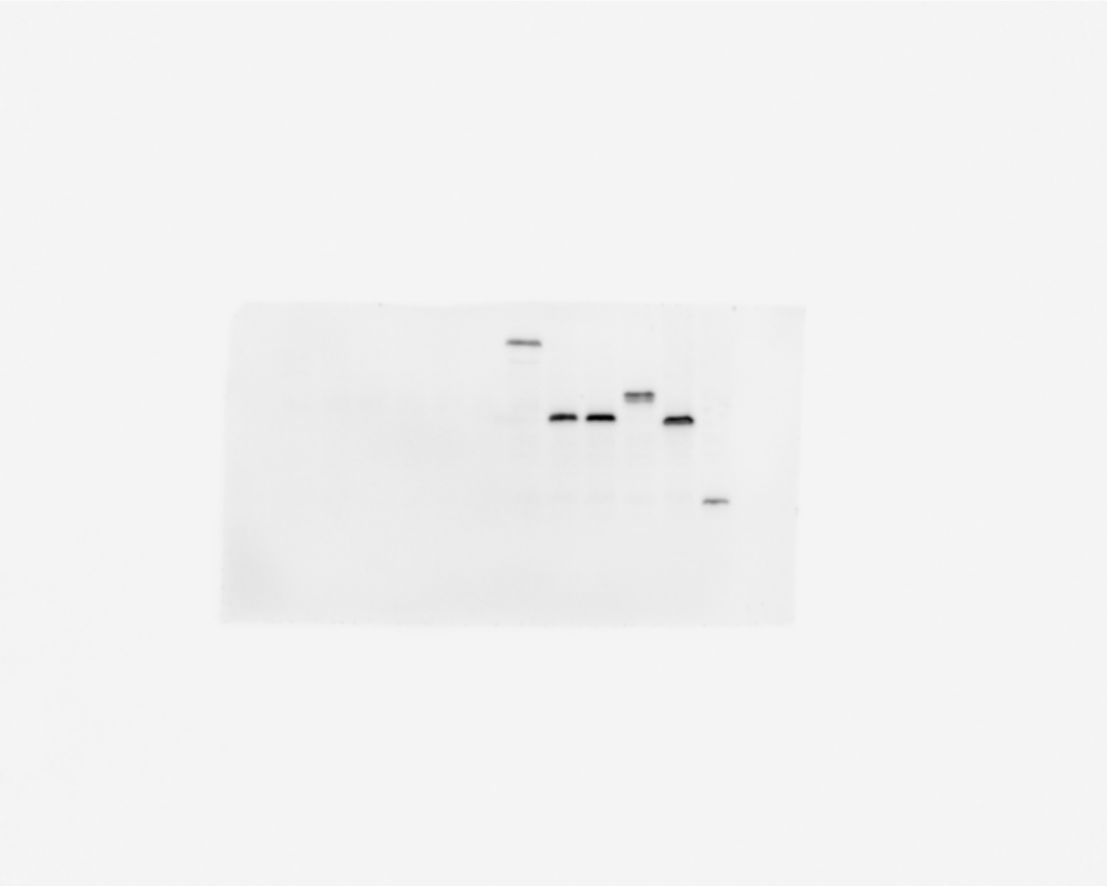

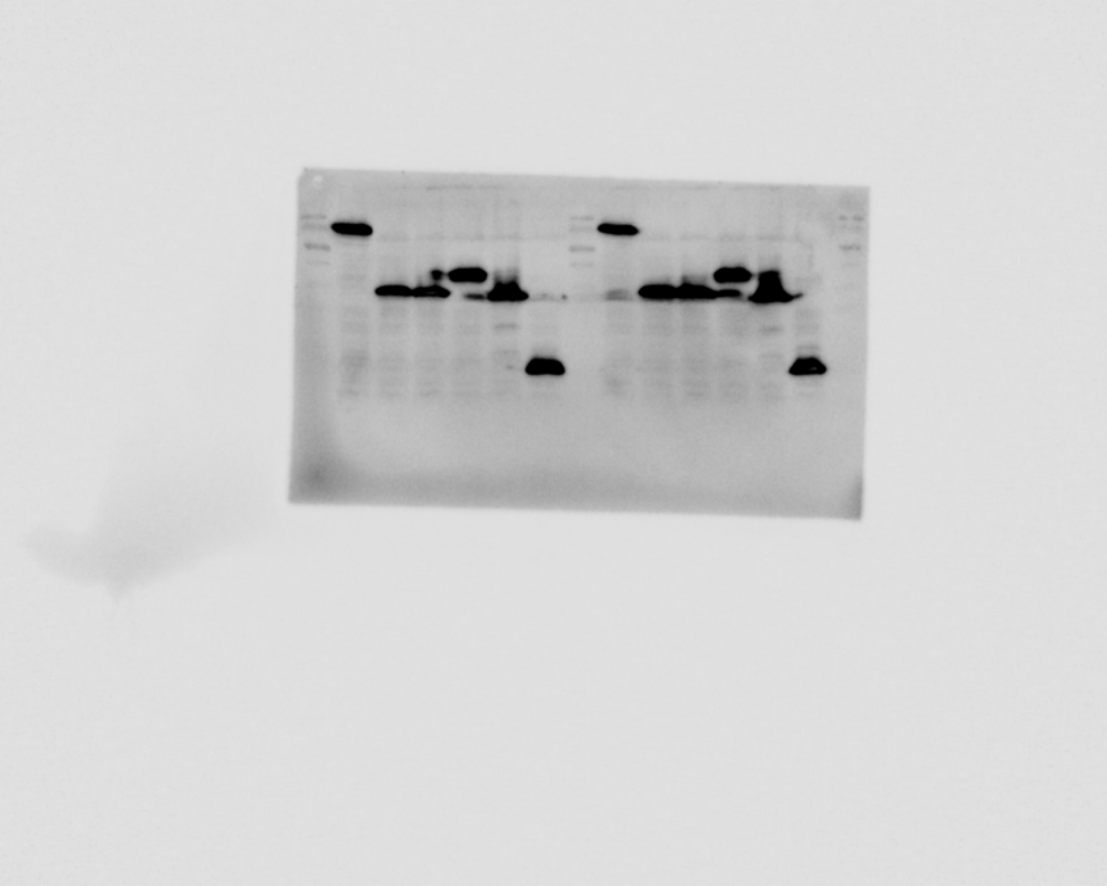

Fig. 3I

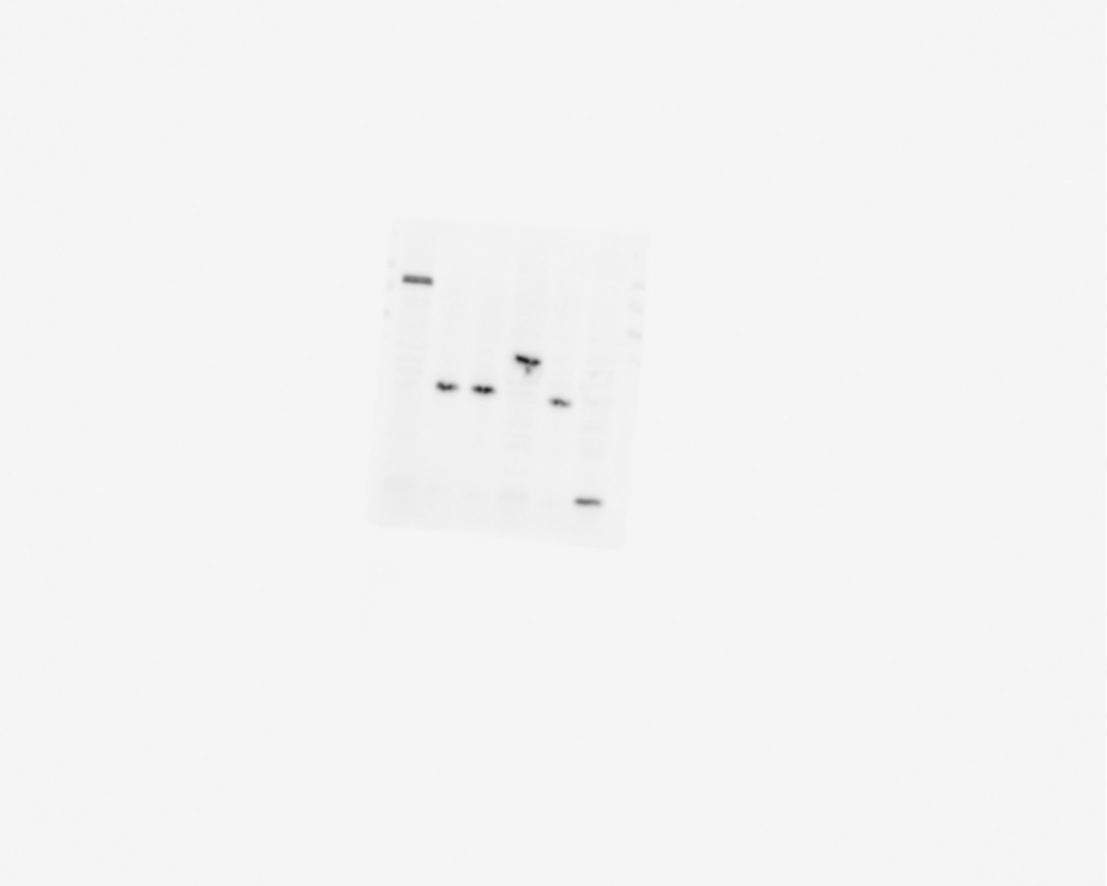

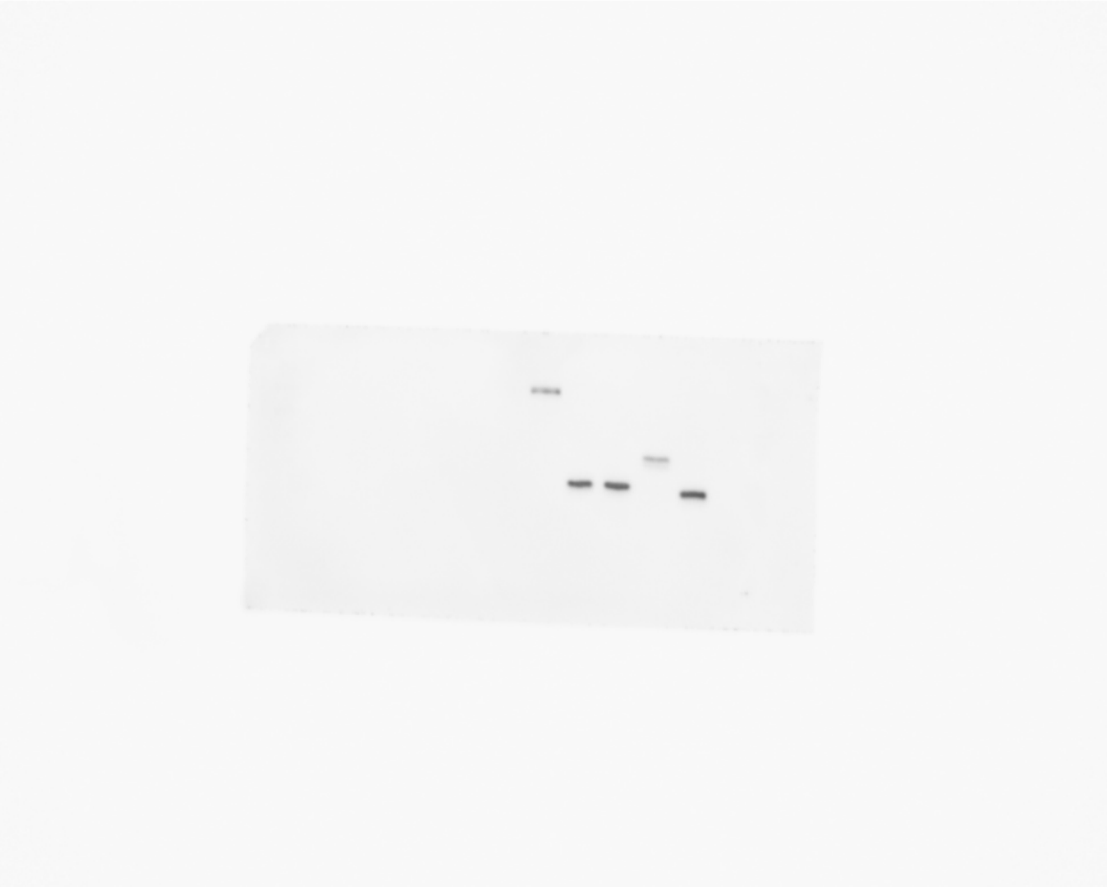

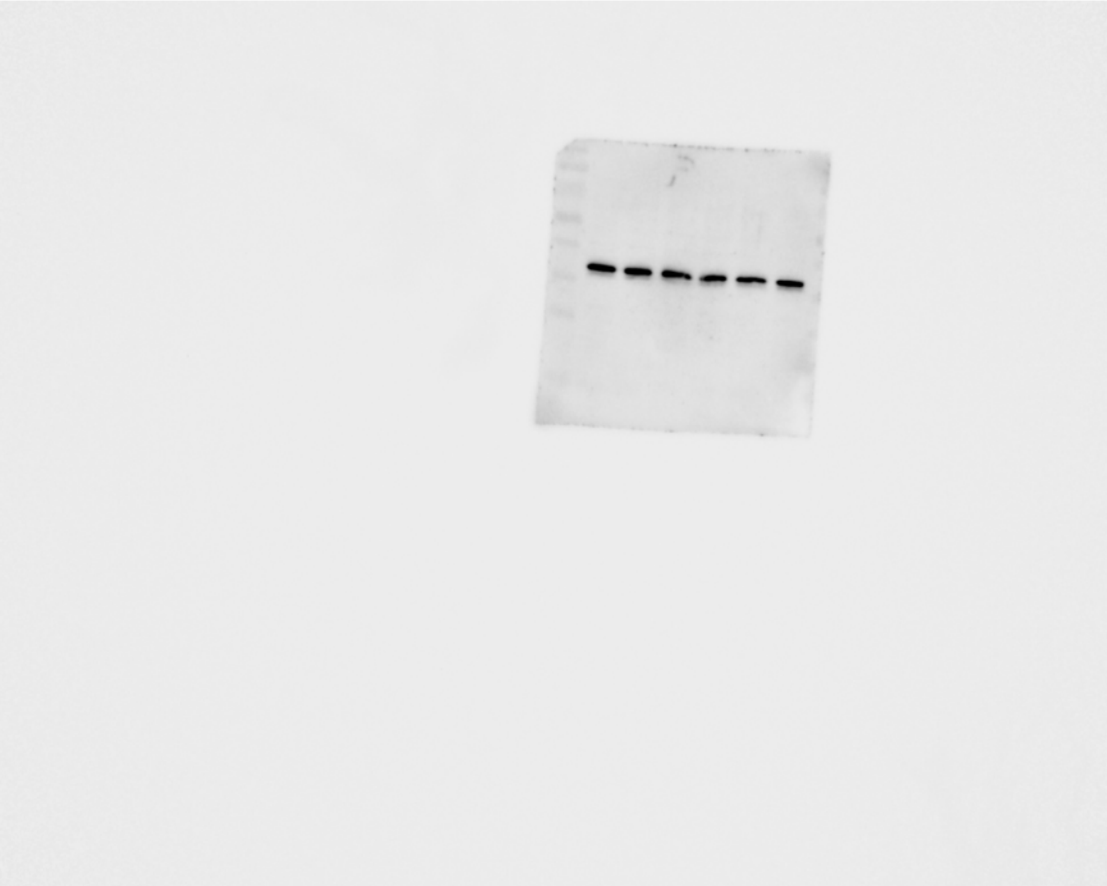

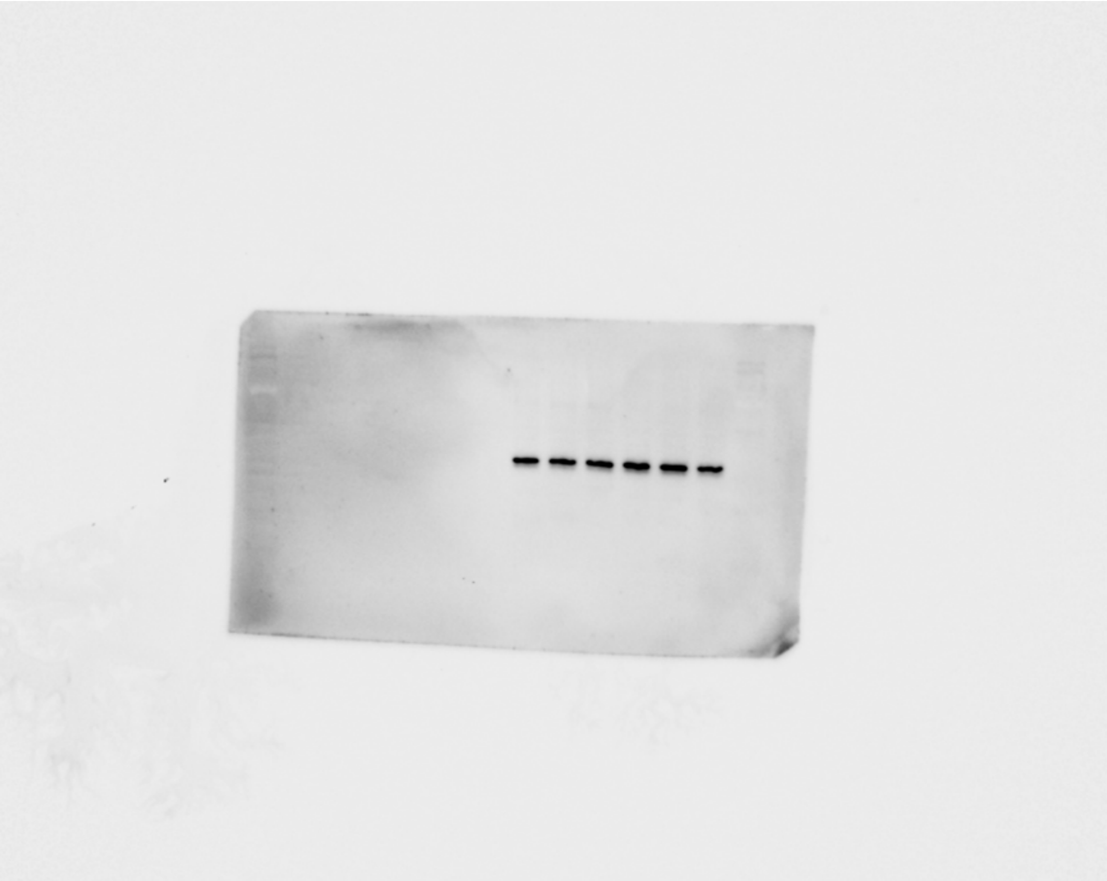

Fig. 3J

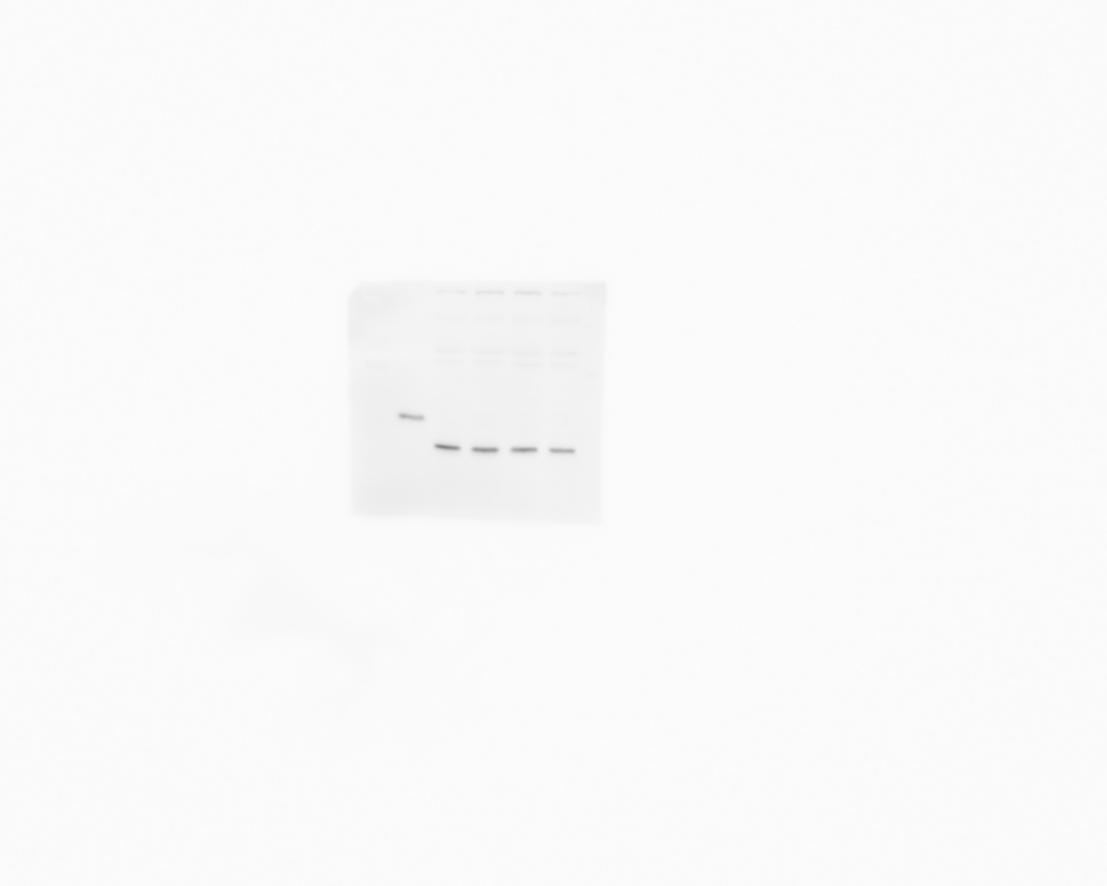

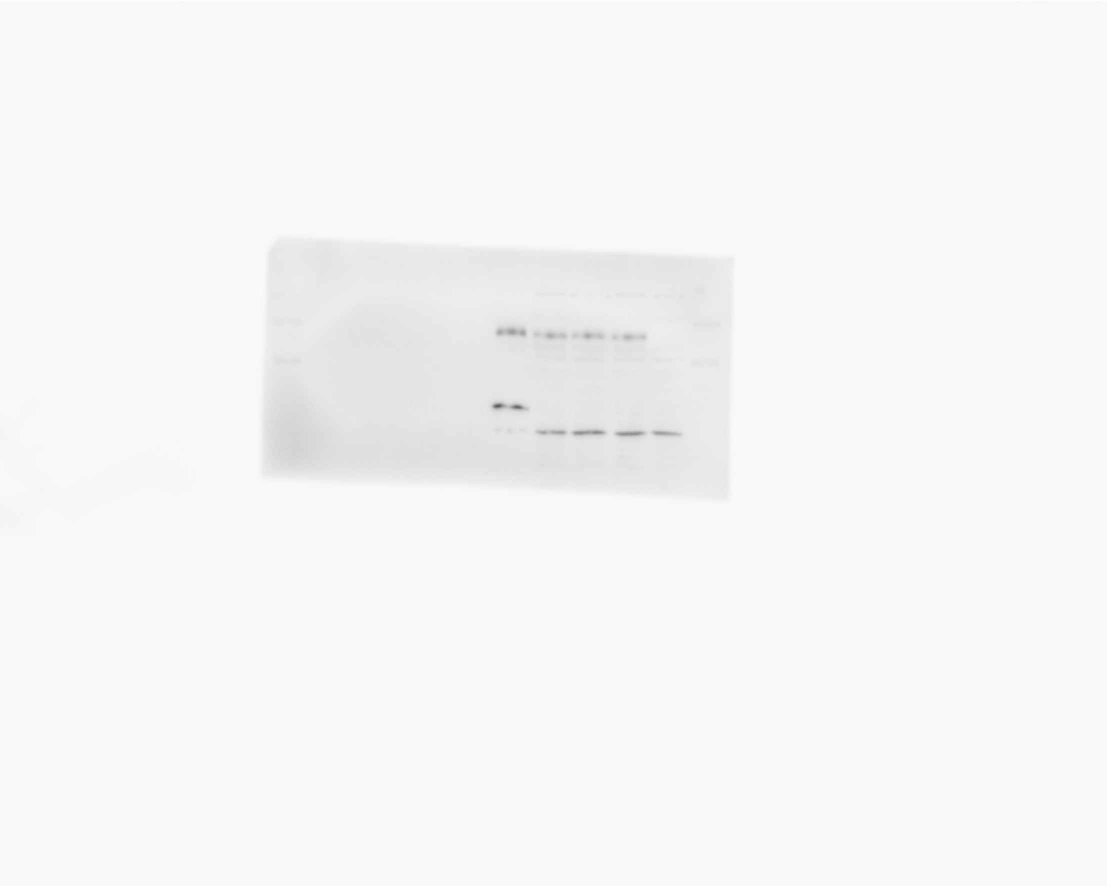

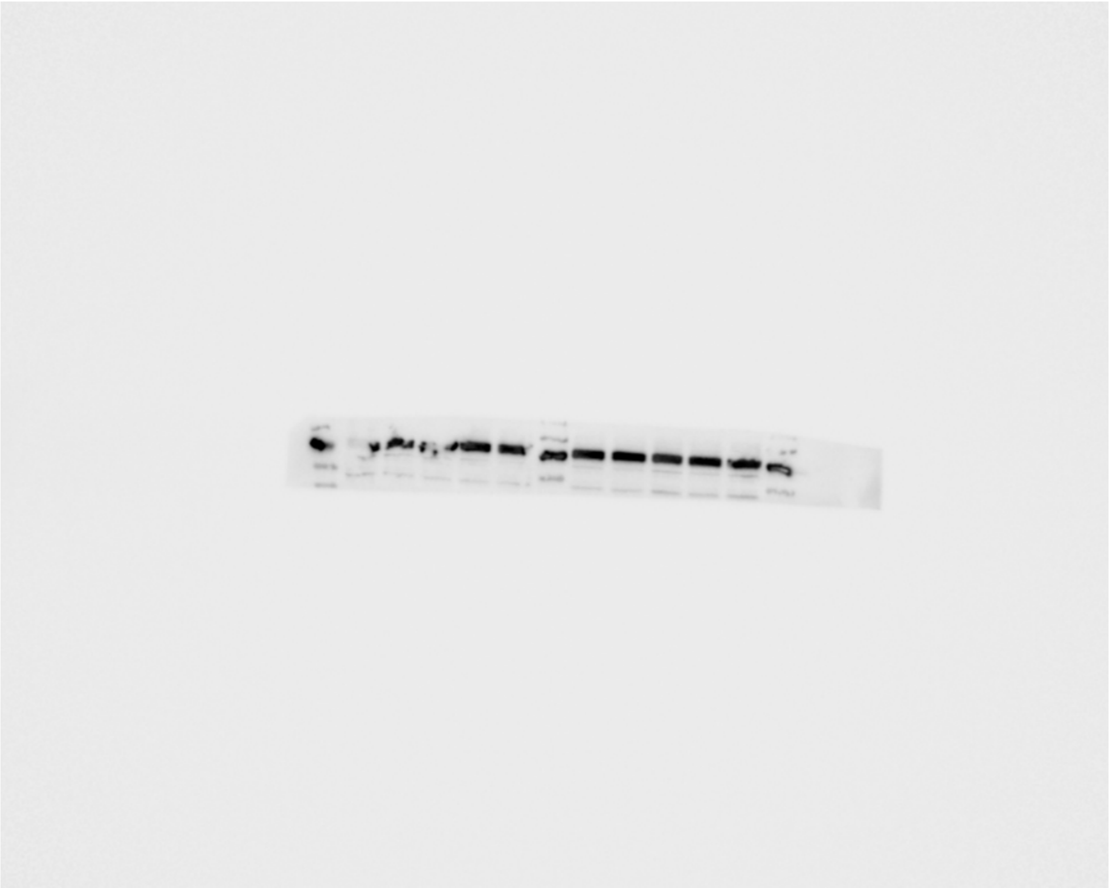

Fig. 3K

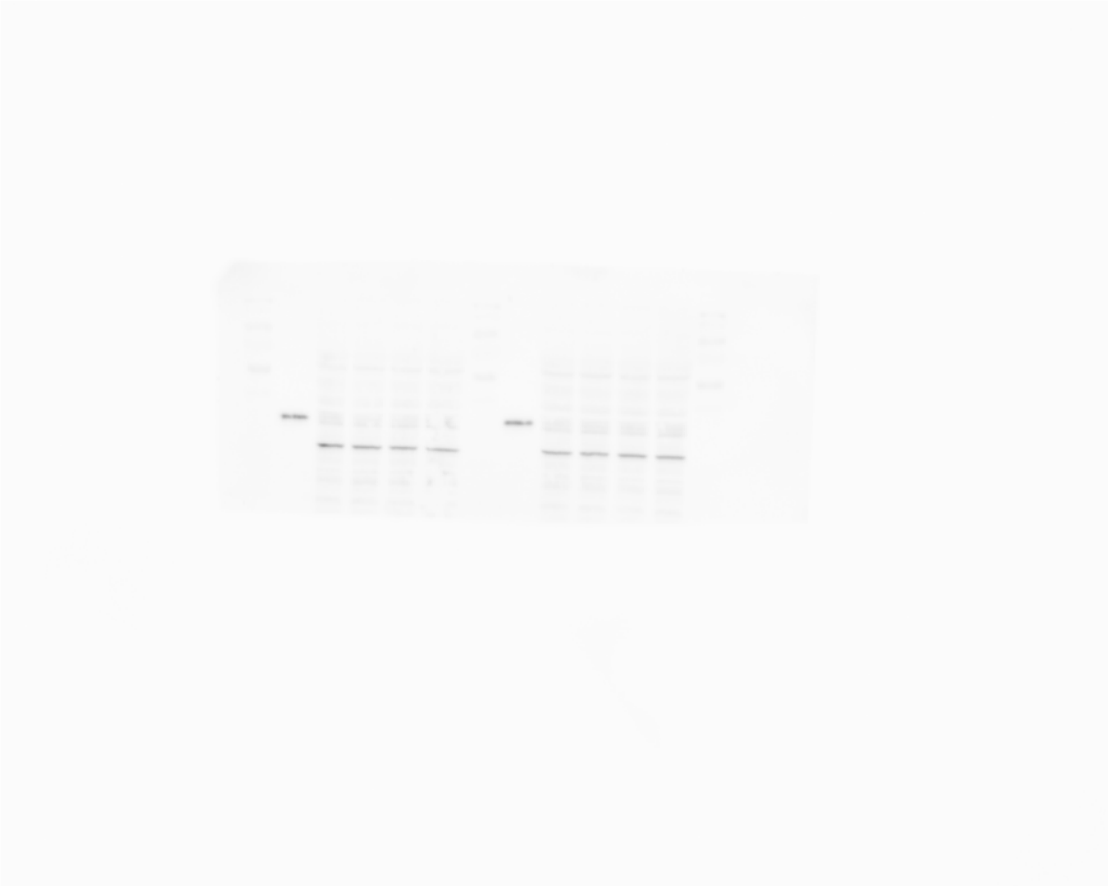

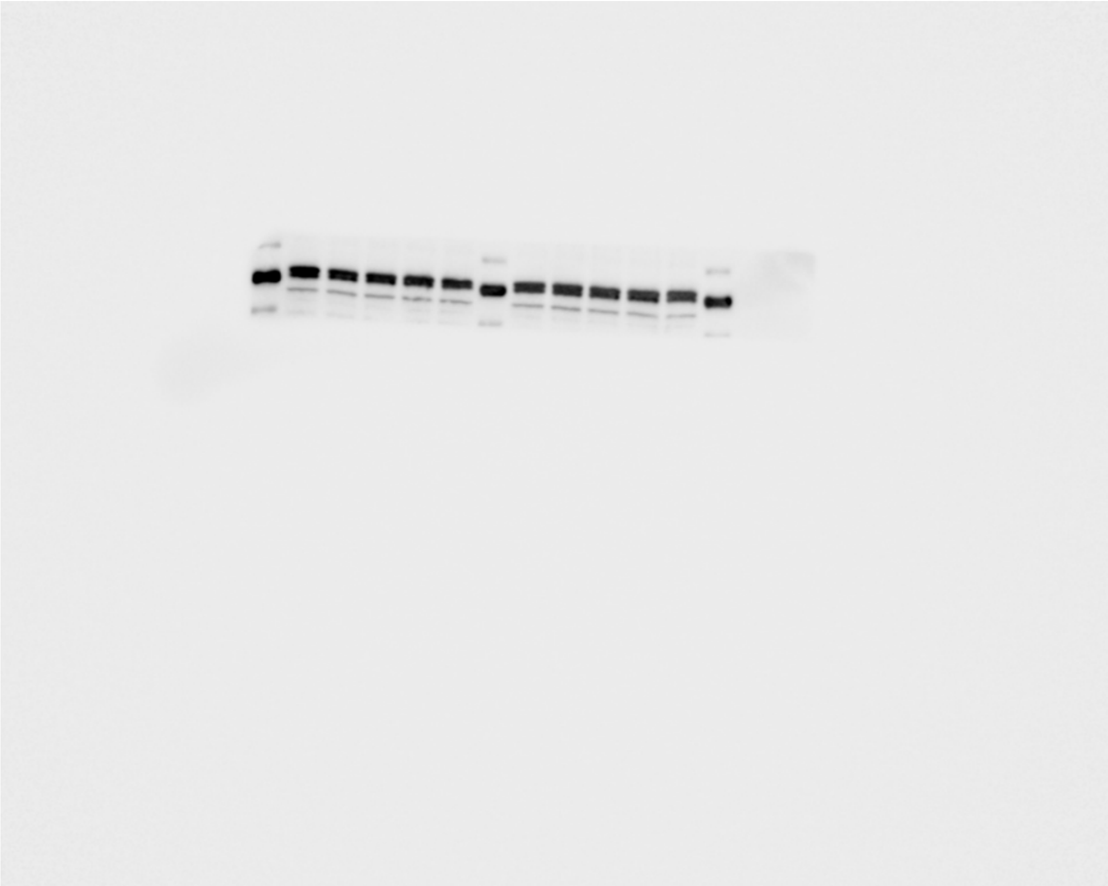

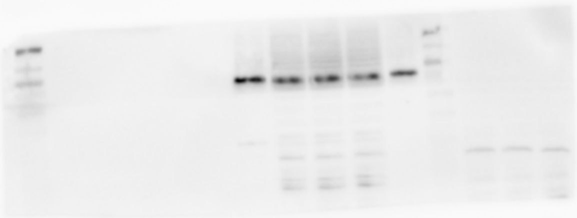

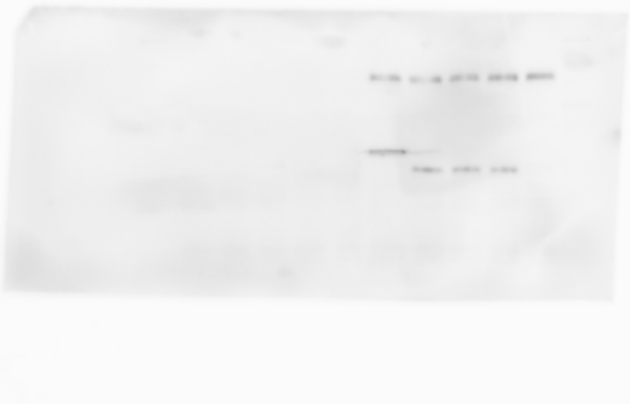

Fig. 4E

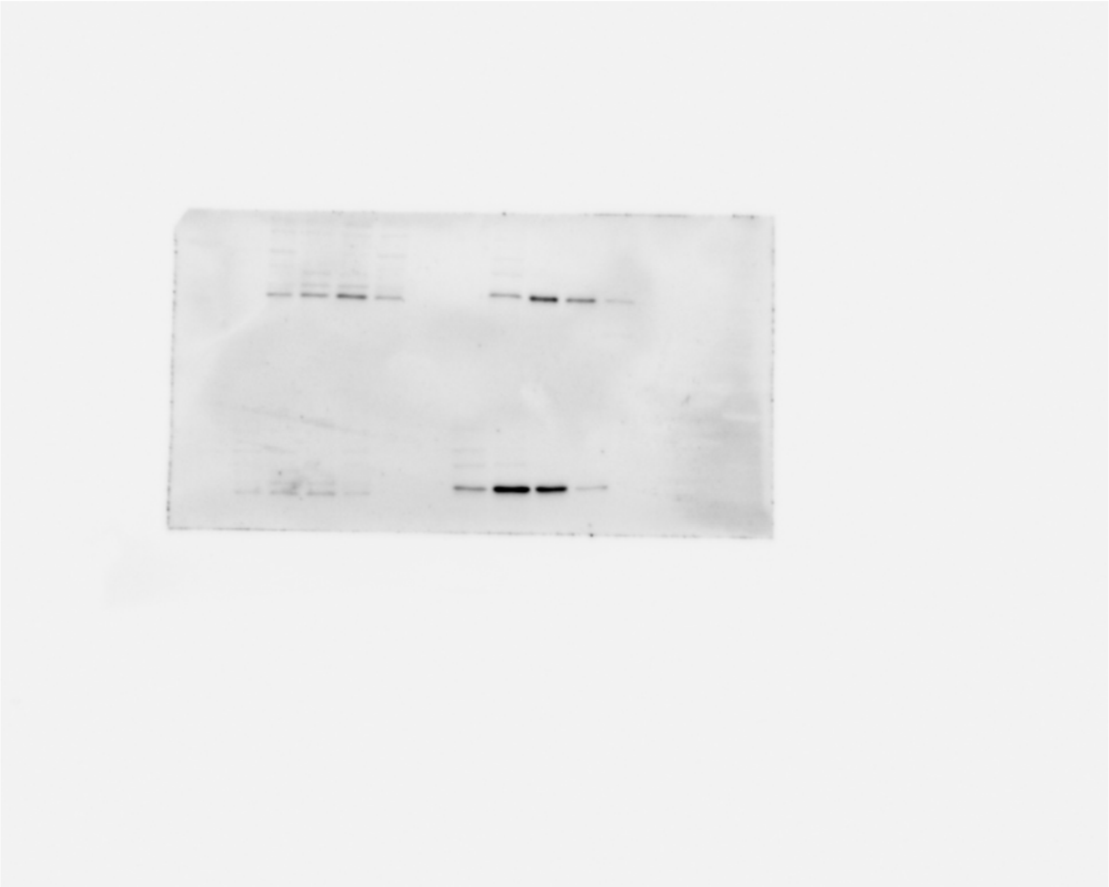

Fig. 4F
LDHB

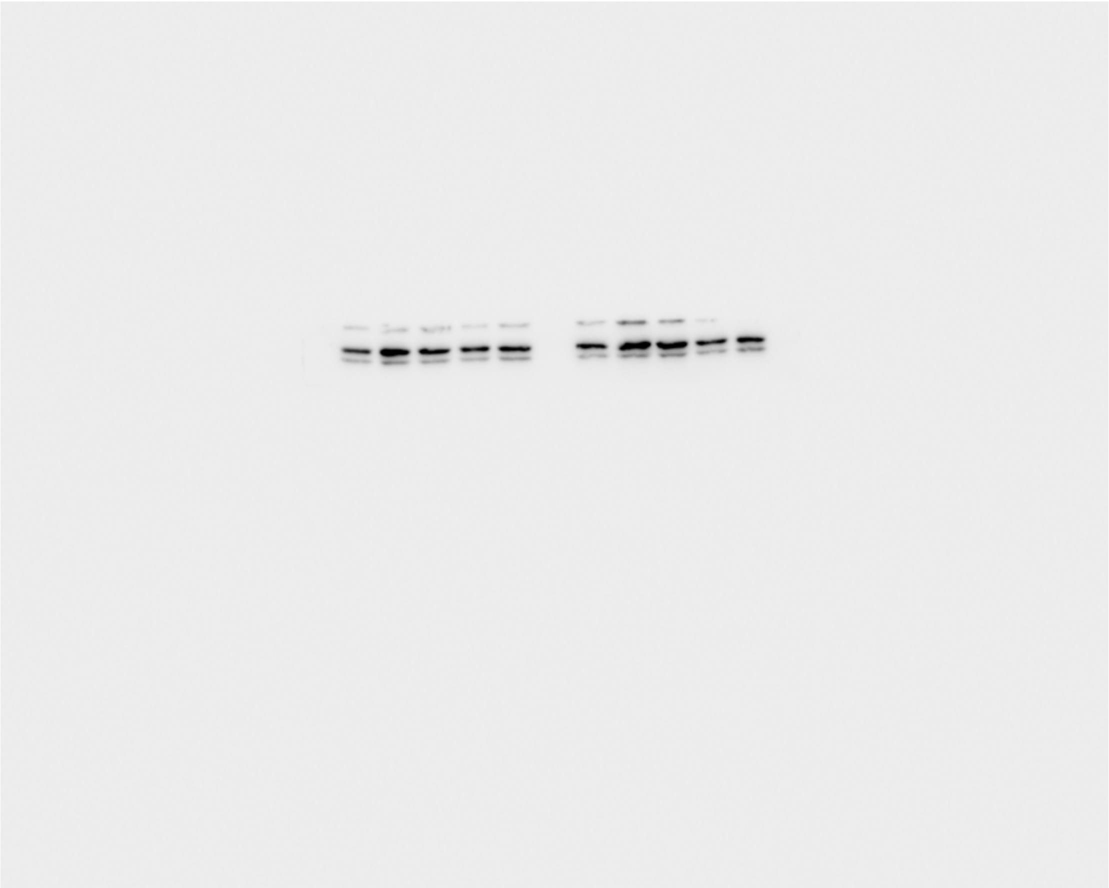

FGFR1

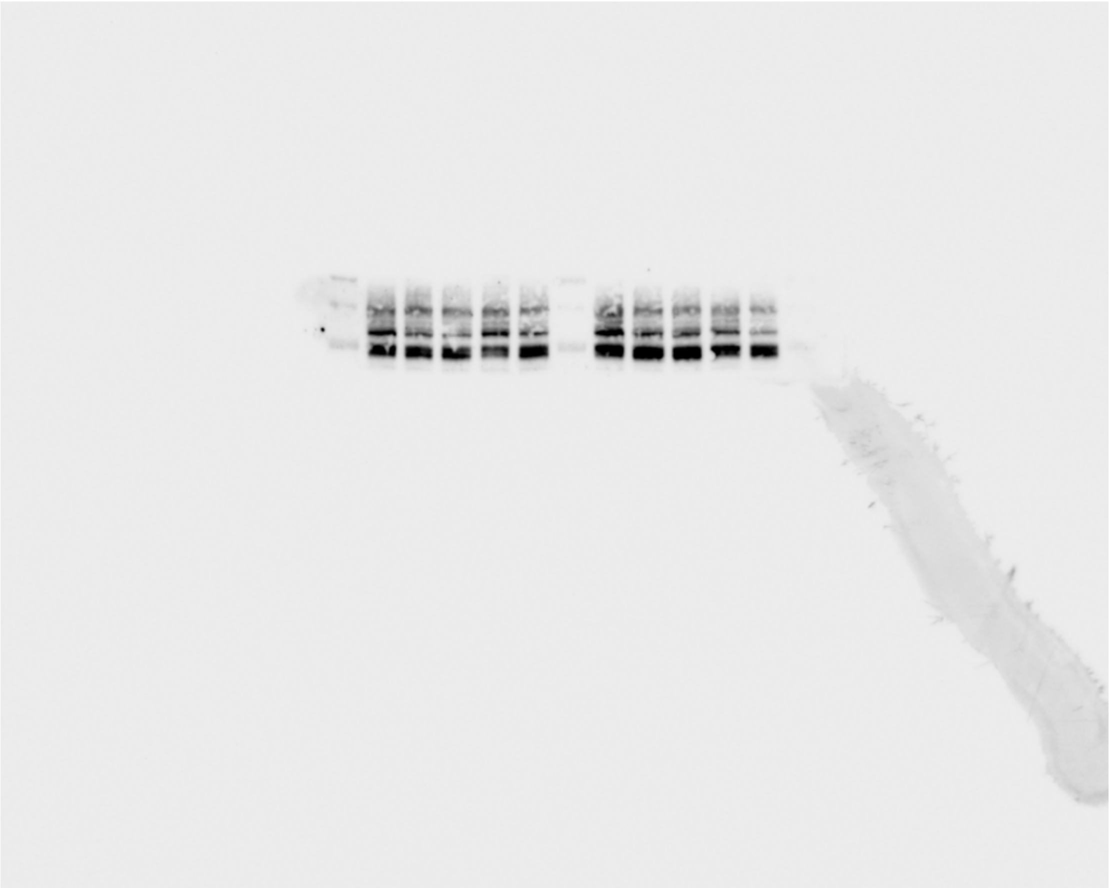

LDHA

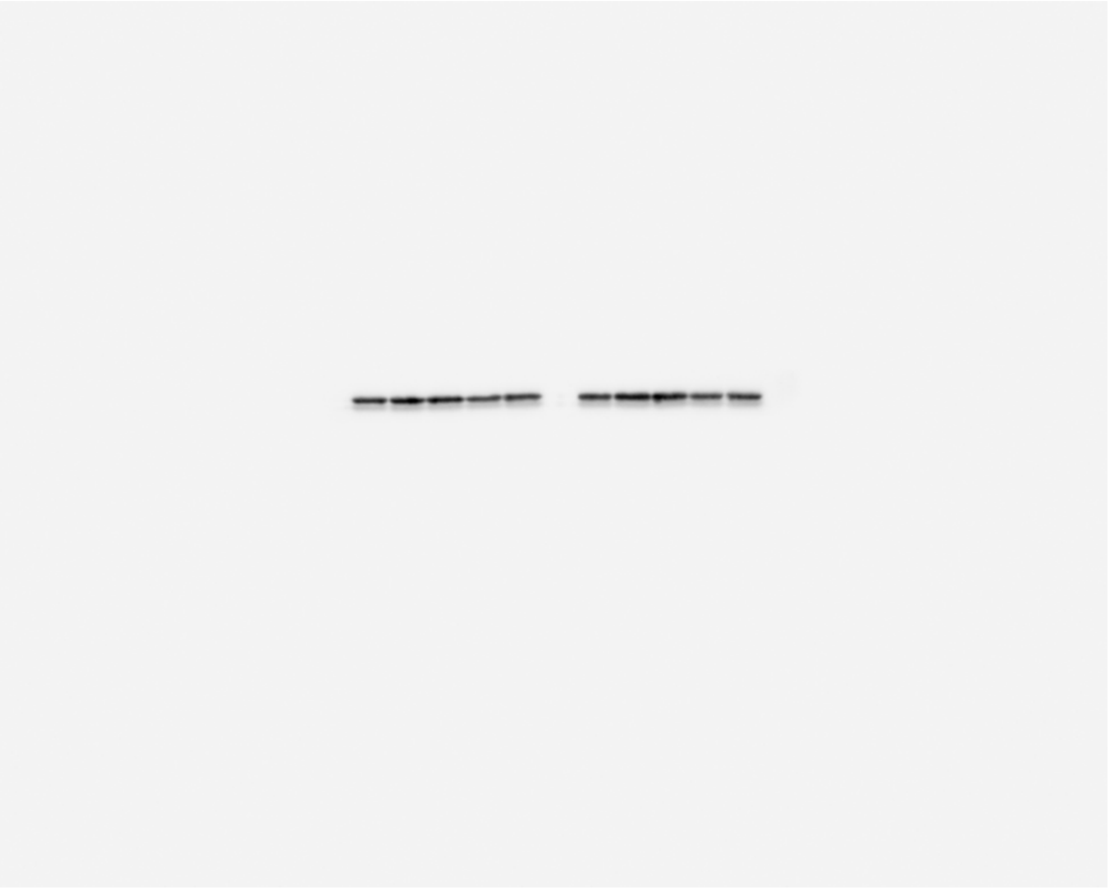

p-LDHA

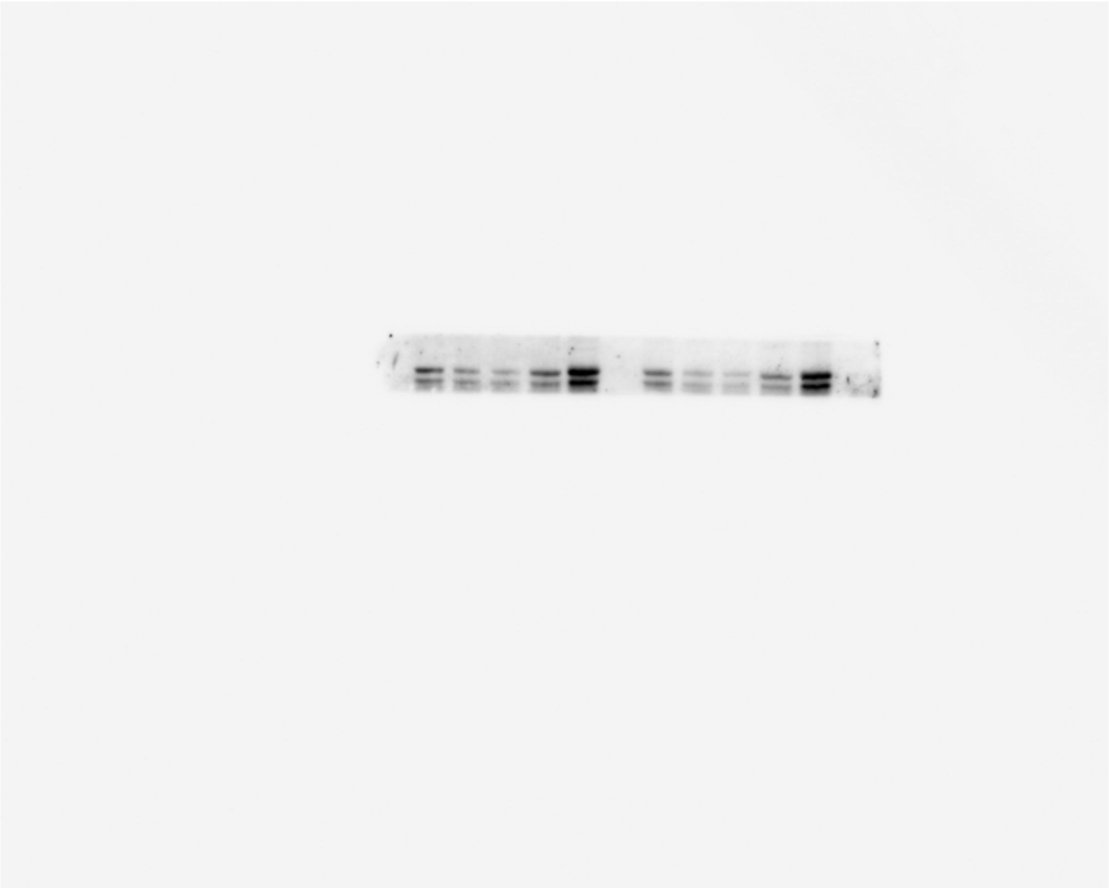

FKBP10

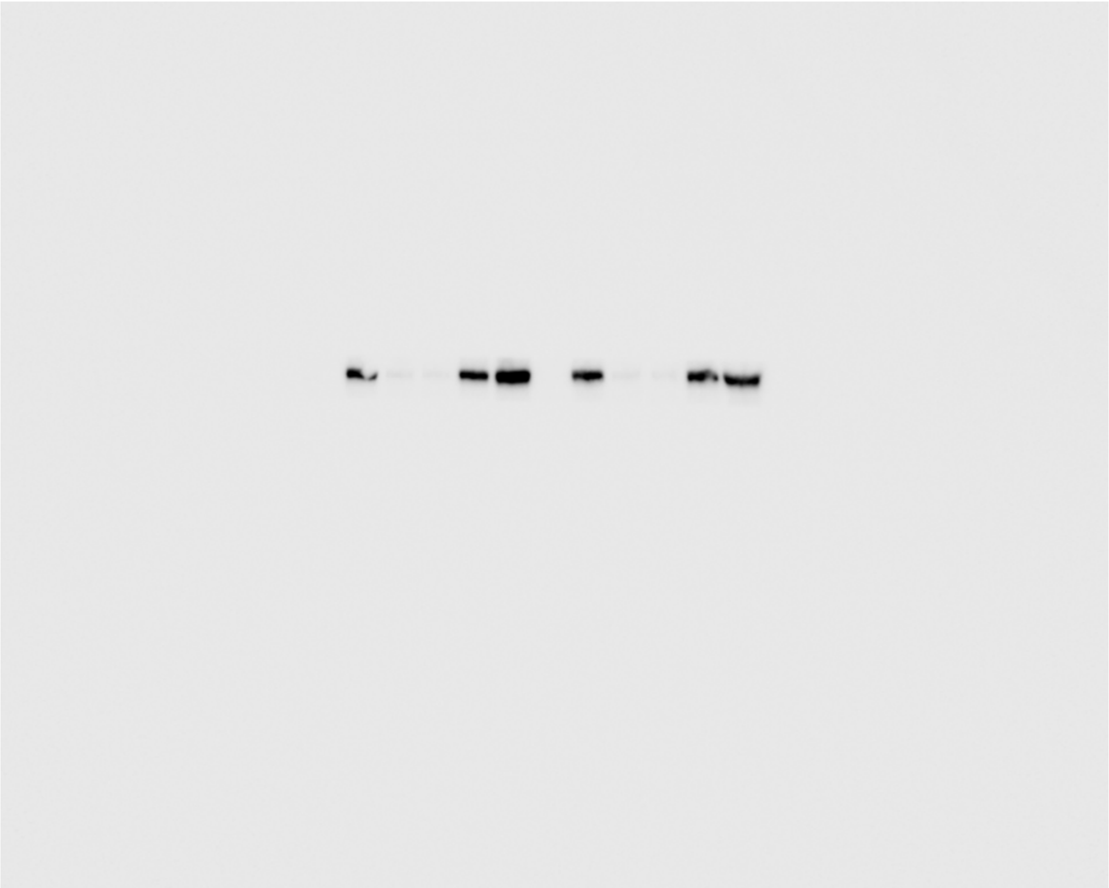

Actin

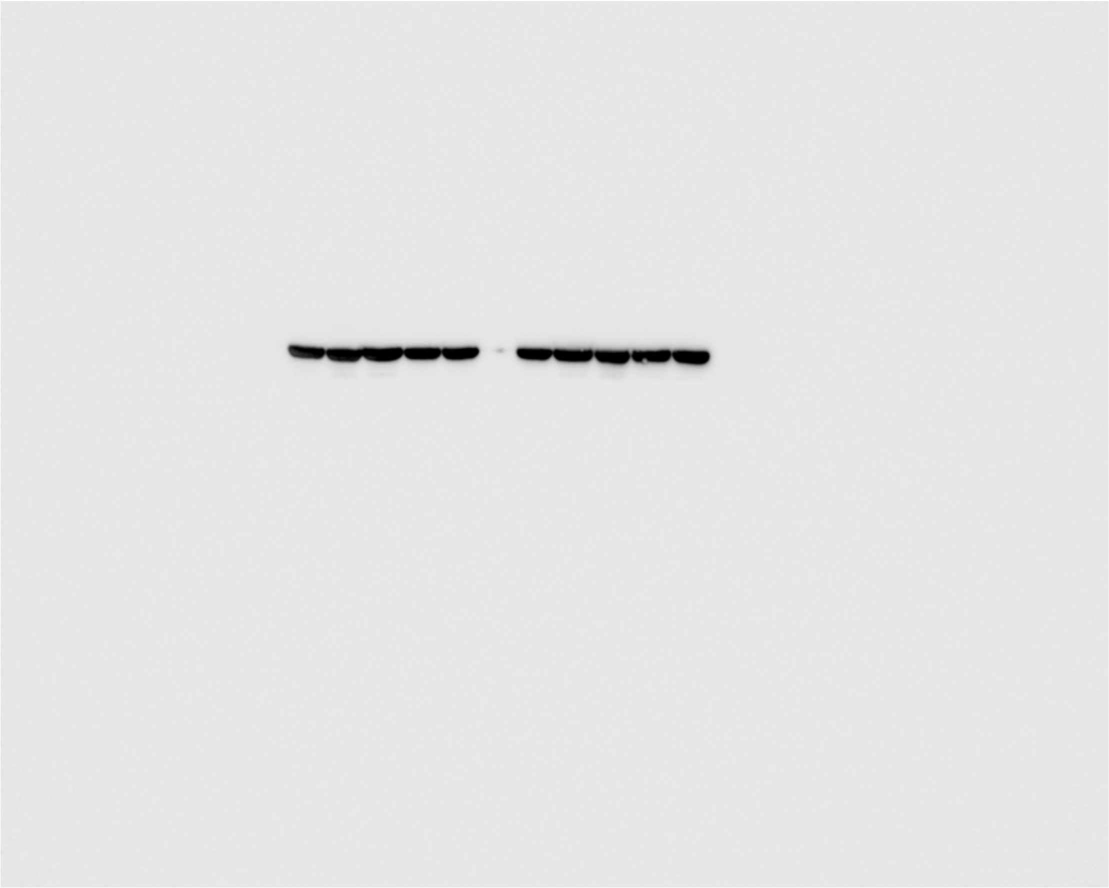

Fig. 4G
Actin

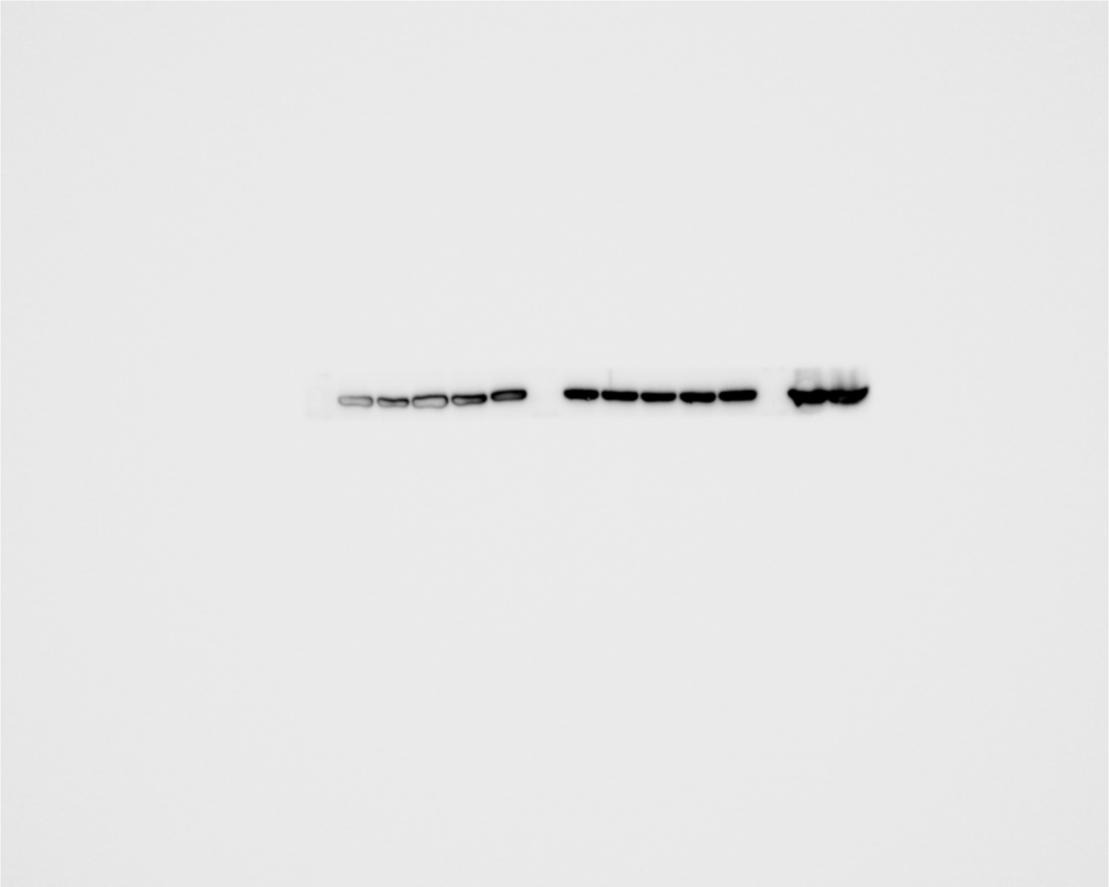

LDHB

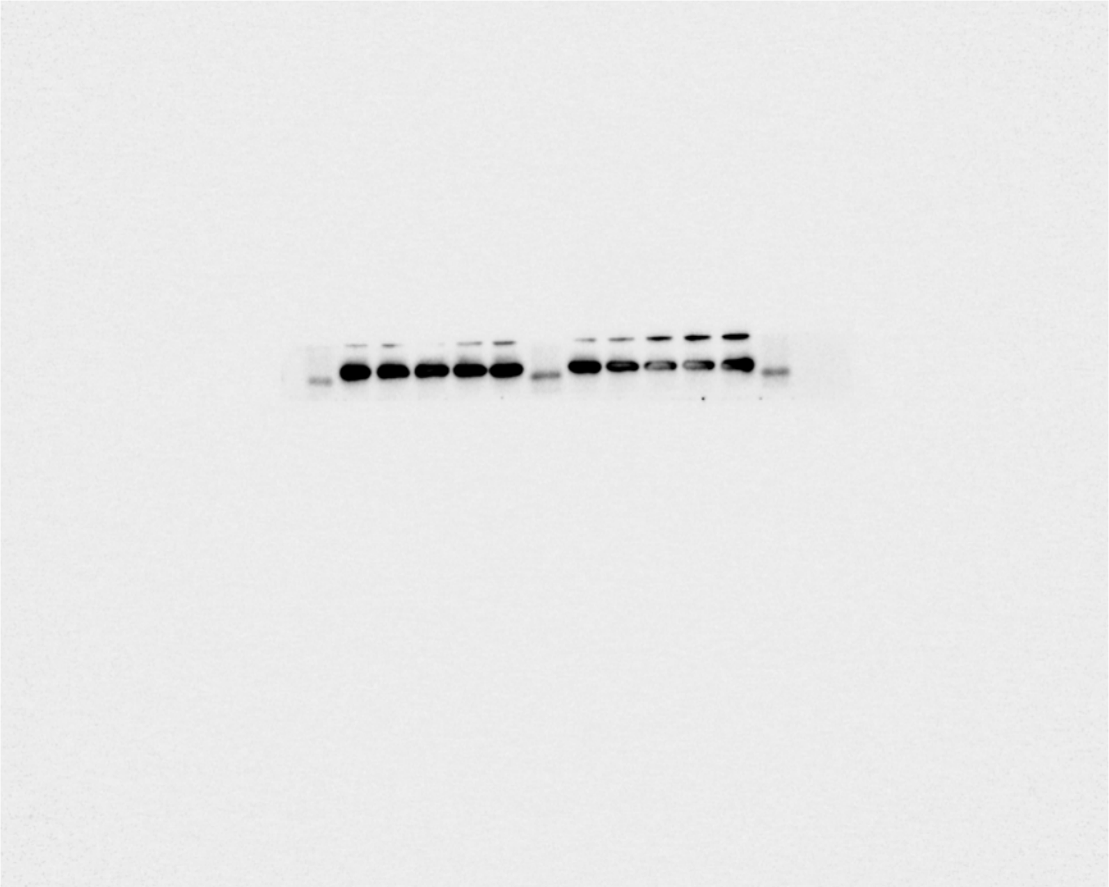

LDHA

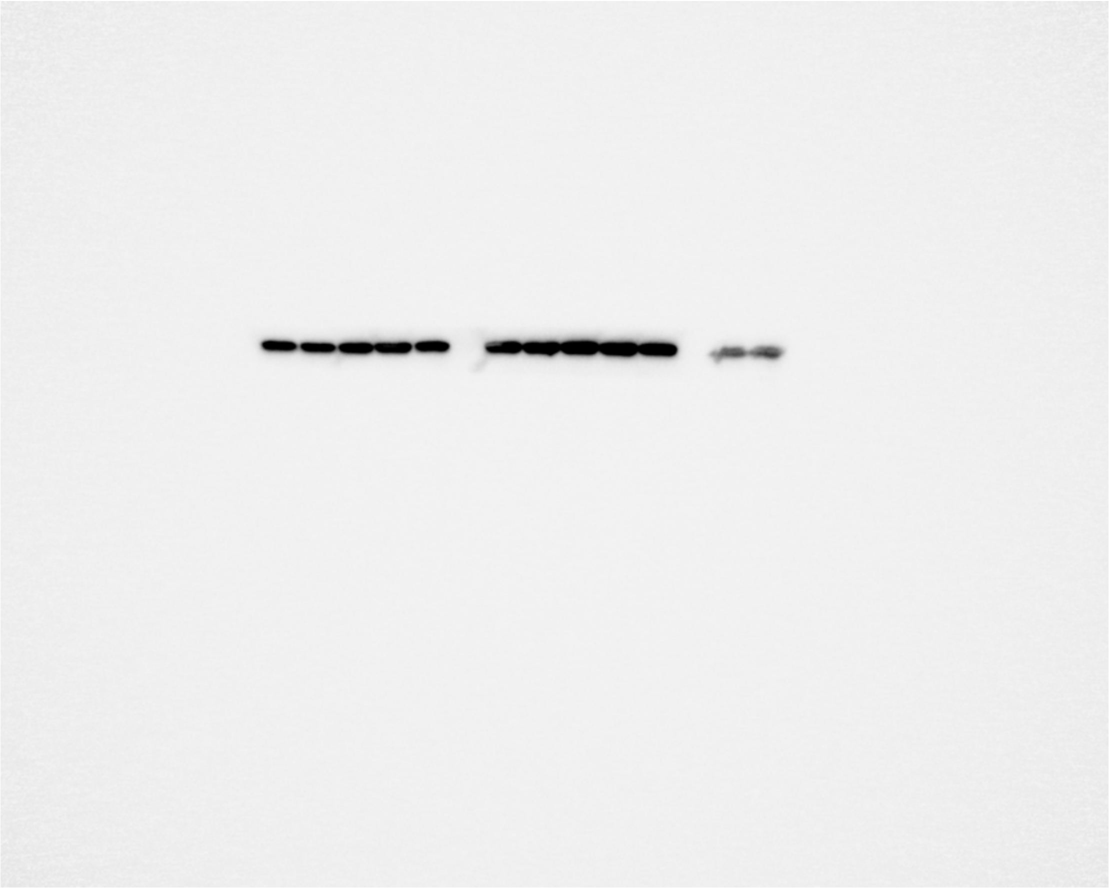

pLDHA

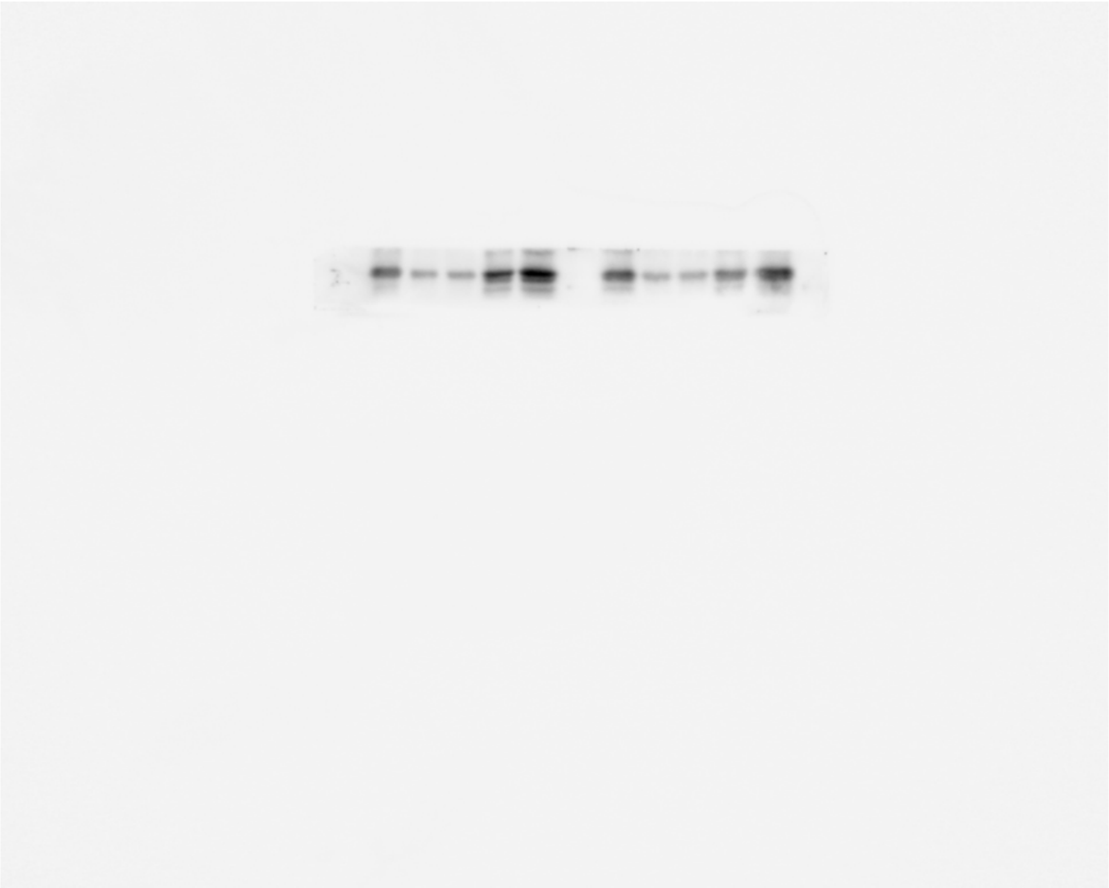

FKBP10

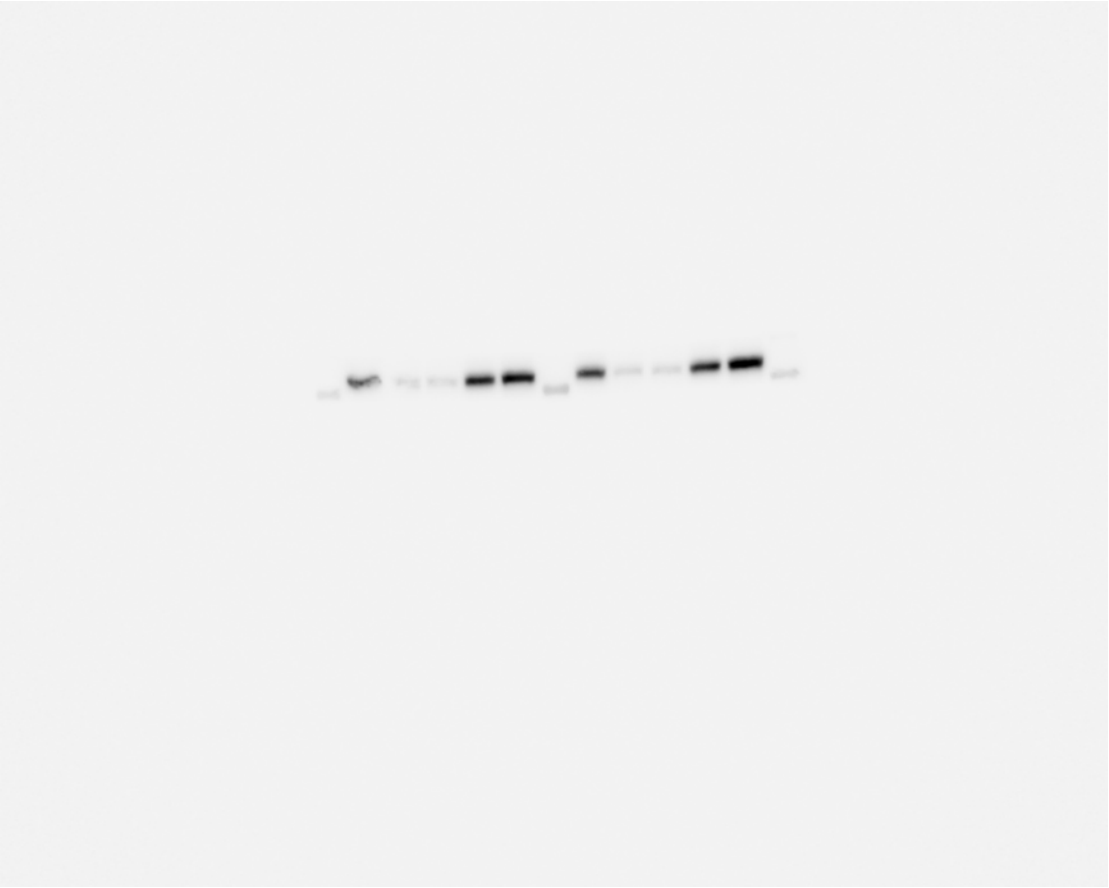

FGFR1

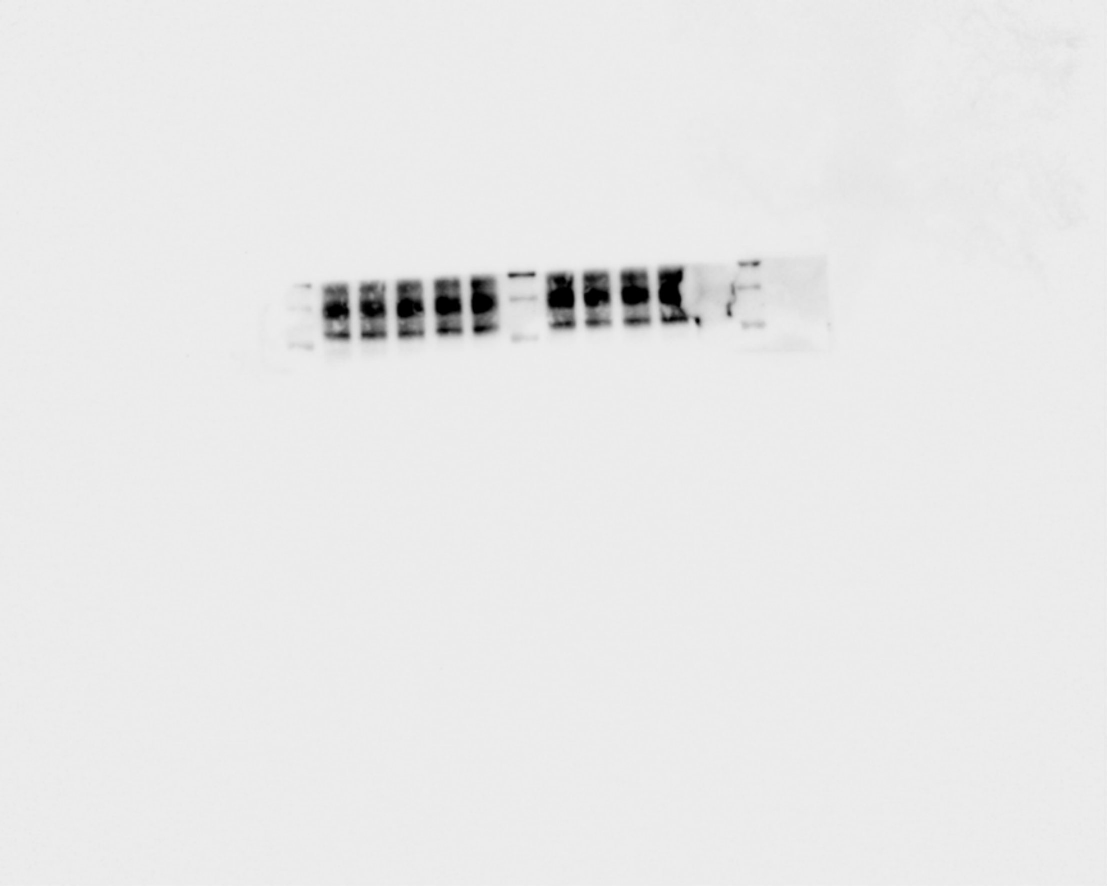

Fig. 4H

Actin

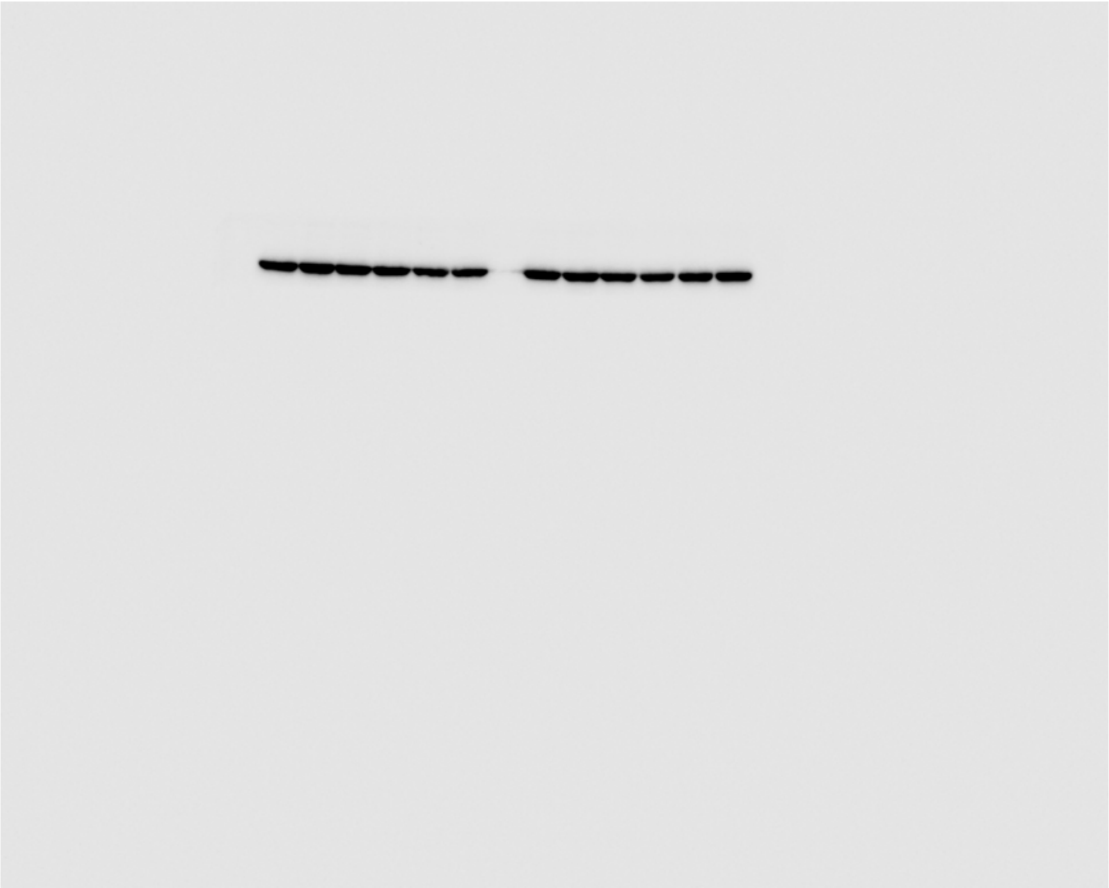

LDHA

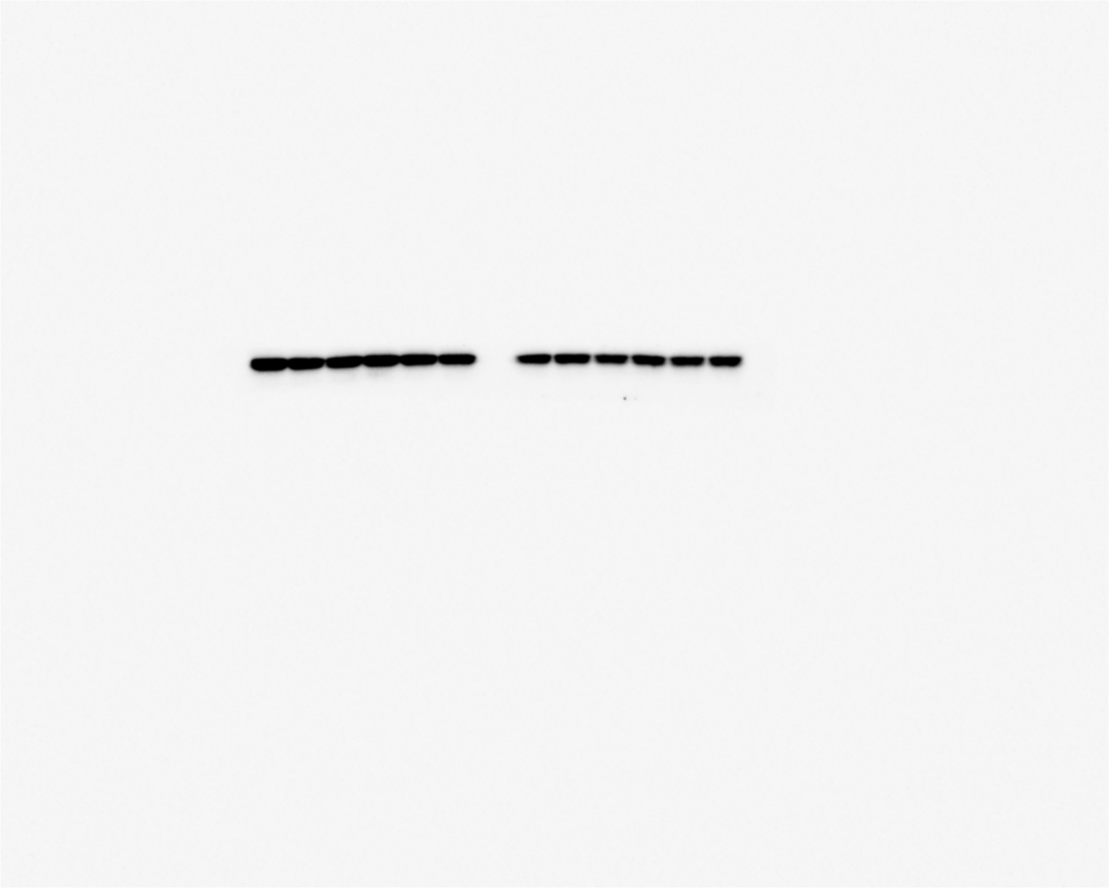

pLDHA

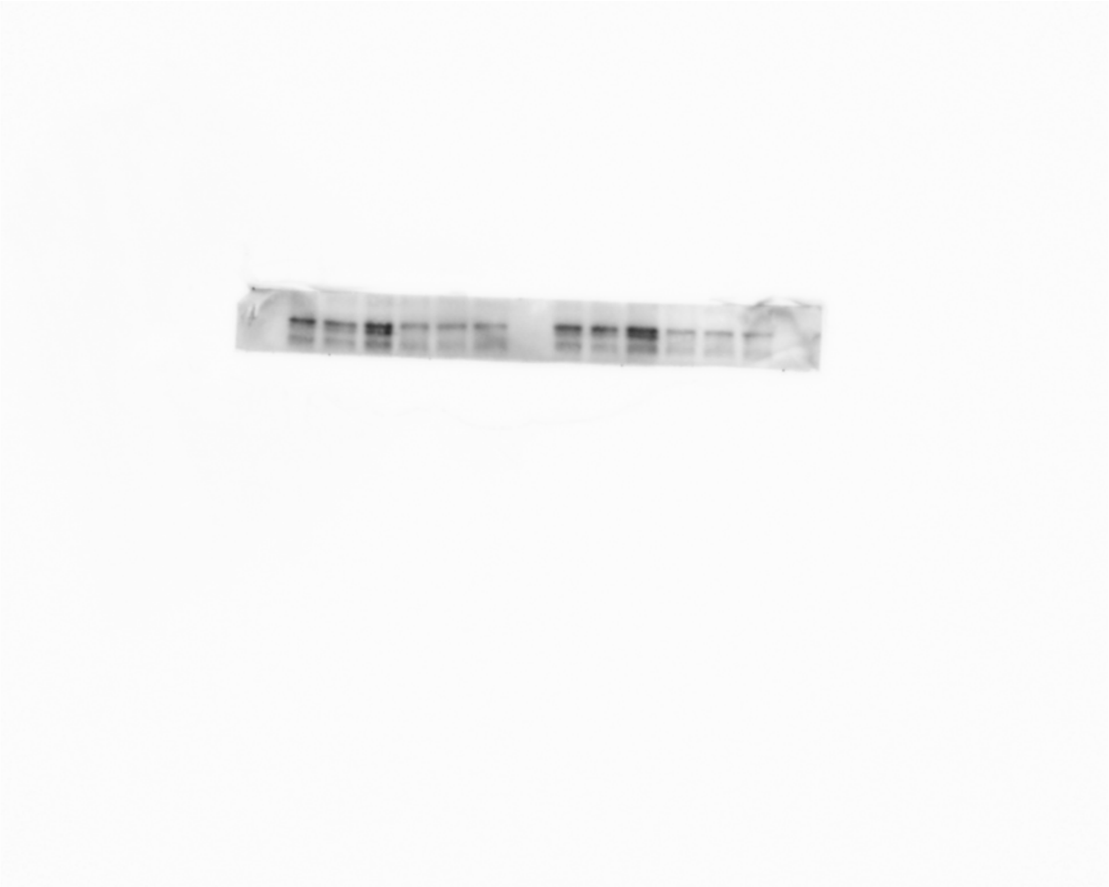

FKBP10

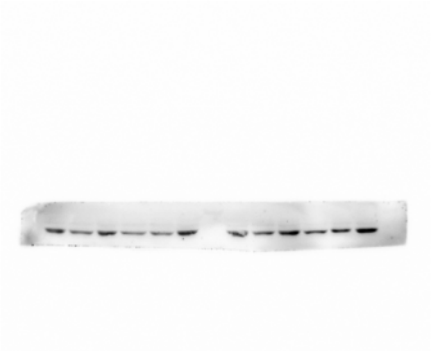

FGFR1

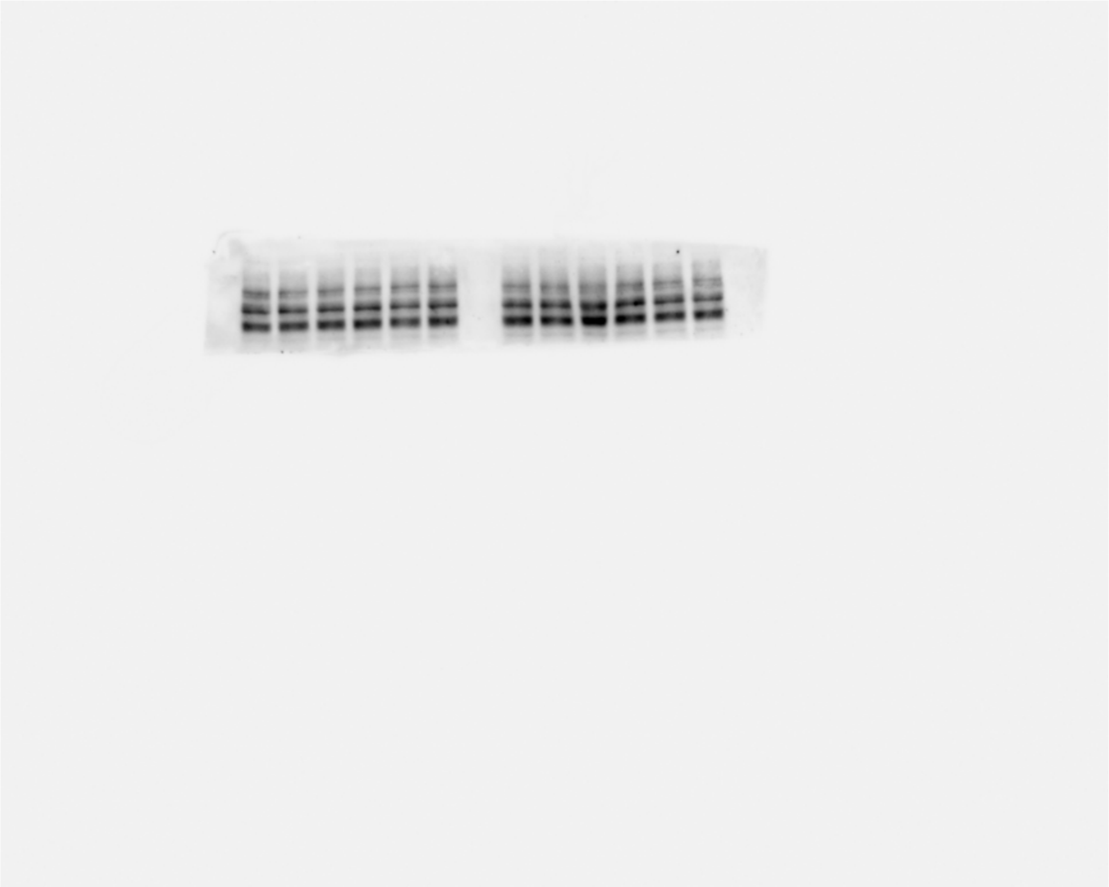

p-FGFR1

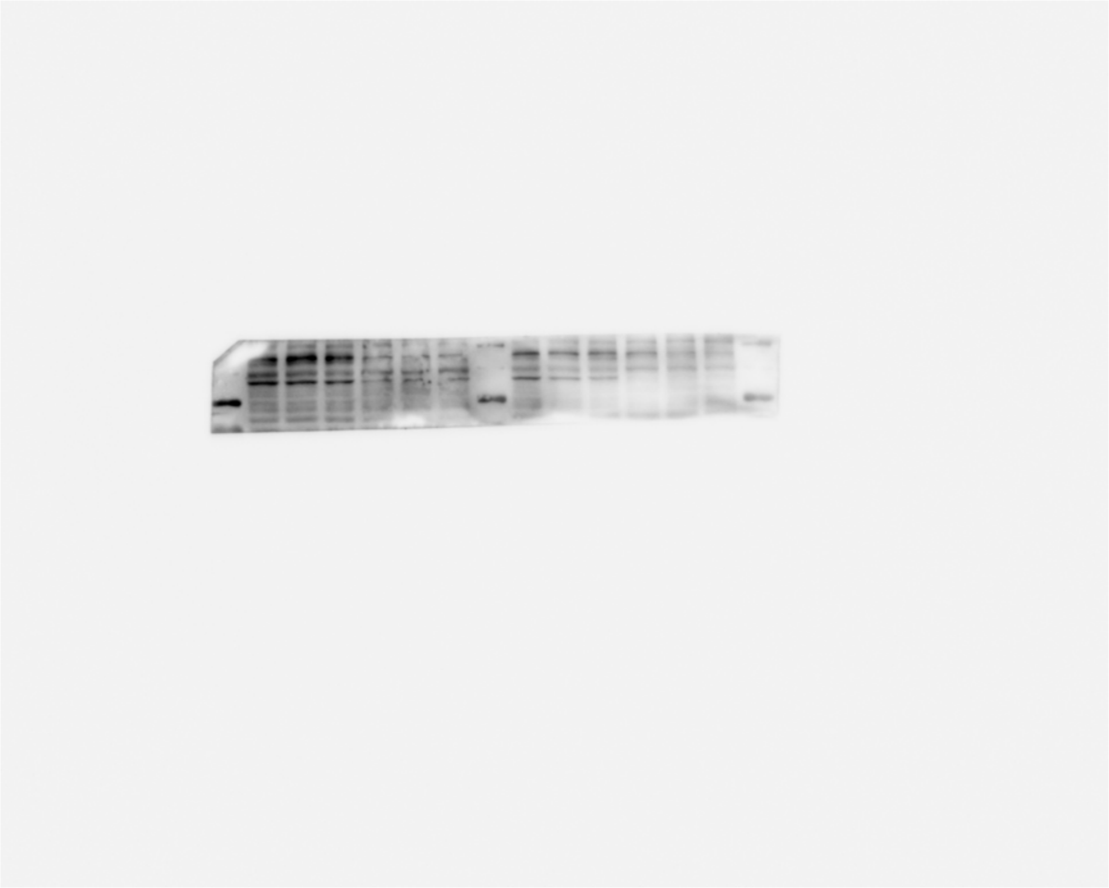

Fig. 4I

FGFR1, LDHA

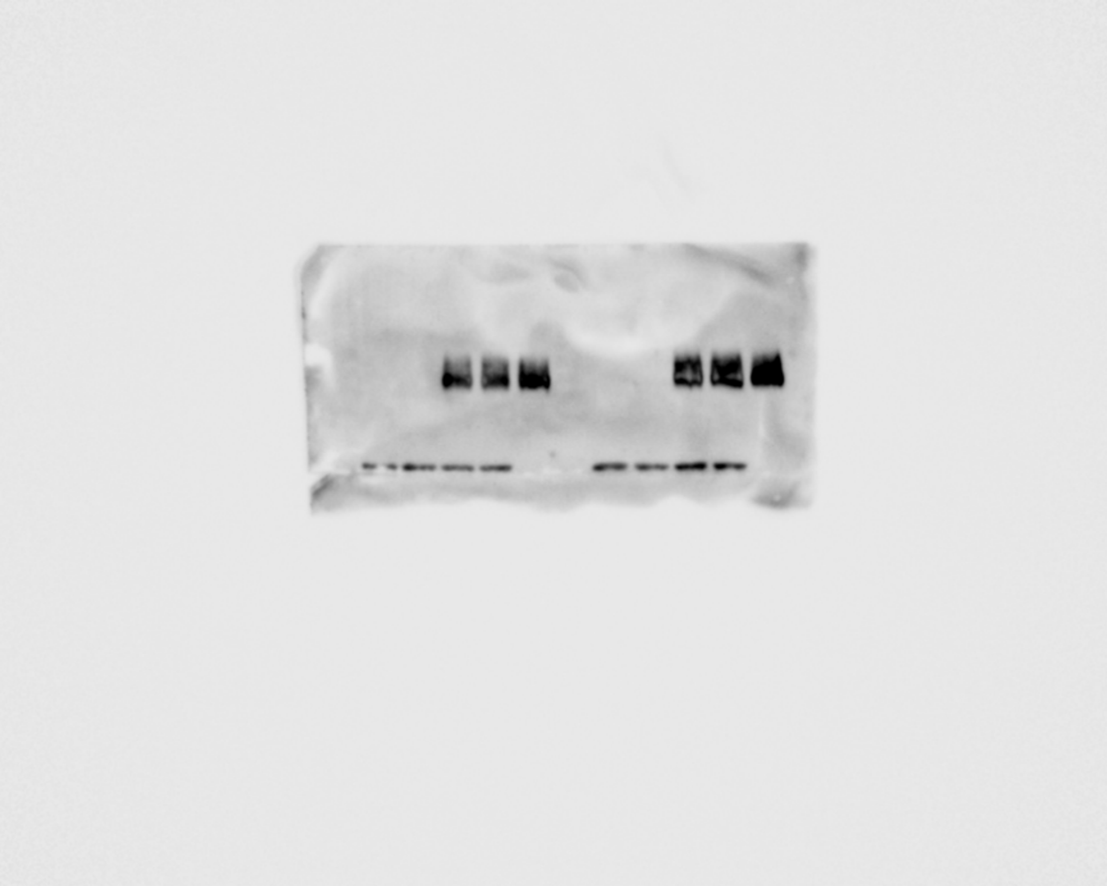

pLDHA

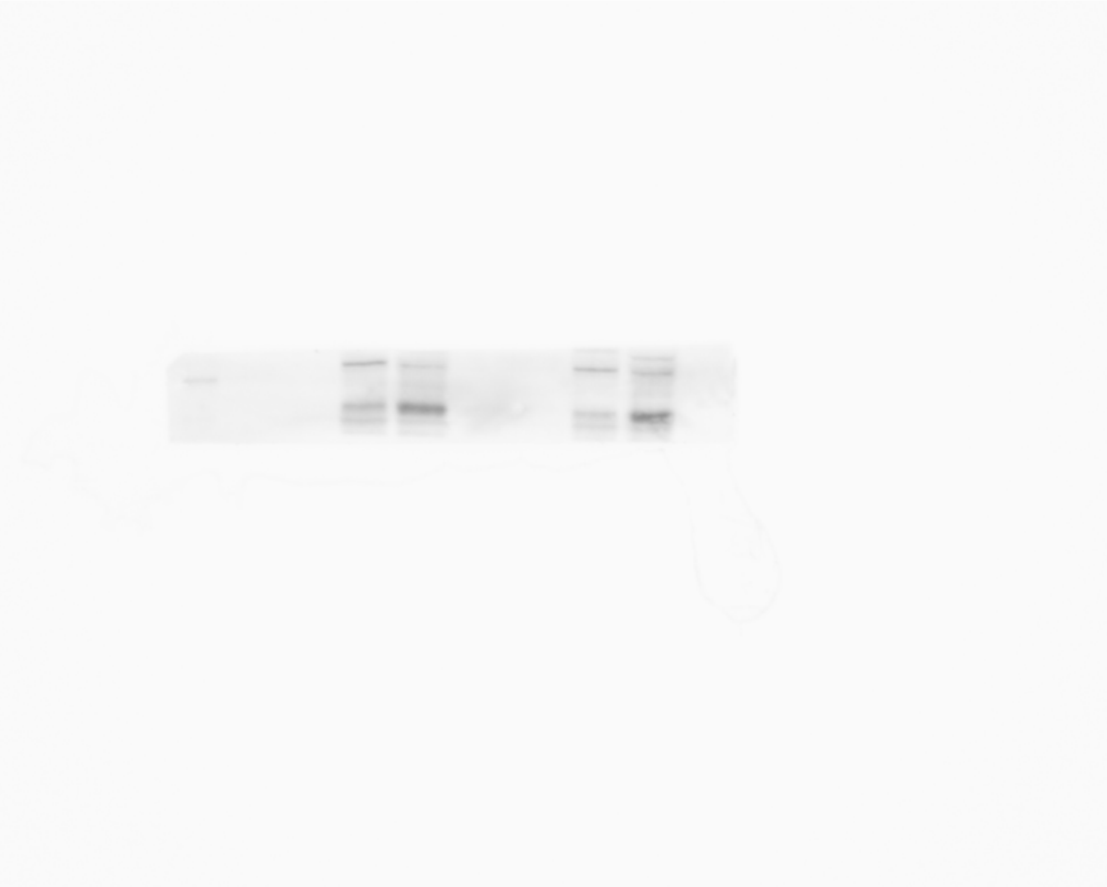

Flag

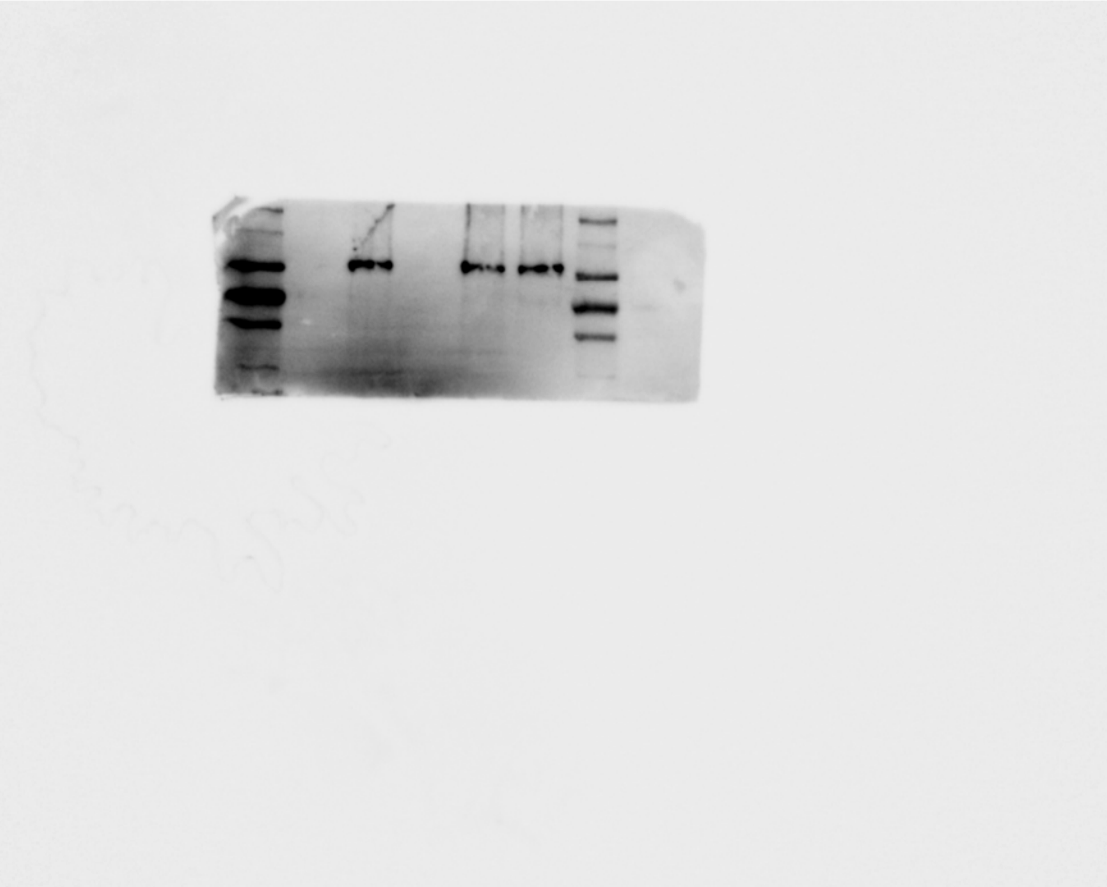

Fig. 5A

Actin

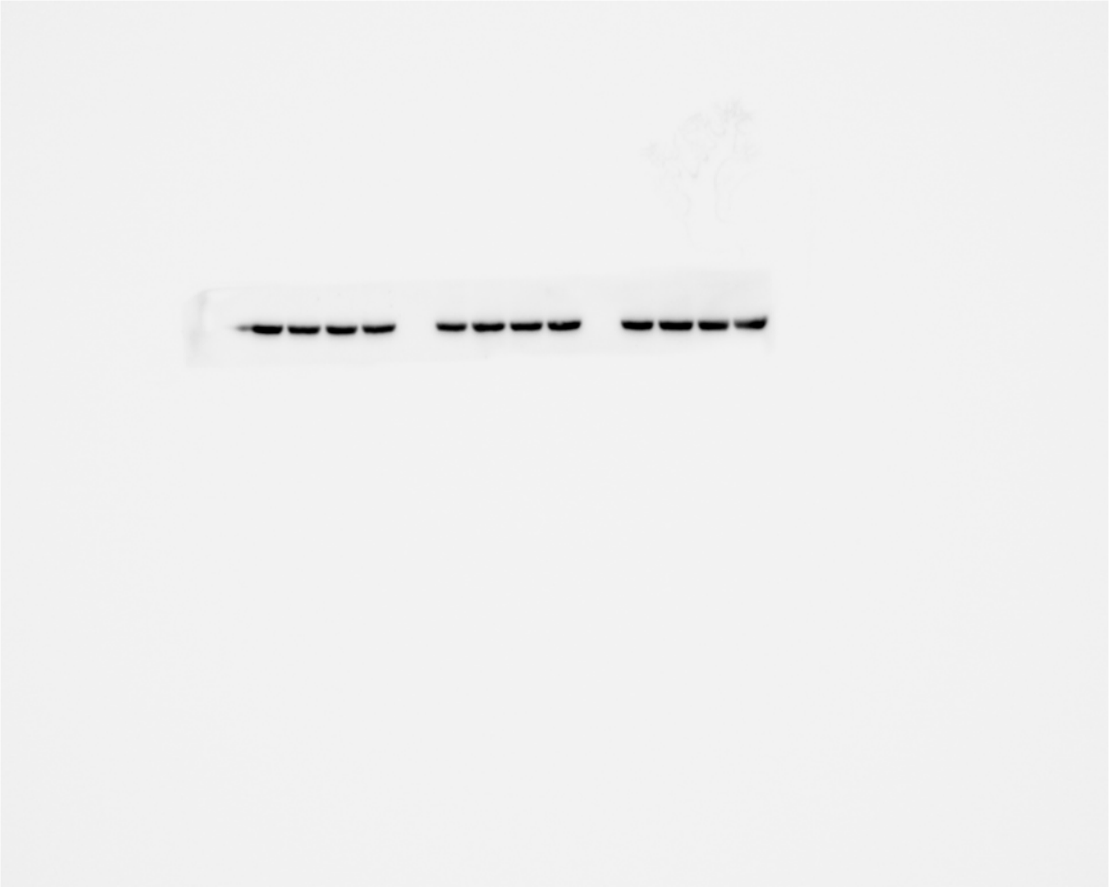

FKBP10

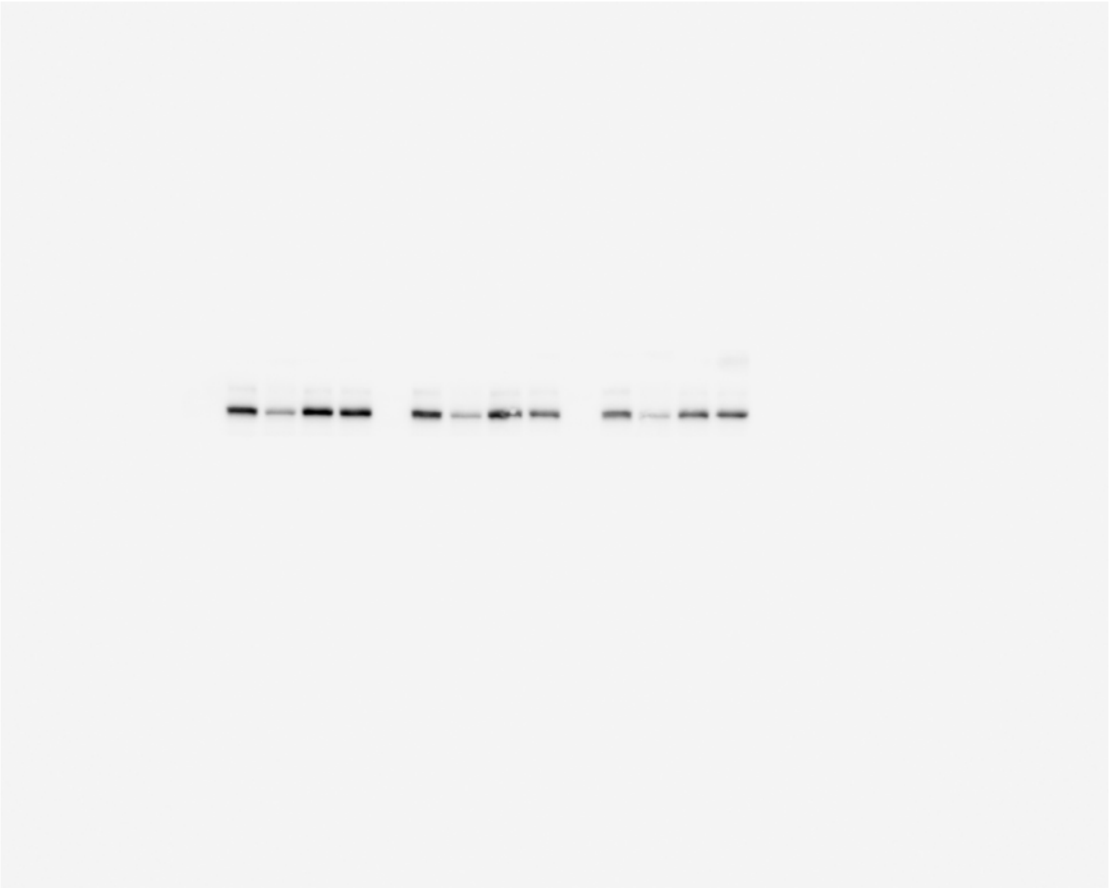

LDHA

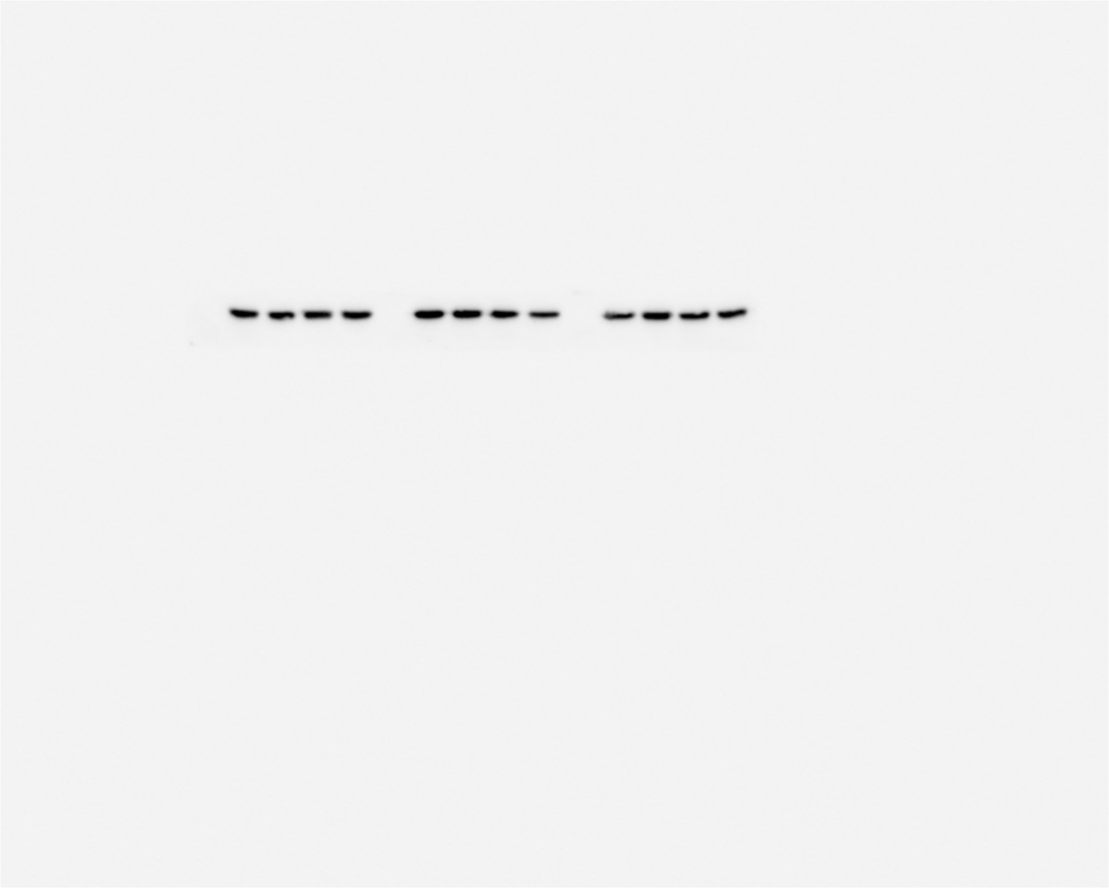

pLDHA

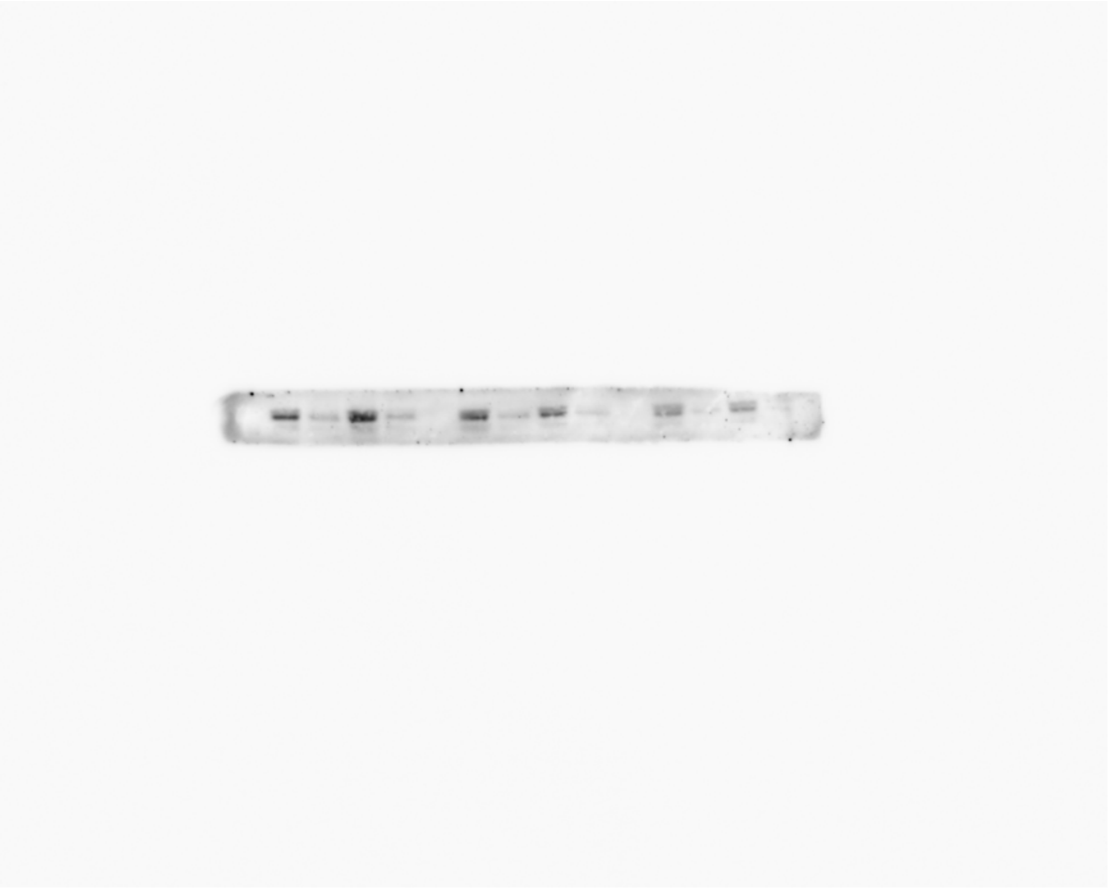

Flag

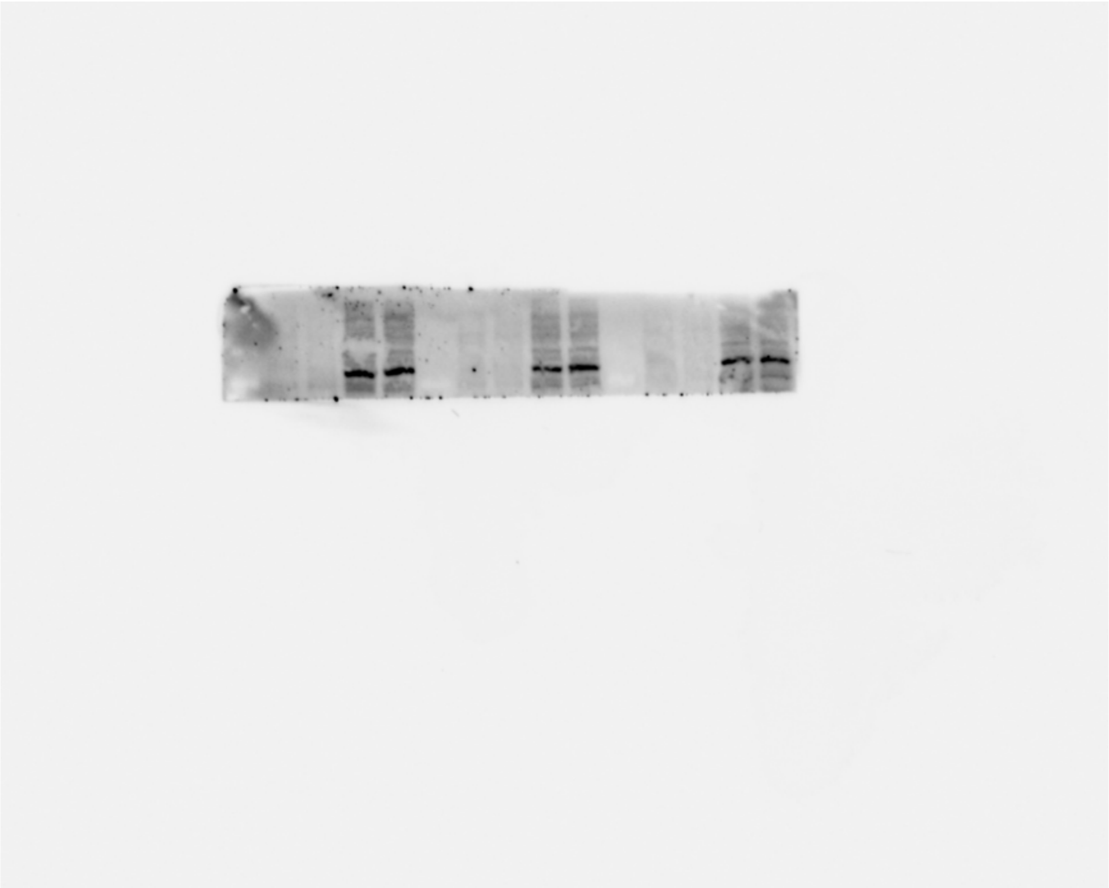

Fig. 5B
Actin

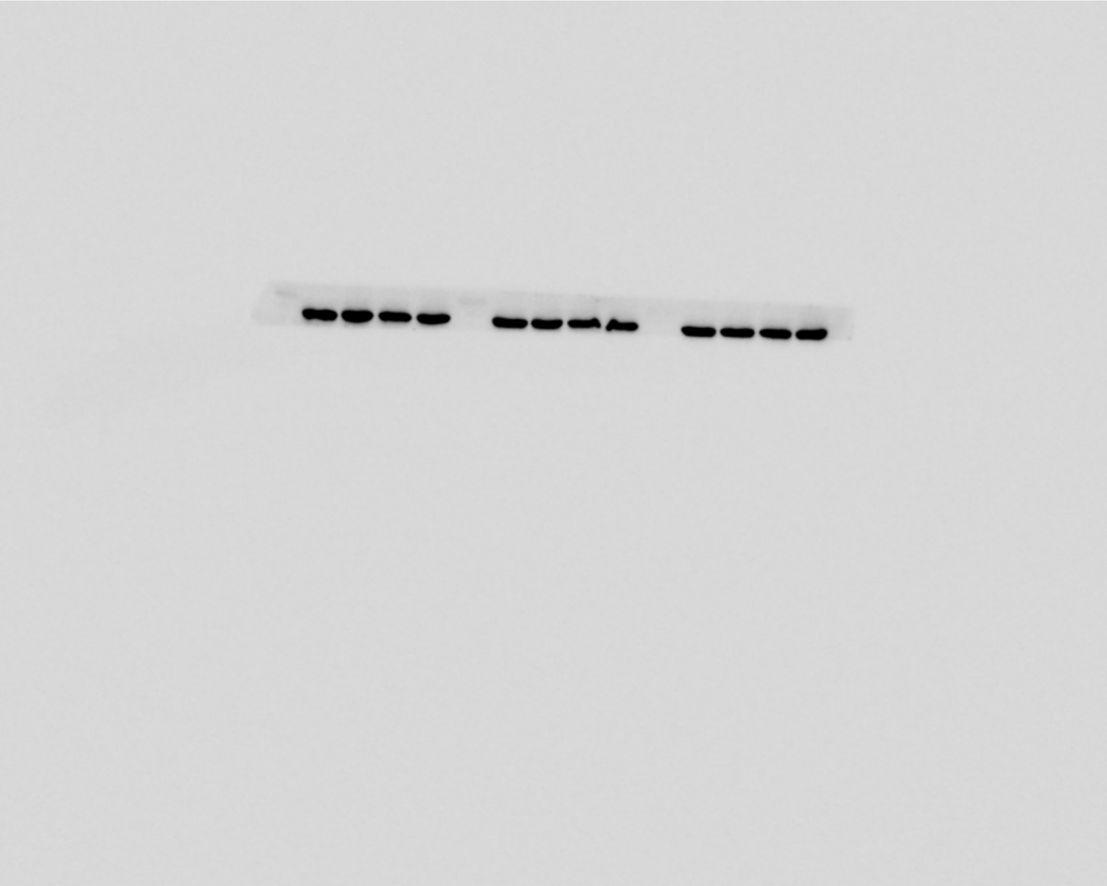

His

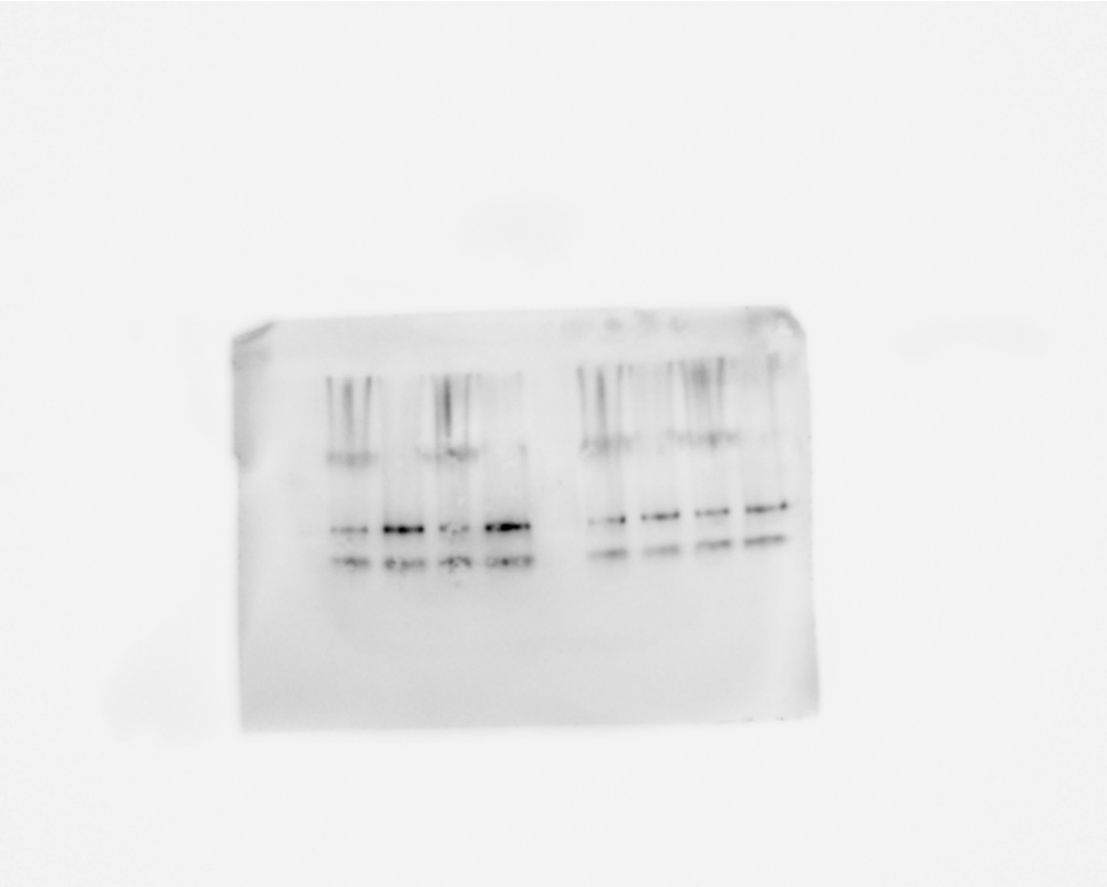

Fig. 6A

Actin

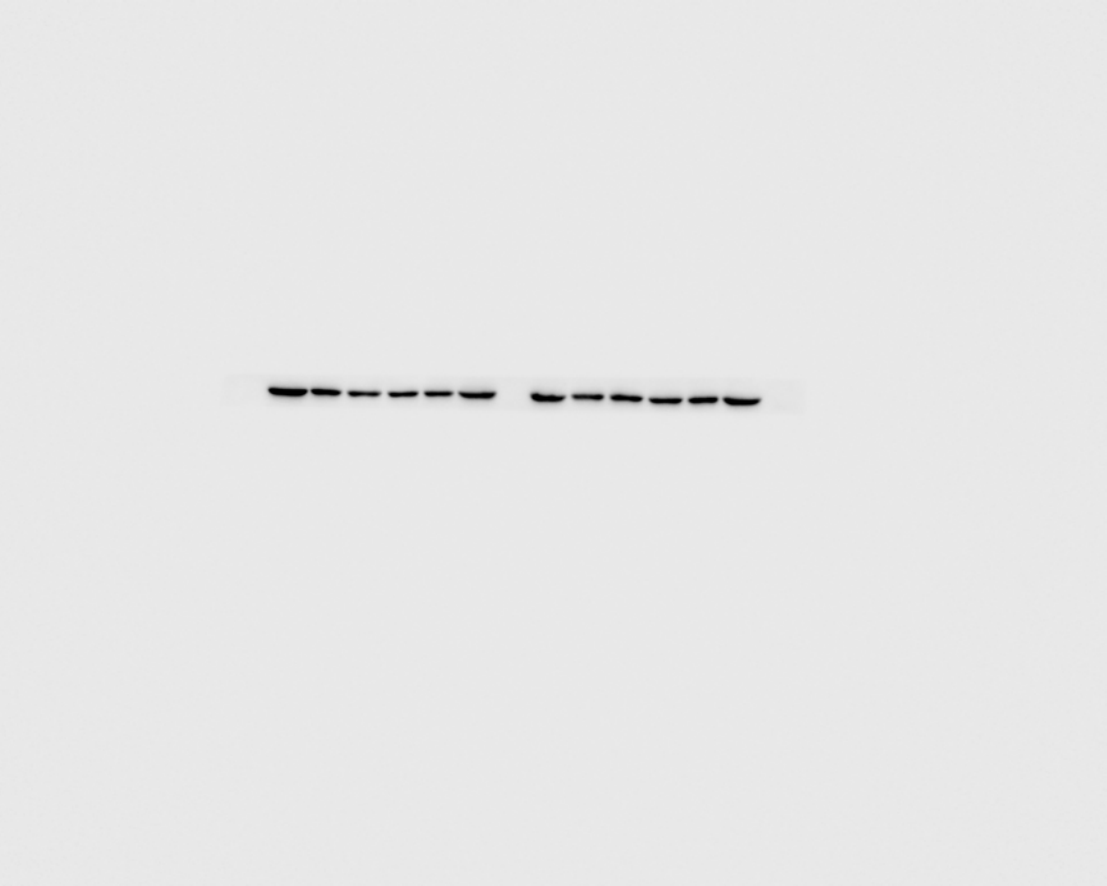

His

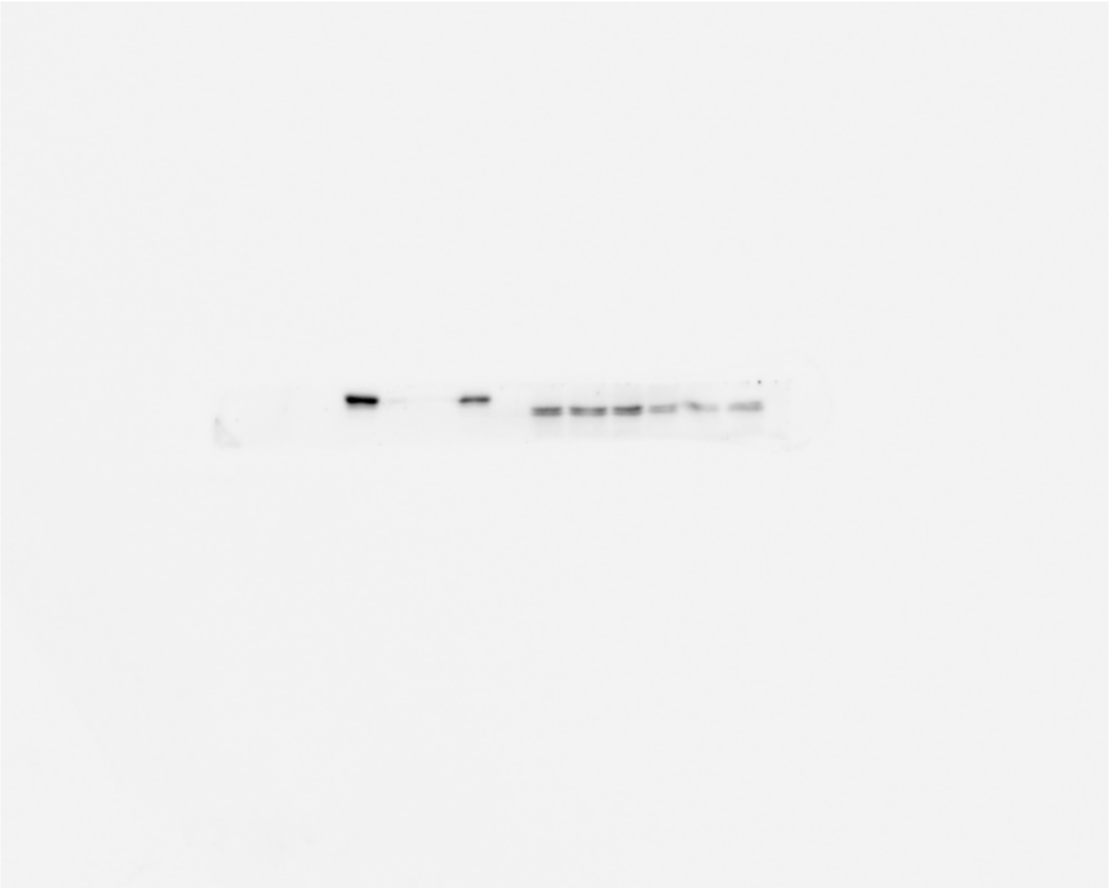

LDHA

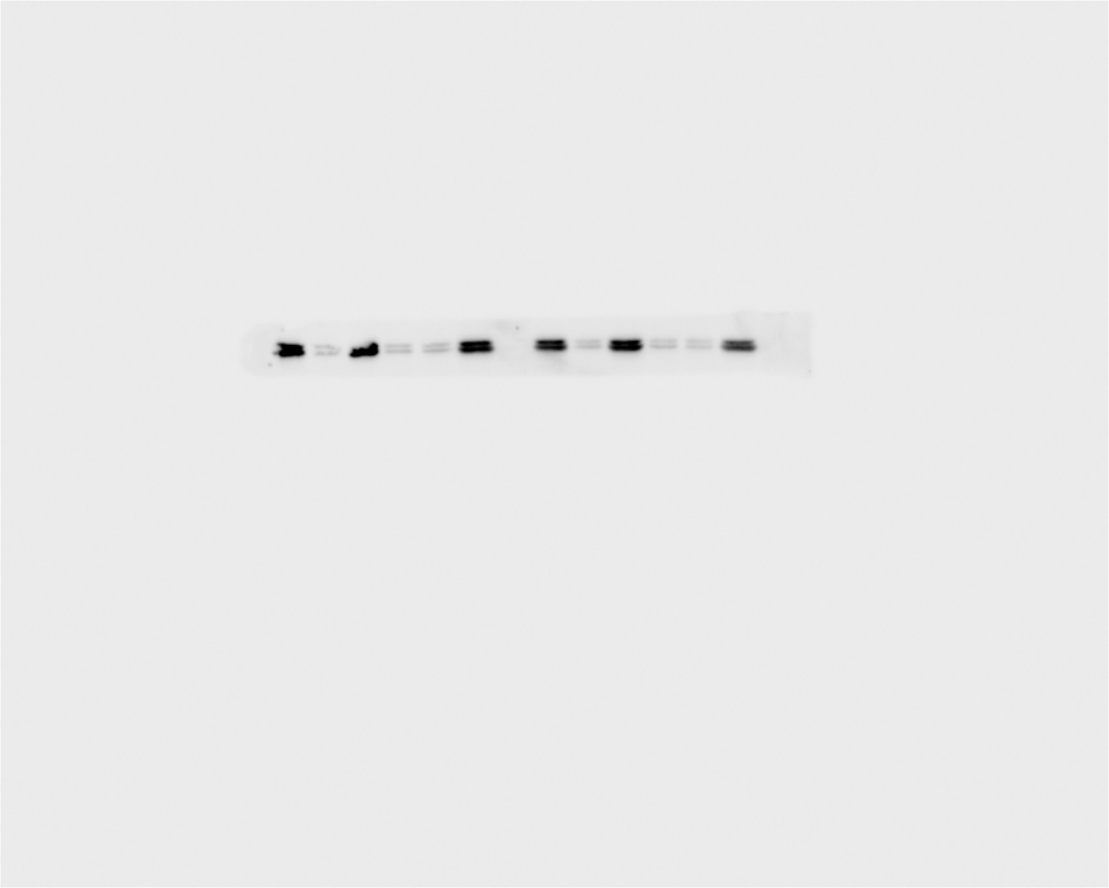

pLDHA

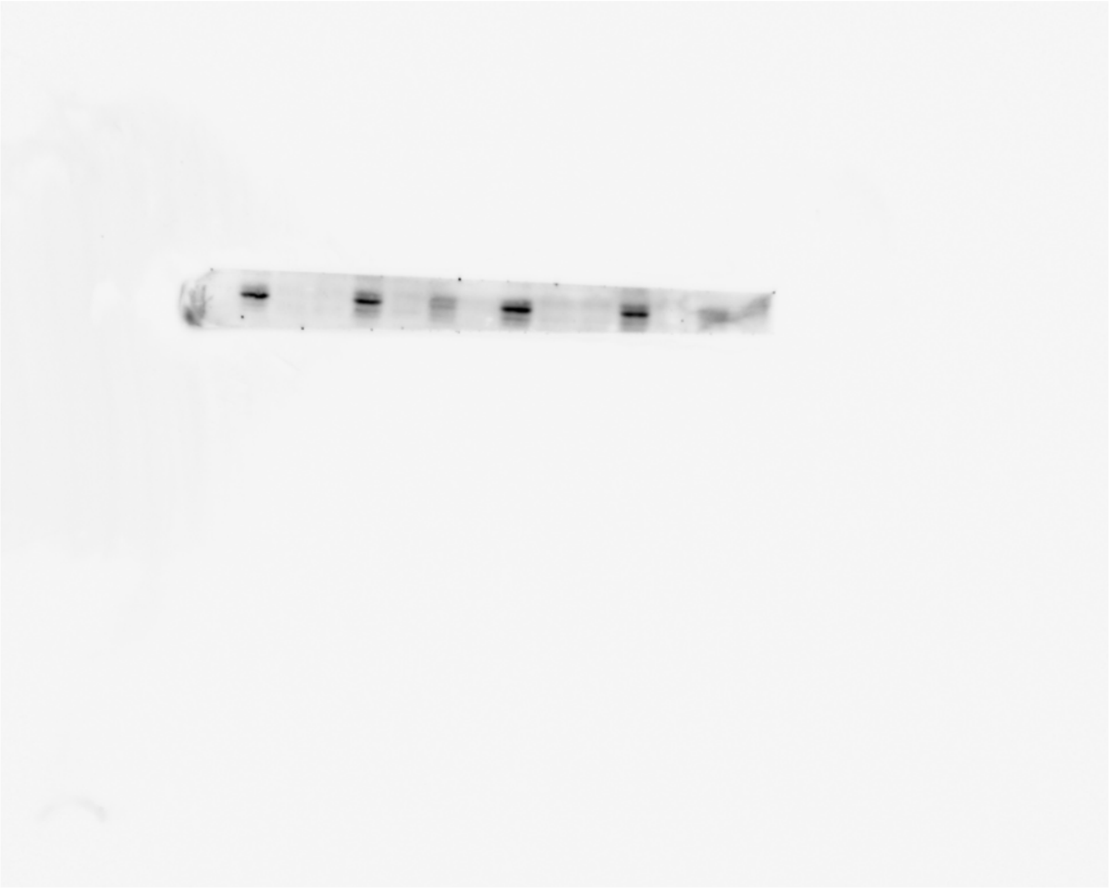

Flag

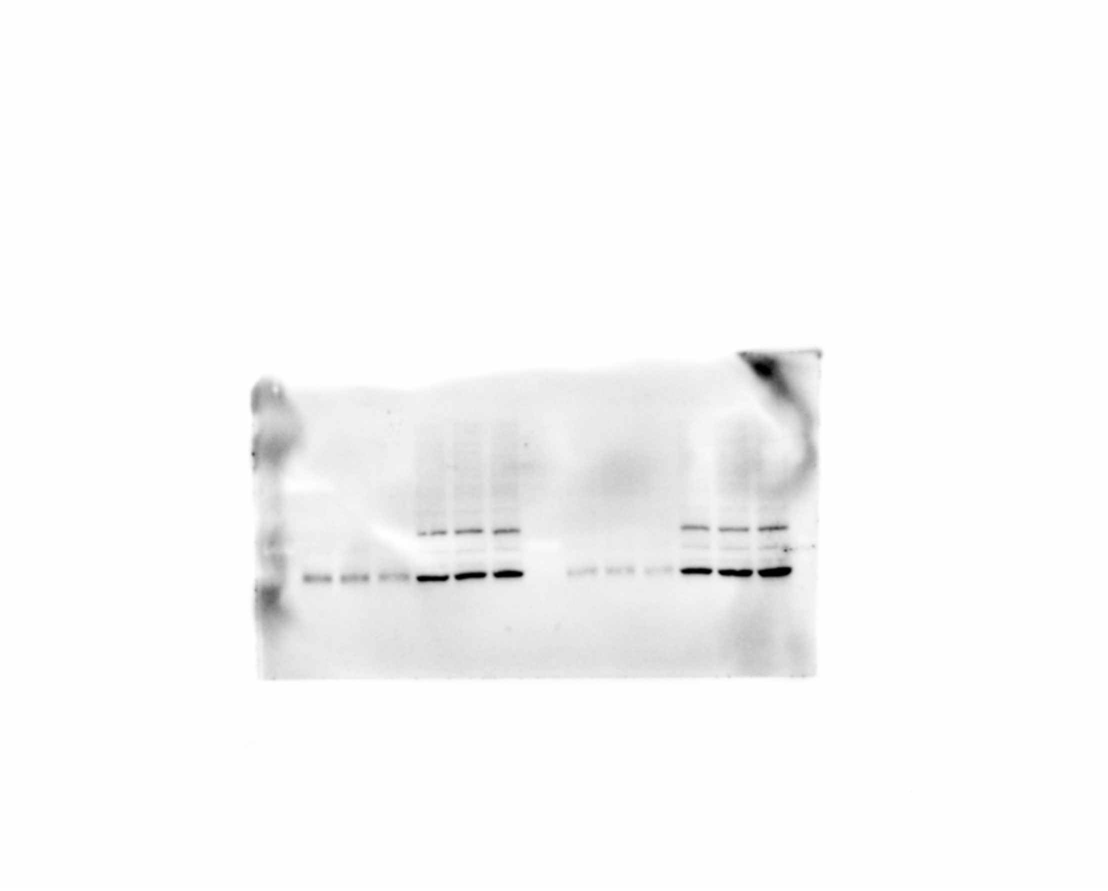

FKBP10

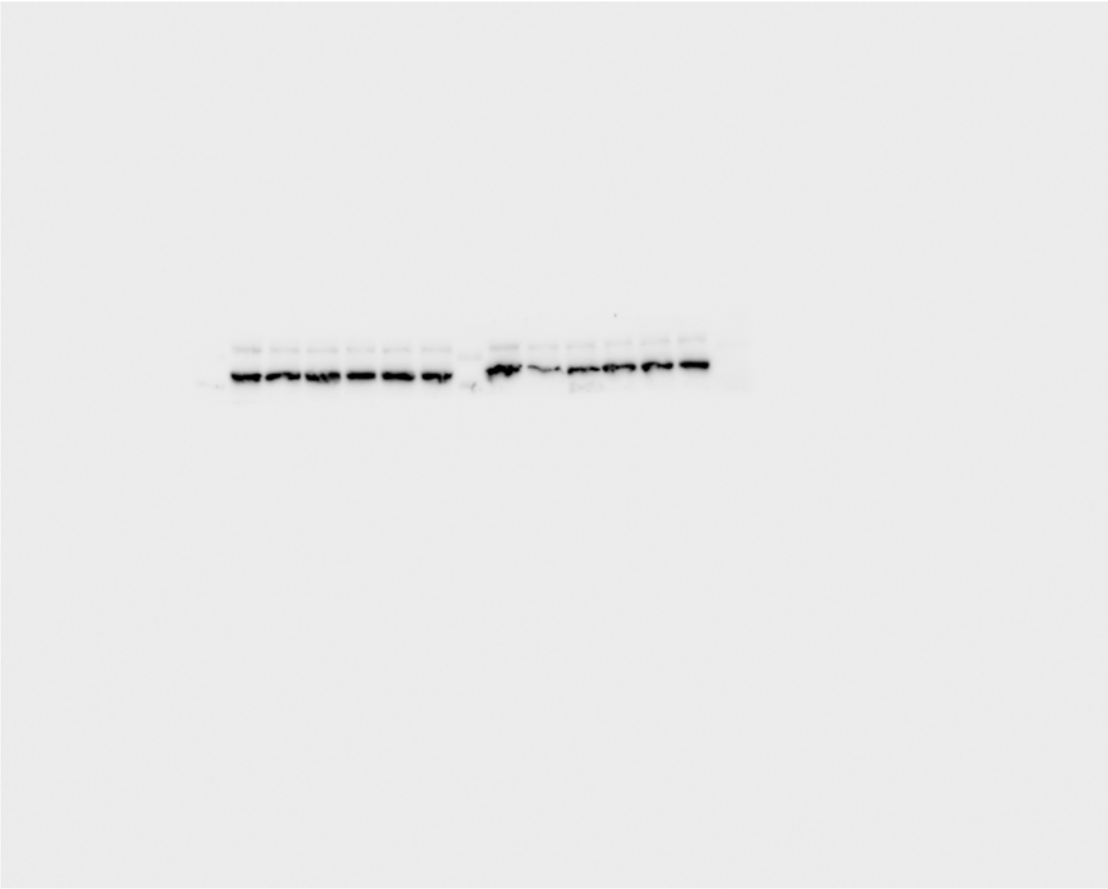

FGFR1

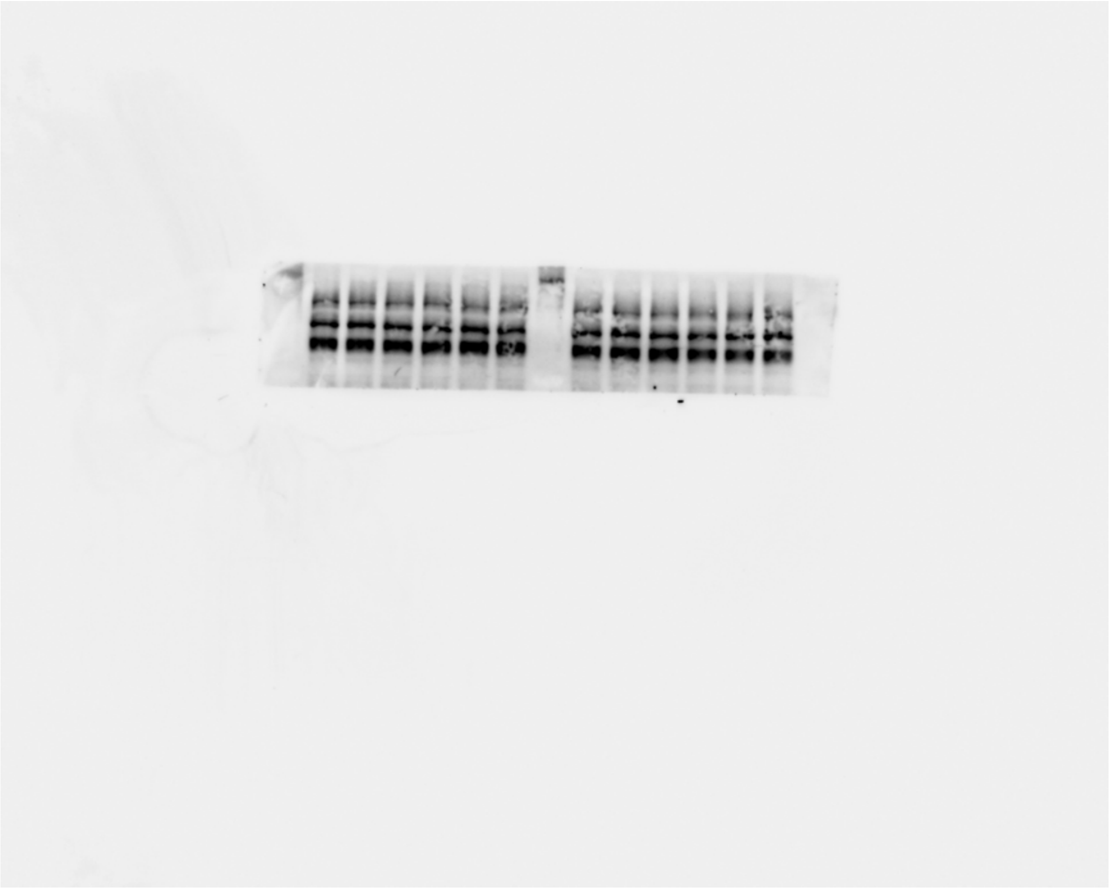

Fig. 6H

Actin

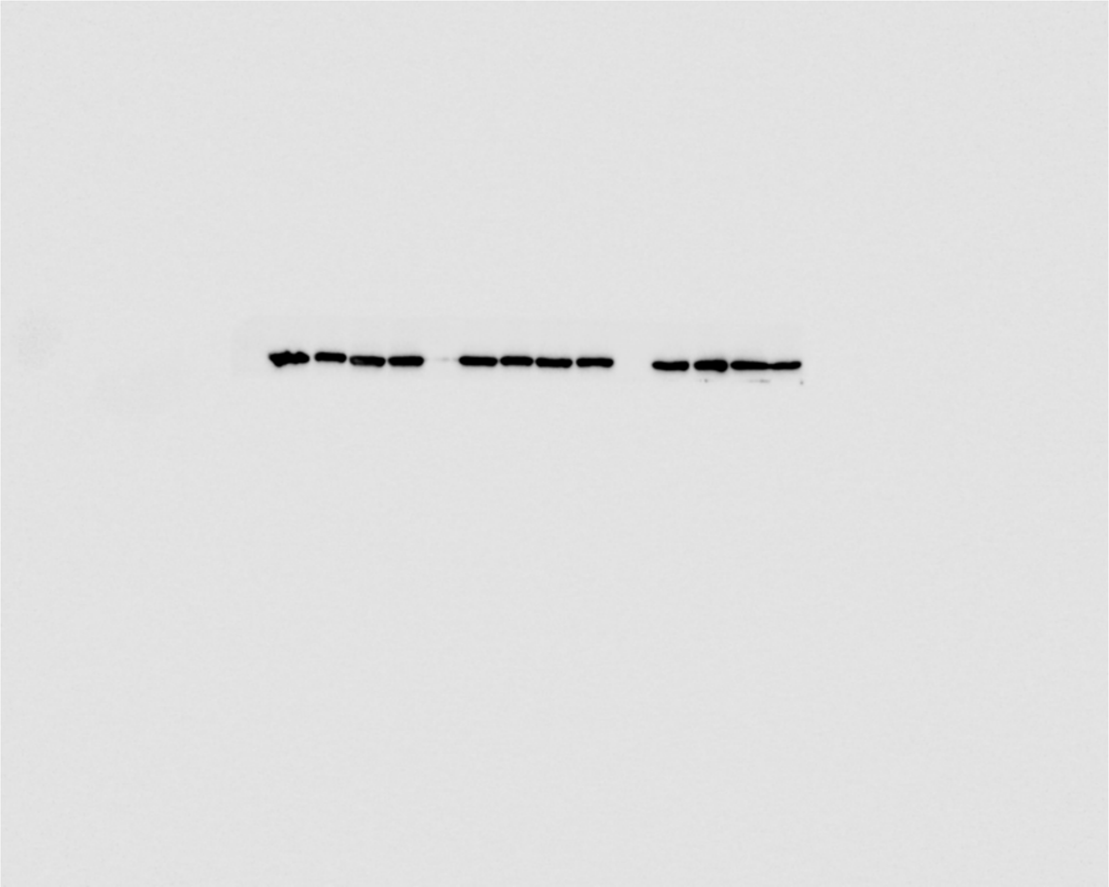

Pan Kla

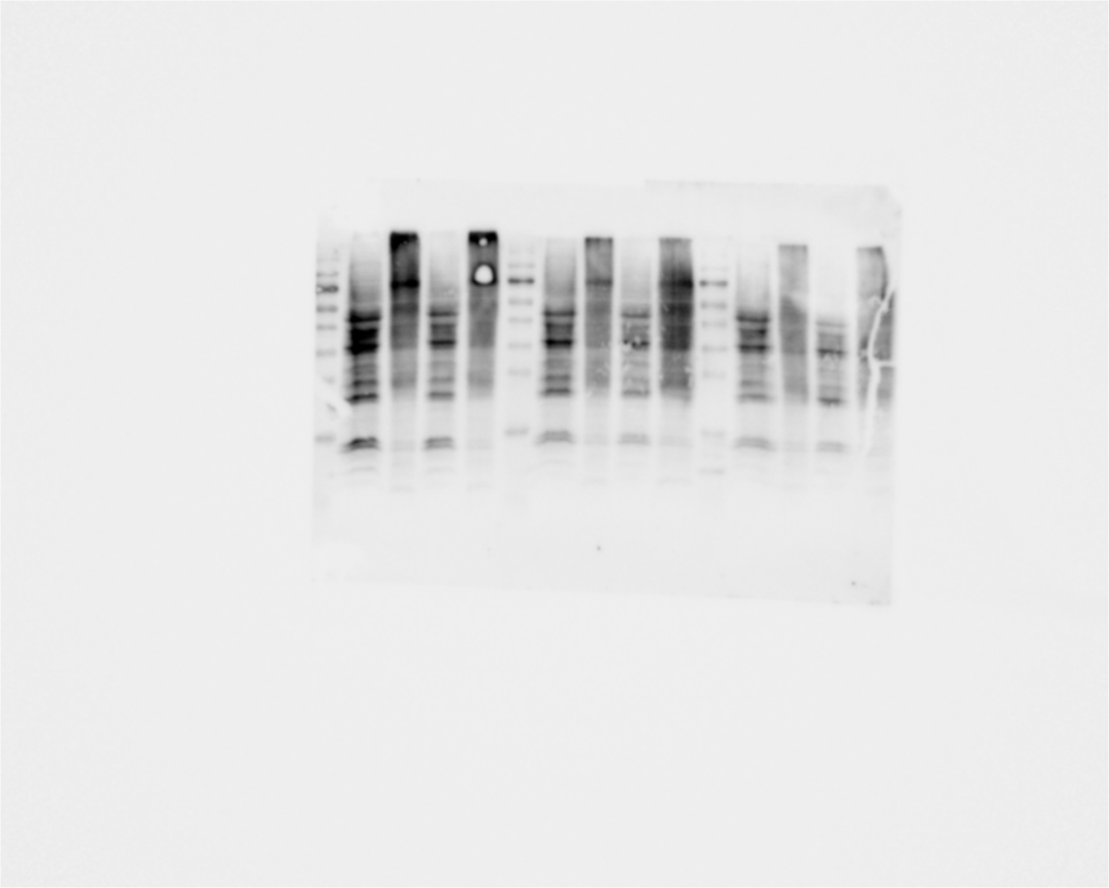

Fig. 6I

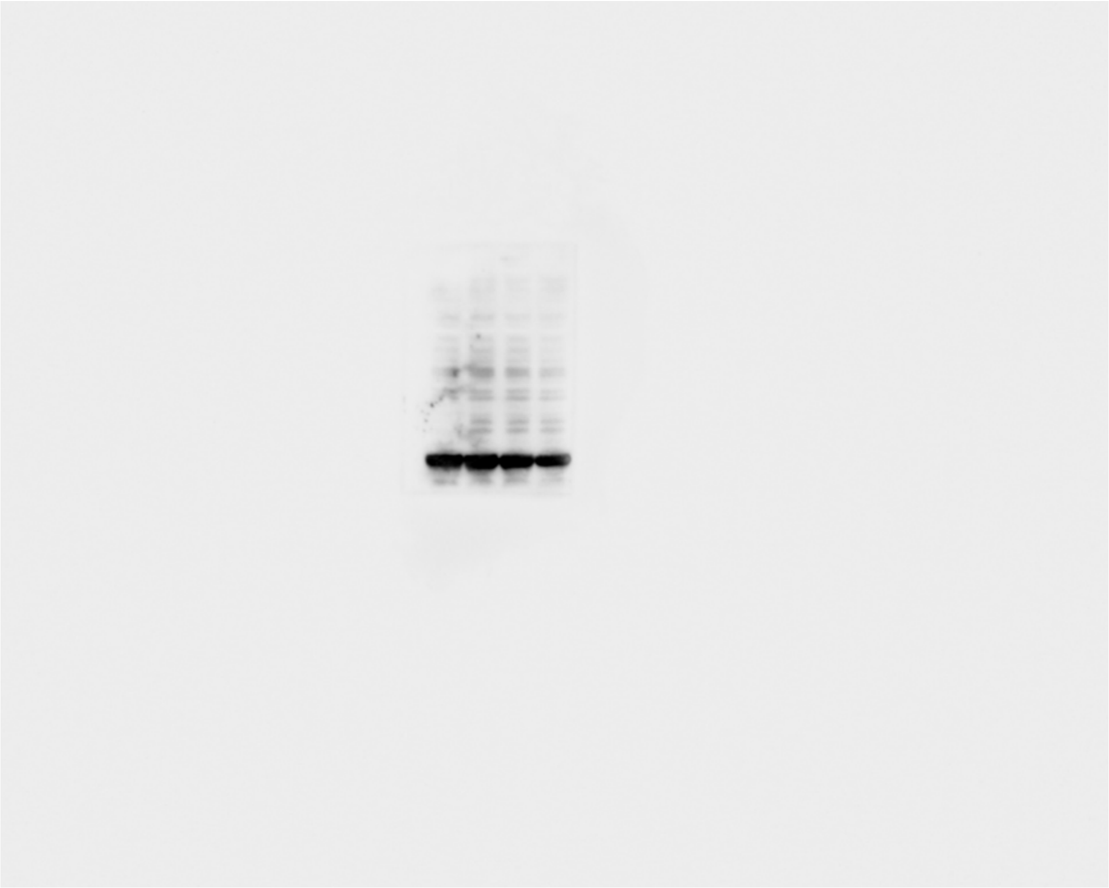

H3K56la

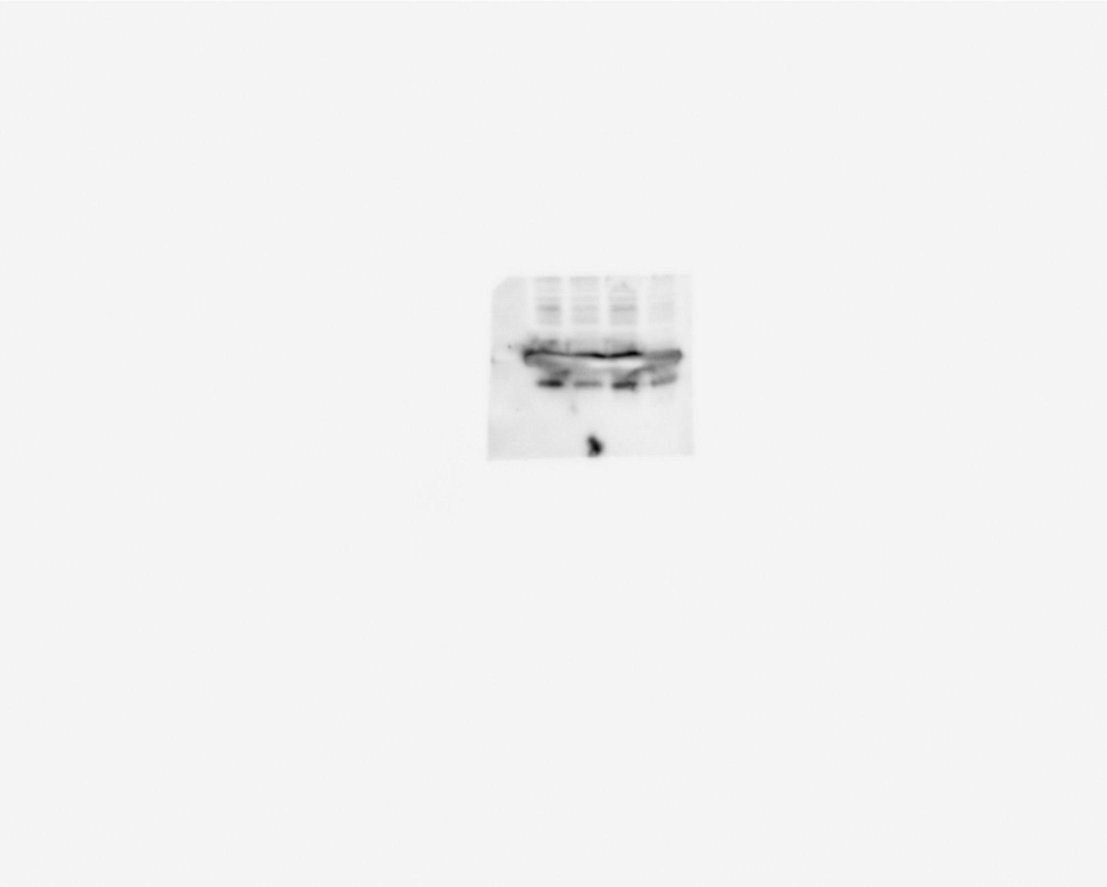

H3K18la

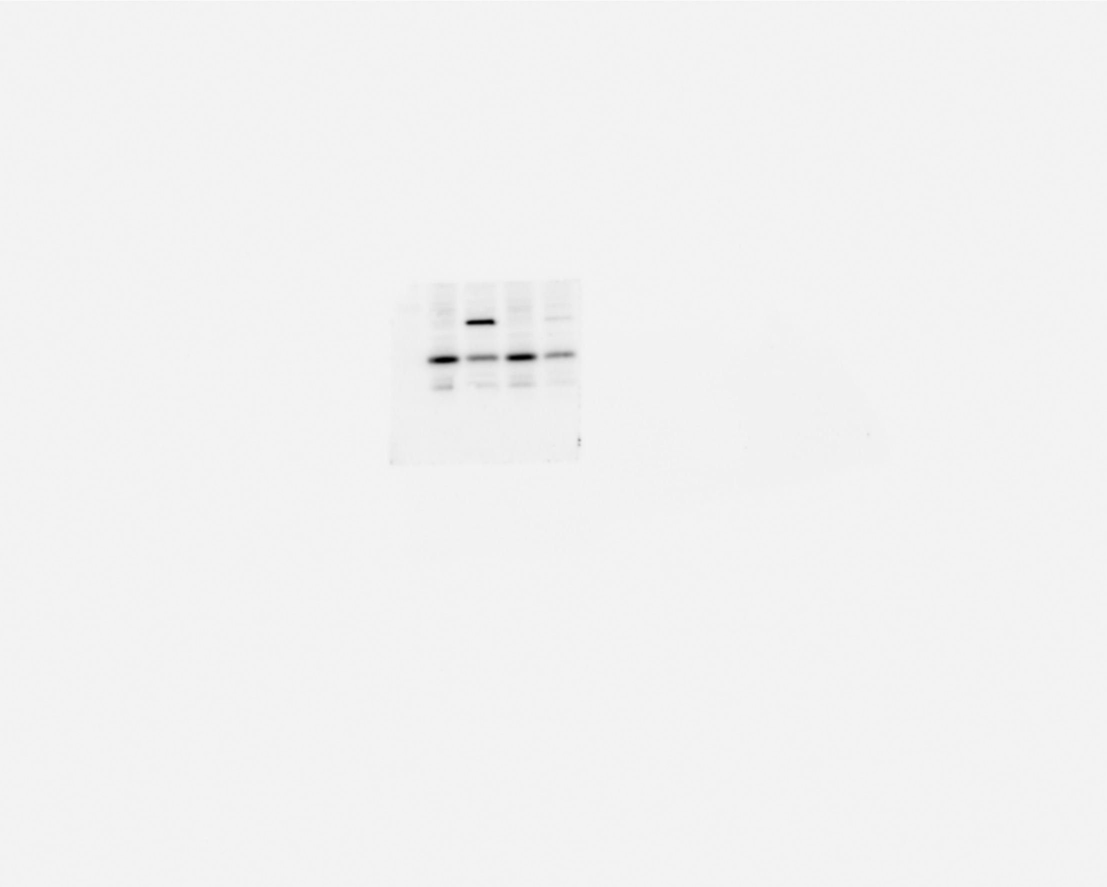

H3K14la

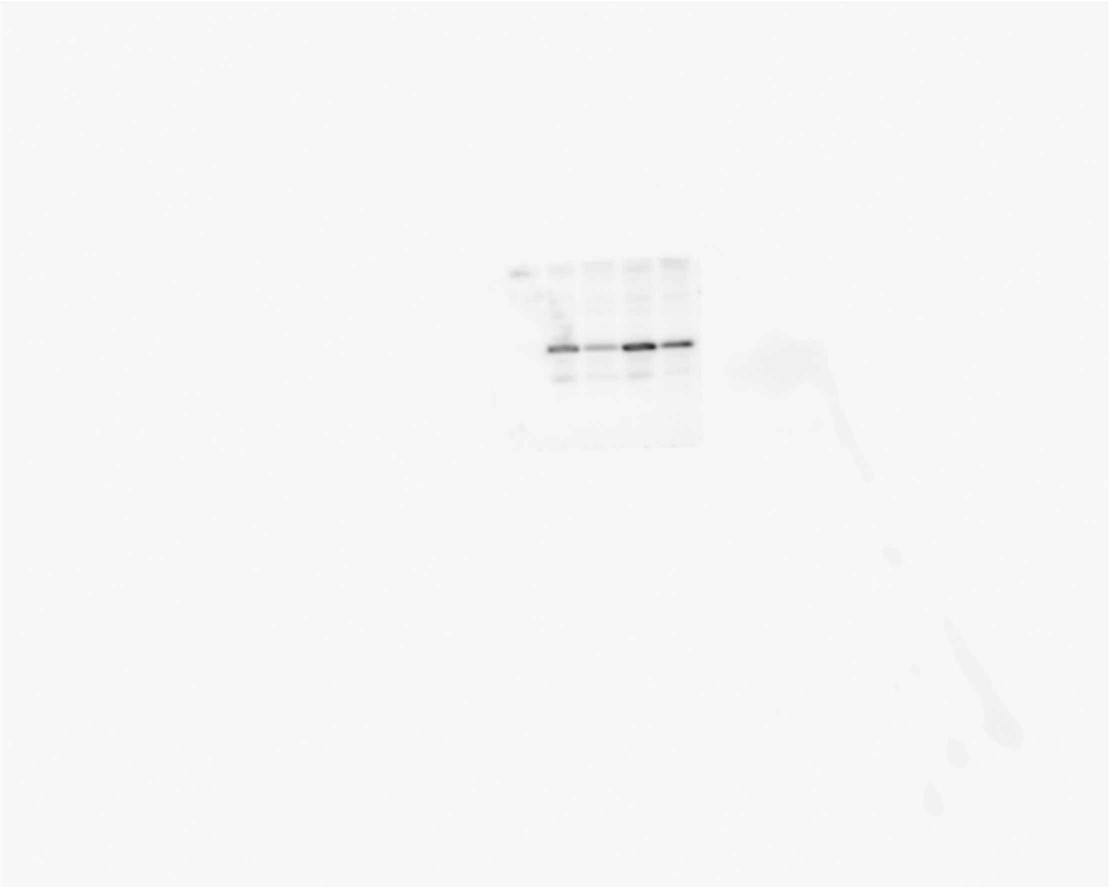

Pan kla

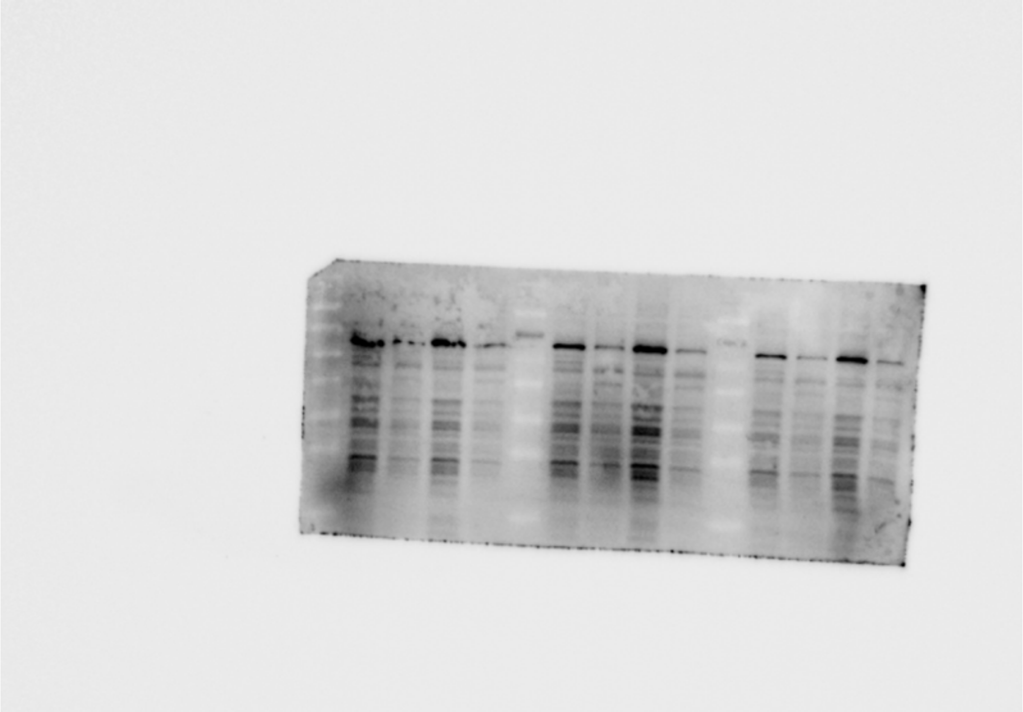

Fig. 7A

Actin

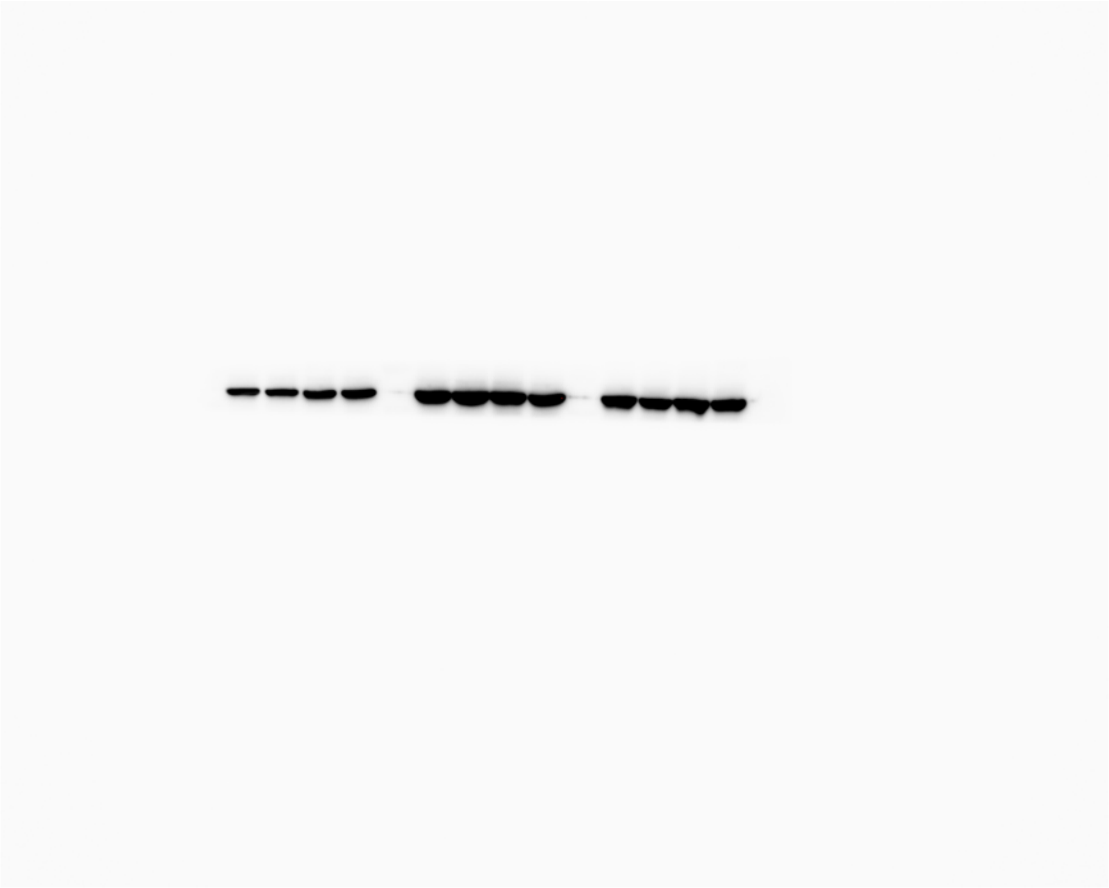

Actin-RCC4

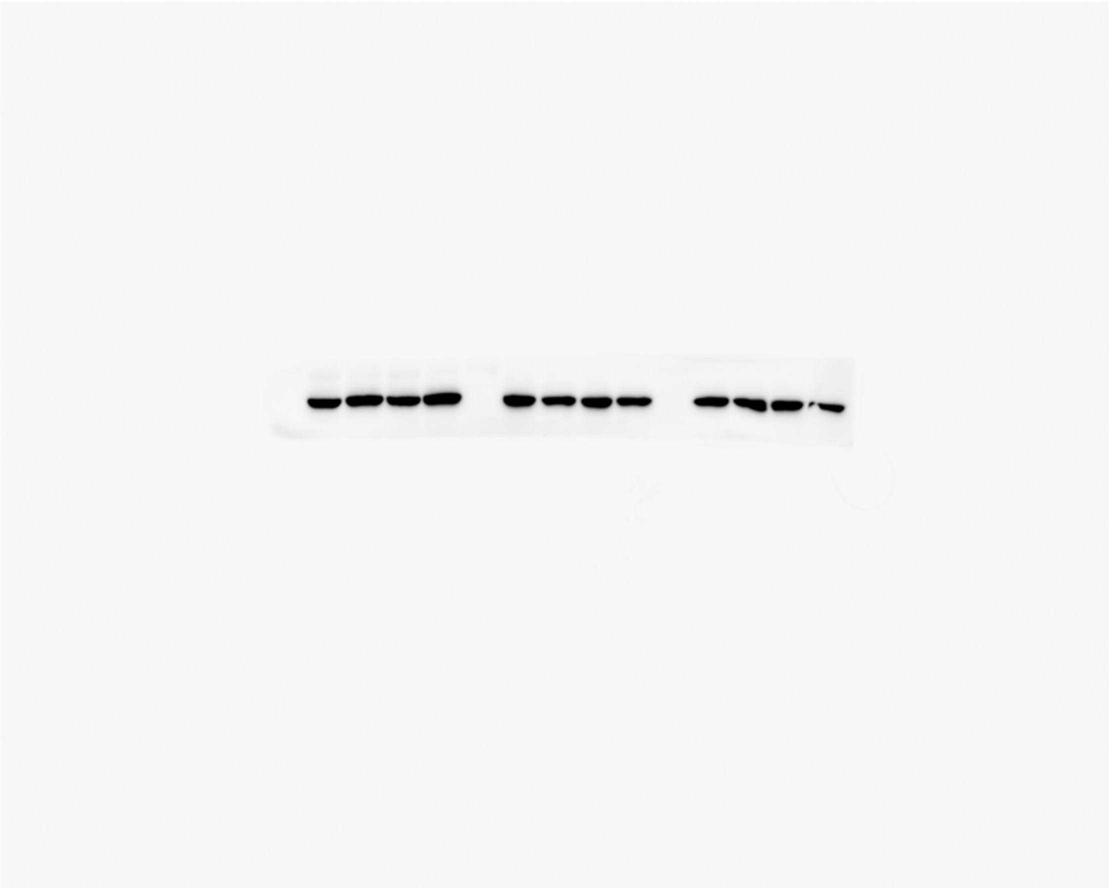

HIF1a-RCC4

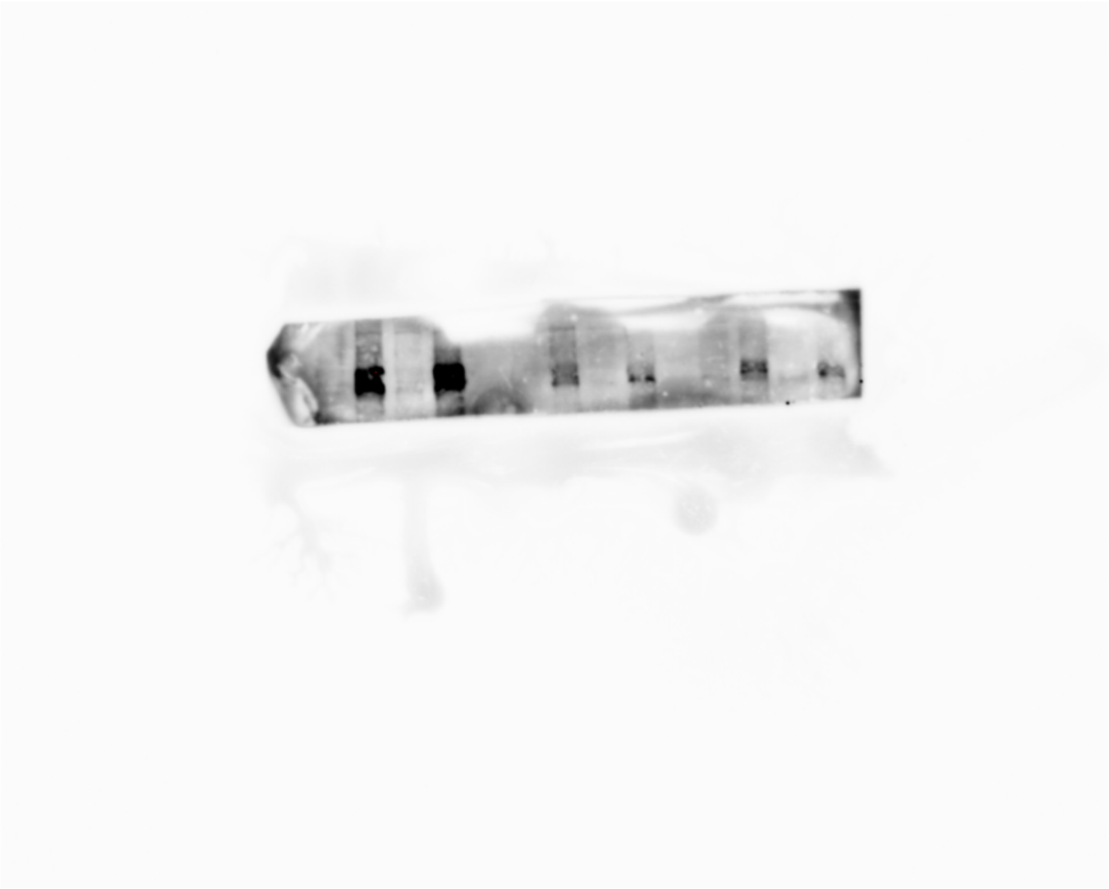

HIF1a-caki1

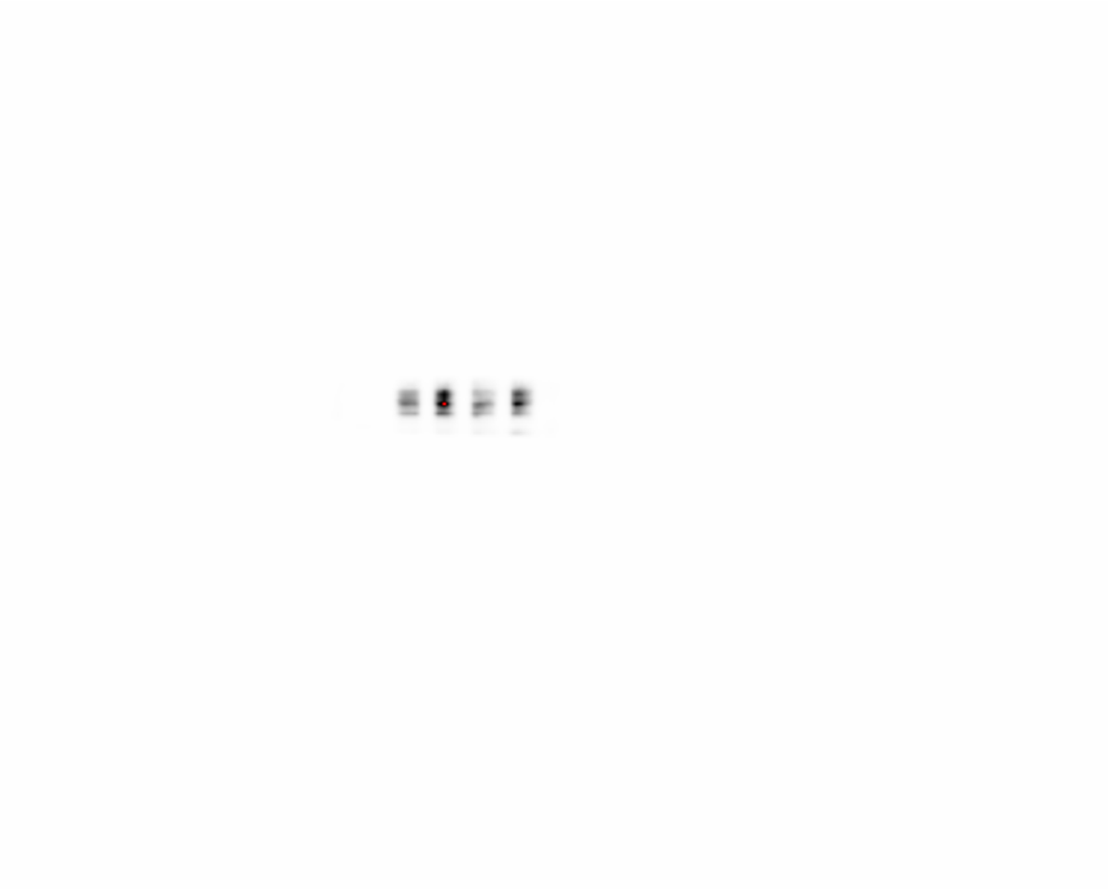

HIF1a-ACHN HK2

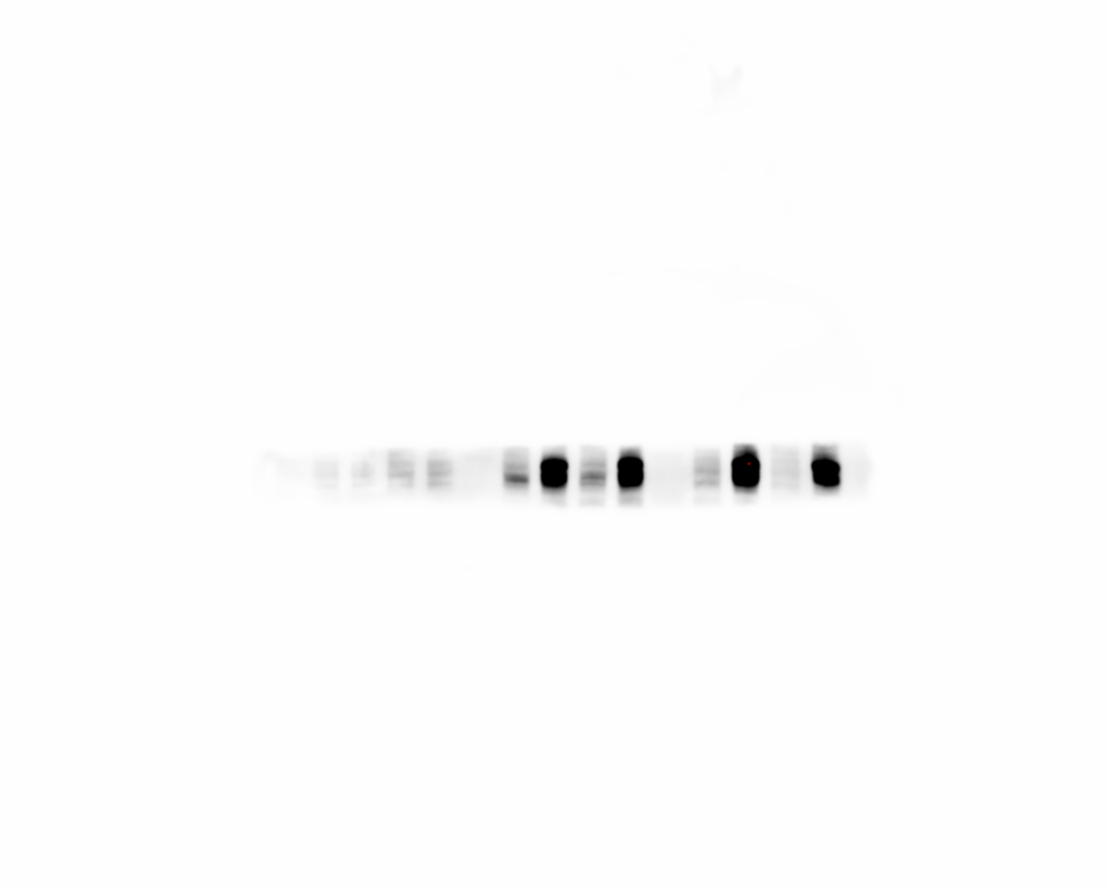

HIF2a-caki1 ACHN HK2

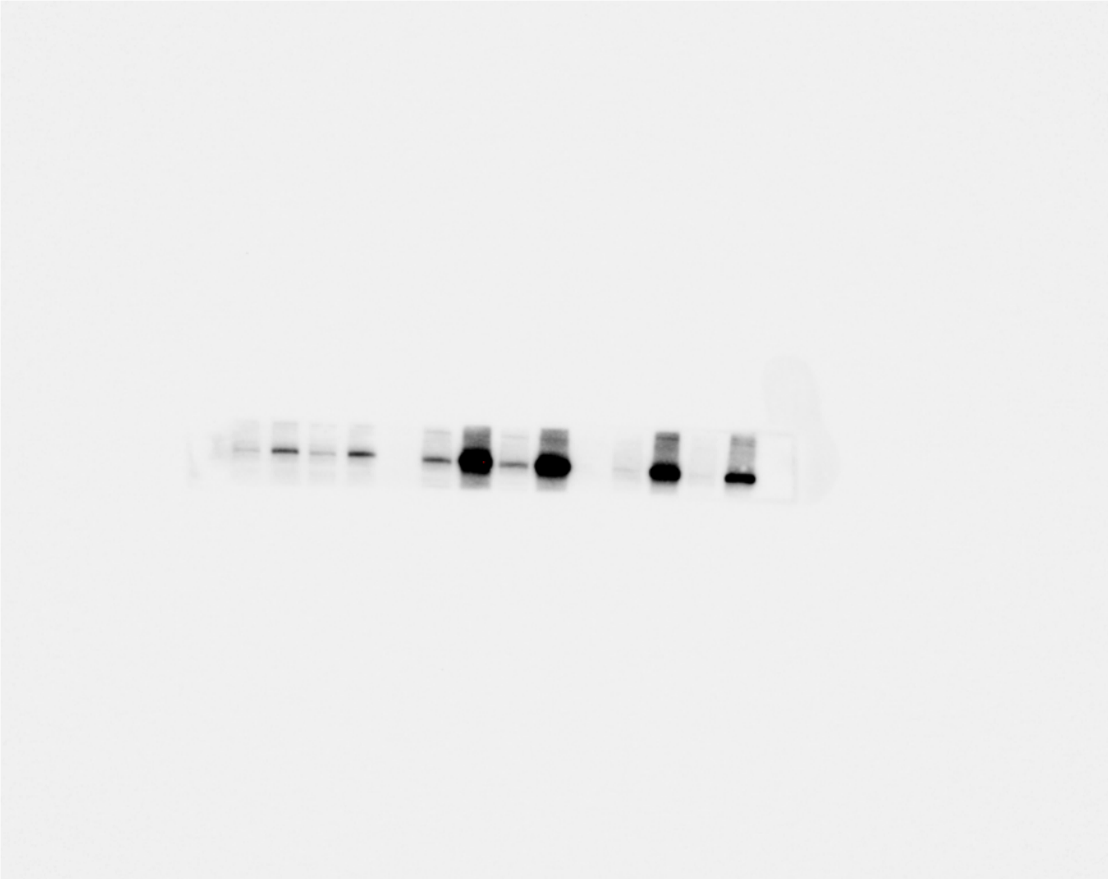

HIF2a-RCC4

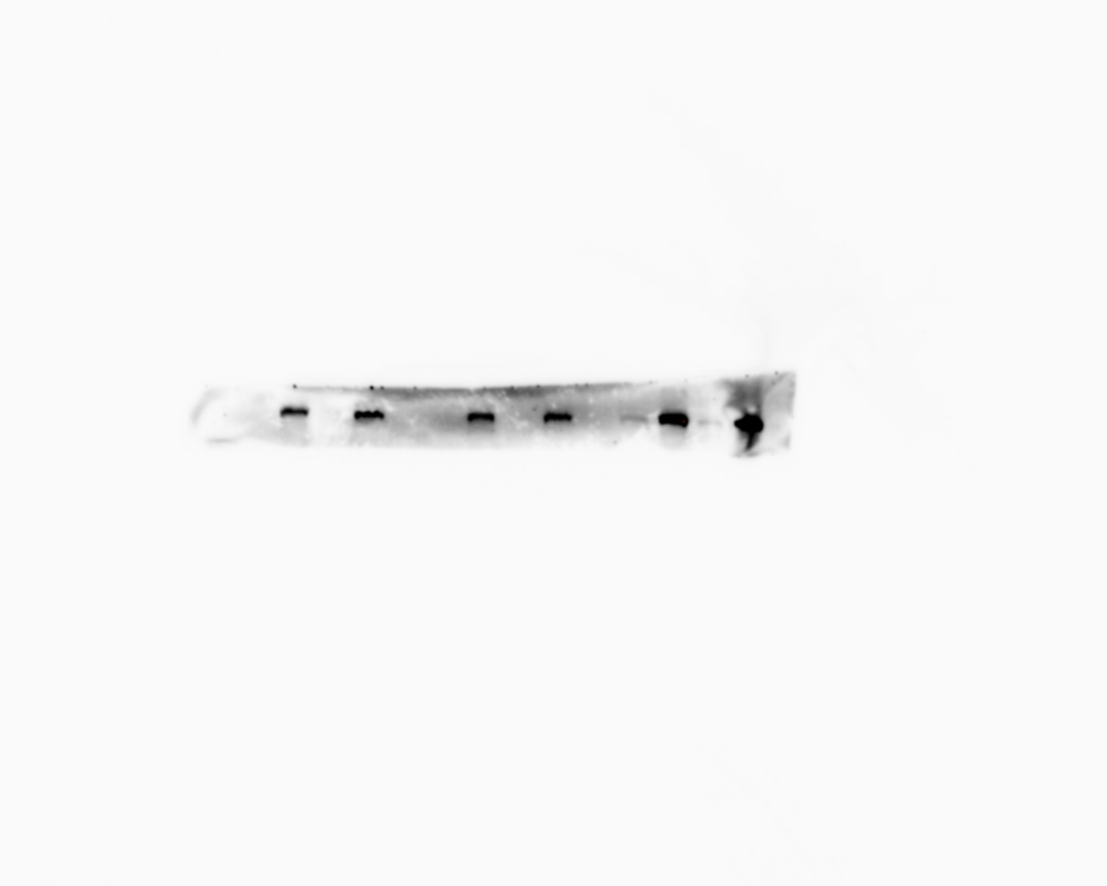

FKBP10-RCC4

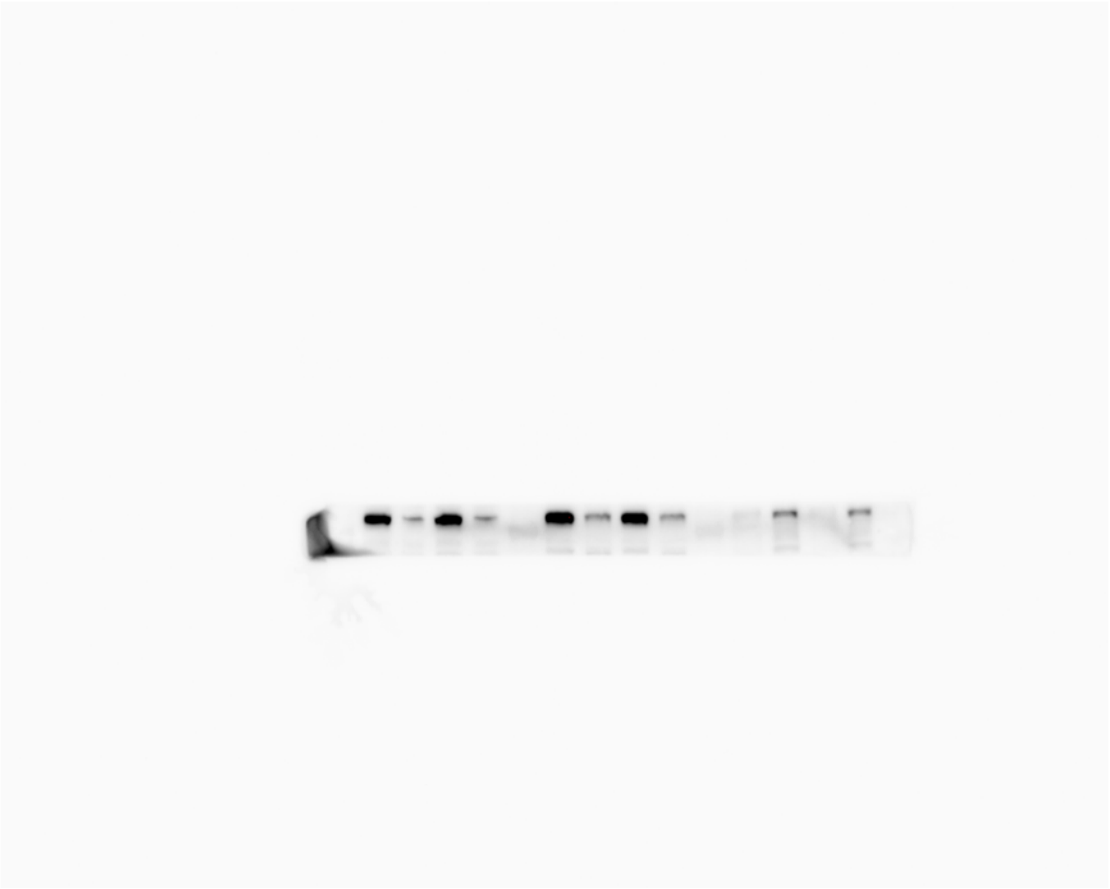

FKBP10-ACHN

FKBP10-Caki1

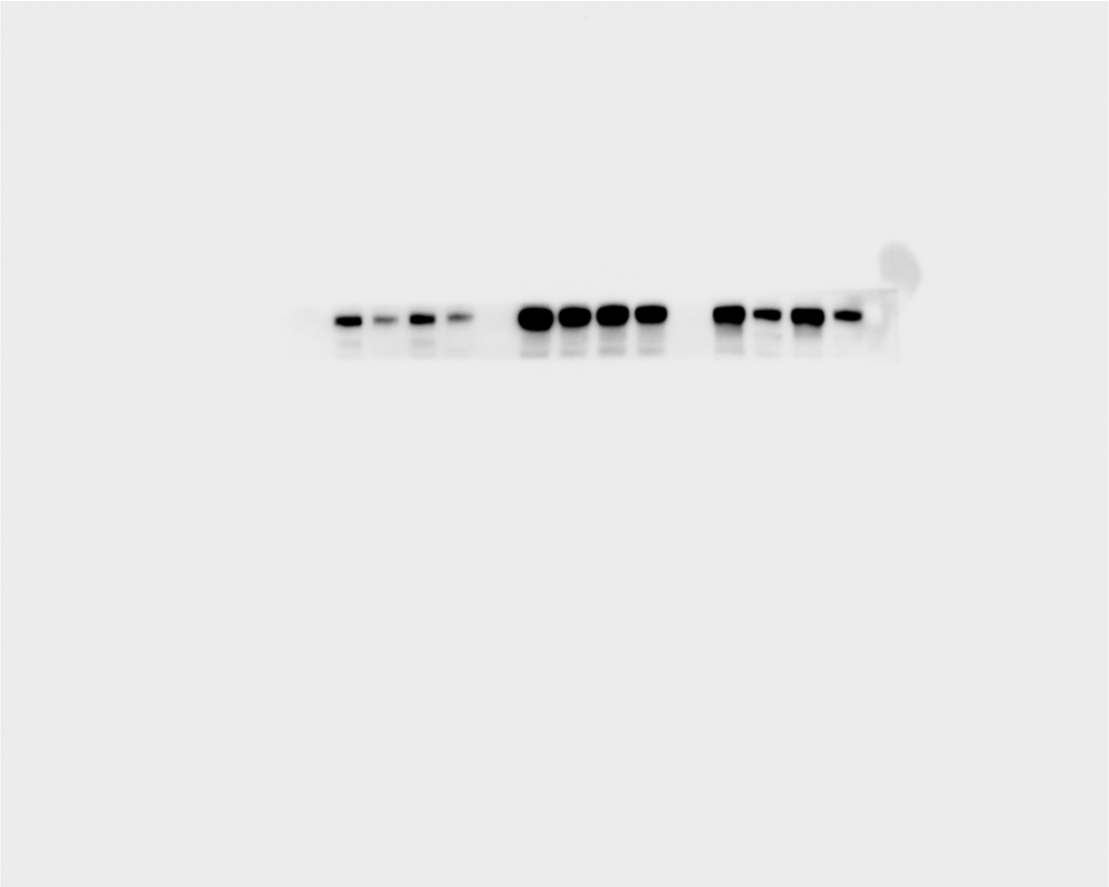

FKBP10-HK2

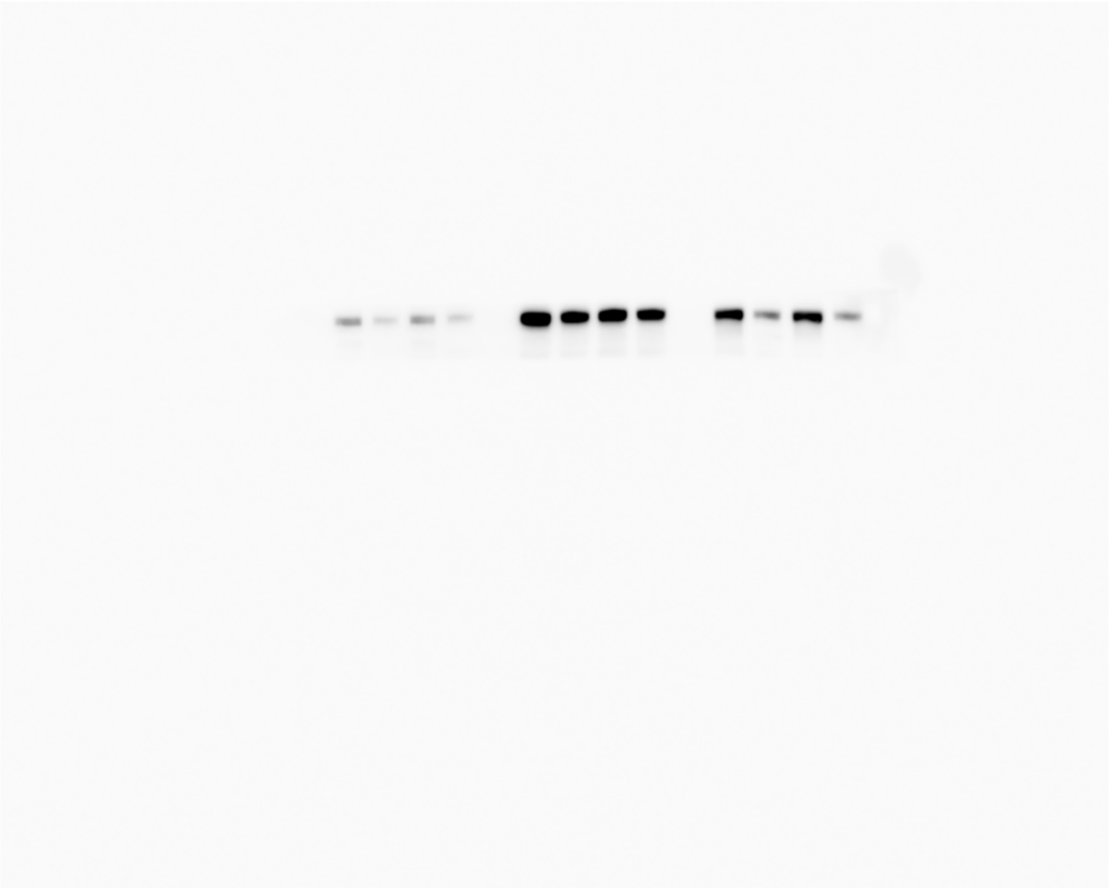

Fig. 7B
Actin

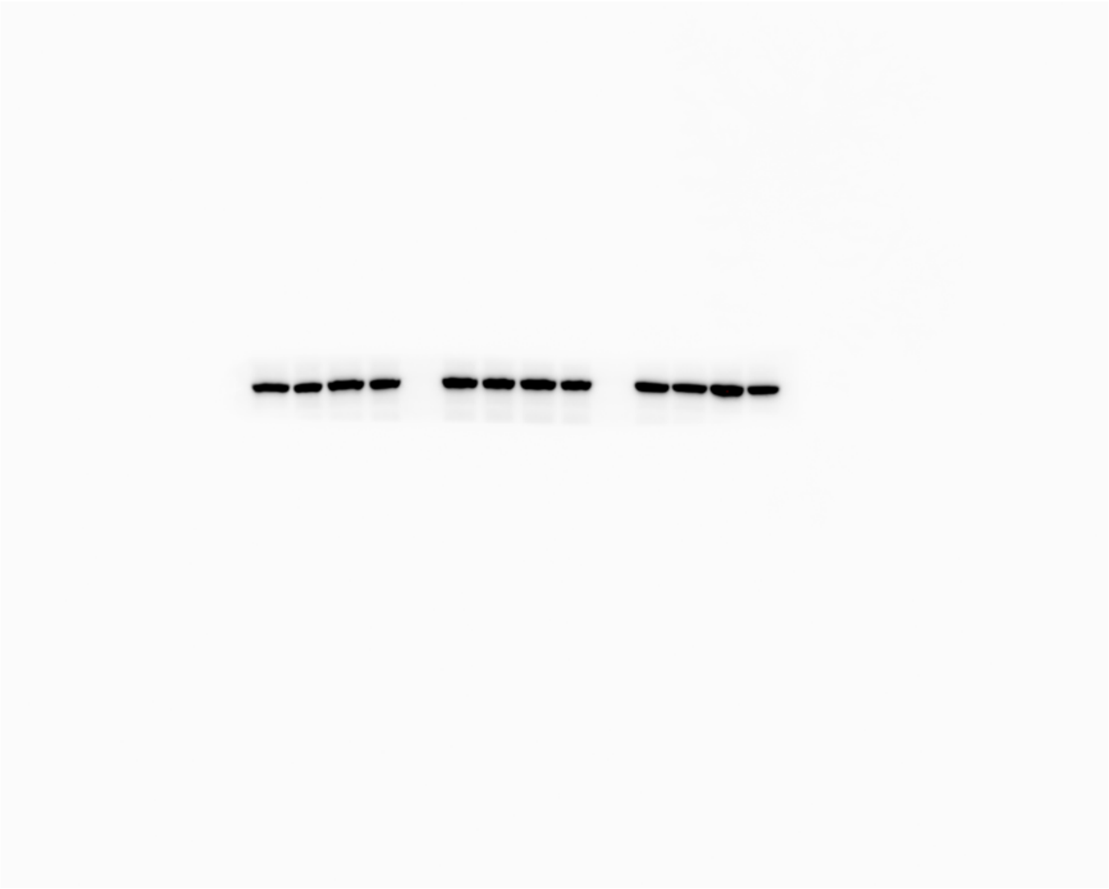

FKBP10

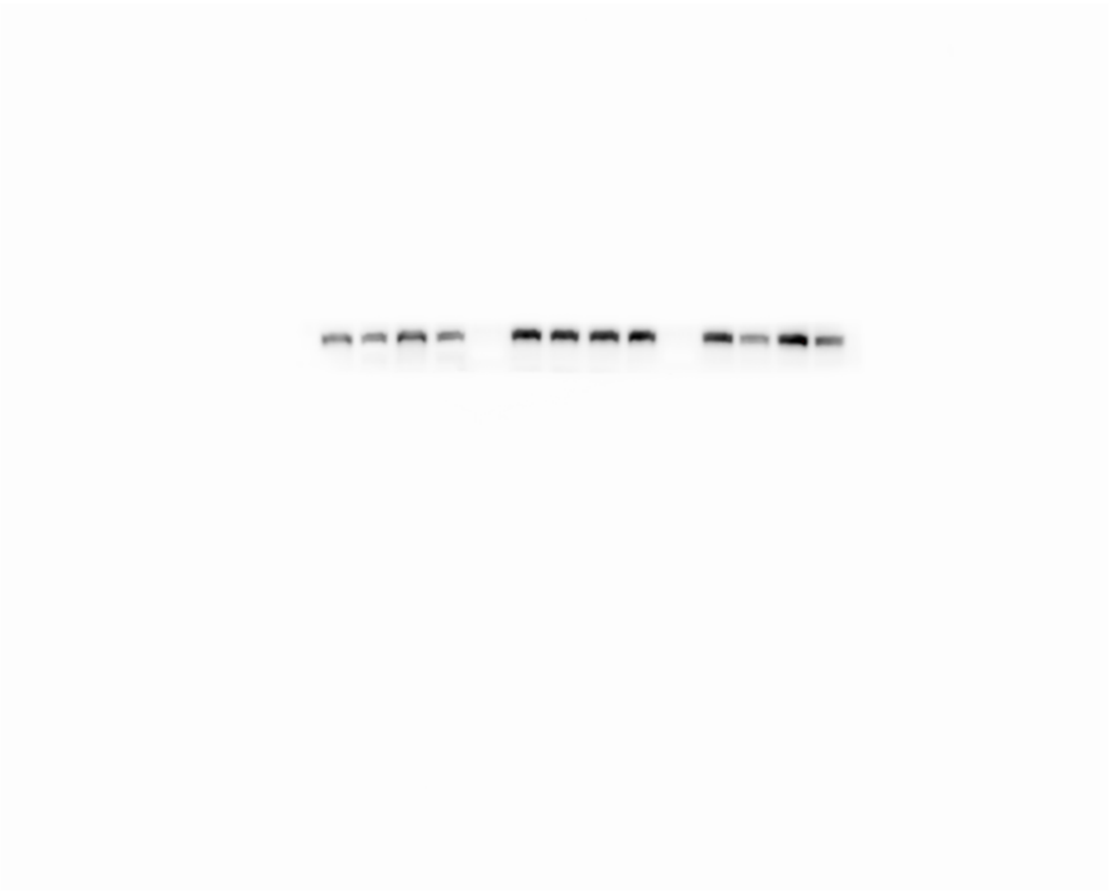

HIF1a

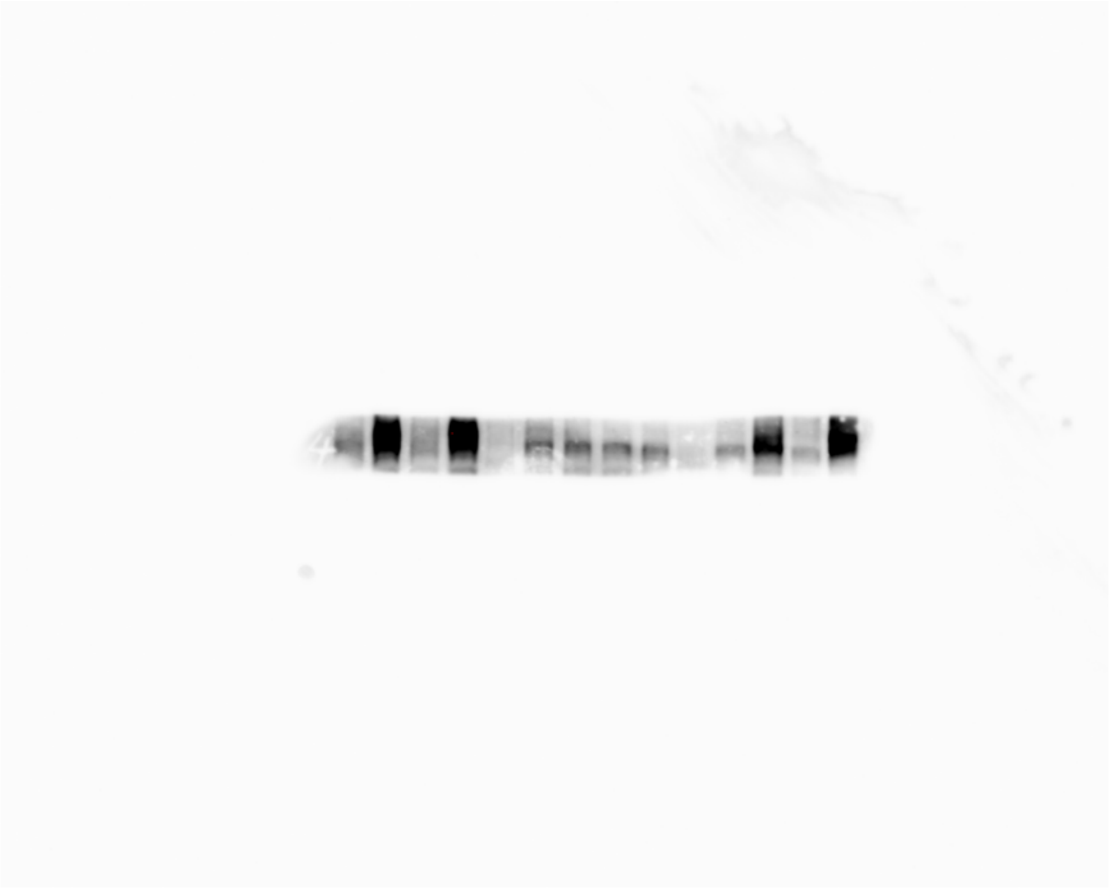

HIF2a

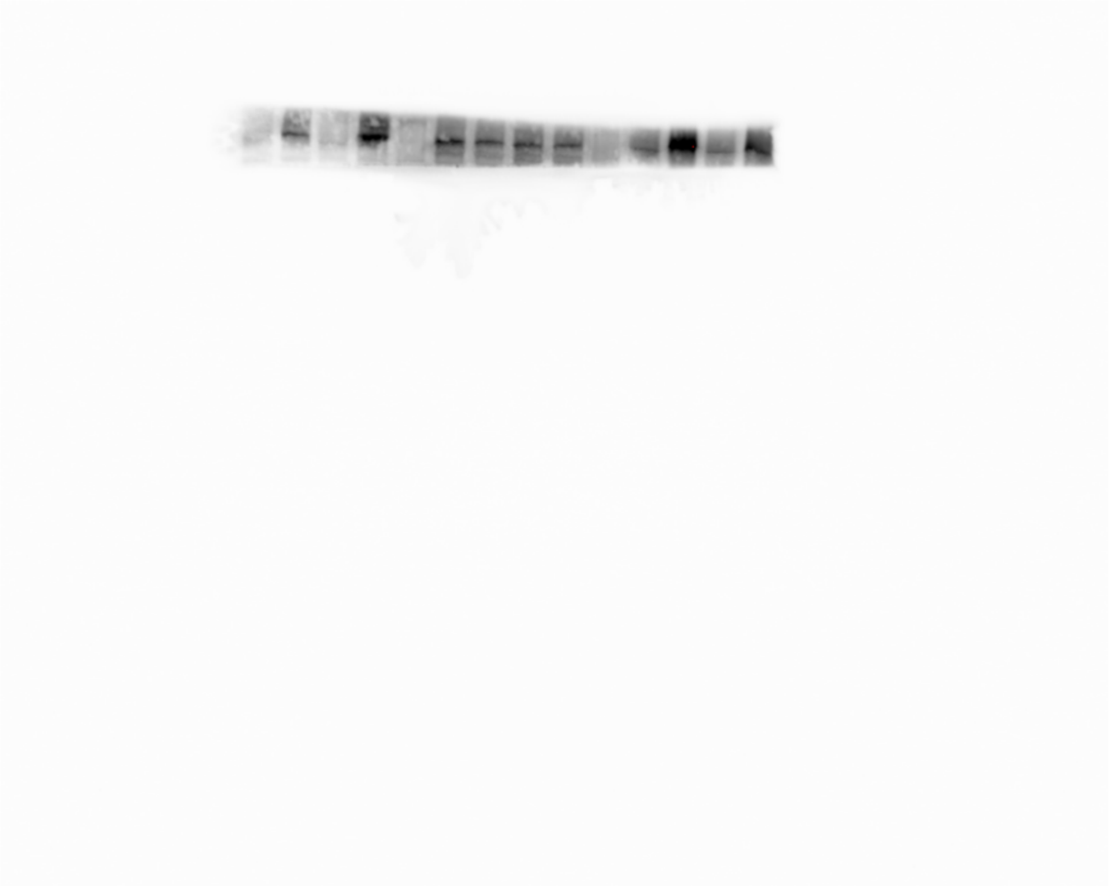

Fig. 7C
Actin

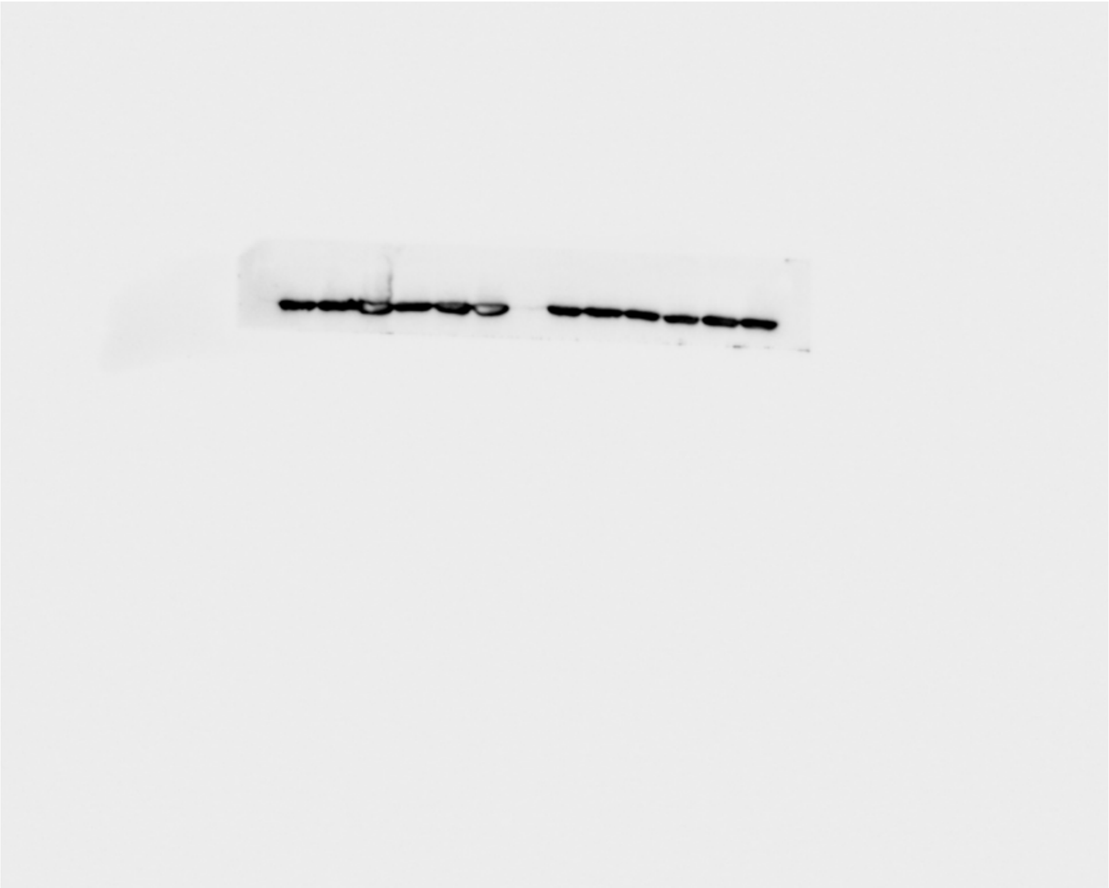

VHL

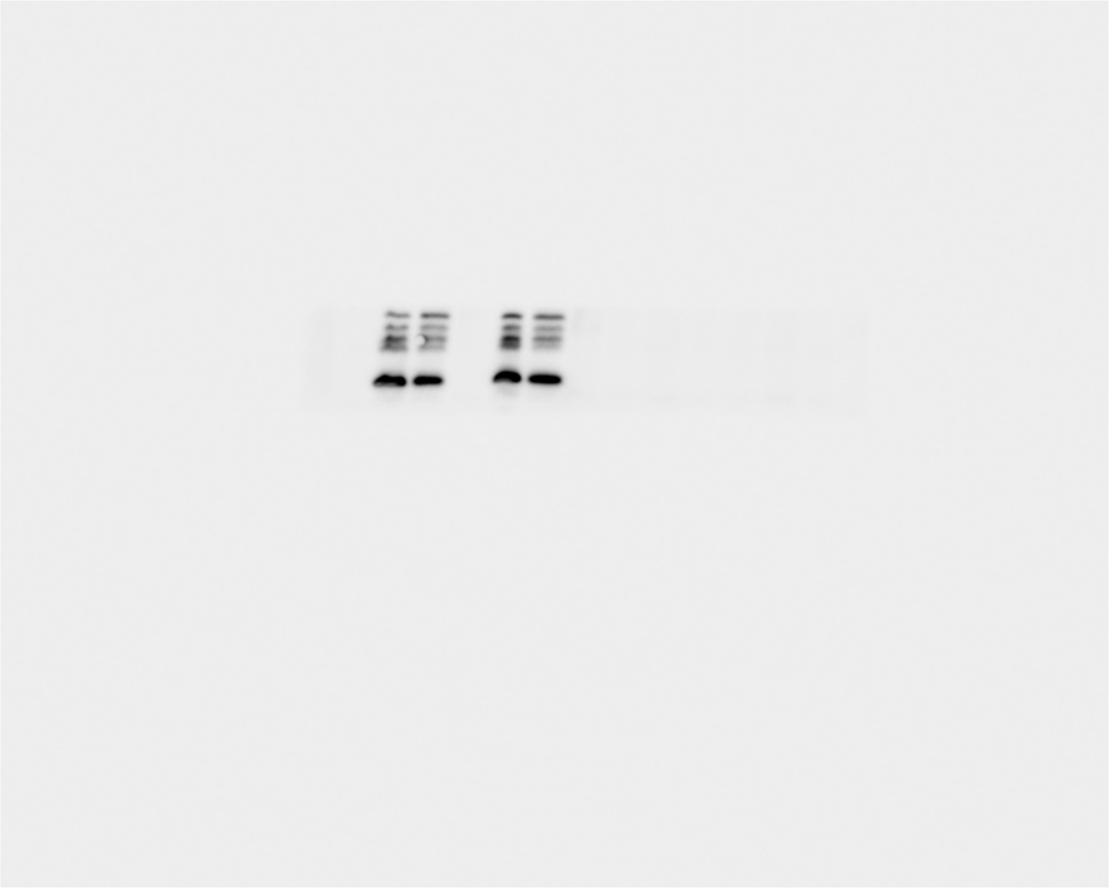

FKBP10

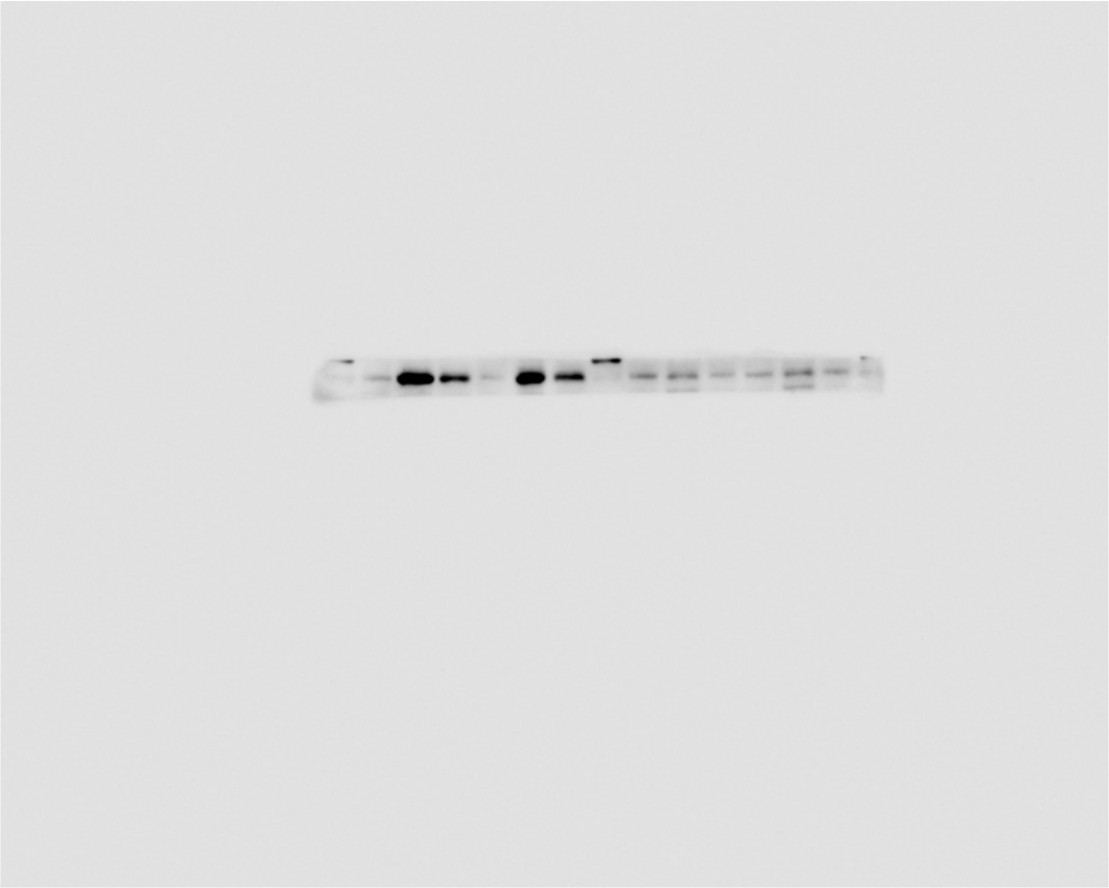

HIF2a

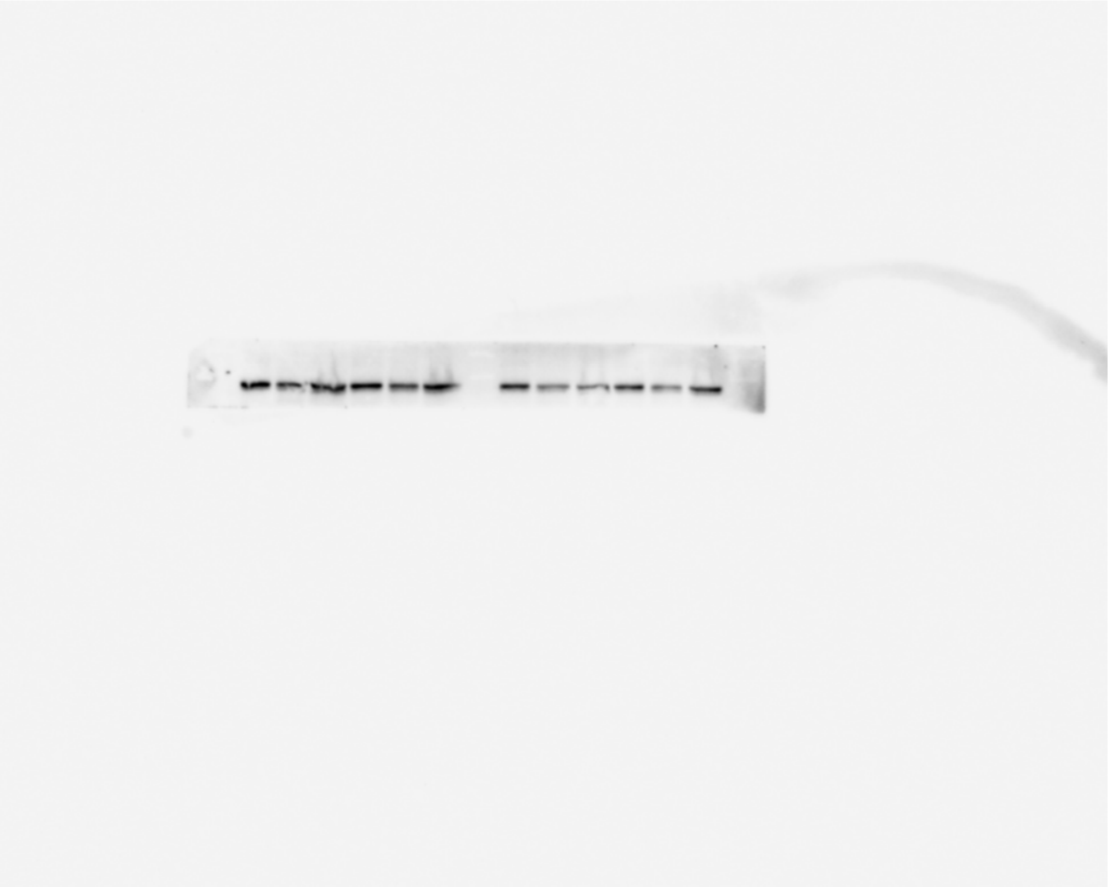

Fig. 7D
Actin

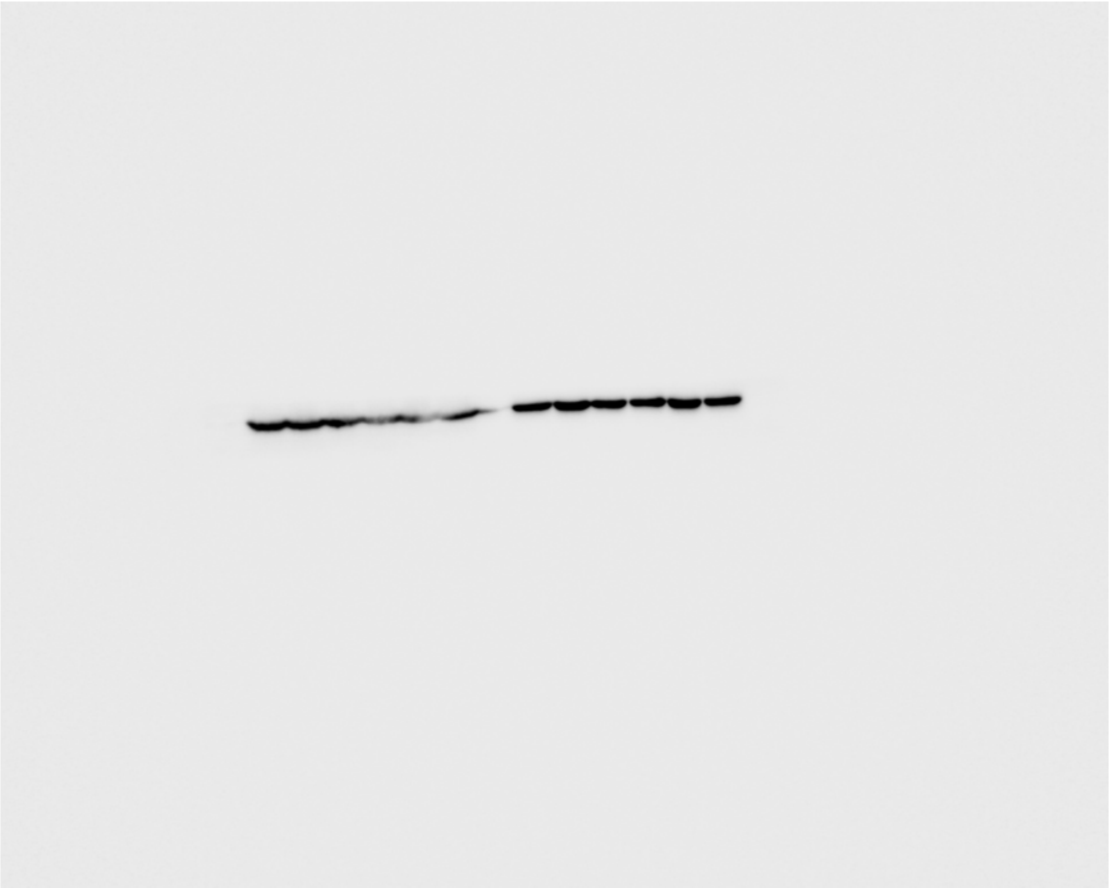

FKBP10
HIF2a

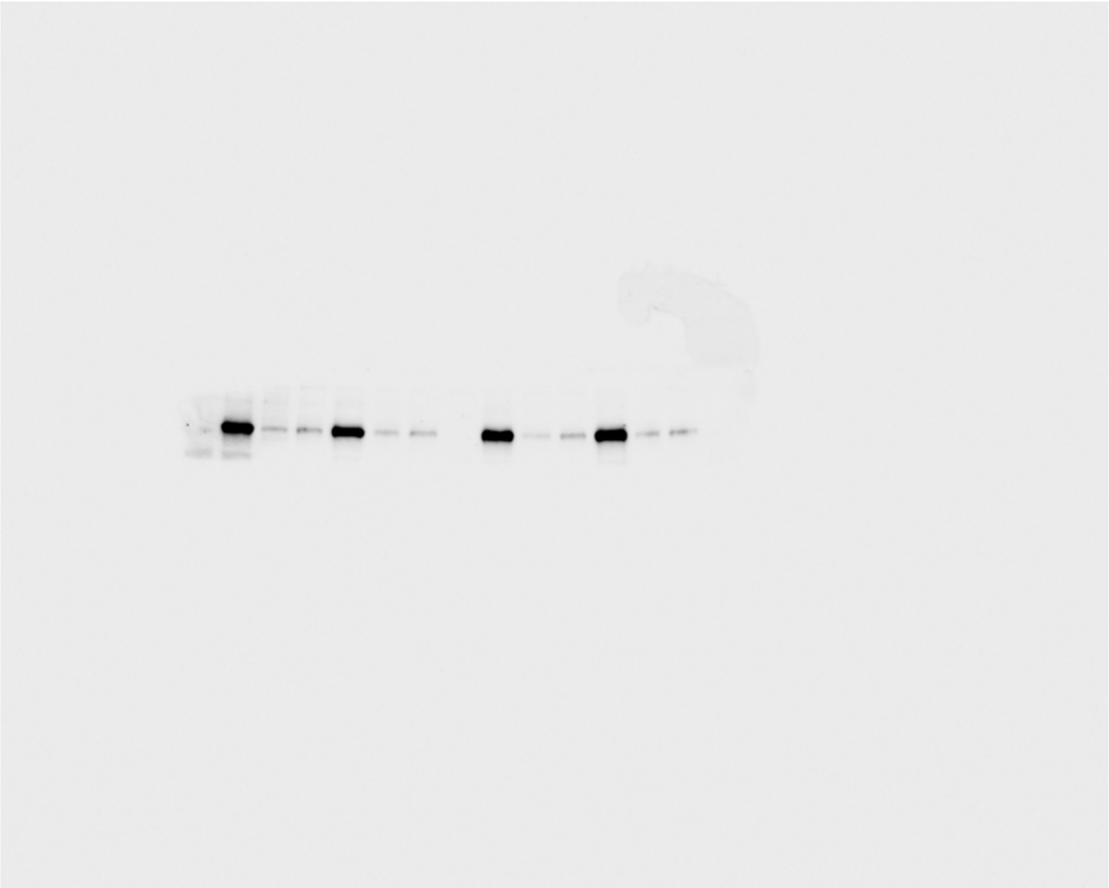

Fig. 7E
Actin

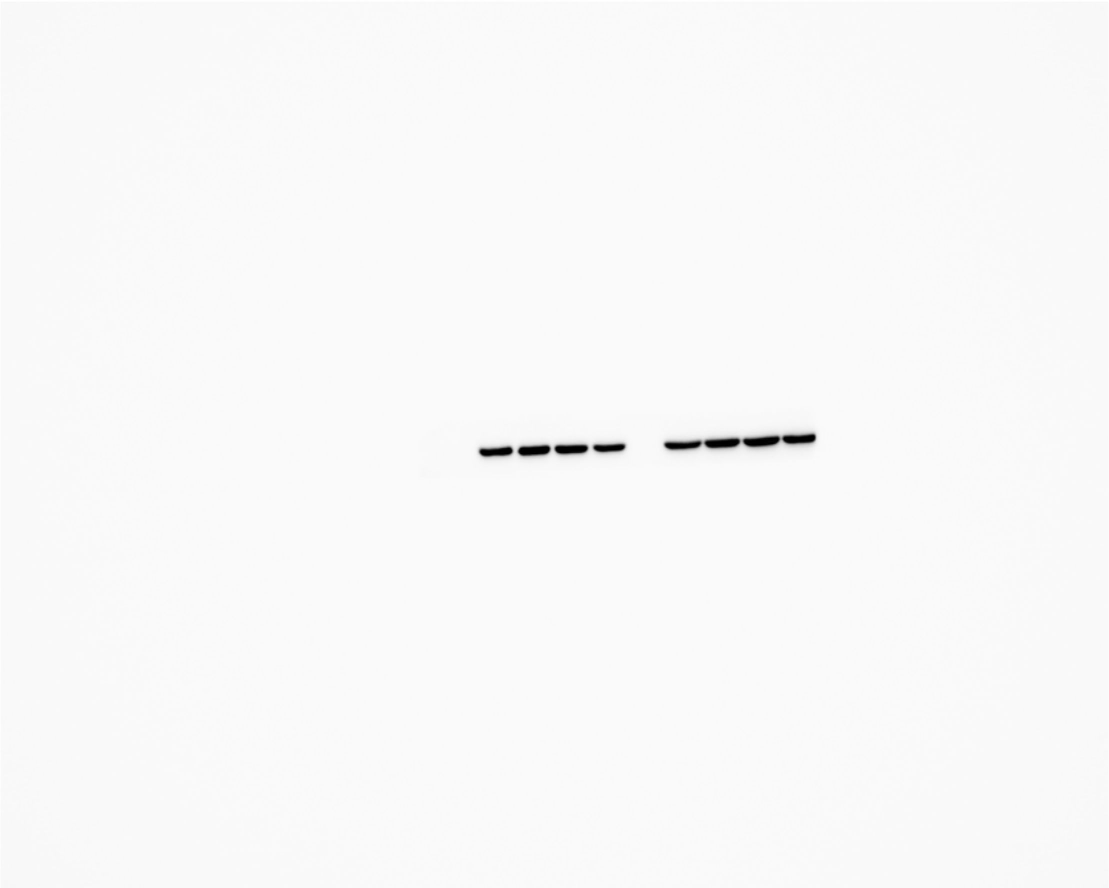

FKBP10

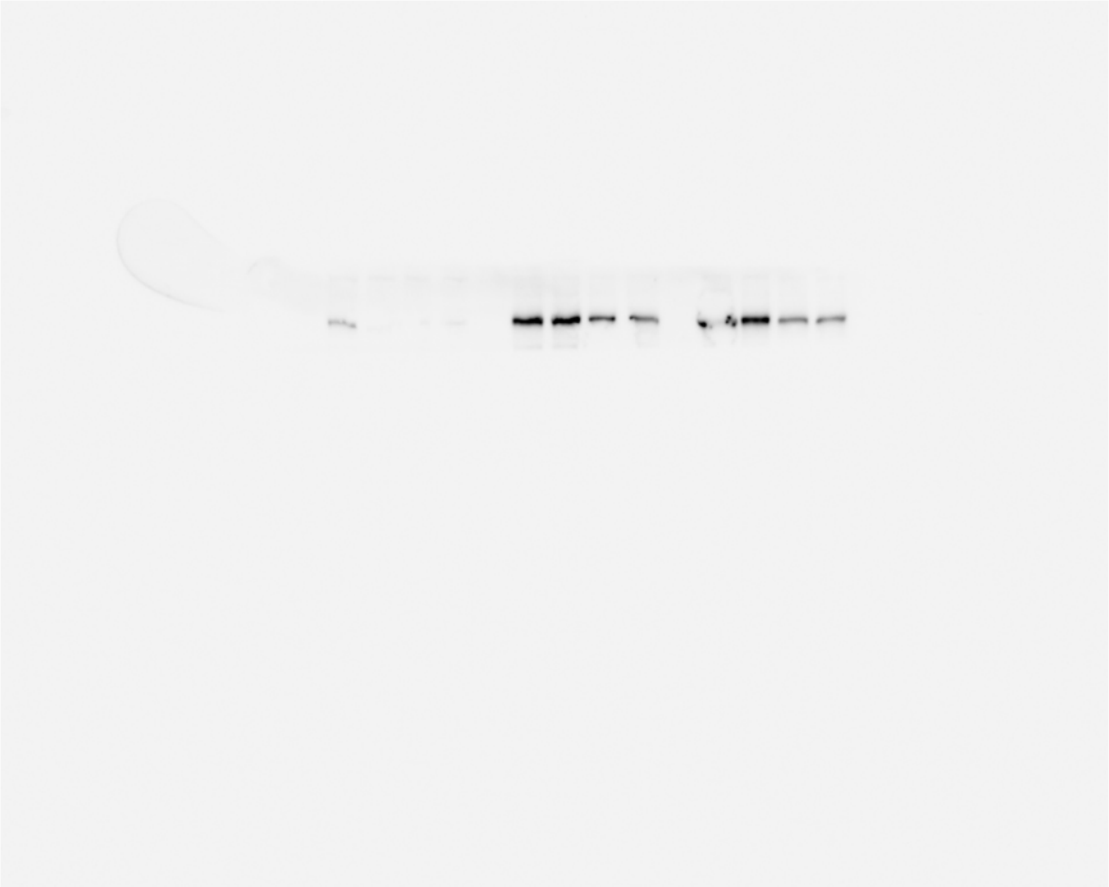

HIF1a

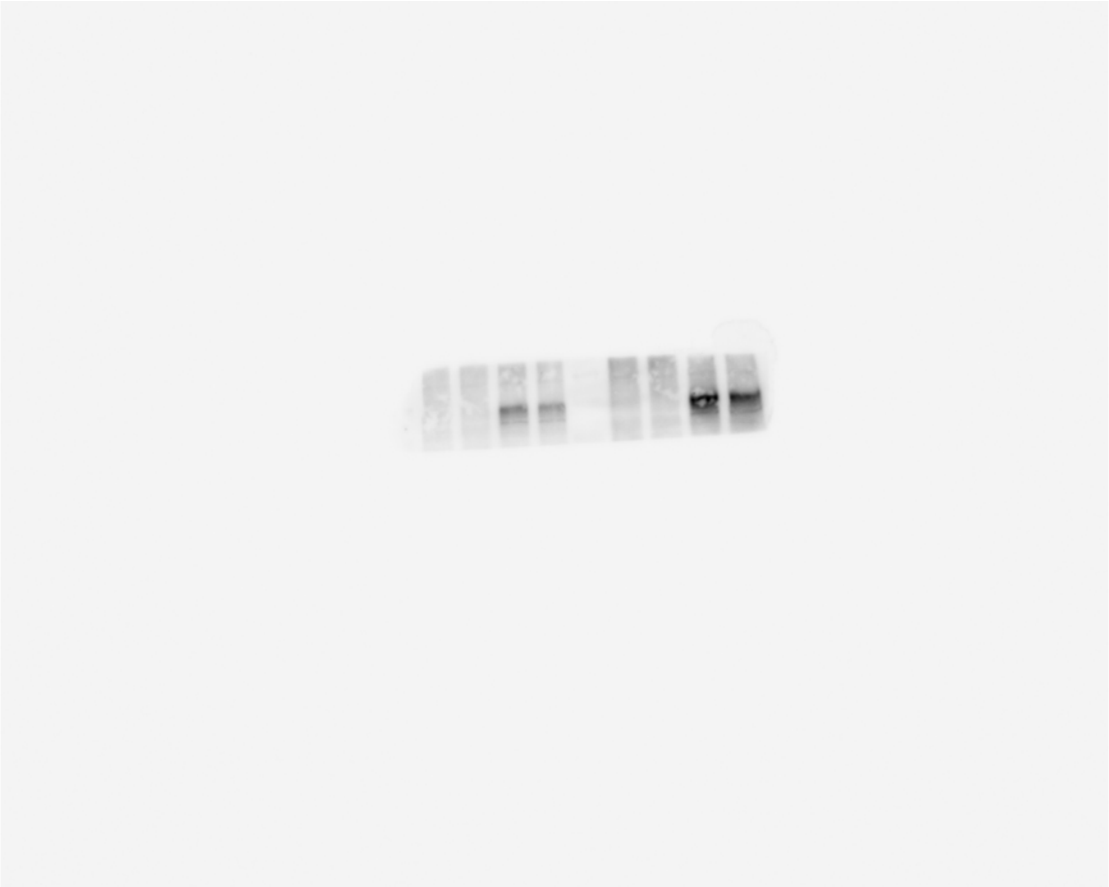

Fig. 7H

Actin

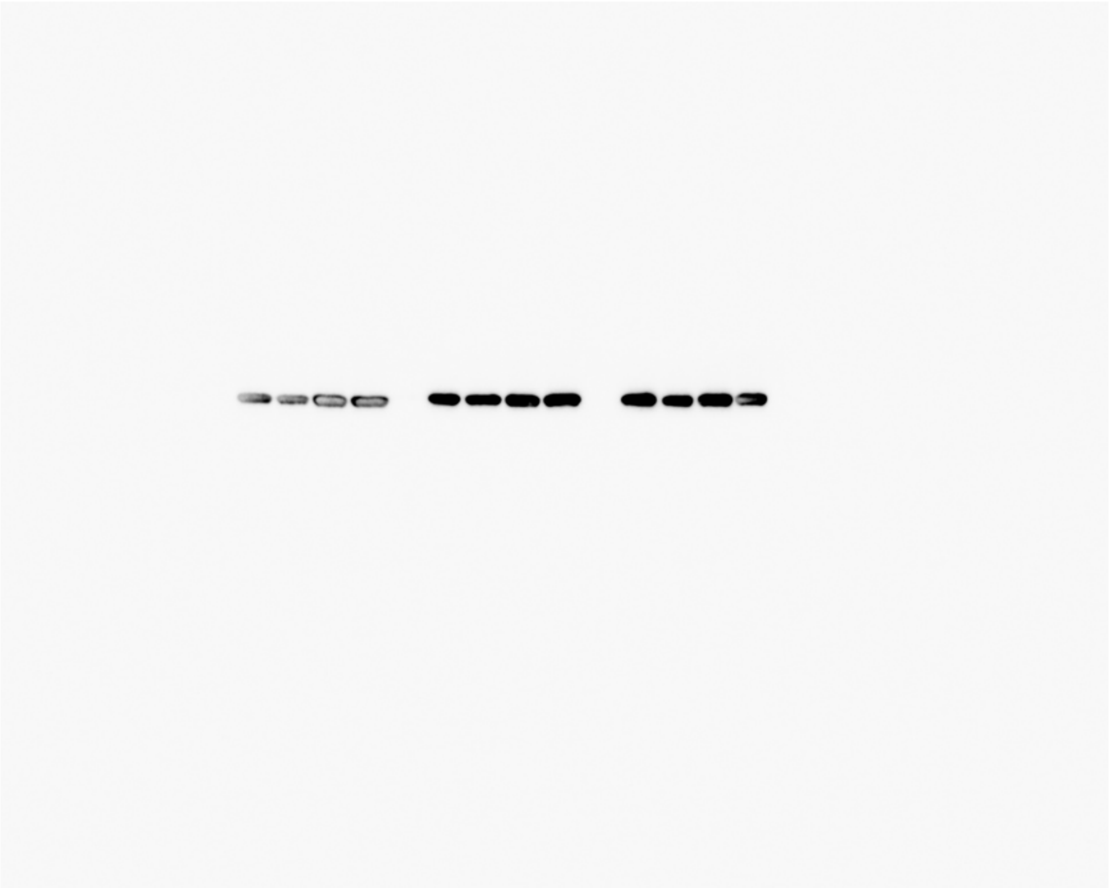

FKBP10

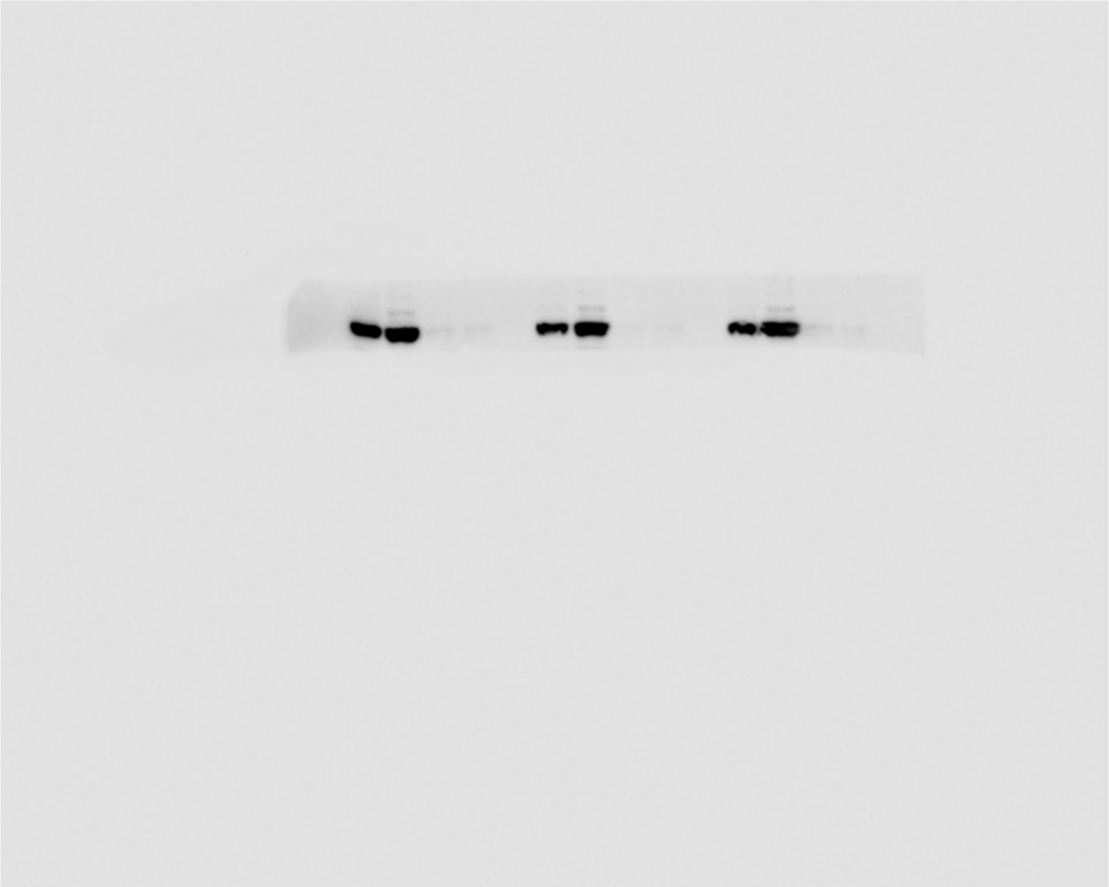

LDHA

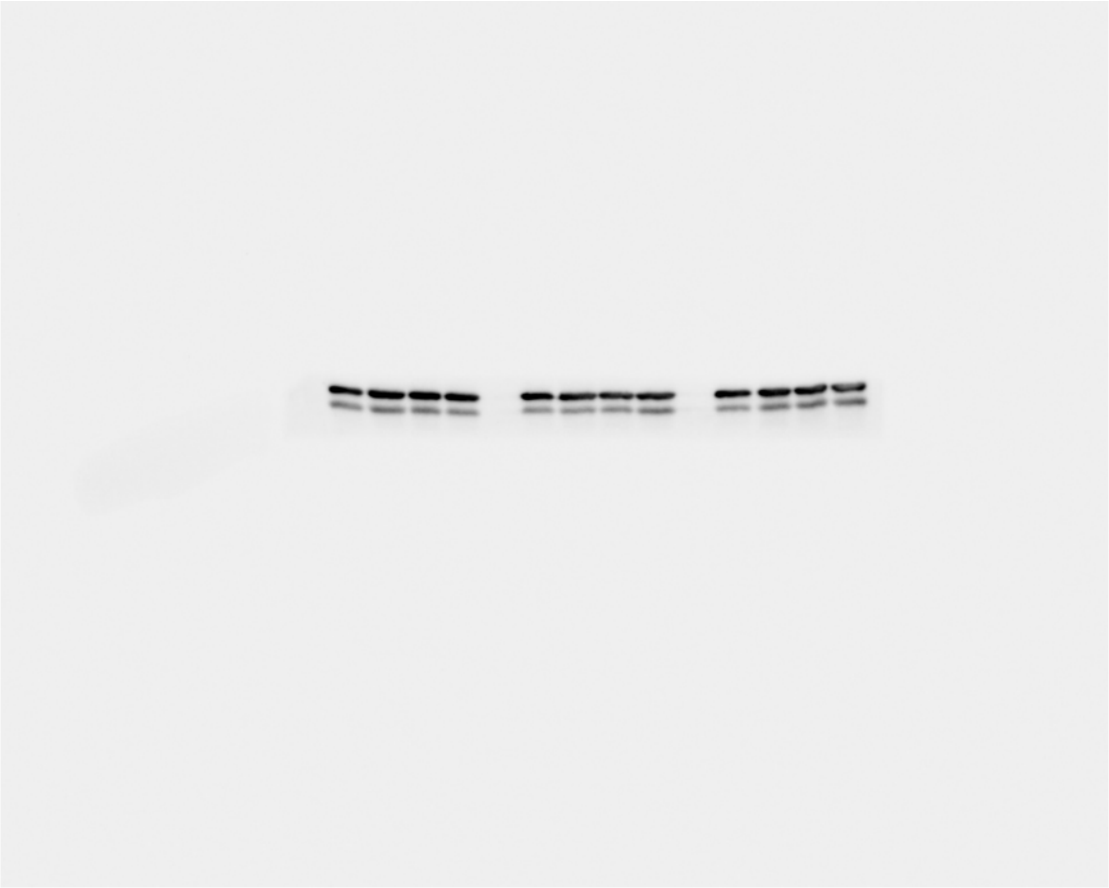

pLDHA

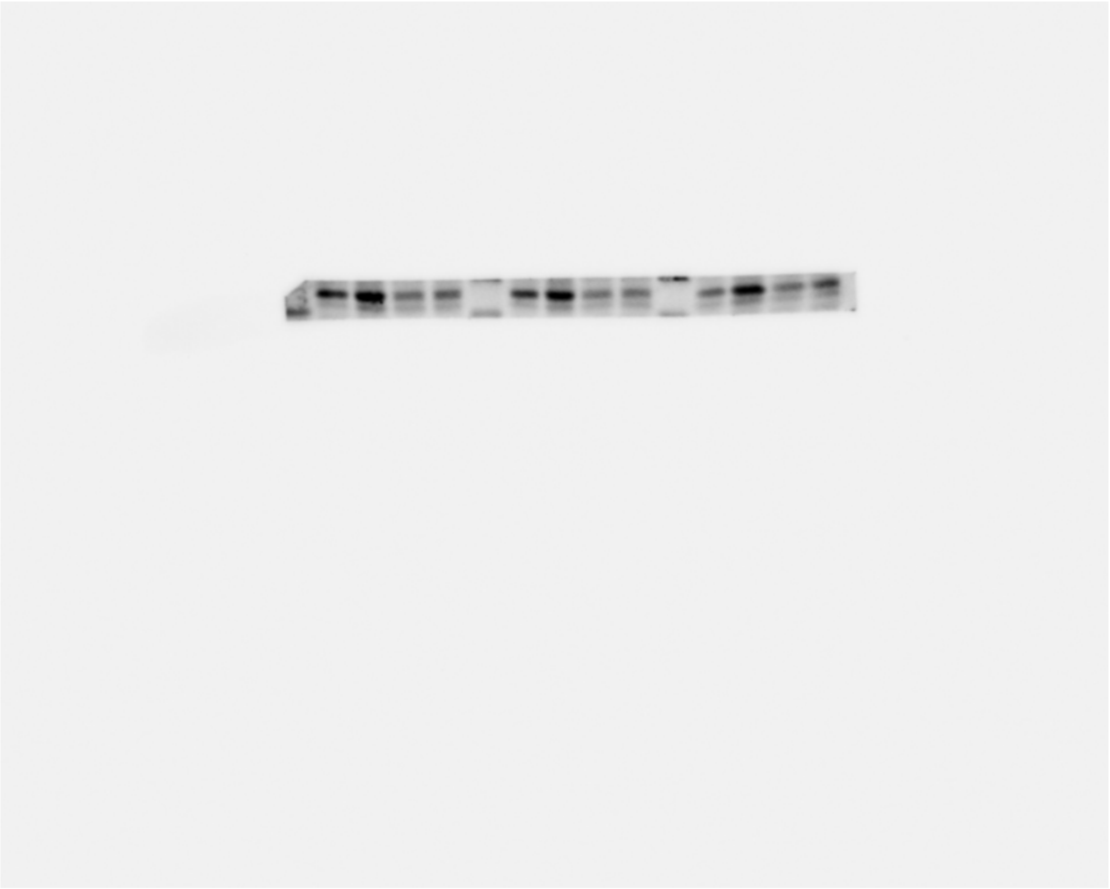

HIF2a

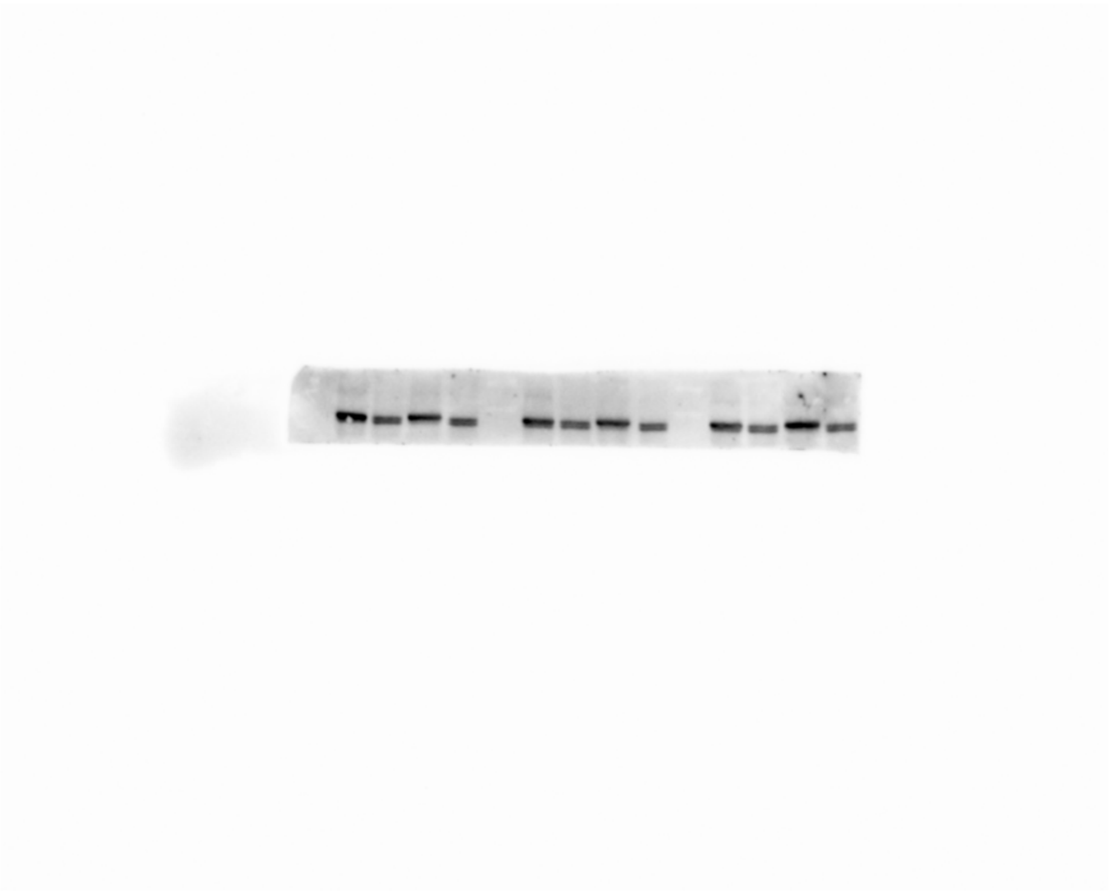

Fig. S1E
Actin

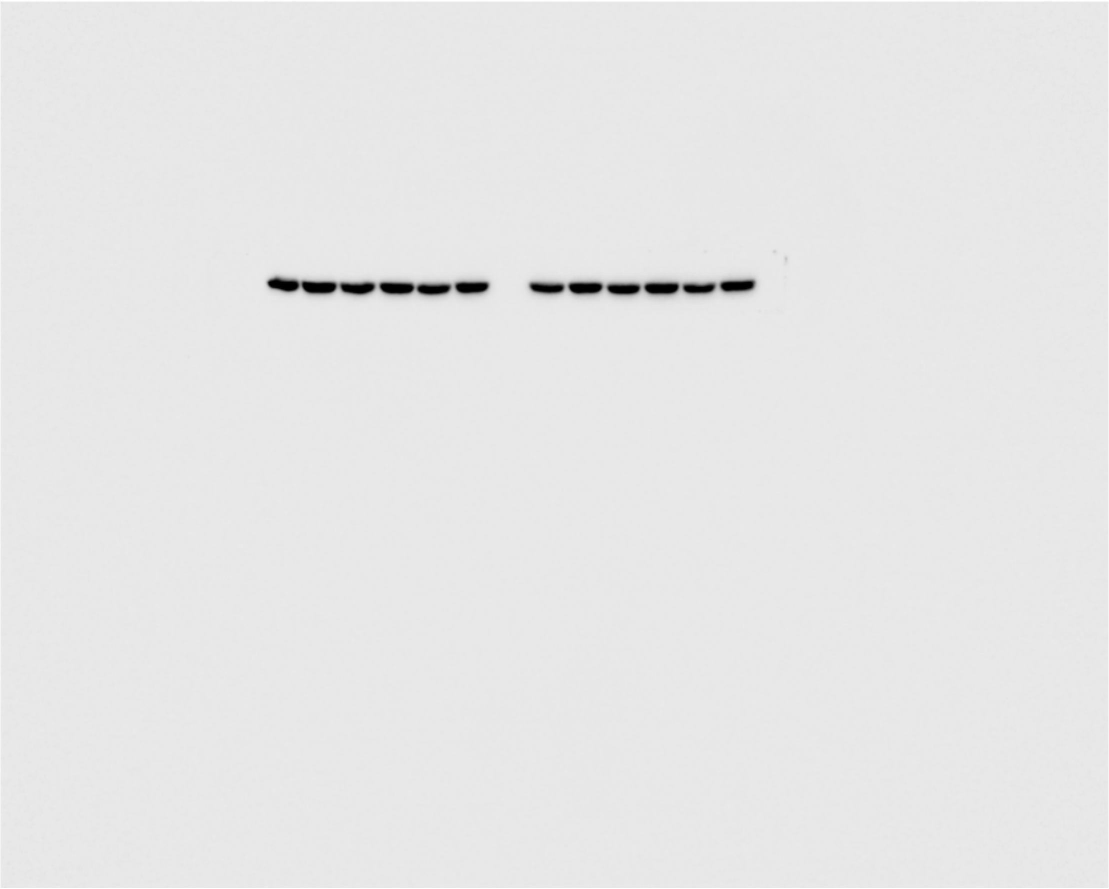

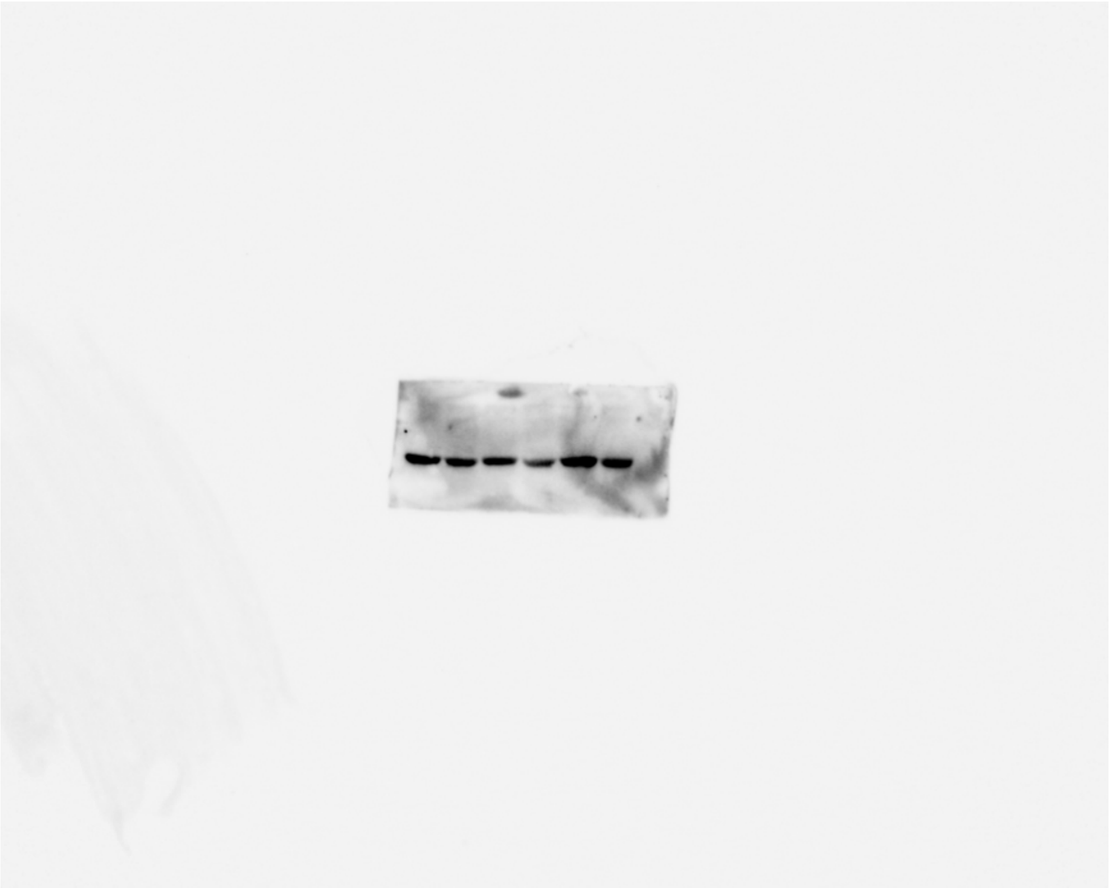

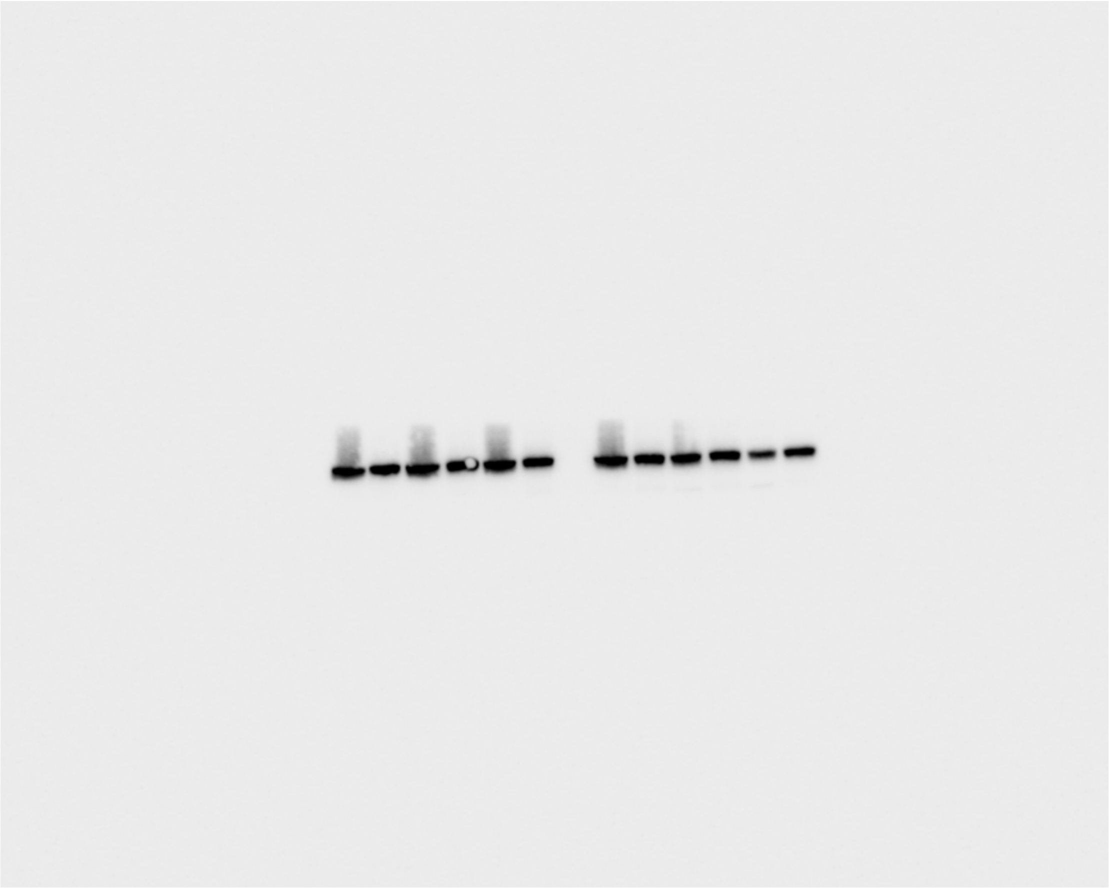

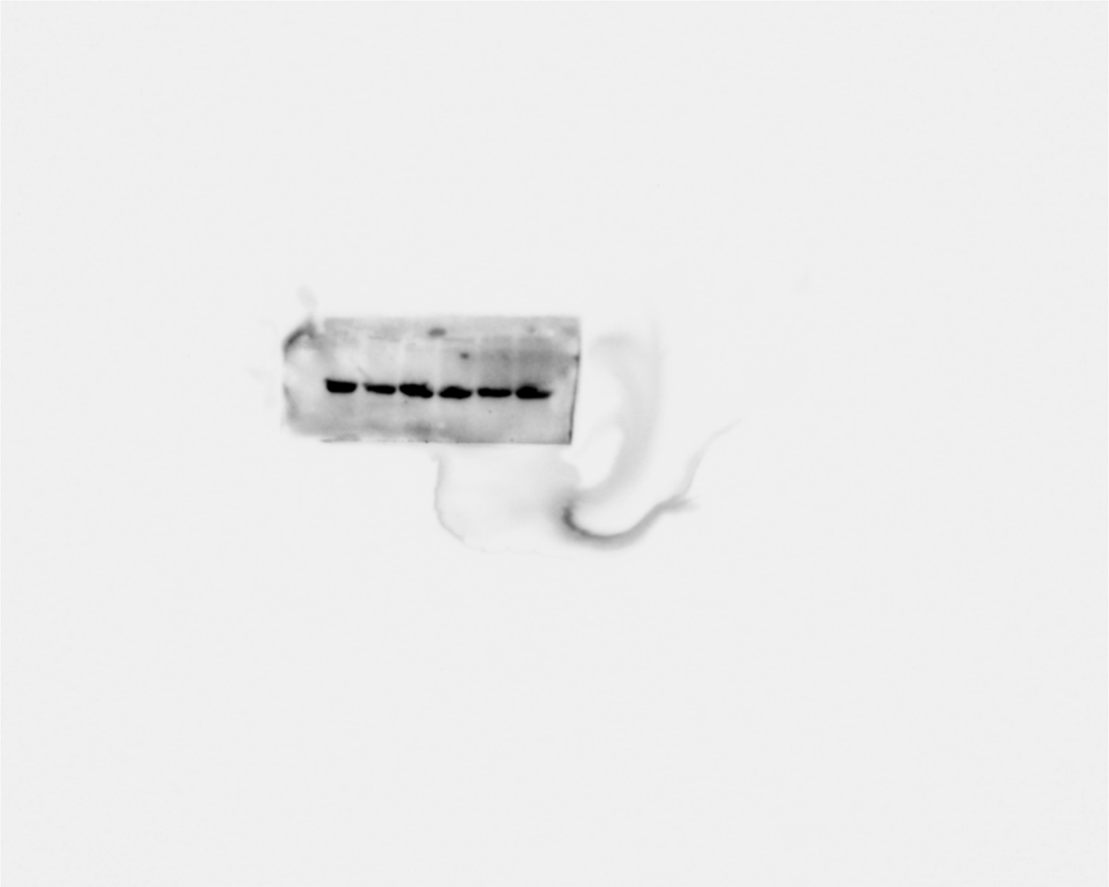

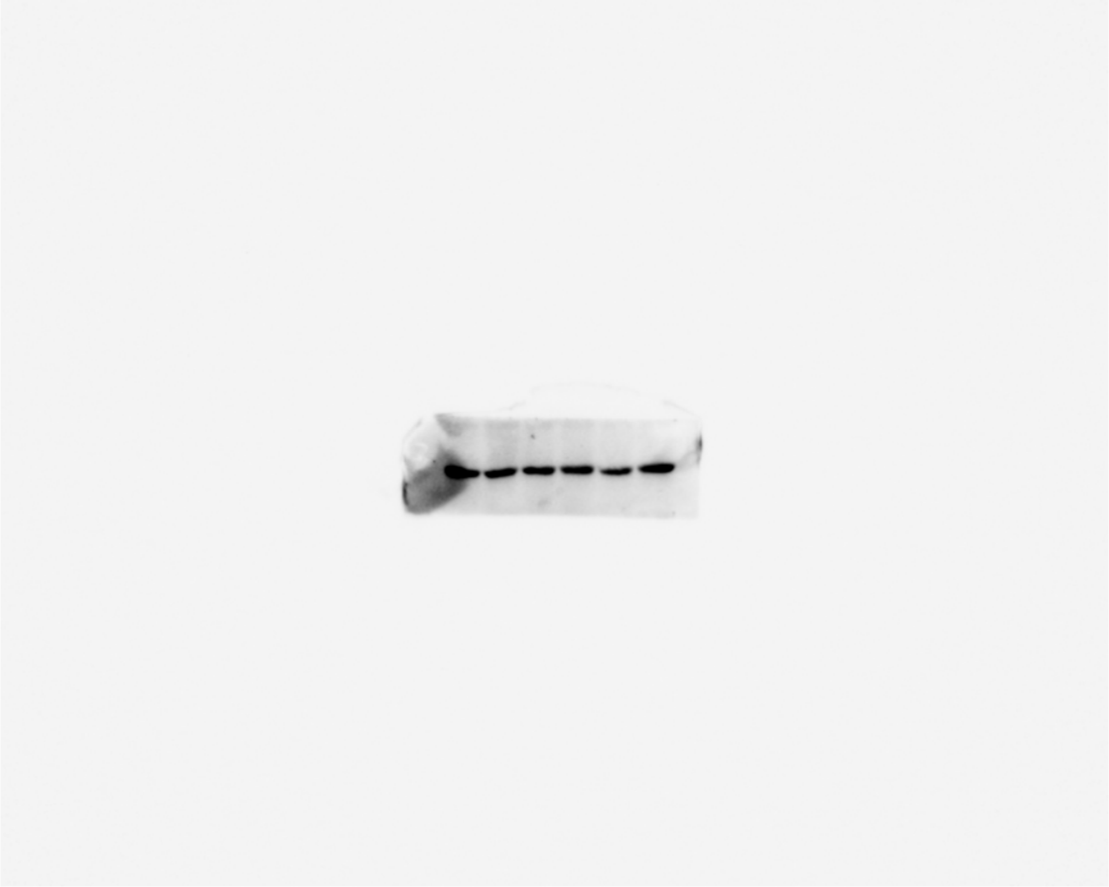

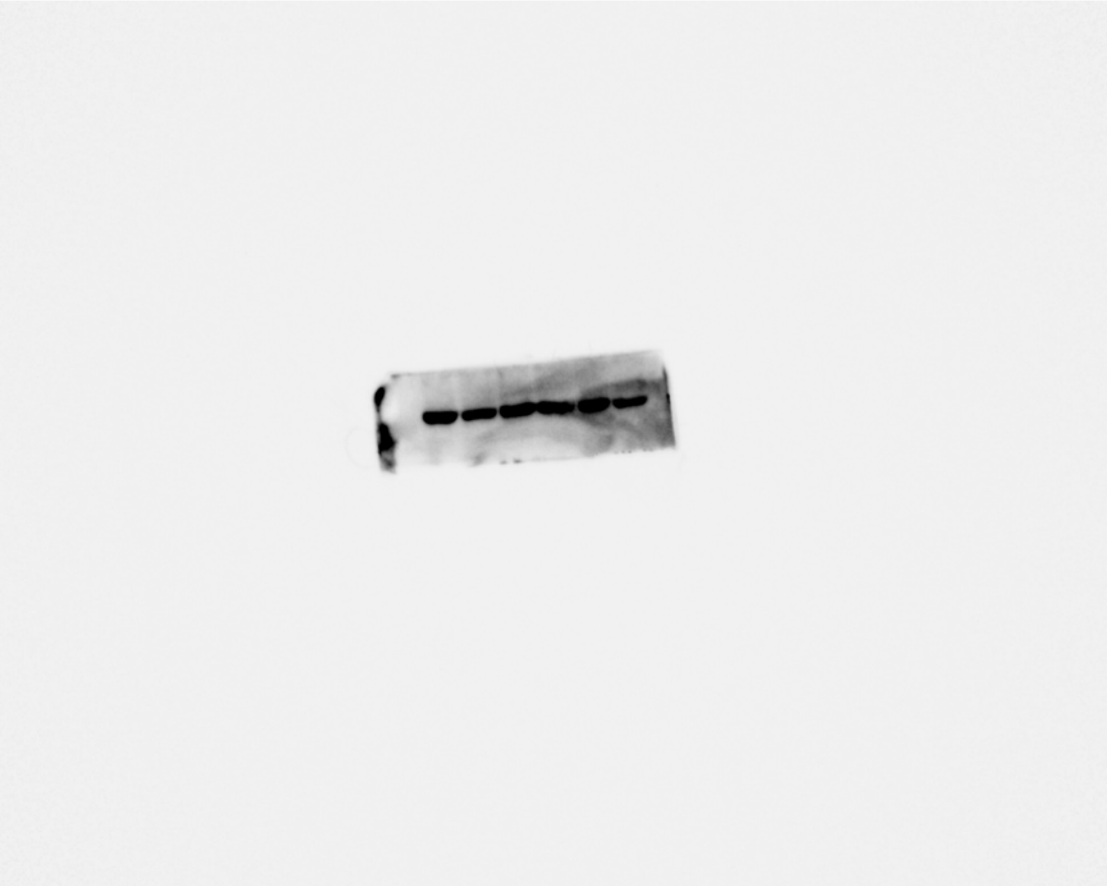

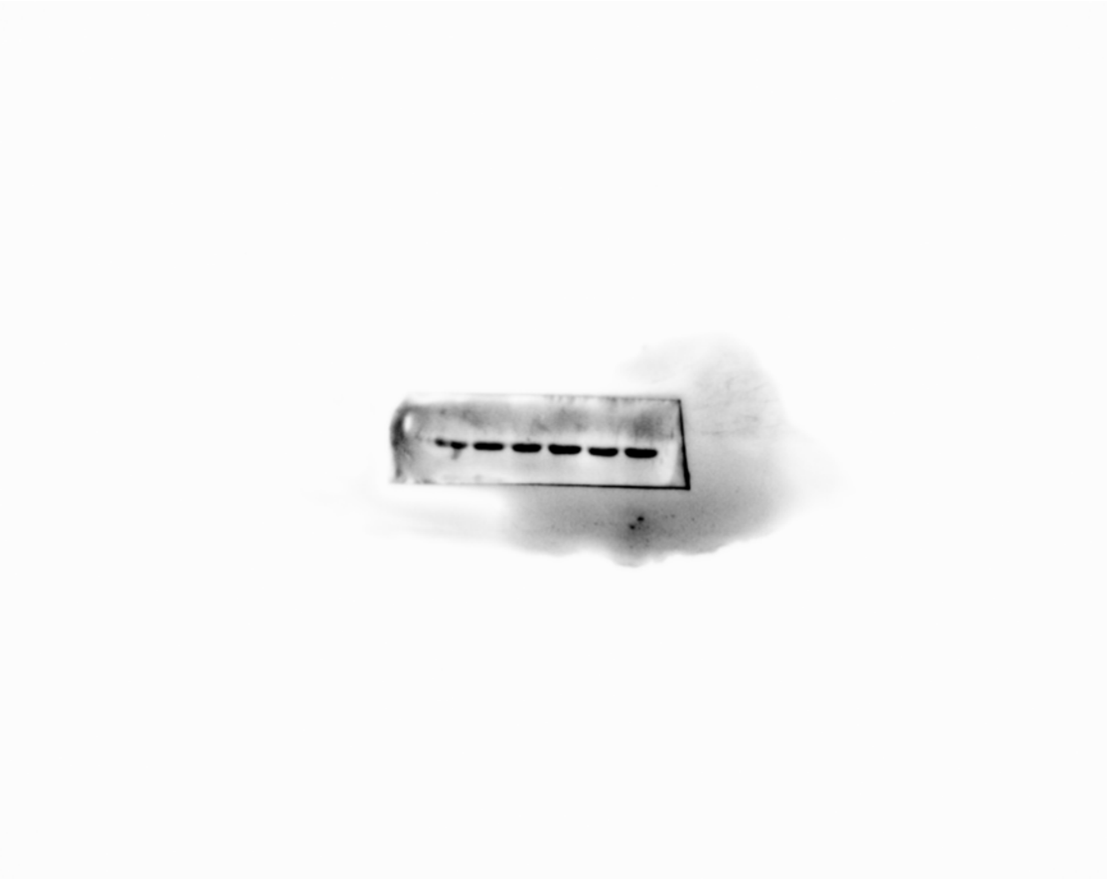

FKBP10

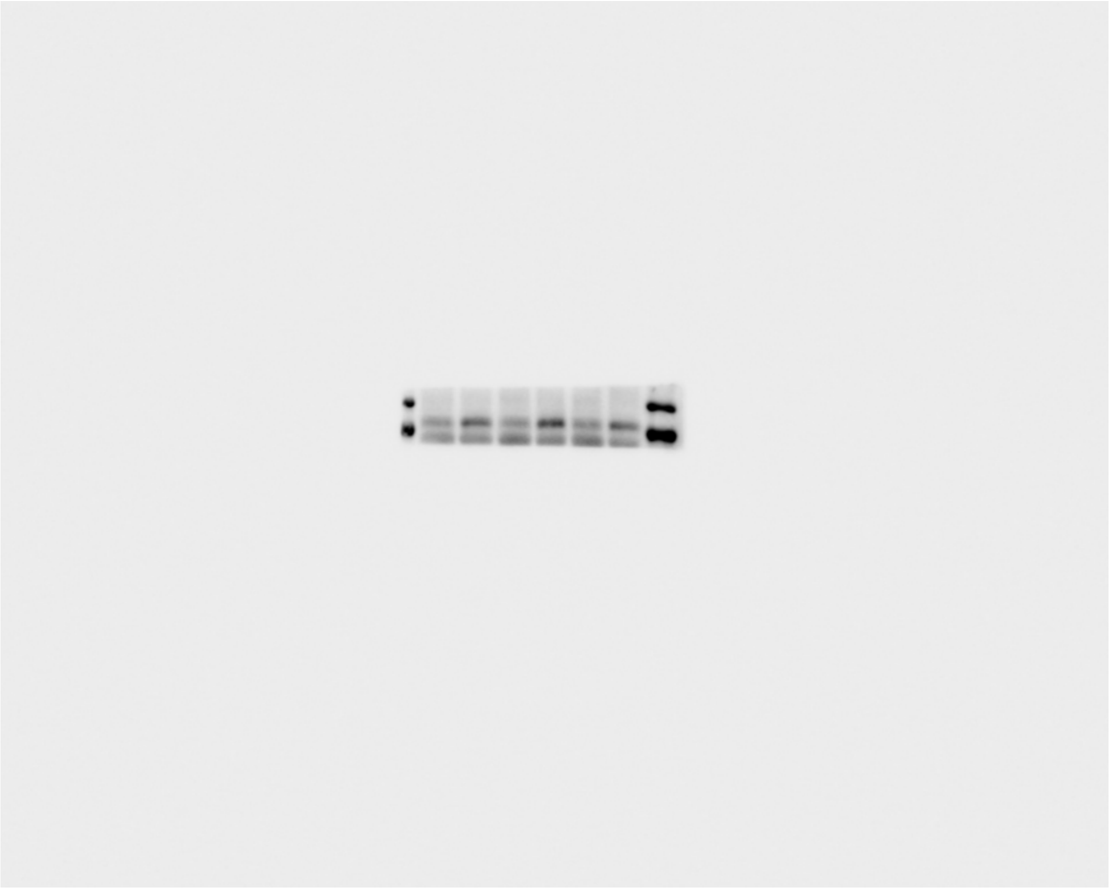

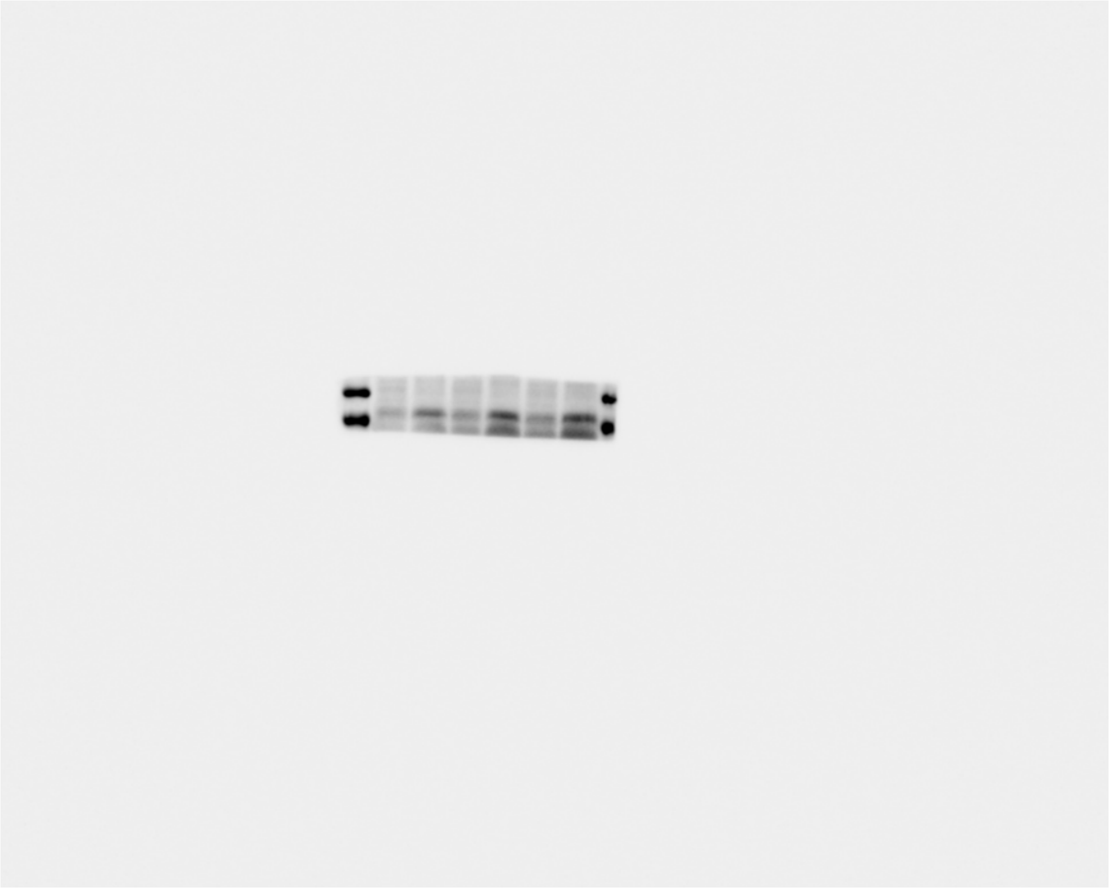

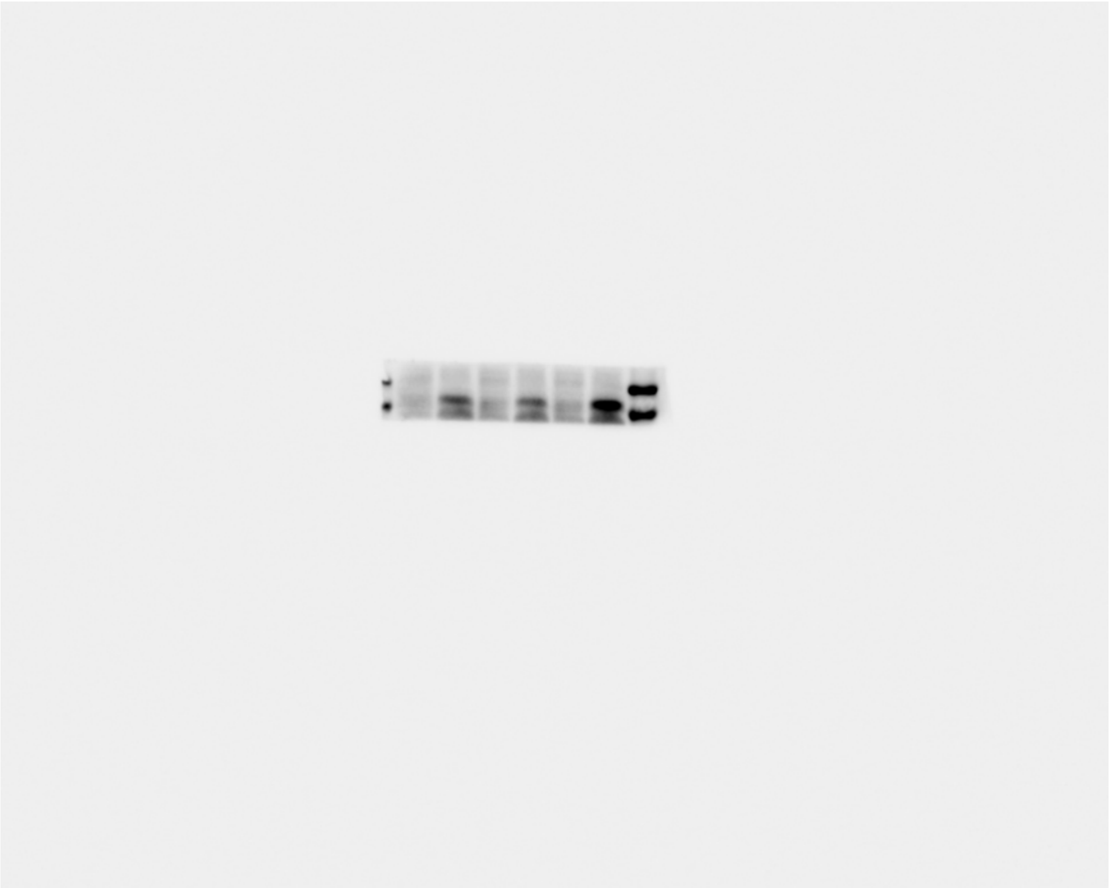

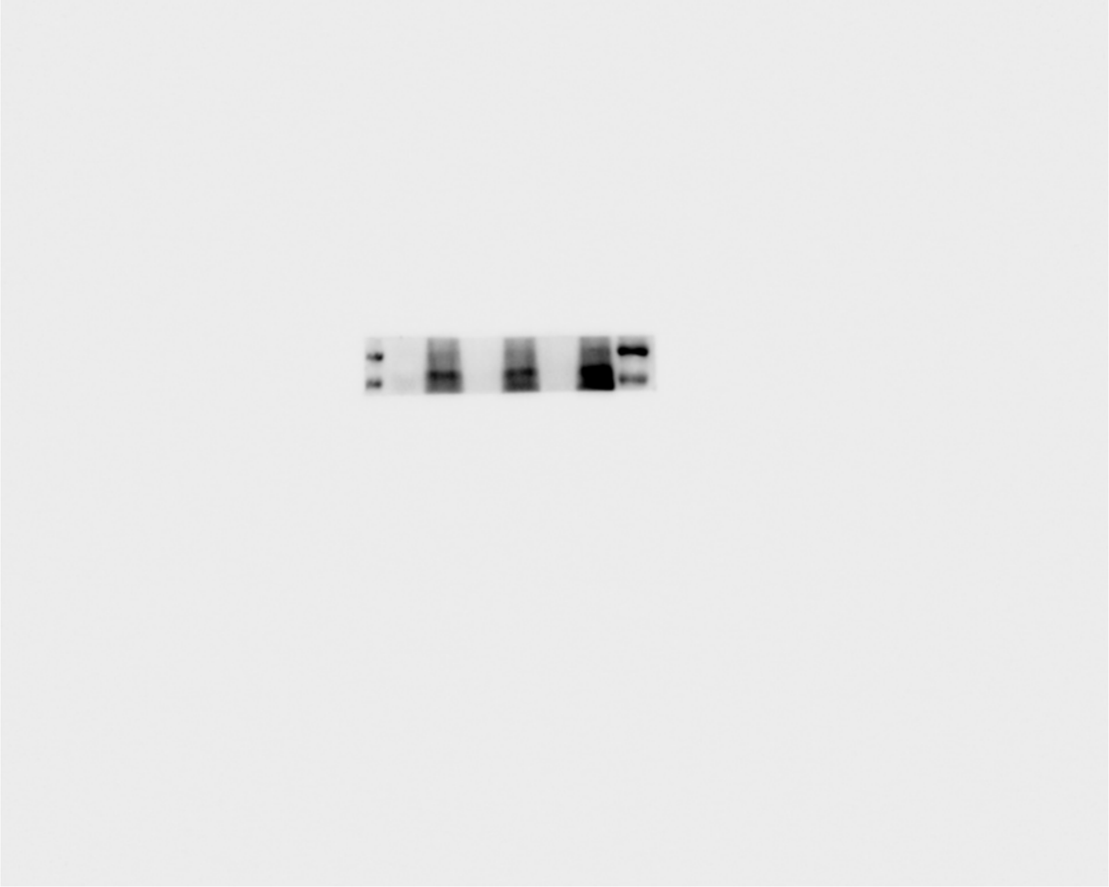

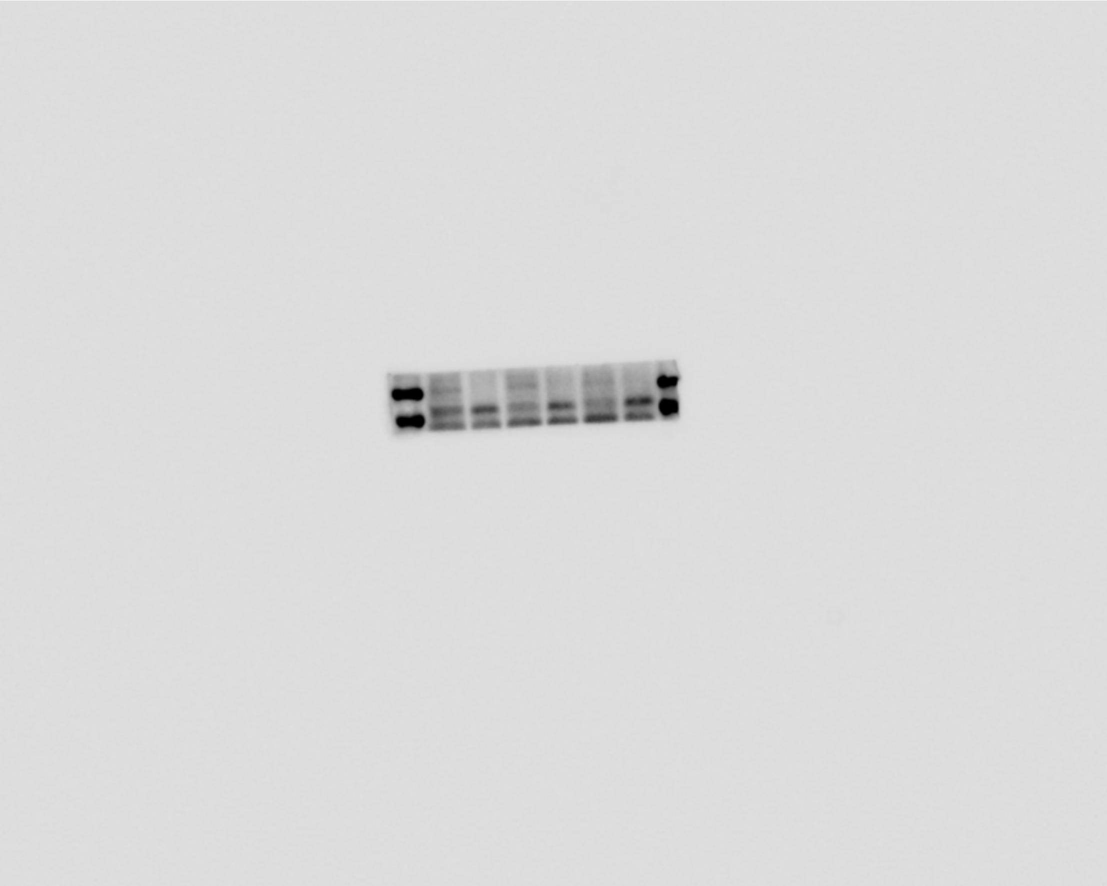

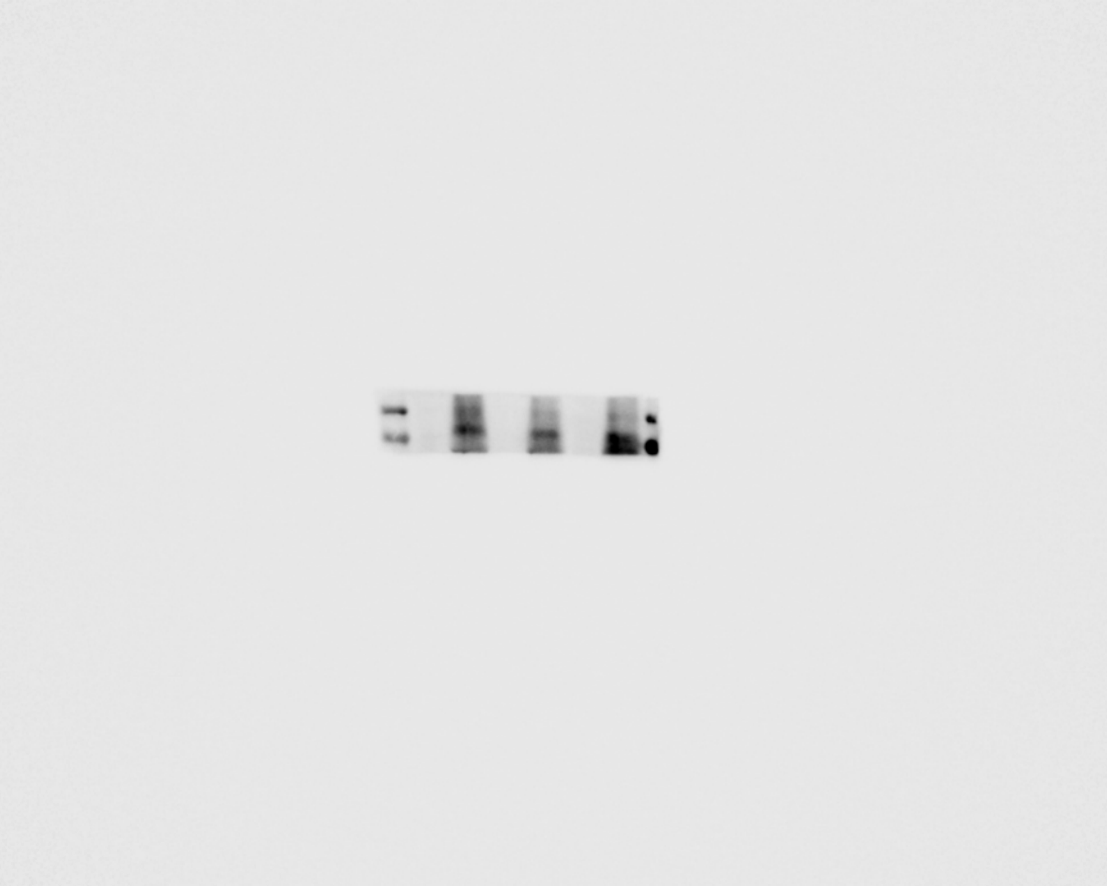

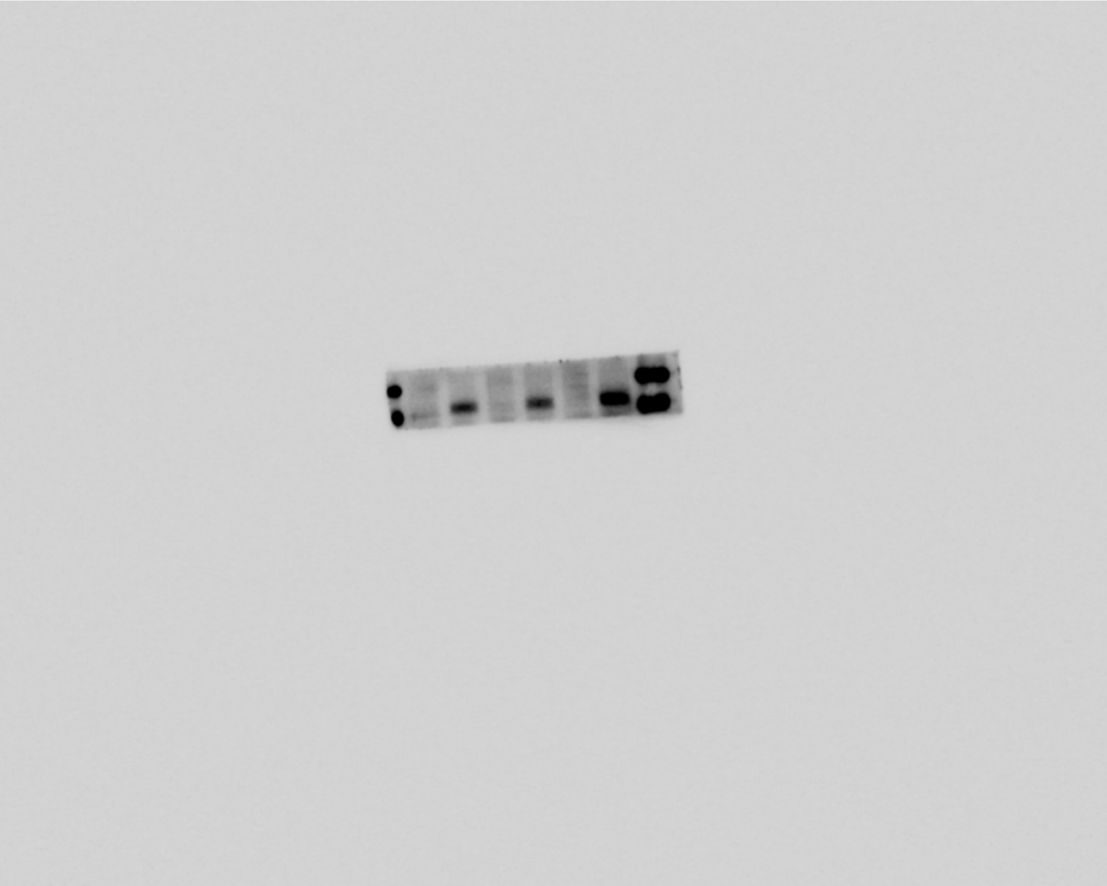

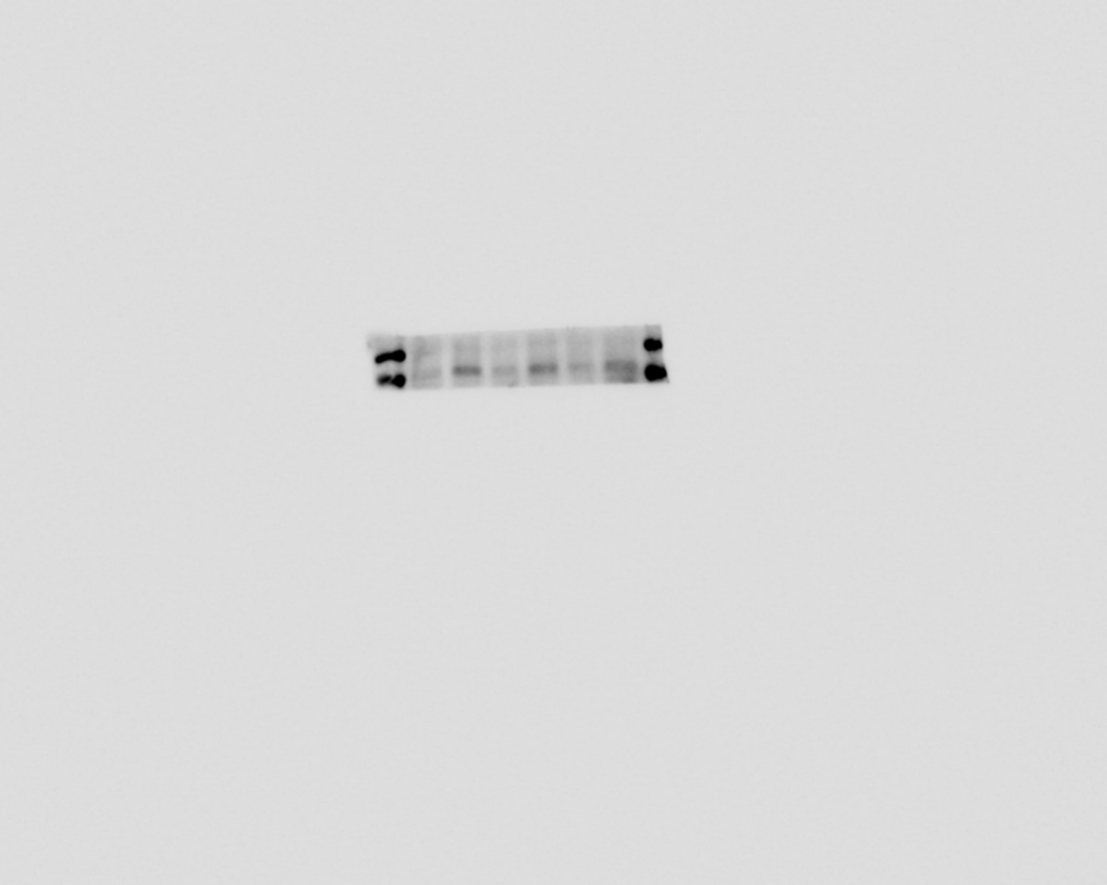

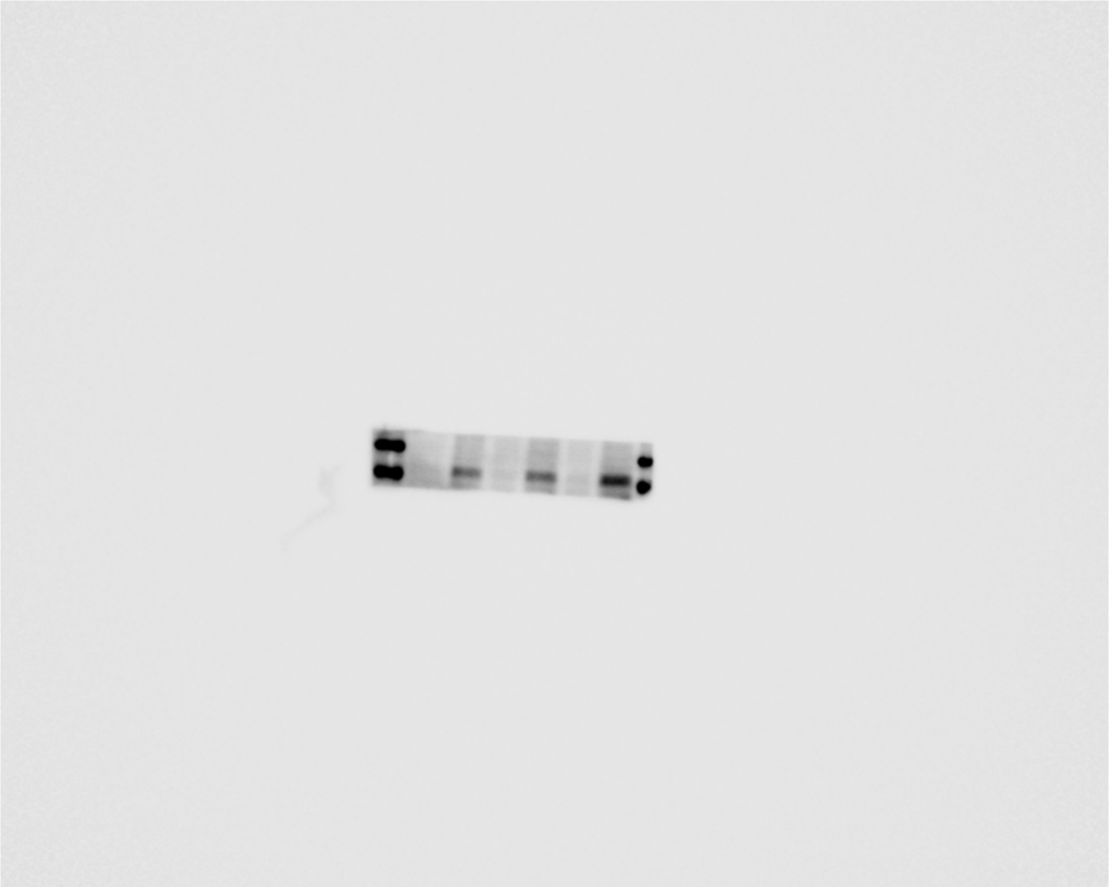

Fig. S1F
Actin

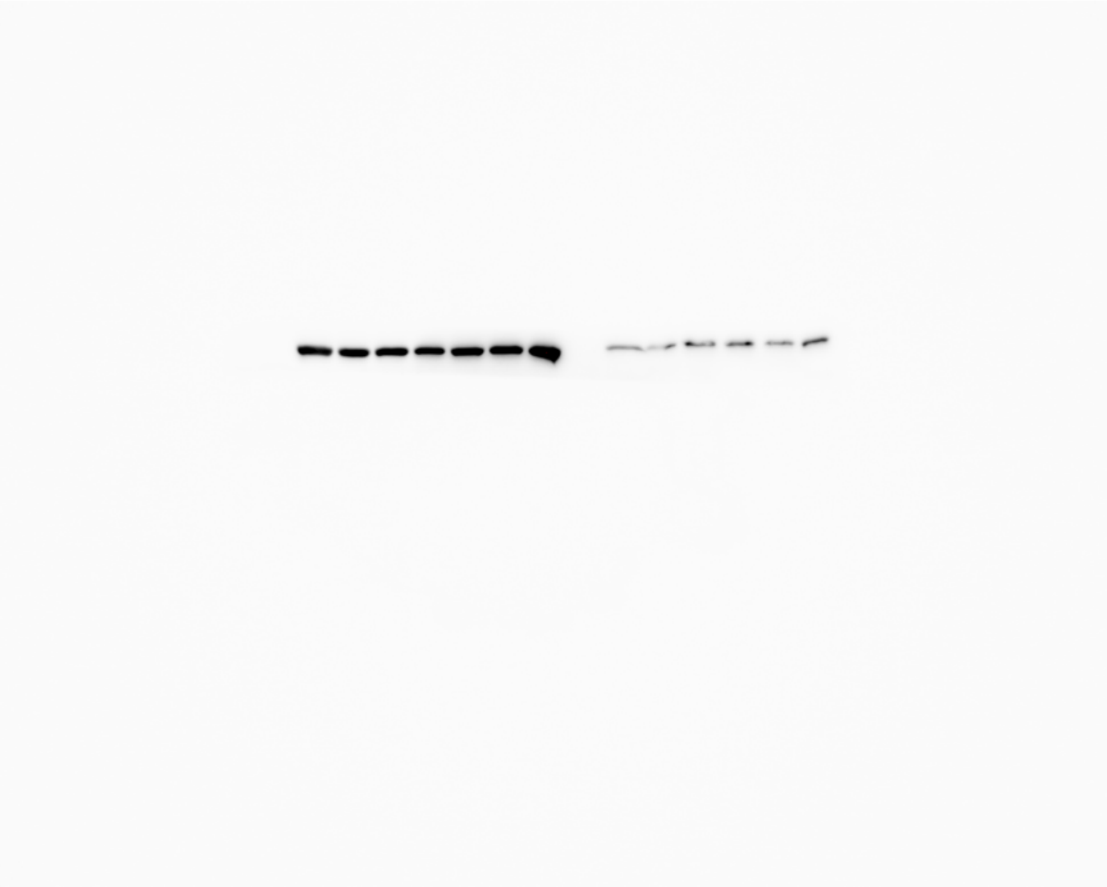

FKBP10

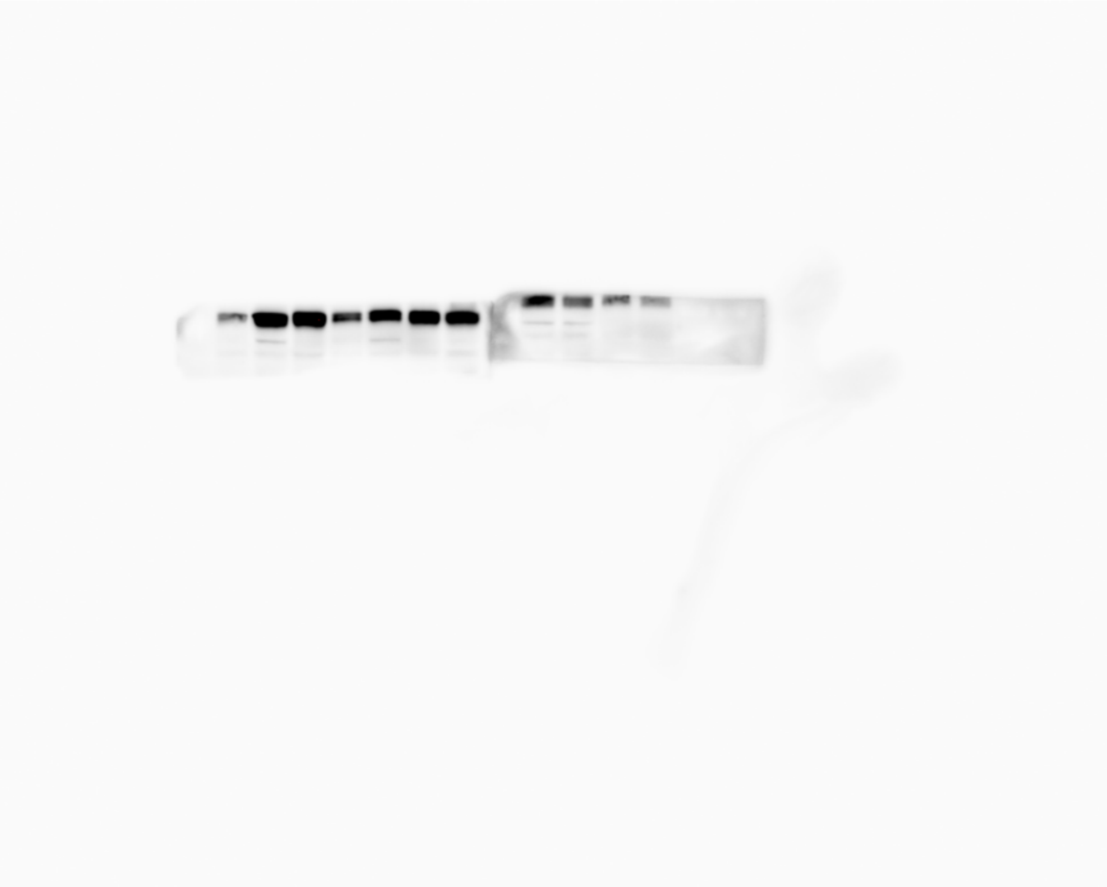

Fig. S2A

Actin

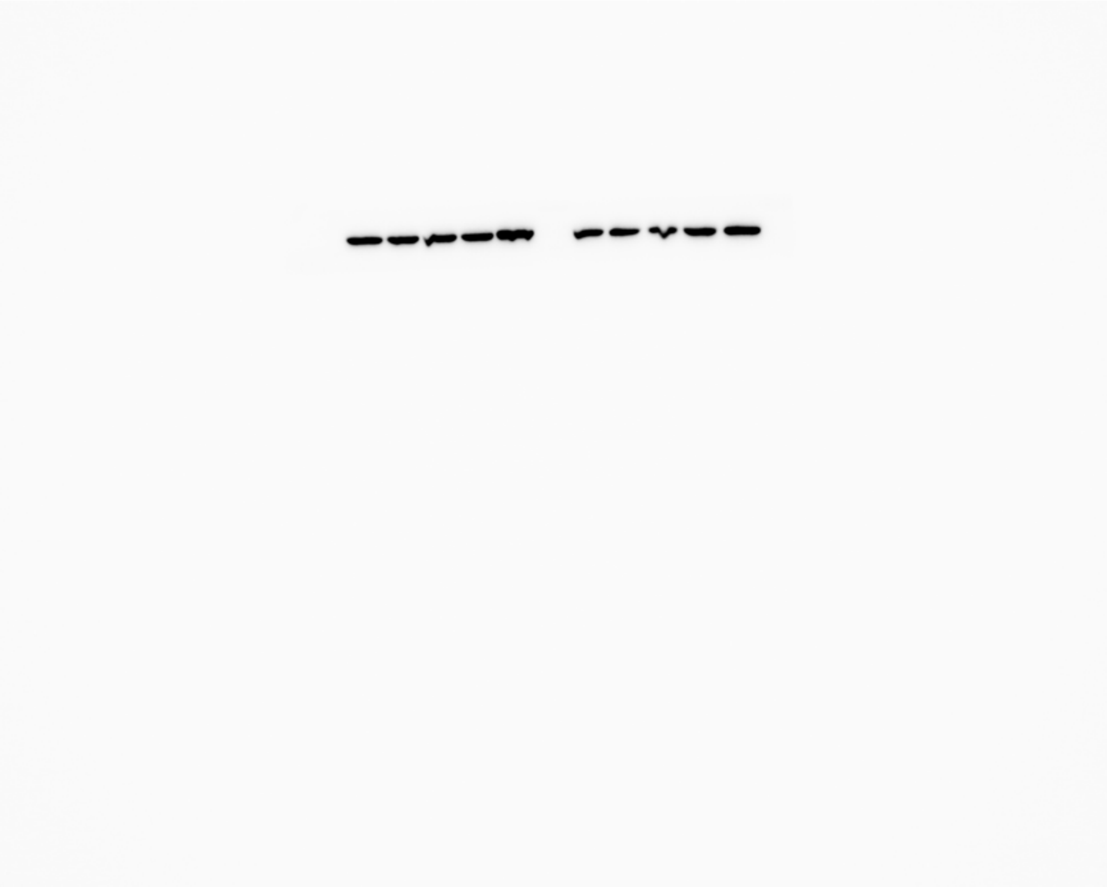

FKBP10

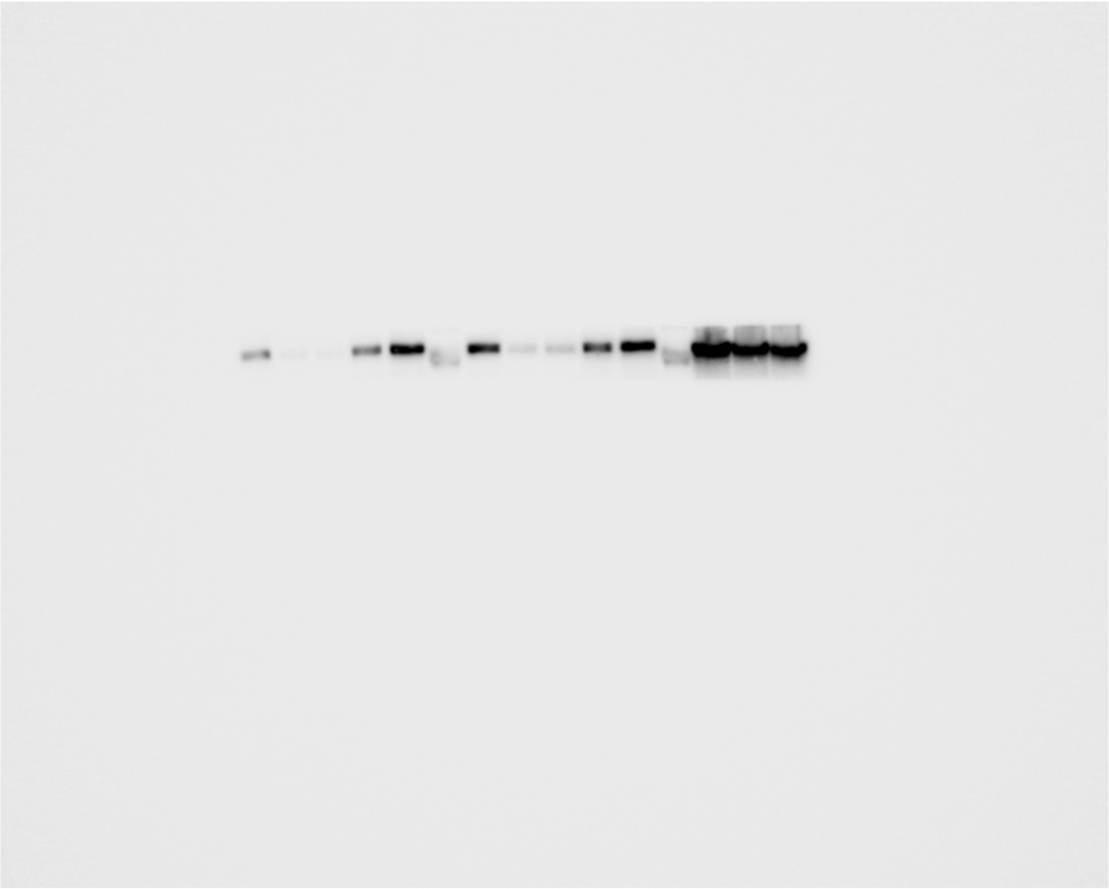

Fig. S2J
Actin

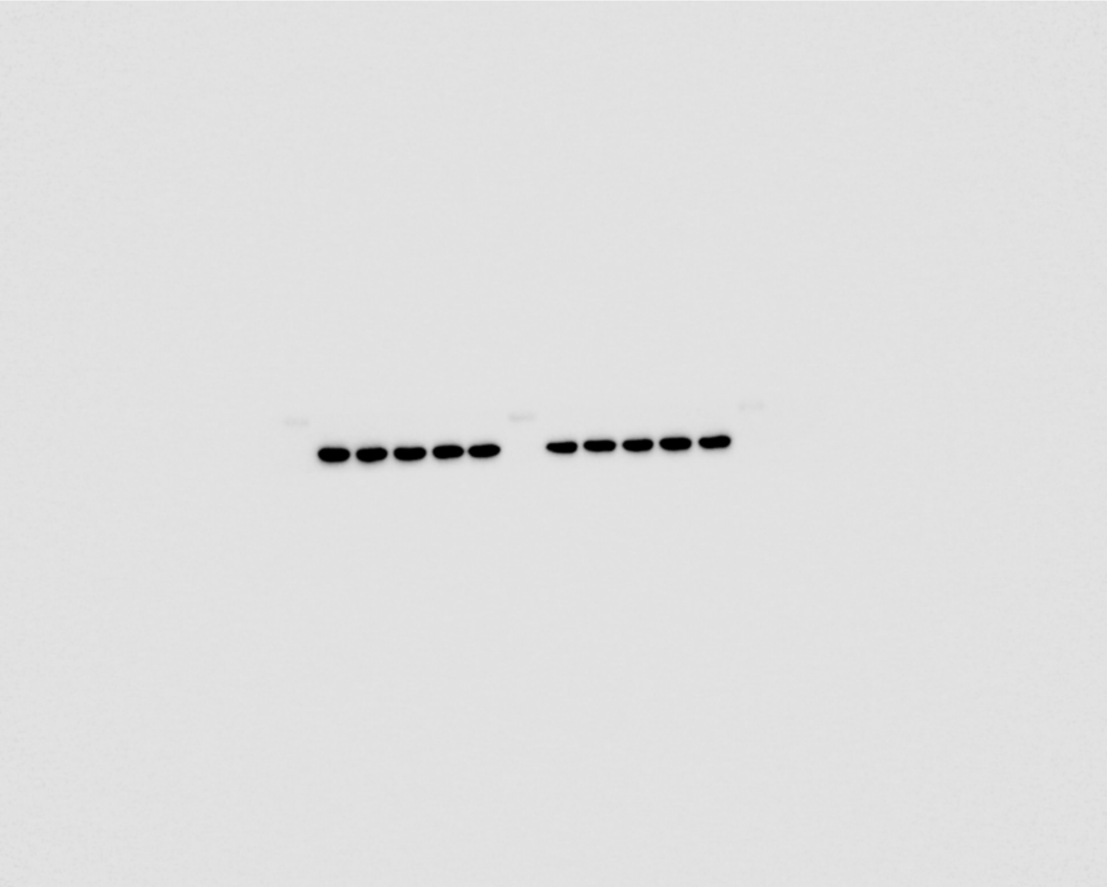

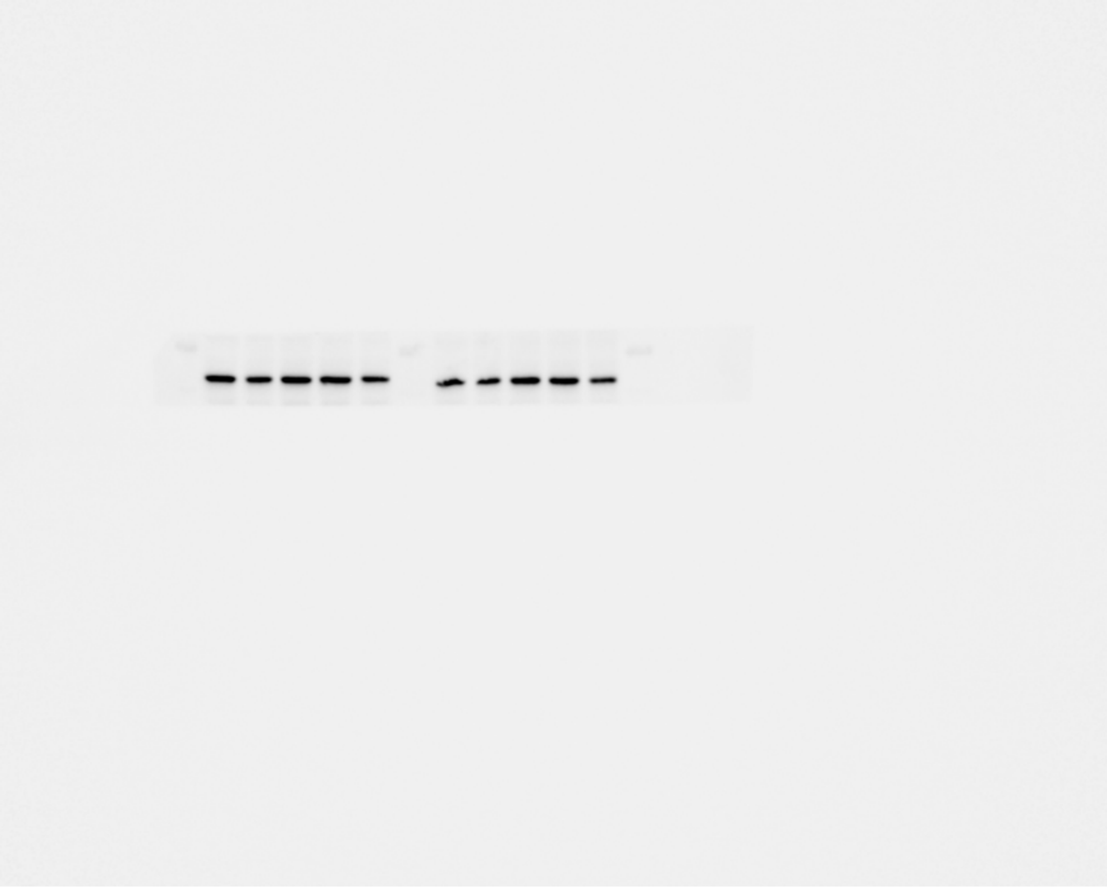

Vimentin

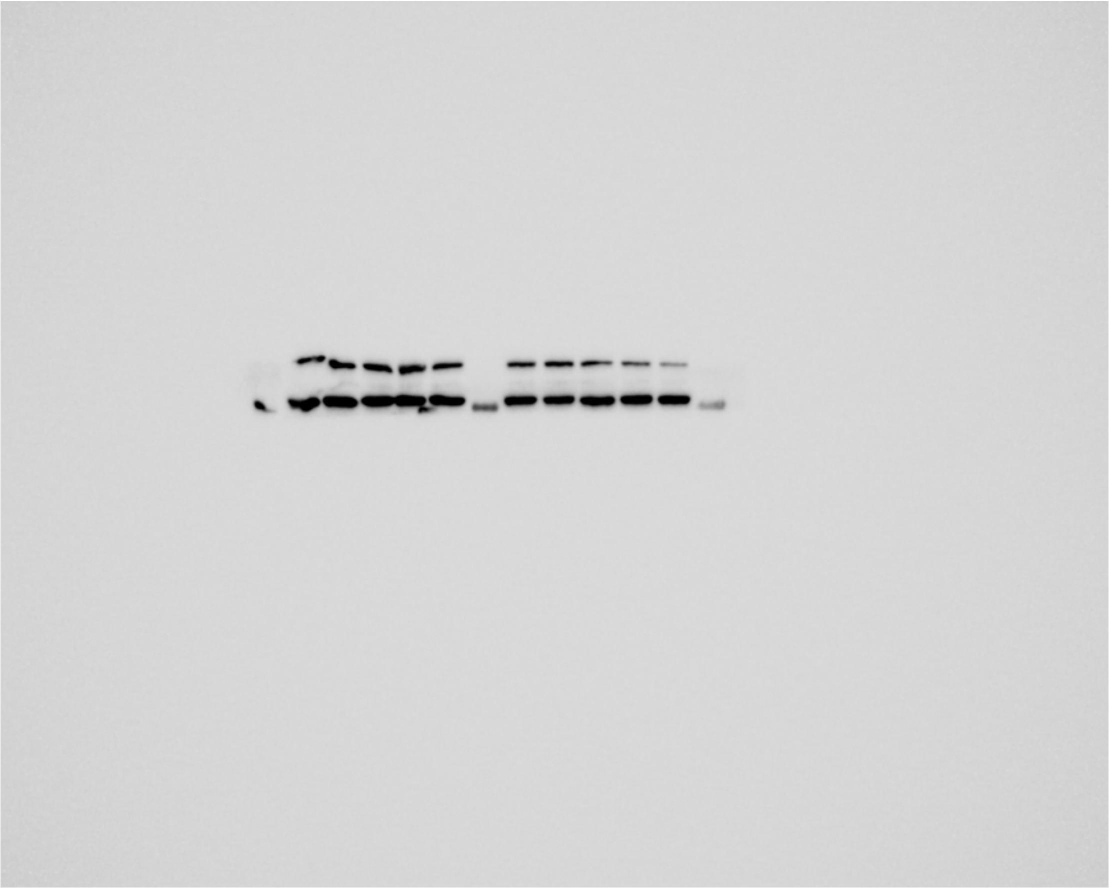

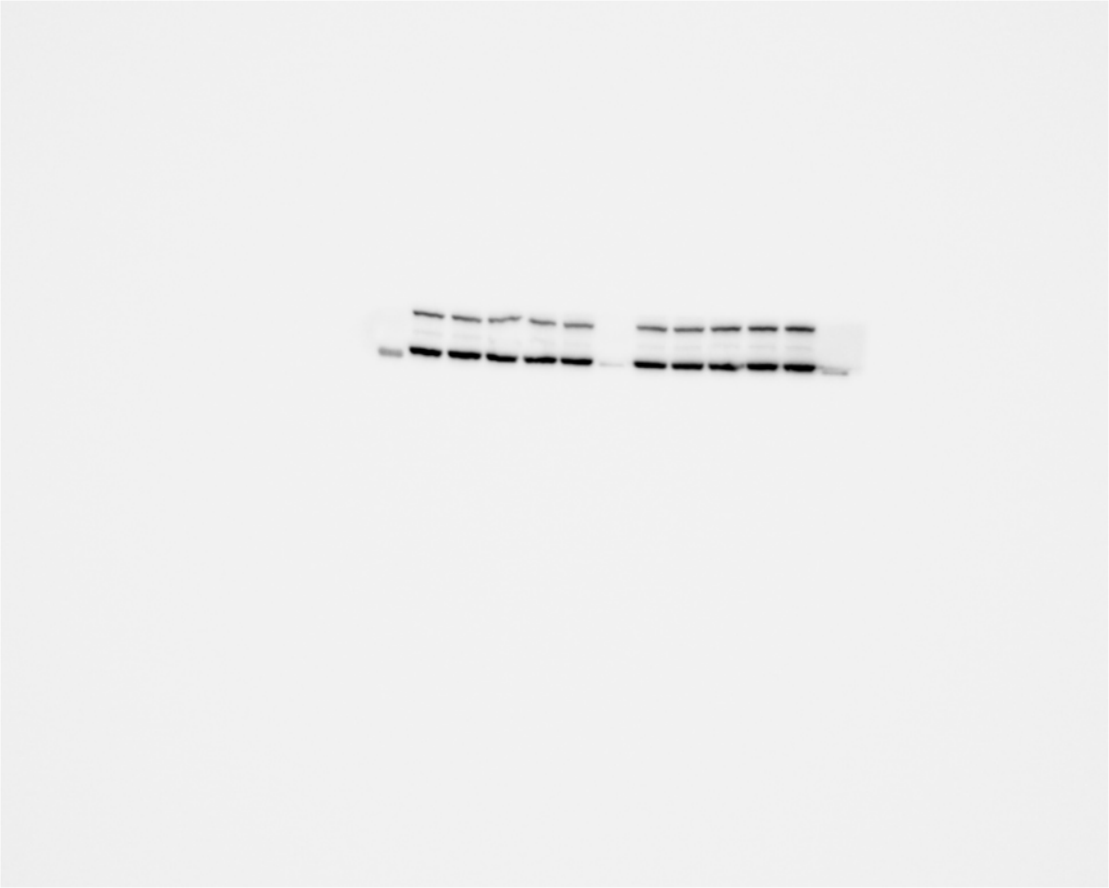

N-cad

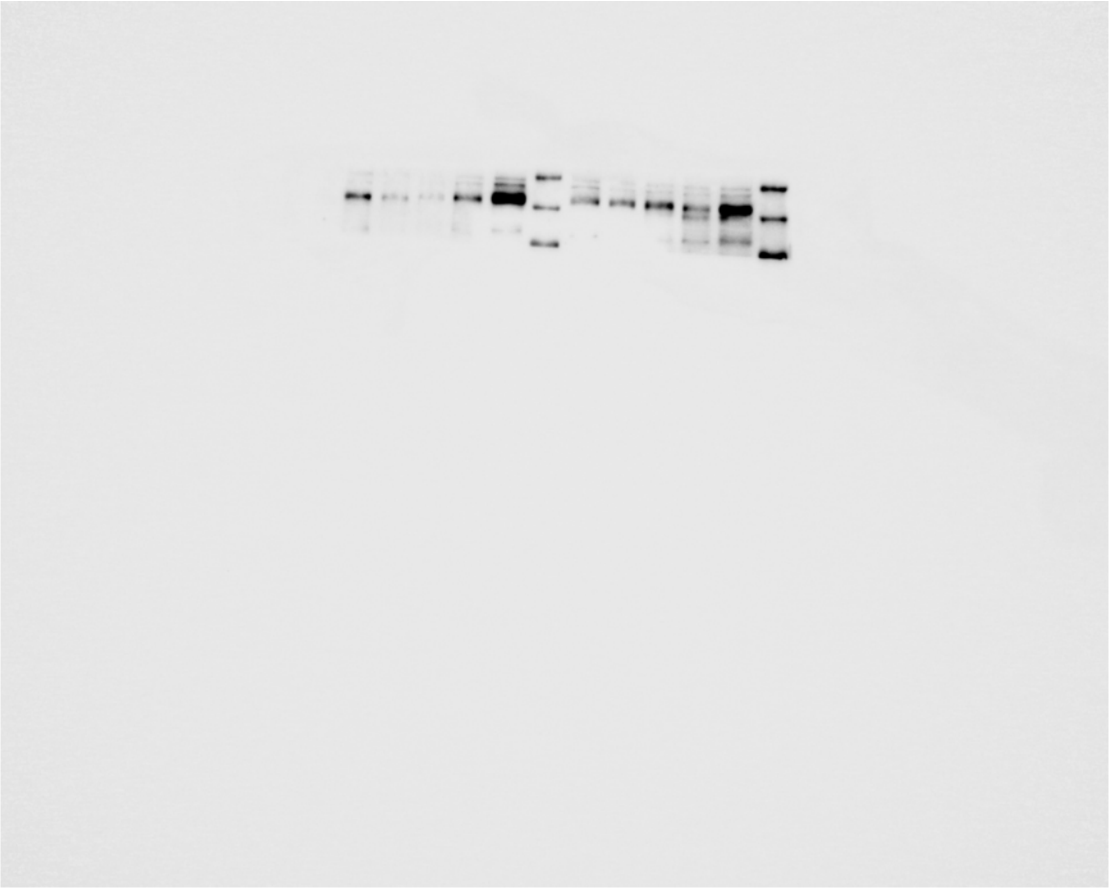

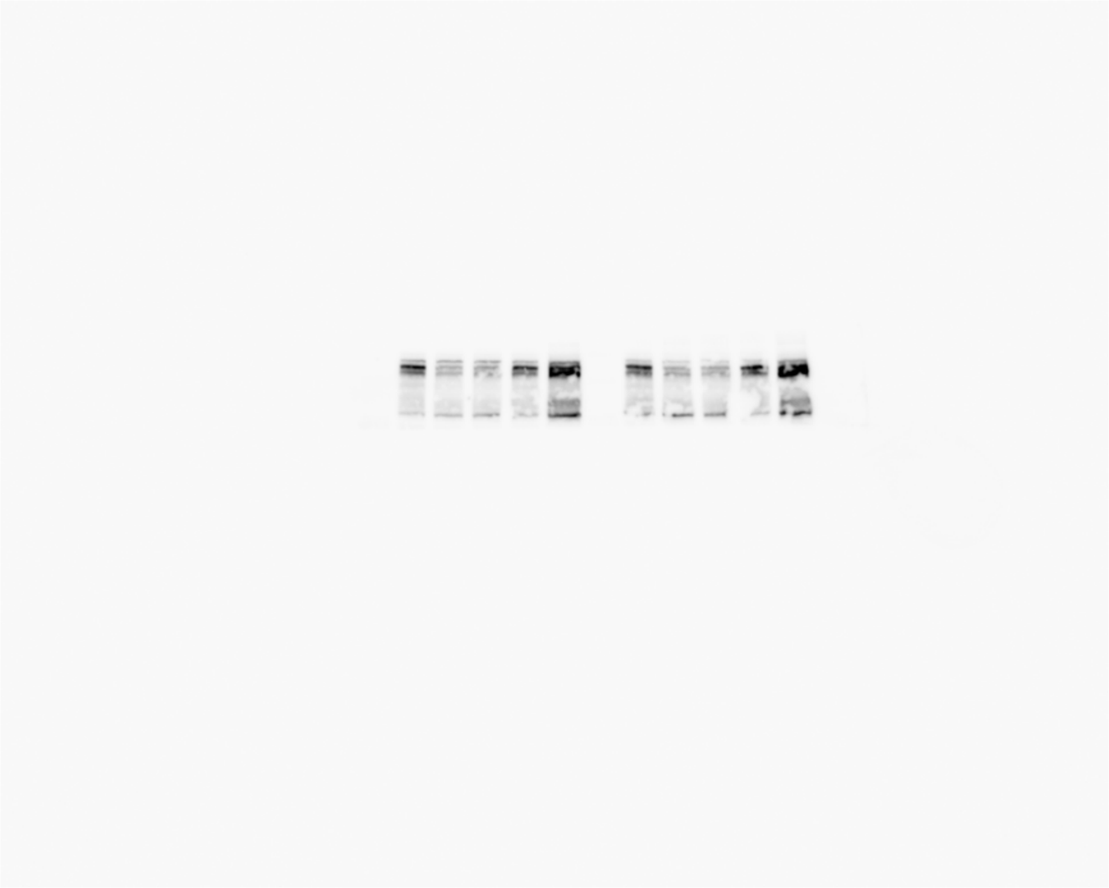

E-cad

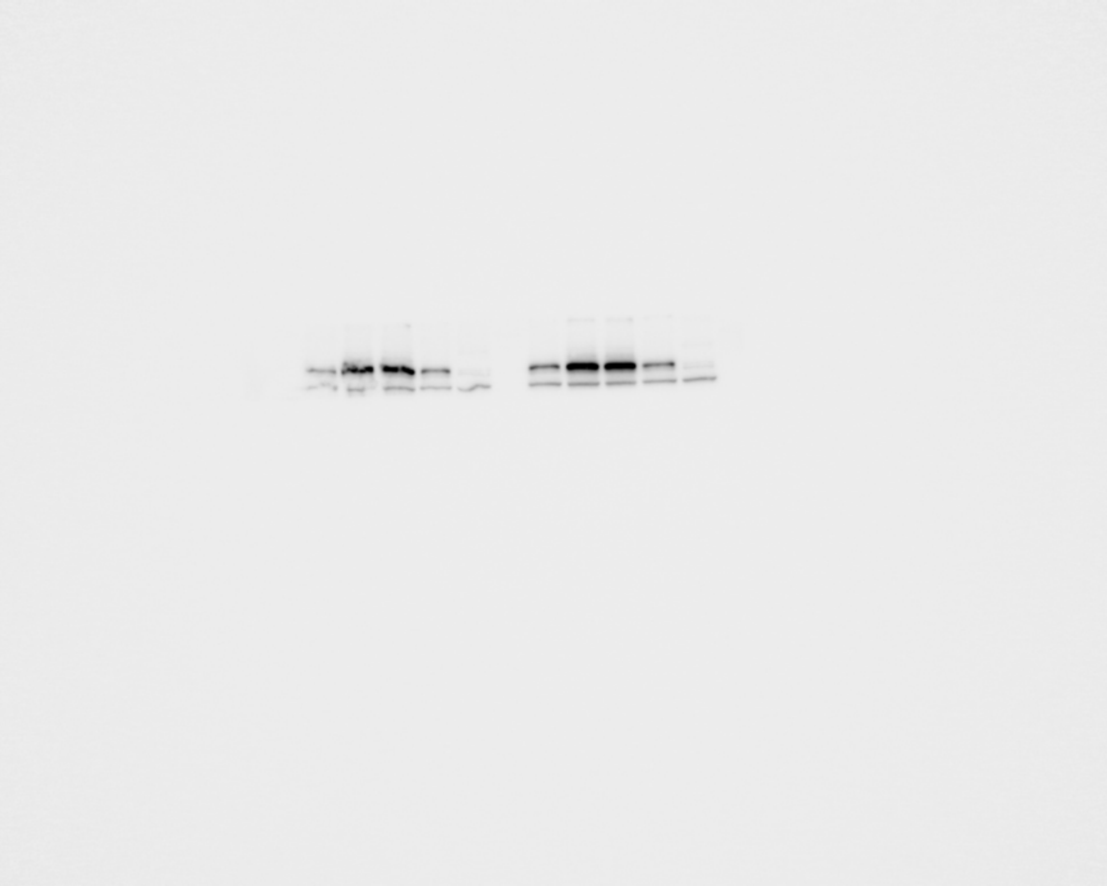

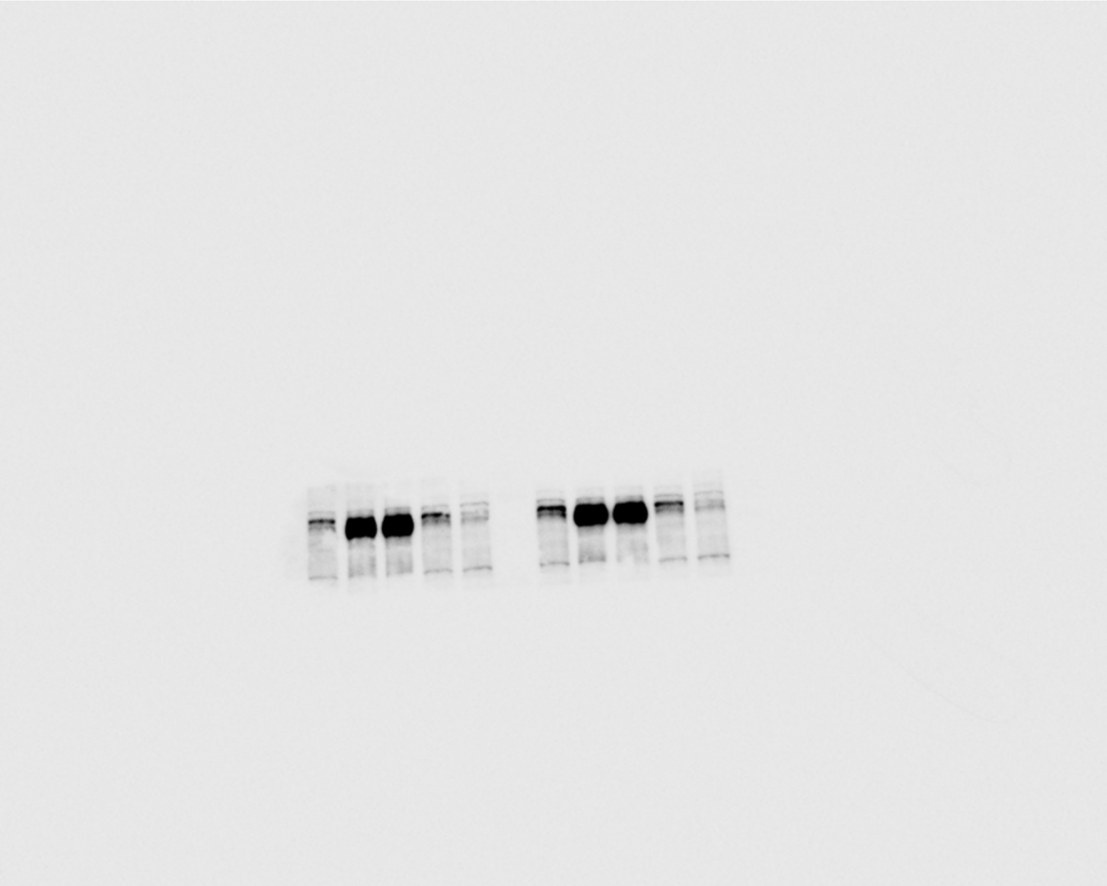

Fig. S4A

Actin

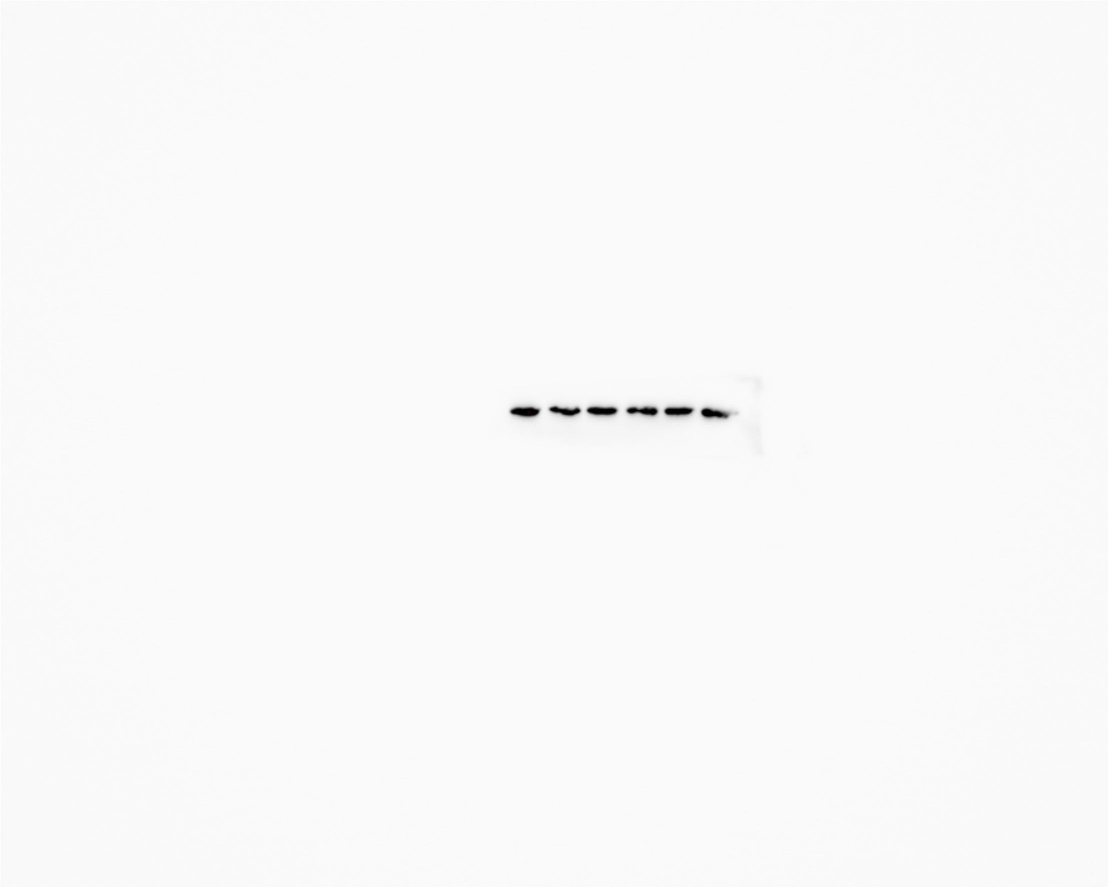

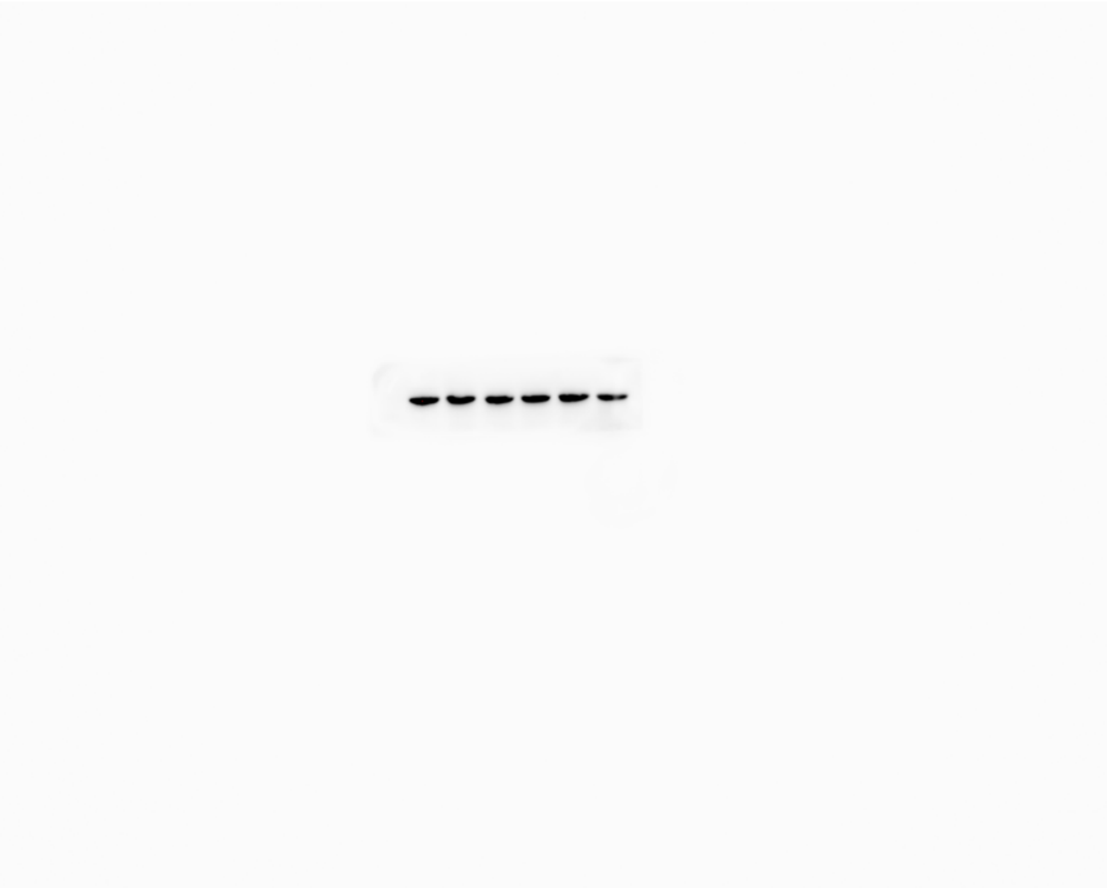

LDHA

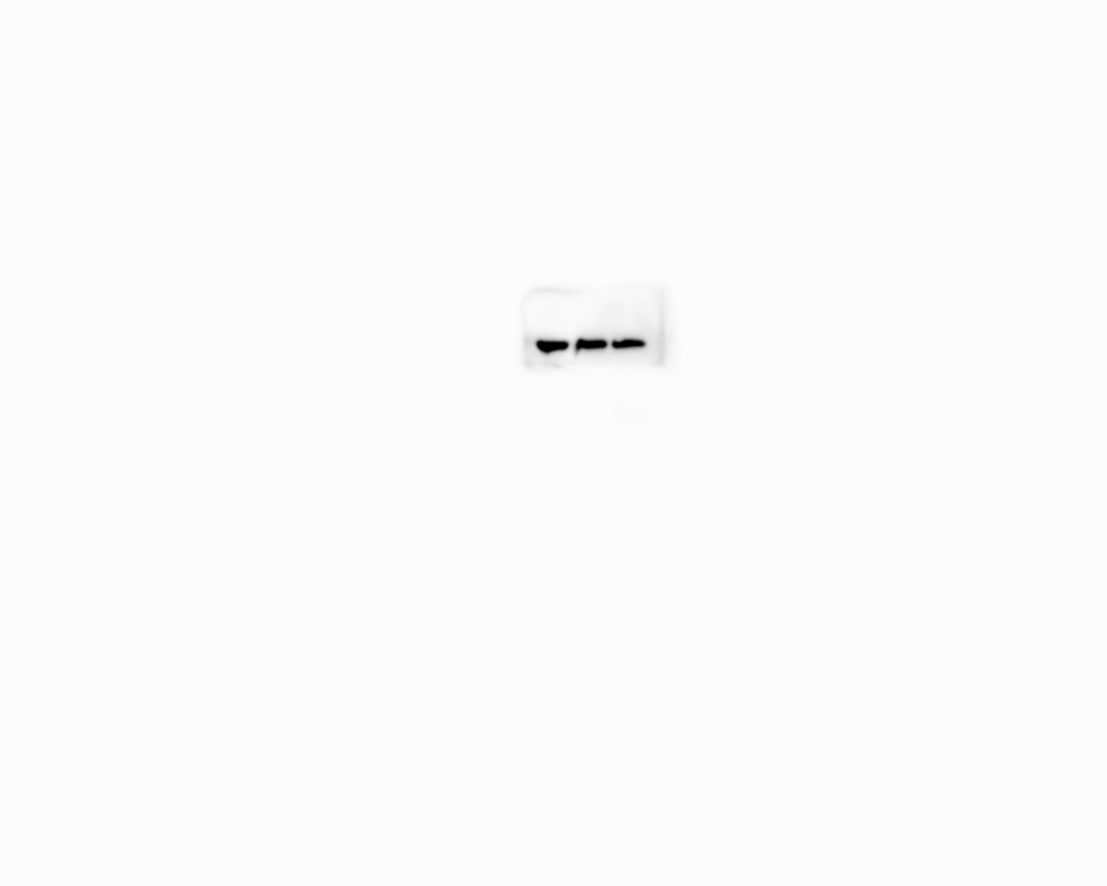

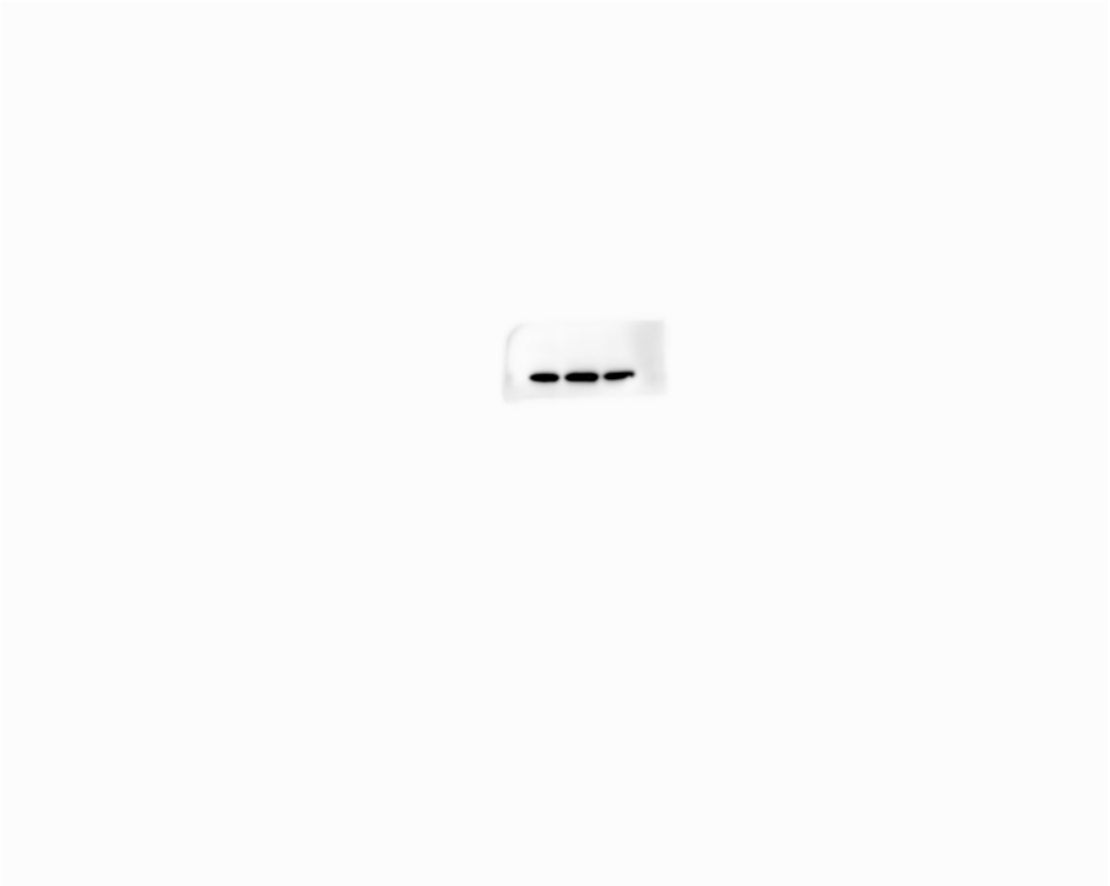

LDHB

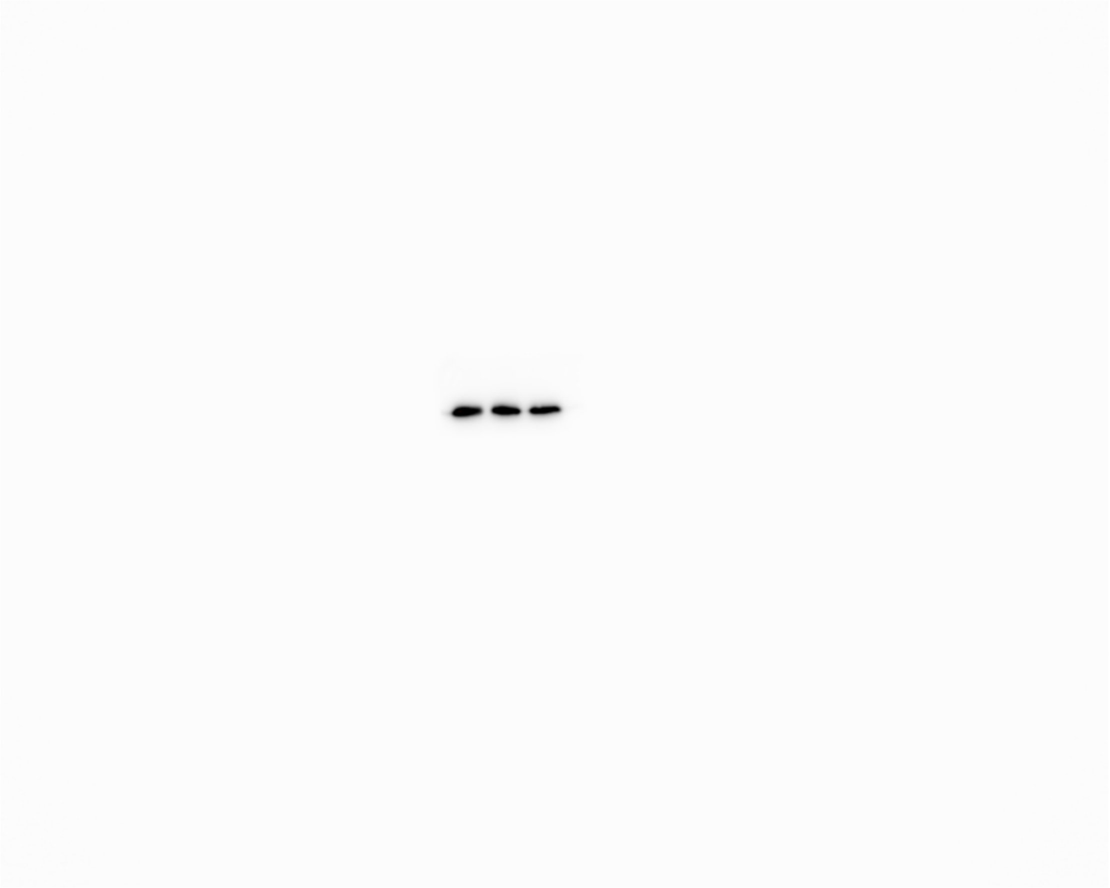

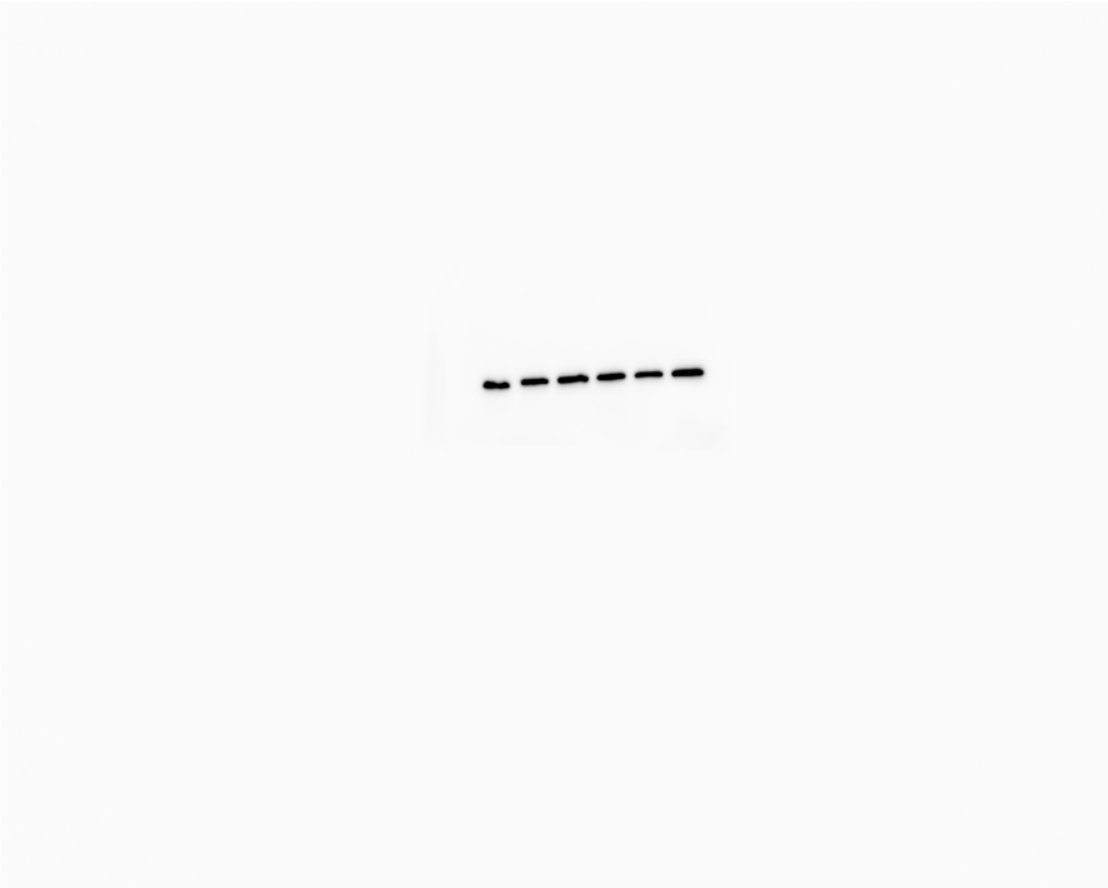

FKBP10

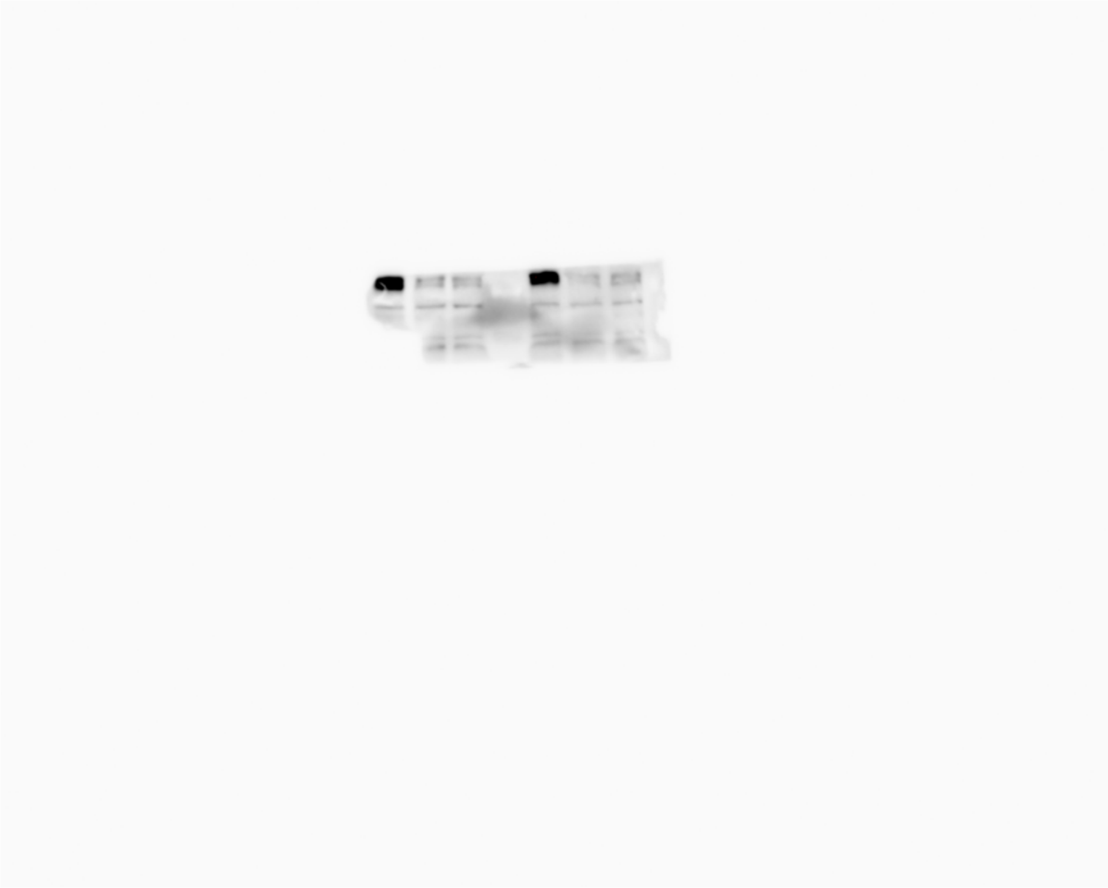

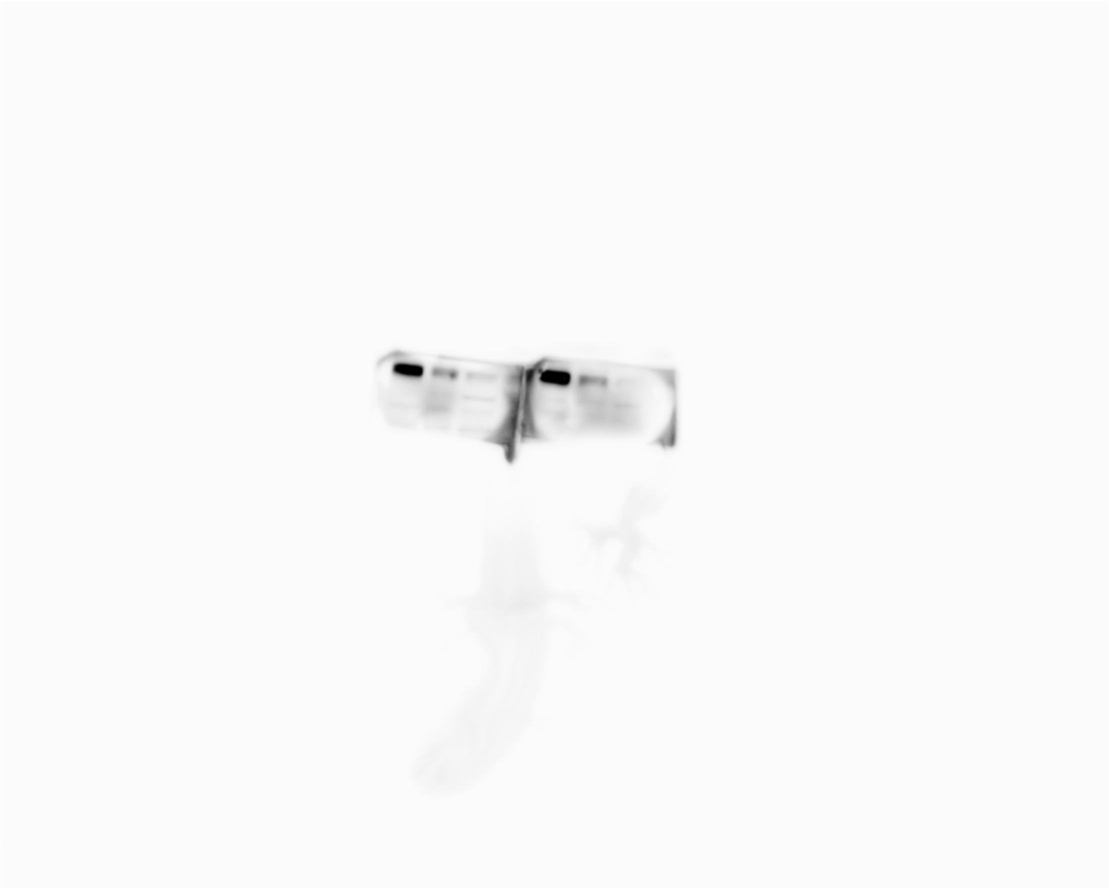

Fig. S5A

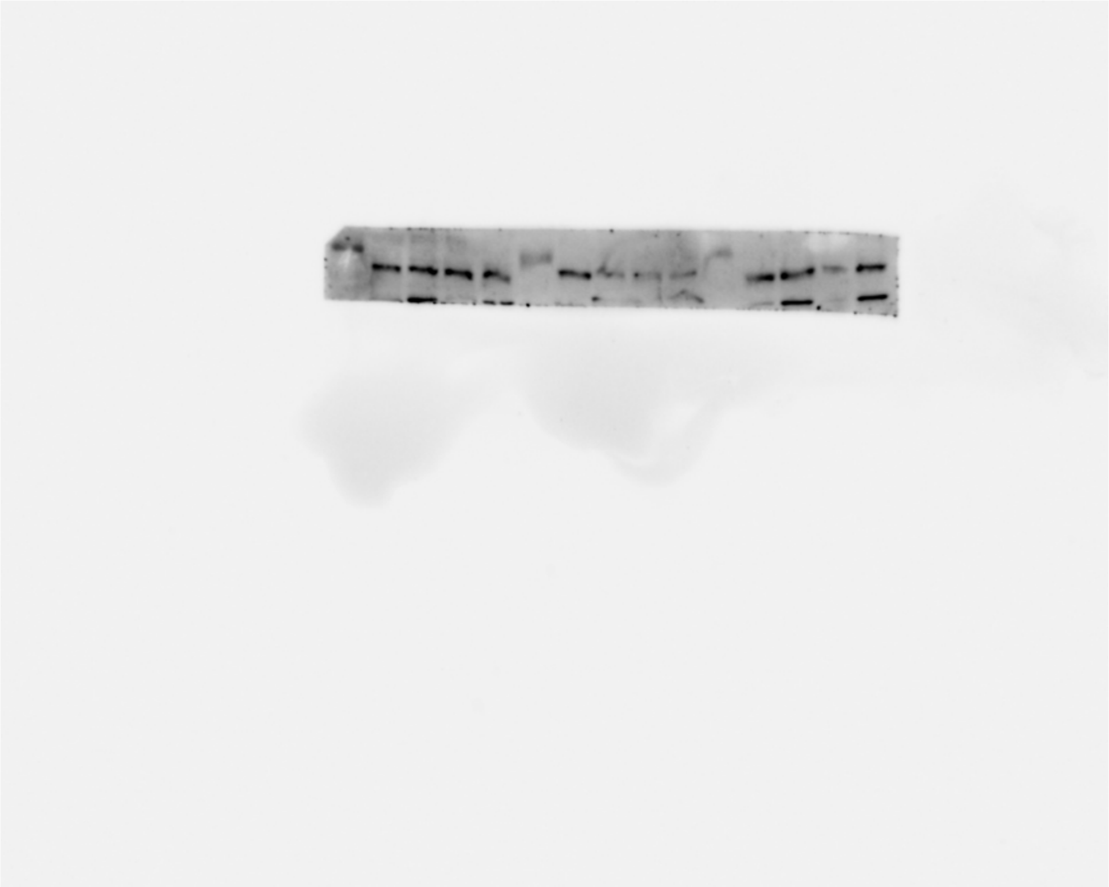

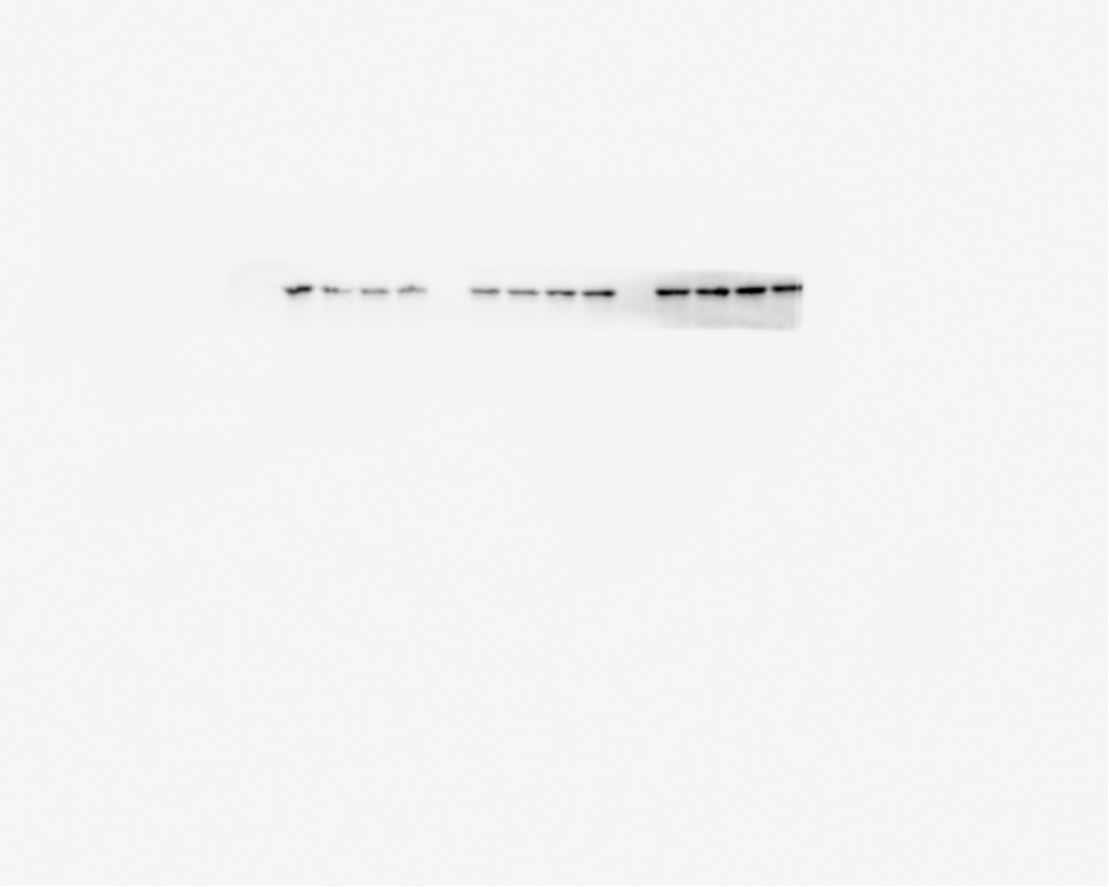

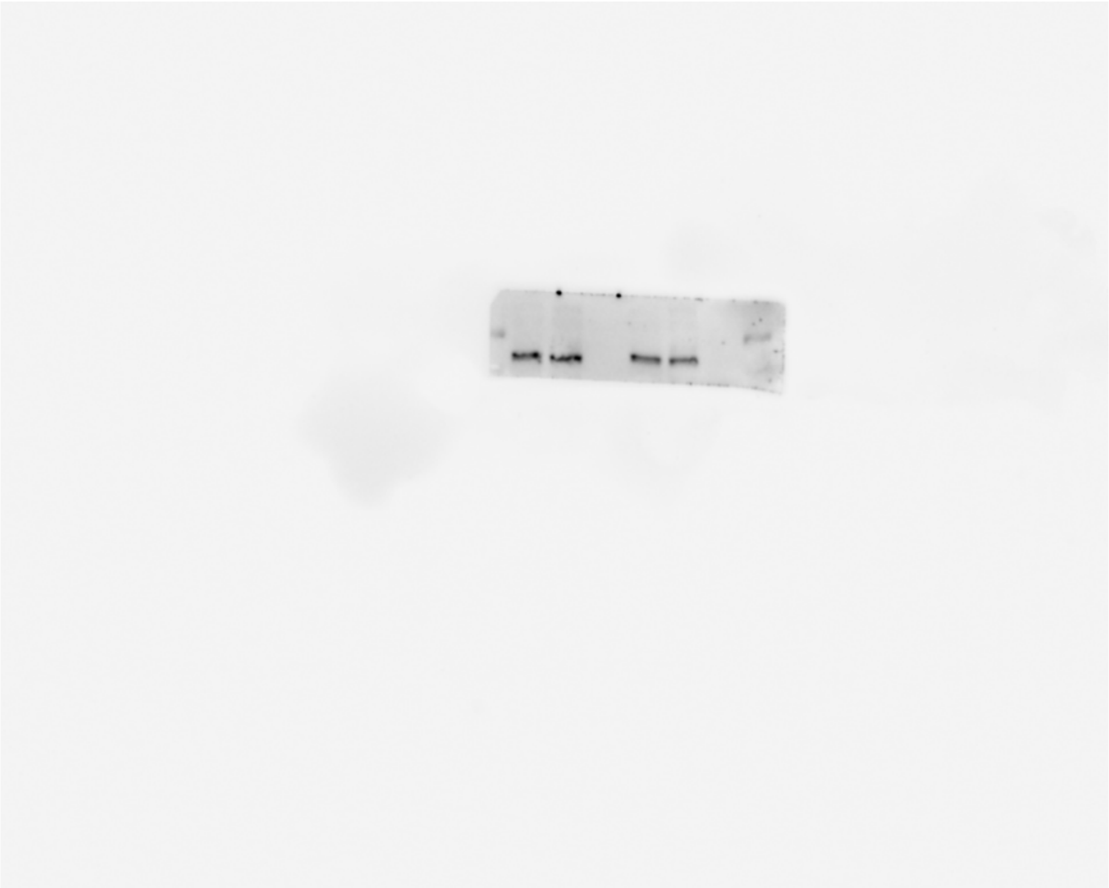

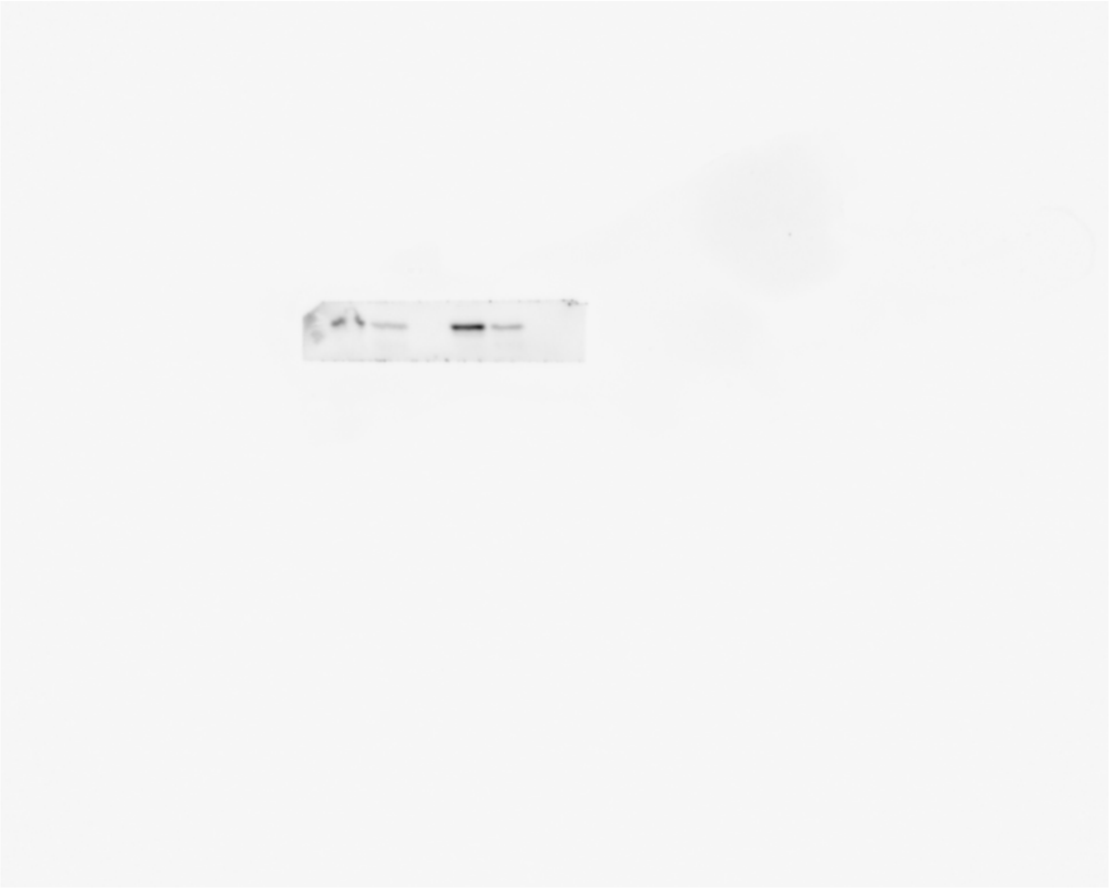

Fig. S6A

Actin

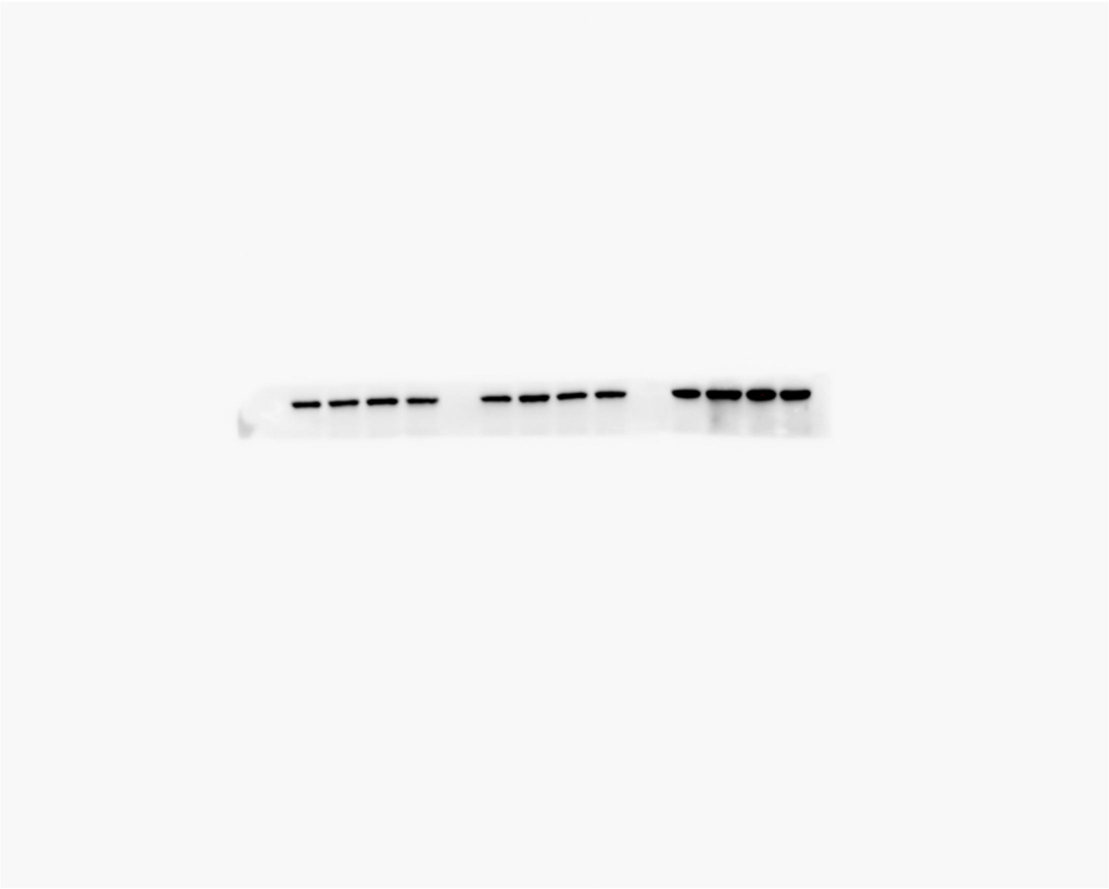

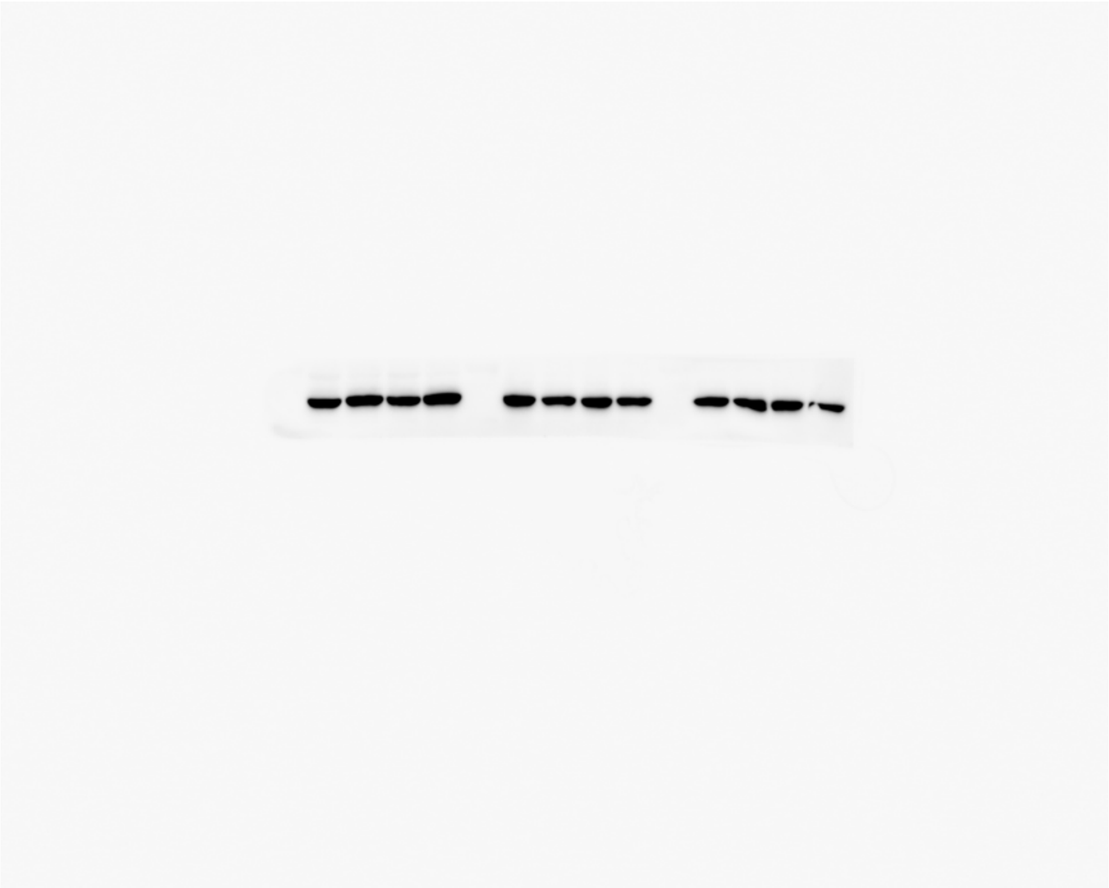

FKBP10

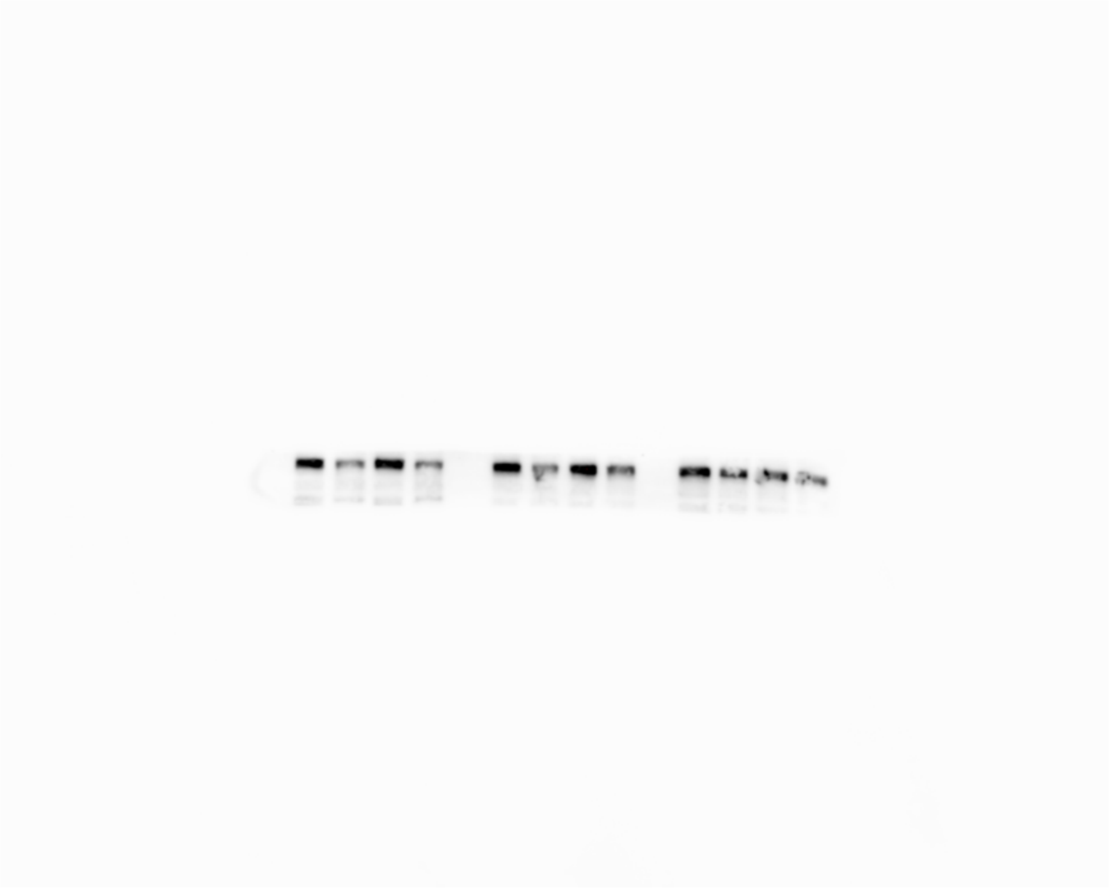

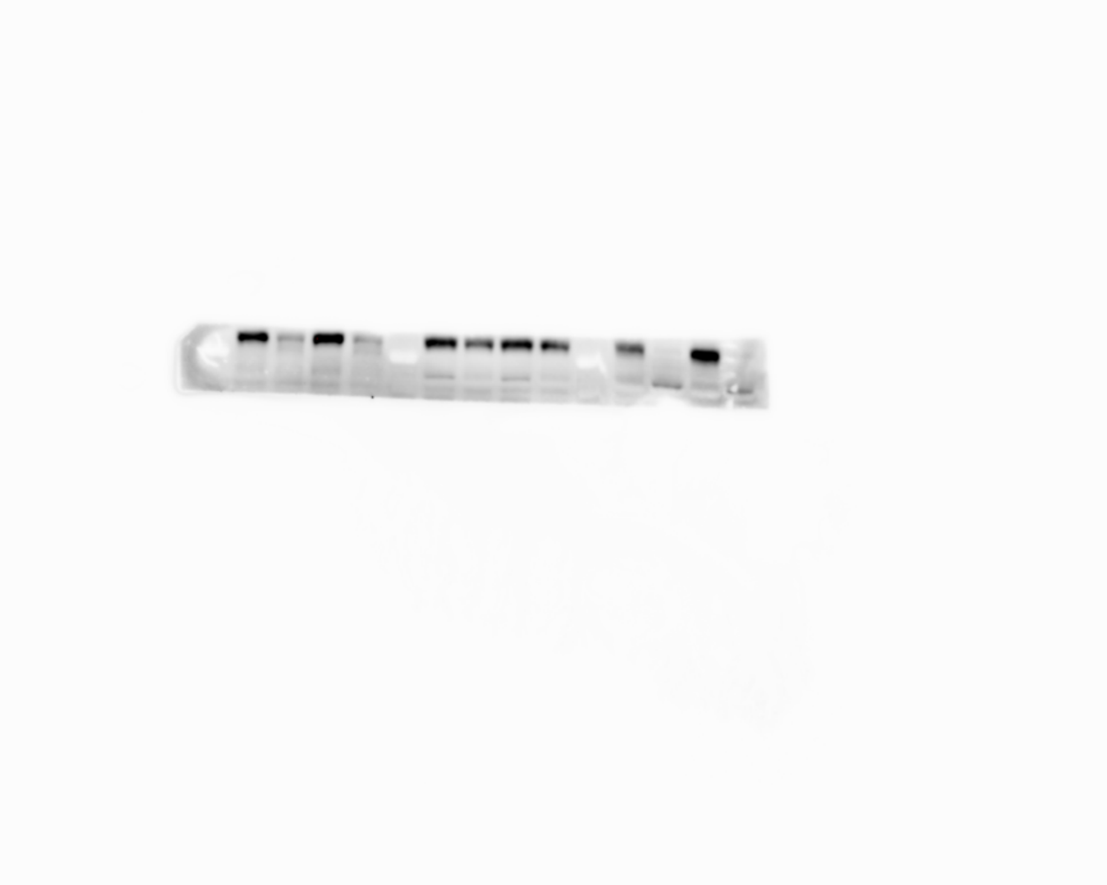

HIF1a

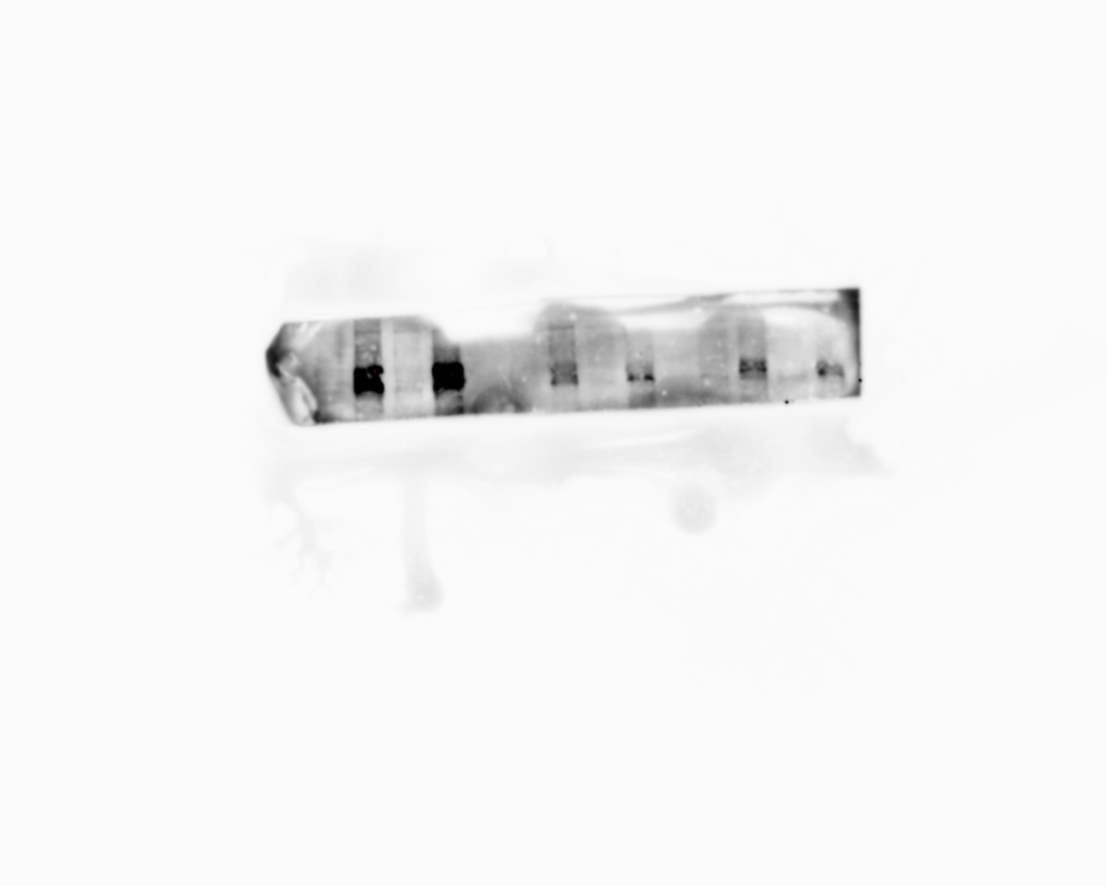

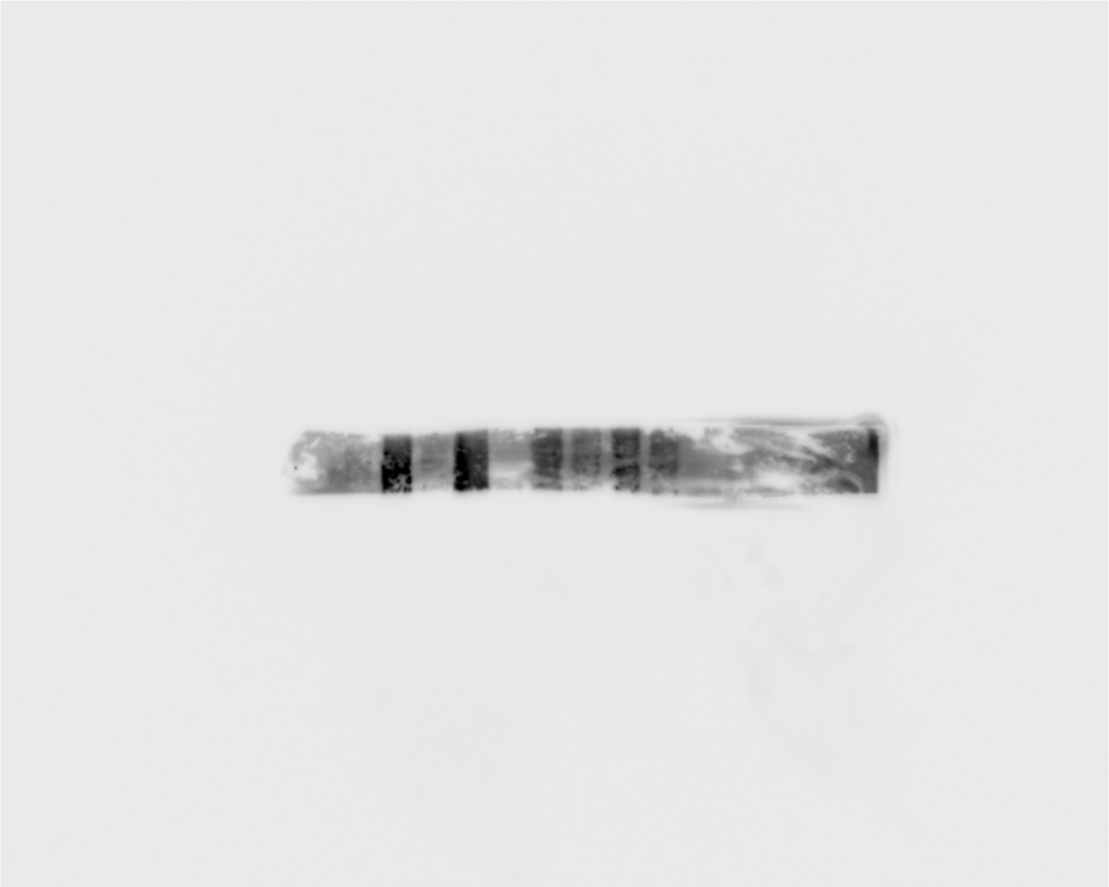

HIF2a

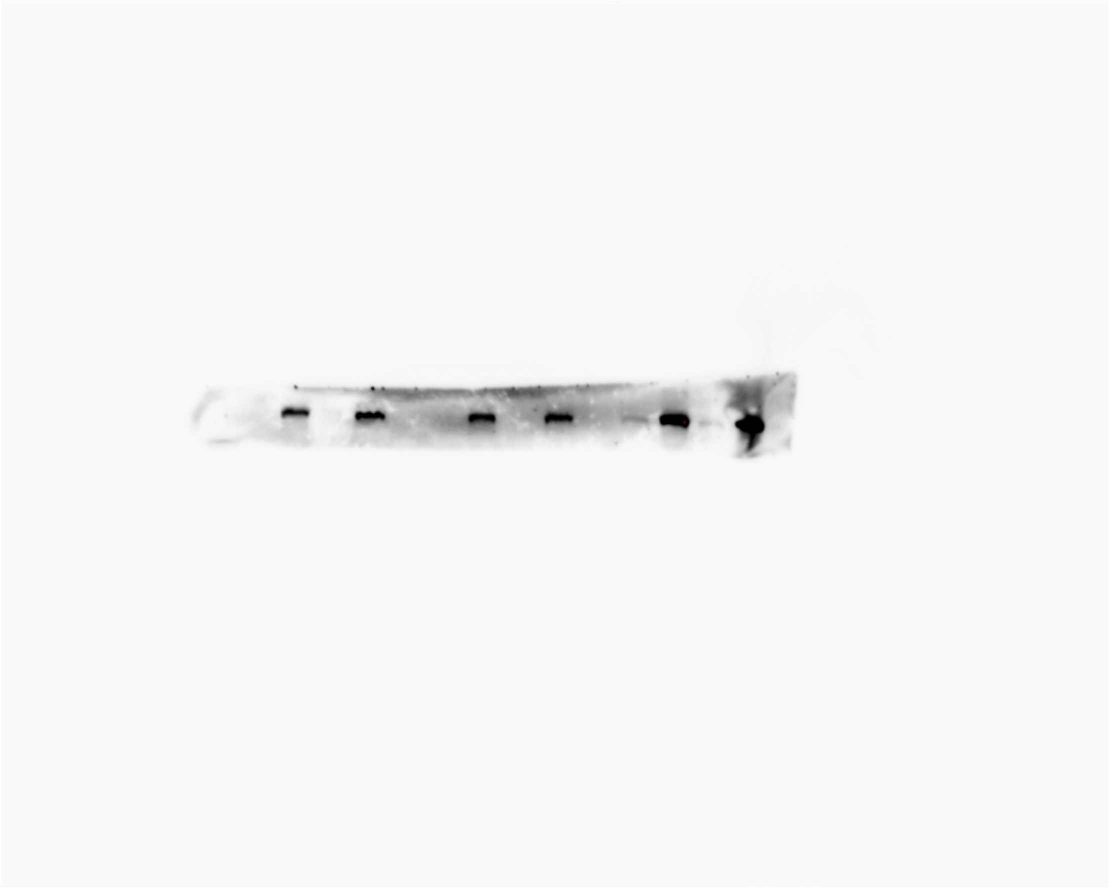

Fig. S7B

Actin

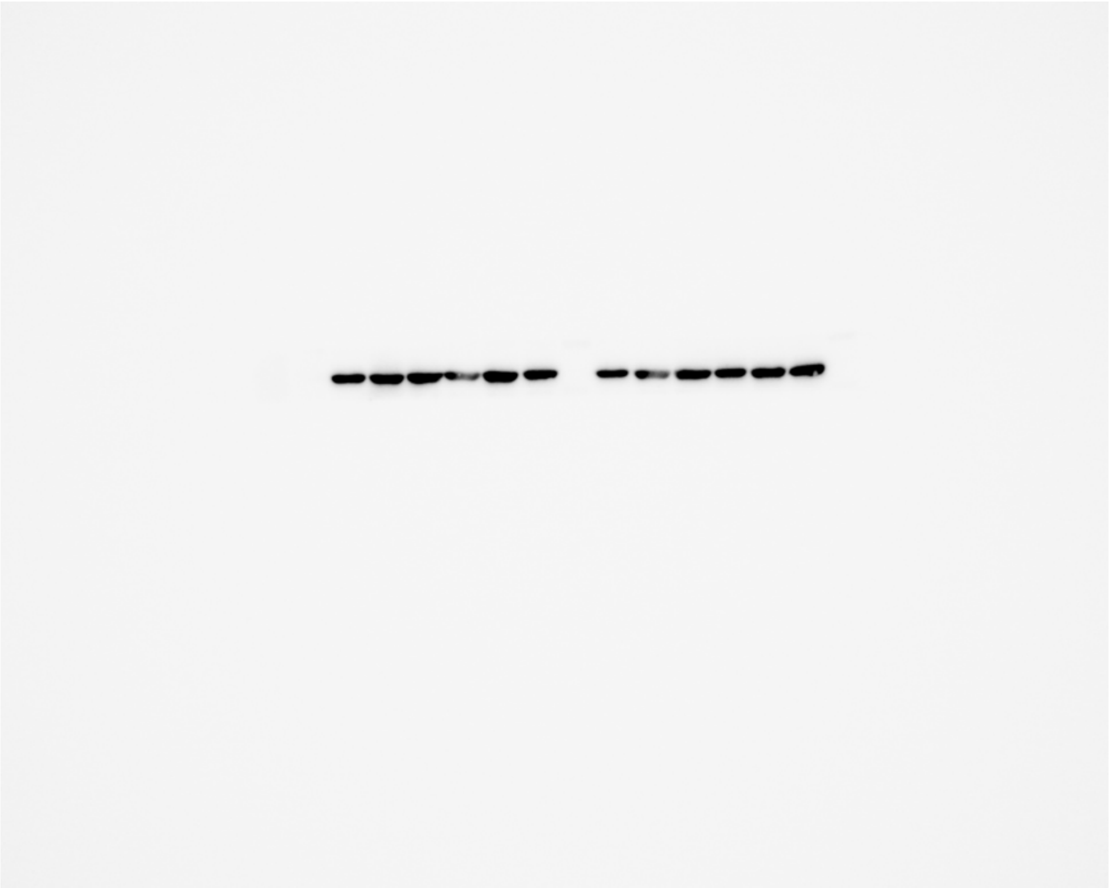

FKBP10

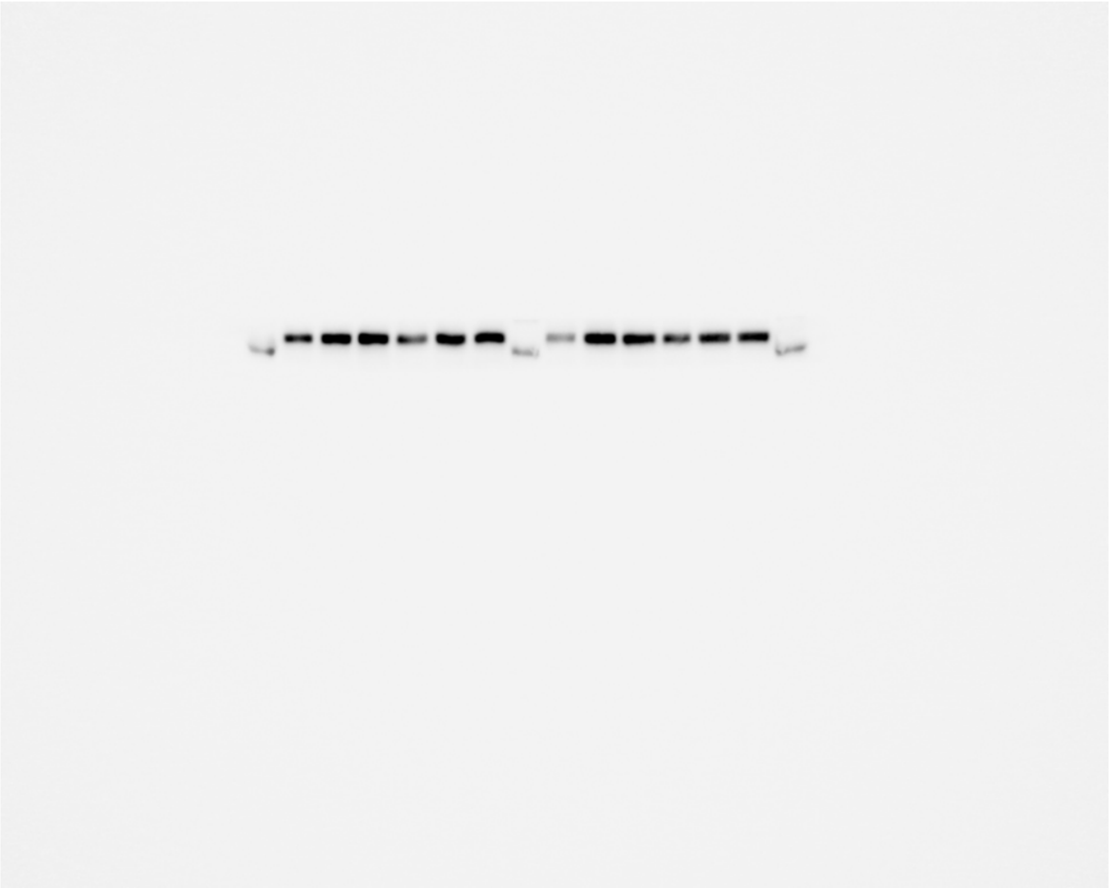

HIF1a

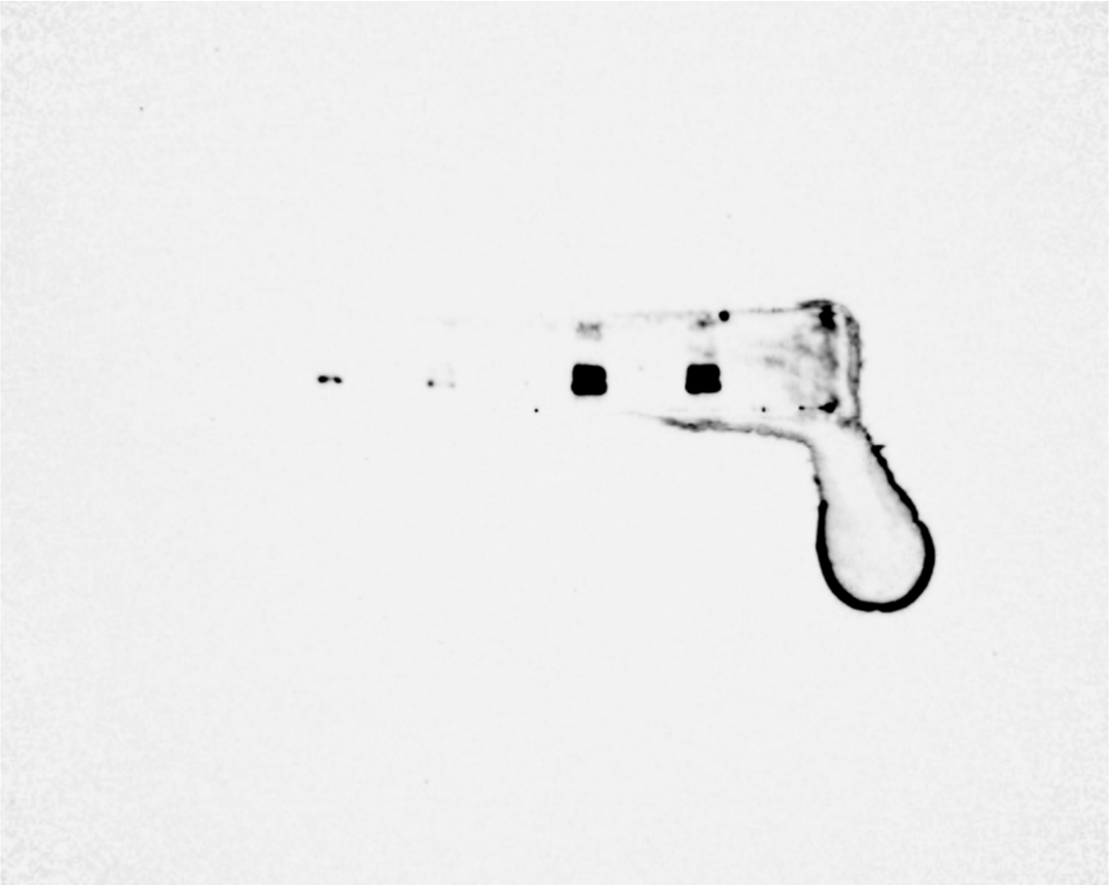

Fig. S7C

Actin

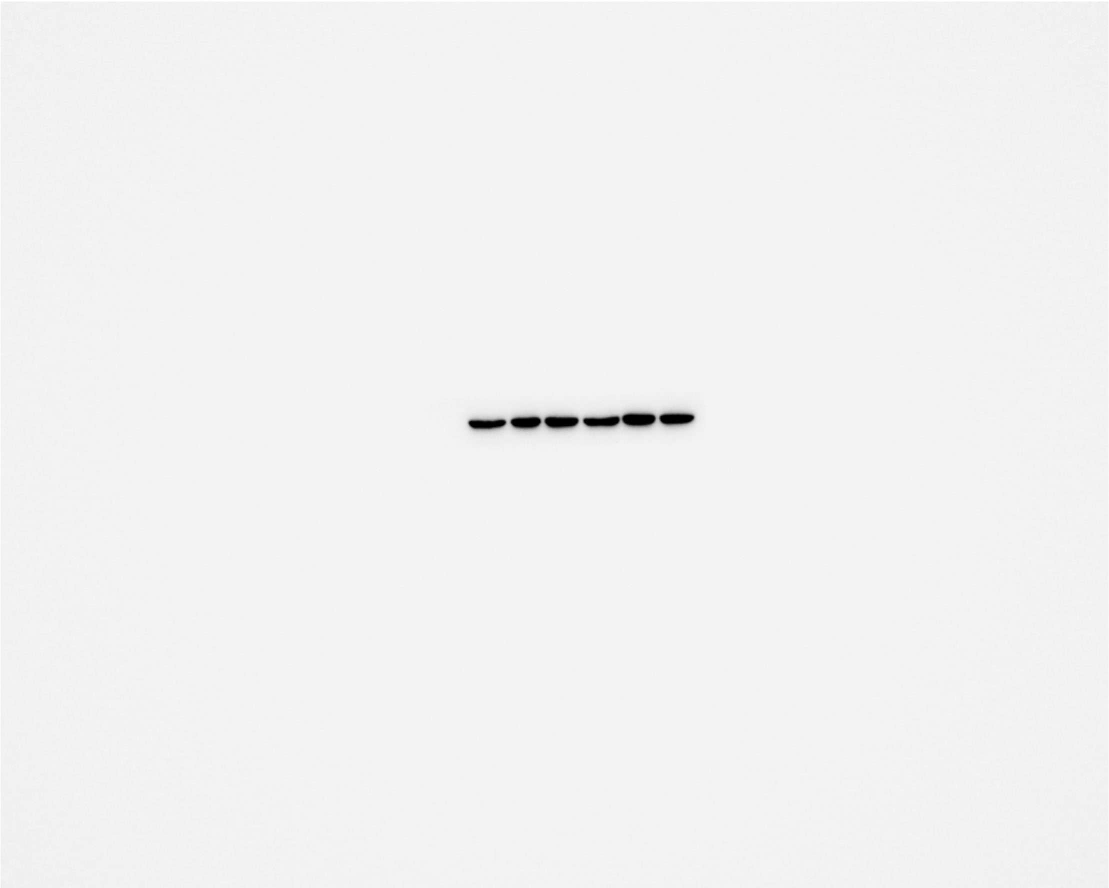

FKBP10

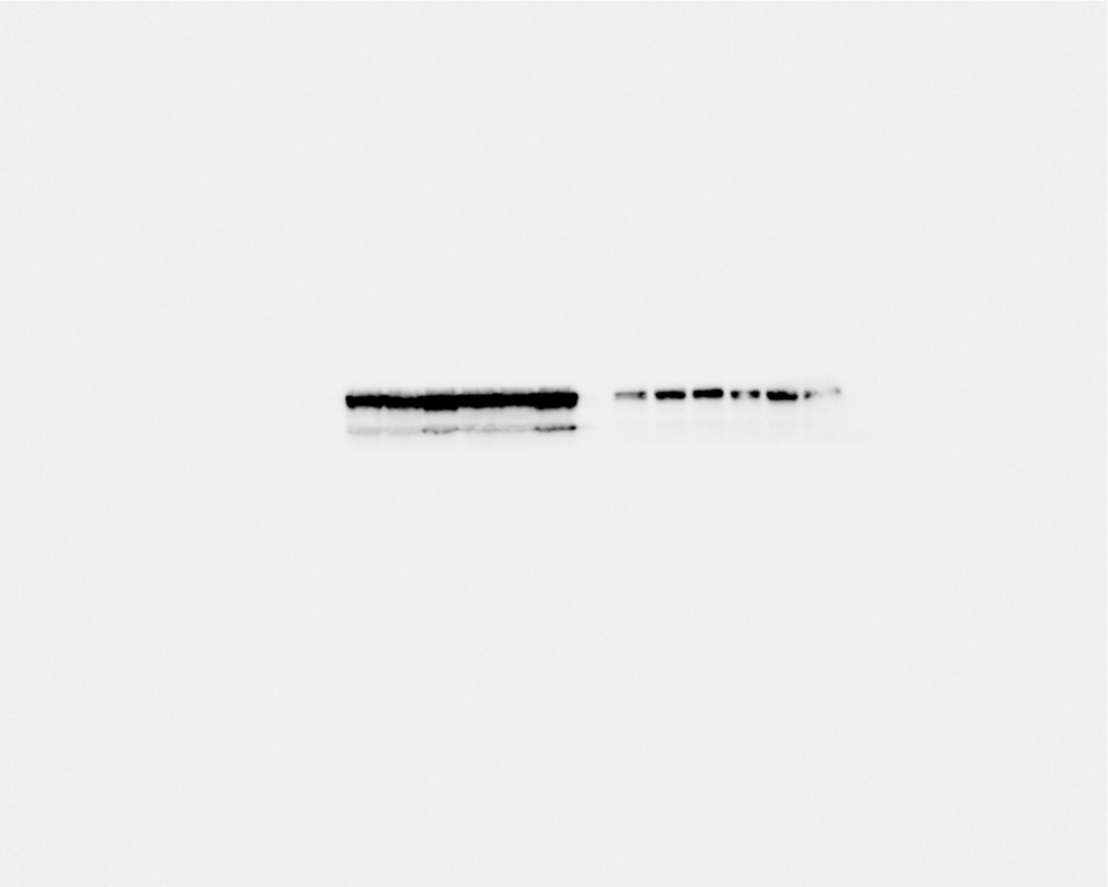

HIF1a

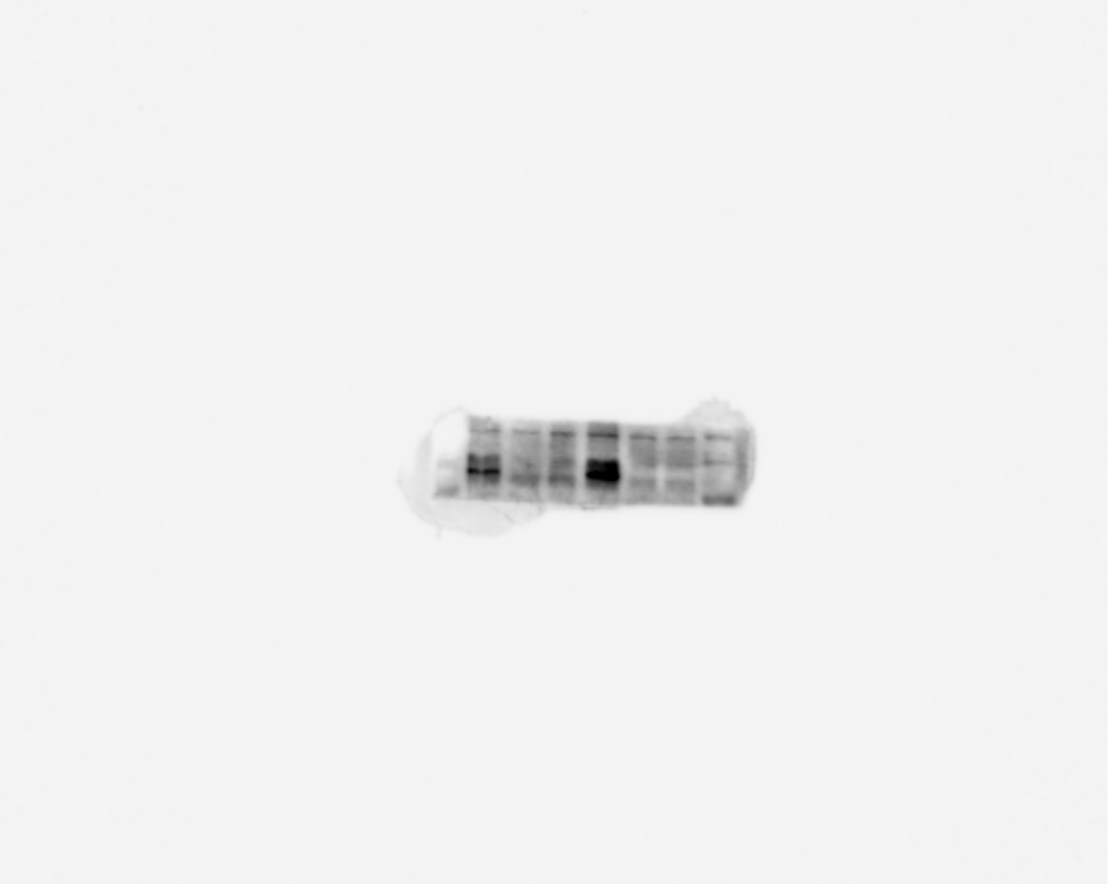

Fig. S7D
Actin

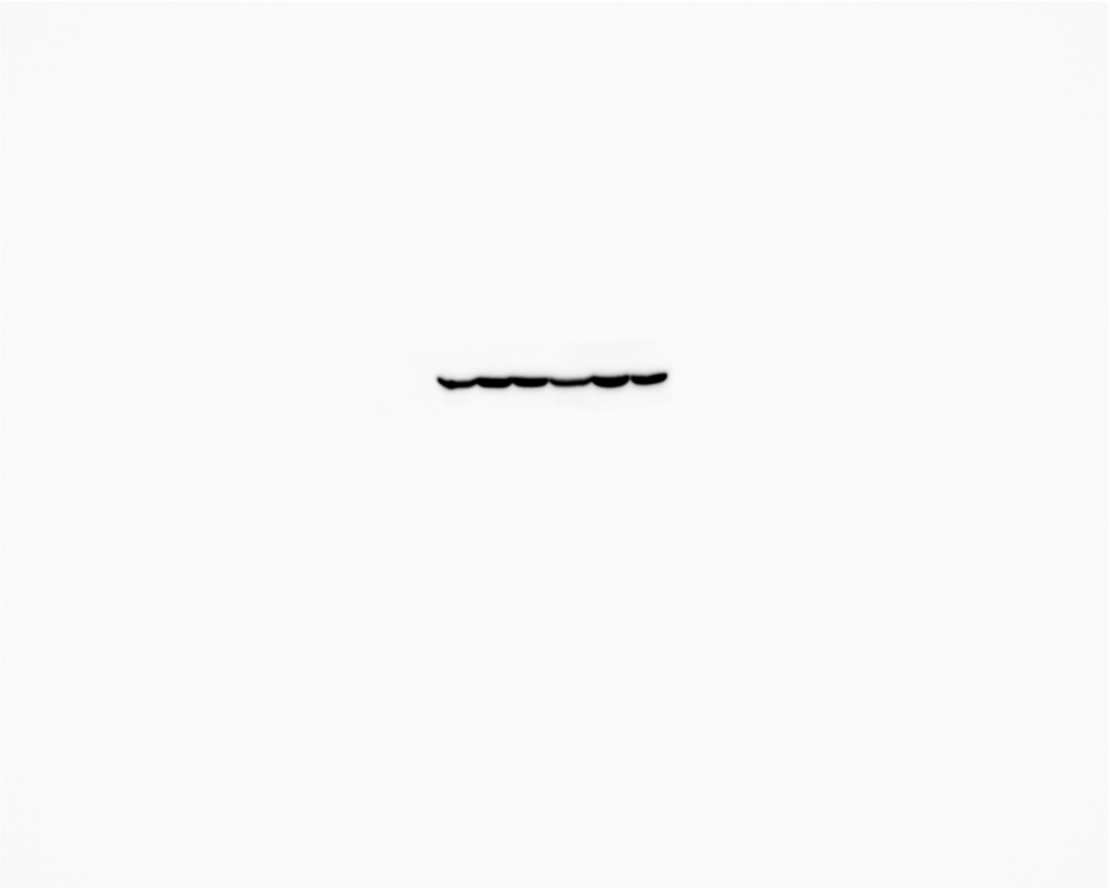

FKBP10
HIF2a

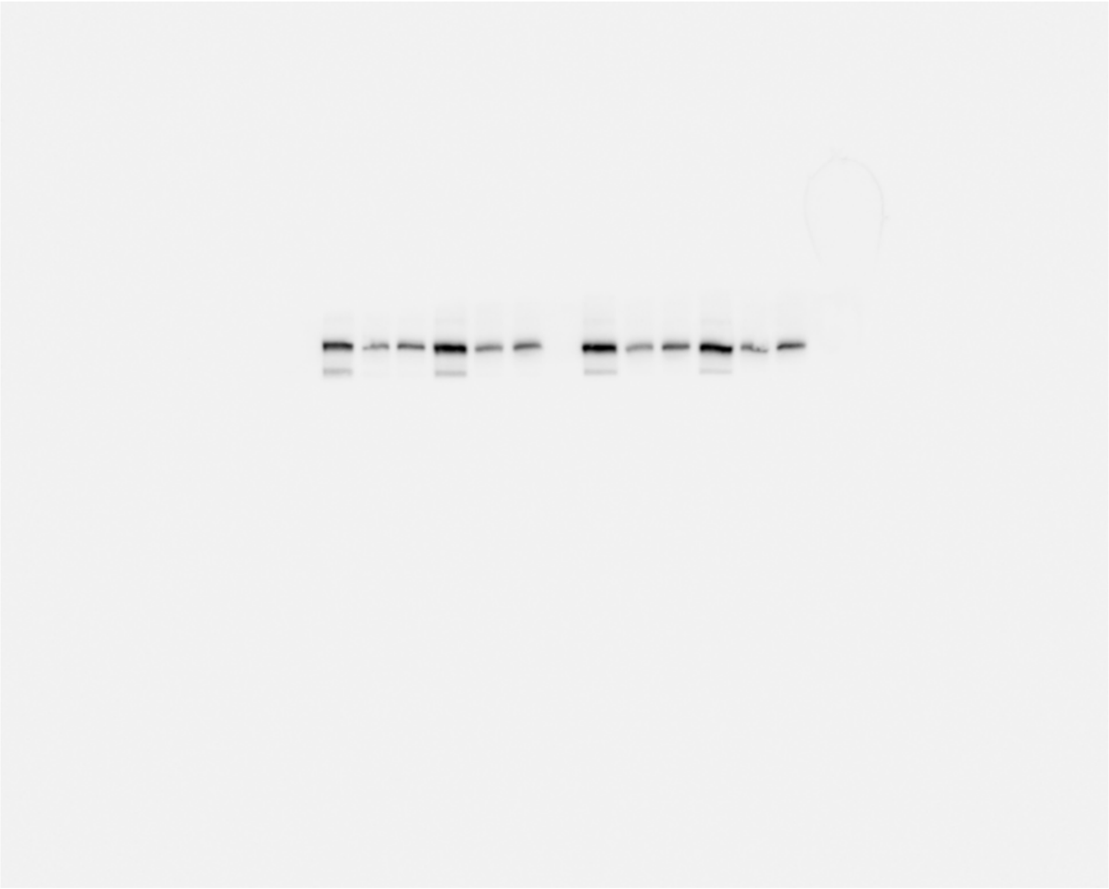

Fig. S7E
Actin

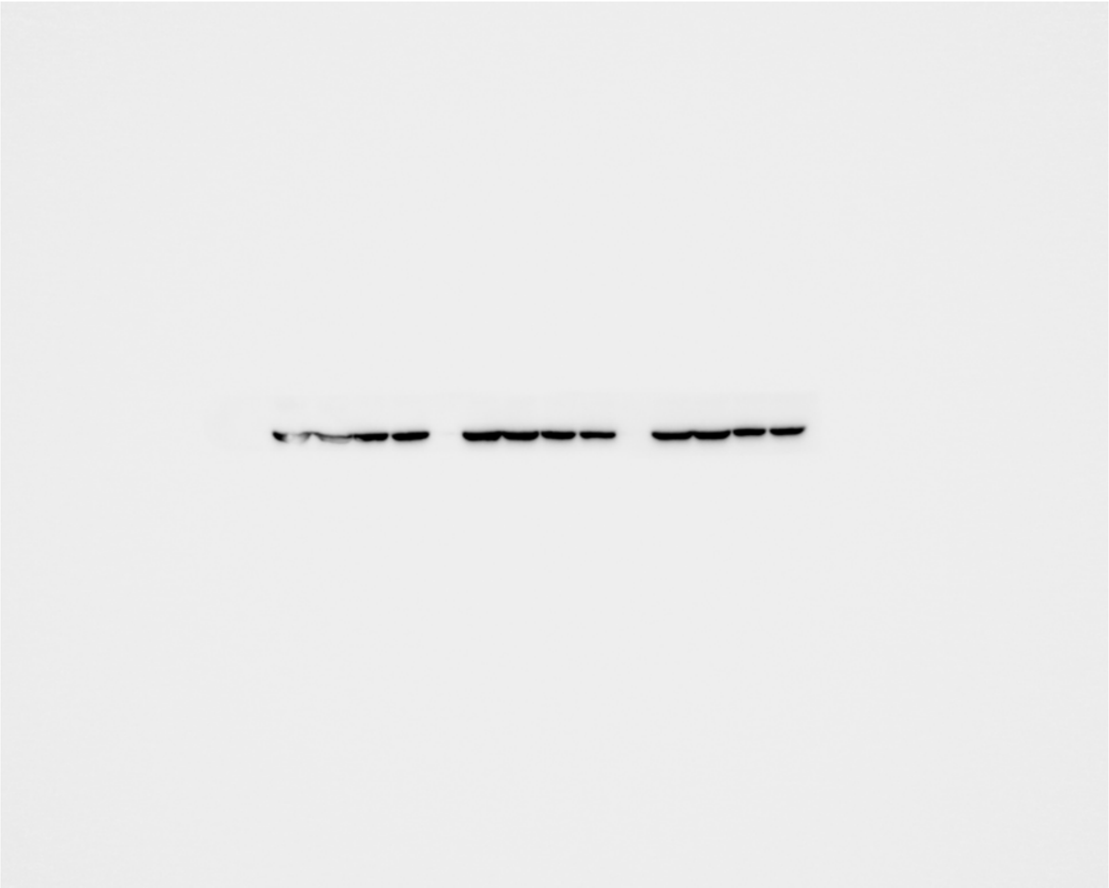

FKBP10

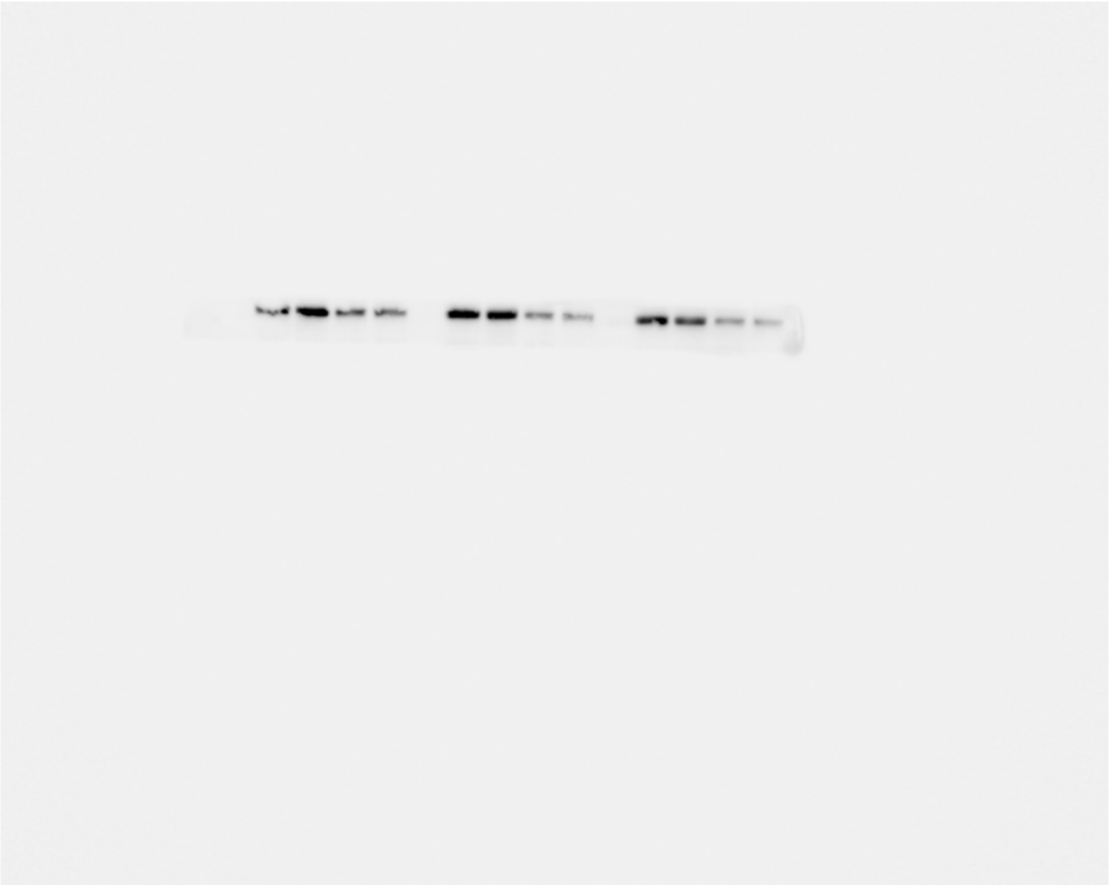

HIF1a

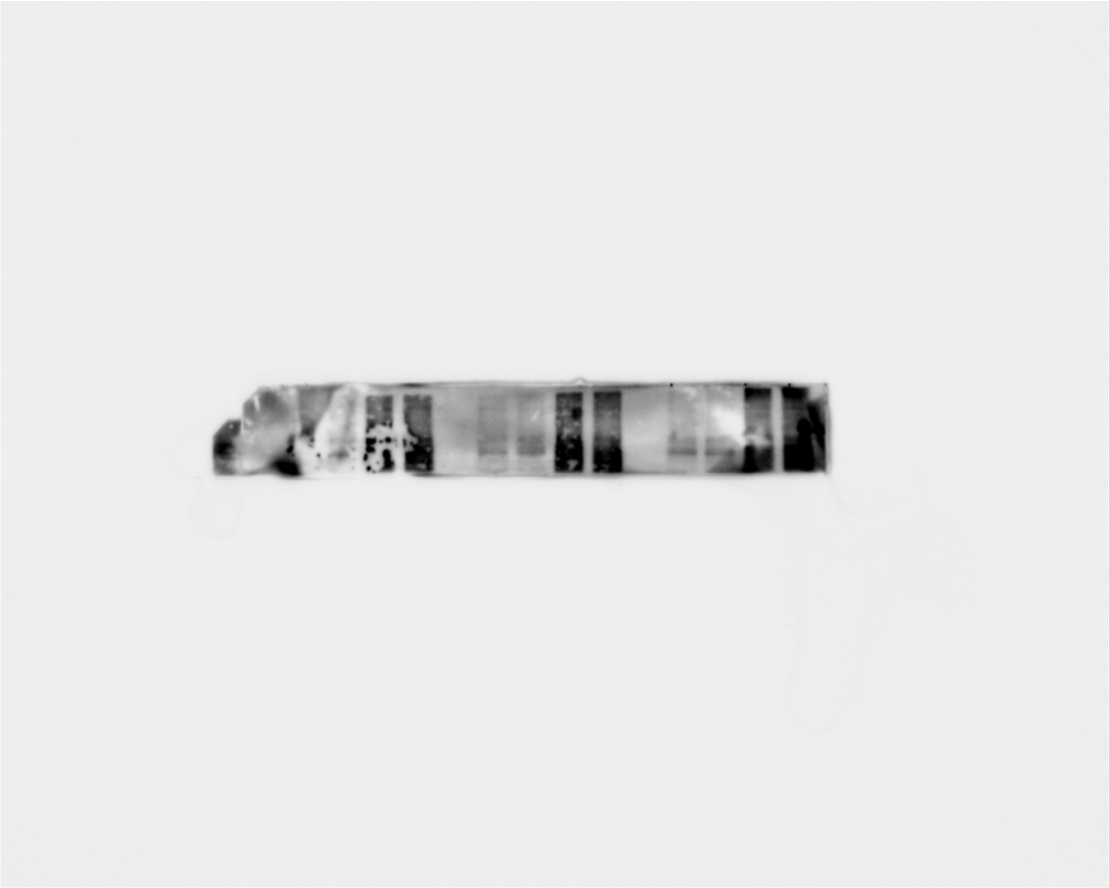

Supplement: Supplementary file 3 — Original Data File [file 41419_2024_6450_MOESM3_ESM.pdf]
